# Development of Predictive Models for Identifying Potential S100A9 Inhibitors Based on Machine Learning Methods

**DOI:** 10.3389/fchem.2019.00779

**Published:** 2019-11-25

**Authors:** Jihyeun Lee, Surendra Kumar, Sang-Yoon Lee, Sung Jean Park, Mi-hyun Kim

**Affiliations:** ^1^Department of Pharmacy, Gachon Institute of Pharmaceutical Science, College of Pharmacy, Gachon University, Incheon, South Korea; ^2^Gachon Advanced Institute for Health Science and Technology, Graduate School and Neuroscience Research Institute, Gachon University, Incheon, South Korea

**Keywords:** S100, machine learning, random forest, ligand-based virtual screening, feature selection, classification, consensus vote, Alzheimer's disease

## Abstract

S100A9 is a potential therapeutic target for various disease including prostate cancer, colorectal cancer, and Alzheimer's disease. However, the sparsity of atomic level data, such as protein-protein interaction of S100A9 with RAGE, TLR4/MD2, or CD147 (EMMPRIN) hinders the rational drug design of S100A9 inhibitors. Herein we first report predictive models of S100A9 inhibitory effect by applying machine learning classifiers on 2D-molecular descriptors. The models were optimized through feature selectors as well as classifiers to produce the top eight random forest models with robust predictability and high cost-effectiveness. Notably, optimal feature sets were obtained after the reduction of 2,798 features into dozens of features with the chopping of fingerprint bits. Moreover, the high efficiency of compact feature sets allowed us to further screen a large-scale dataset (over 6,000,000 compounds) within a week. Through a consensus vote of the top models, 46 hits (hit rate = 0.000713%) were identified as potential S100A9 inhibitors. We expect that our models will facilitate the drug discovery process by providing high predictive power as well as cost-reduction ability and give insights into designing novel drugs targeting S100A9.

## Introduction

Drug R&D is currently facing a productivity crisis to overcome low productivity as well as high risk/high return in the context of economics (Scannell et al., 2012; Mullard, 2014; Mignani et al., 2016; Bendtsen et al., 2017). In order to develop an efficient and cost-effective R&D process (Bendtsen et al., 2017), computing and simulations have decreased the traditional resource demand for drug R&D (Kapetanovic, 2008; Bendtsen et al., 2017). In particular, an early stage of drug discovery involves virtual screening (VS) to identify therapeutic targets or hit compounds (Walters et al., 1998; Bajorath, 2002; Oprea and Matter, 2004; Shoichet, 2004). Successful VS depends on the predictive power of predictors and the quality of the virtual library and dataset used. When the 3D-structure of a molecular target is available, structure-based virtual screening (SBVS) is considered prior to ligand-based virtual screening (LBVS) or SBVS/LBVS in combination due to an easy understanding of the predictive (atomic level) model and empirical evidence on bioactive conformation as well as the activity resulting from interaction between a target and a compound (Lavecchia and Di Giovanni, 2013; Sliwoski et al., 2014; Gadhe et al., 2015; Lavecchia, 2015; Jang et al., 2018; Lee et al., 2018; Yadav et al., 2018). Recently, the conceptual advance of drug targeting from “single target” to “protein-protein interactions (PPI),” it is unsatisfactory to obtain atomic level confidence of a novel druggable target with only partial structural information. It is therefore very difficult for researchers to propose a druggable binding site of a new molecular target for drug design without the background science or evidence. Therefore, when promising drug targets have insufficient information or when multiple targets need to be considered together, LBVS is commonly used, where the known active small molecules are used as screening templates. With improvement in the volume, quality, velocity, and accessibility of molecular data, versatile machine learning (ML) algorithms like support vector machine (SVM) (Cortes and Vapnik, 1995), Naïve Bayes (NB) (Domingos and Pazzani, 1997), decision tree (DT) (Breiman, 2017), and ensemble methods, such as random forest (RF) (Breiman, 2001) have contributed to the improvement of LBVS predictors (Geppert et al., 2010; Lo et al., 2018). With these advances, we can expect a diversity of training data like the heterogeneous property of activity index (or assay methods) and structural diversity beyond the congenericity of active compounds. In the case of classification models, selection methods for molecular descriptors (selectors) as well as classification algorithms (classifiers) decide the predictive power and coverage of models (Melville et al., 2009). Therefore, it is natural that multiple trials on various combinations of learning methods and feature sets coupled with raw dataset can facilitate the best performance of classifiers (Stahura and Bajorath, 2005; Domingos, 2012).

The S100 protein family is one of the challengeable drug target candidates (Donato, 2001; Ryckman et al., 2003). They are low molecular weight (ca. 100 amino acids) proteins with high similarity within the subfamily, and comprise two metal-binding EF-hands and a hinge. Due to their biophysical properties, they tend to form protein complexes (e.g., heterodimer like S100A8/S100A9, homodimer like S100B/S100B Donato, 1999), ligand-protein complex like S100A/RAGE complex (Yatime et al., 2016) rather than remaining as a single protein in a cell. Therefore, in the spite of many biological and pathological studies on several S100A9-mediated diseases, such as prostate cancer (Hermani et al., 2005), colorectal cancer (Kim et al., 2009), Alzheimer's disease (Horvath et al., 2015), and other neurodegenerative disorders (Gruden et al., 2017; Iashchishyn et al., 2018), atomic level knowledge is limited for SBVS or structure-based drug design of S100A9 inhibitors. Notably, the characterization of S100A9 complex has been updated, such as the hydrophobic binding of V-RAGE domain into S100A9 homodimer (Chang et al., 2016), V-RAGE domain into S100A9/S100A12 heterodimer (Katte and Yu, 2018) following the first X-ray report (Itou et al., 2002). However, the small molecule, *CHAPS* of the reports is a detergent (for protein stabilization or solubilizing) rather than a drug inducing functional change of S100A9. In addition, the SPR measurement of Q-compounds recently produces the question, whether the inhibition of Q-compounds is non-specific or specific (Björk et al., 2009; Yoshioka et al., 2016; Pelletier et al., 2018). Therefore, a ligand-based model can is required to compensate current insufficient characterization for targeting S100A9. For the purpose, maximum collection of the available data and selection of the most relevant features should be considered. Very delightfully, competitive inhibitors binding to S100A9 in the presence of the target receptors, such as RAGE, TLR4/MD2, and EMMPRIN (CD147) were reported in three patents (Fritzson et al., 2014; Wellmar et al., 2015, 2016). However, the patents proposed neither a druggable binding site nor different interaction mode between the target receptors. In other words, despite the presence of the inhibitors, no reliable predictive model has been reported to identify novel S100A9 inhibitors.

**Figure 1:**
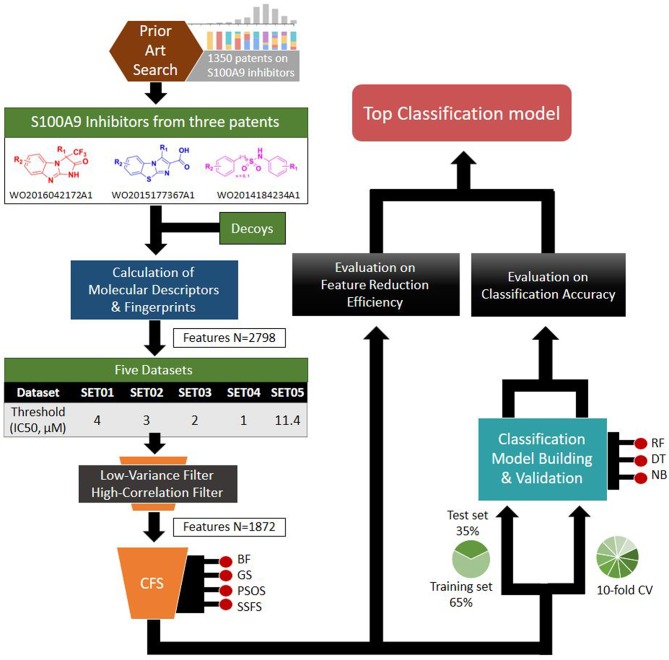
Workflow depicting the process of the top classification model development.

Based on the S100A9 competitive inhibitors of the patents, we present herein, the first predictive models using multi-scaffolds of competitive inhibitors (binding to the complex of S100A9 with rhRAGE/Fc, TLR4/MD2, or rhCD147/Fc) as a training set. For the purpose, highly efficient feature sets was considered in this study. Even though the input data matrix consisting of a low number of rows (data points/compounds) and a large number of columns (features) is never special in 2D/3D-QSAR or classification models built from limited and insufficient biological data (Guyon and Elisseeff, 2003; Muegge and Oloff, 2006), data processing (filtering, suitability, scaling) and feature selection were considered to remove irrelevant and redundant data (Liu, 2004; Yu and Liu, 2004). Adding a few other features to a sufficient number of features often leads to an exponential increase in prediction time and expense (Koller and Sahami, 1996; Liu and Yu, 2005), and whenever a large screening library is generated, feature generation of the library can be a practical burden. Further, because more irrelevant features hinder classifiers from identifying a correct classifying function (Dash and Liu, 1997), the feature optimization process is essential to increase the learning accuracy of the classifier and to escape the curse of dimensionality that emerge in a consequence of high dimensionality (Bellman, 1966). In addition, versatile machine learning models were built resulting from 5 × 4 × 3 trials: (1) five IC_50_ thresholds between activeness and inactiveness, (2) four feature selectors, and (3) three classifiers, thereby resulting in comprehensive validation of 60 models. The overall workflow depicted in Figure 1 was designed to select the optimal classification models with the best predictive ability and efficiency. In particular, we tried to gain a golden triangle between cost-effectiveness, speed, and accuracy. For this purpose, compact feature selection was critical for more than six million library screening showing the original data matrix of six million compounds (rows) × ca. 3,000 features (columns).

## Algorithms and Methods

### Datasets

**Figure 2:**
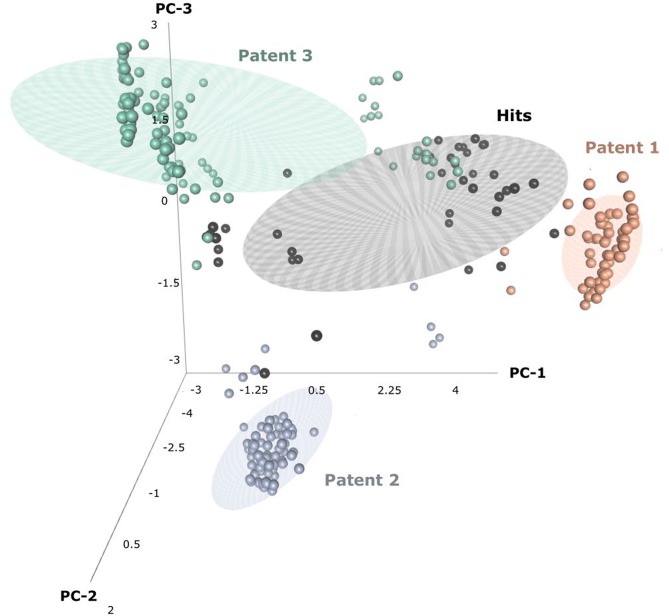
Three-dimensional principal component analysis(PCA) of hits and patent molecules. Patent 1, Patent 2, and Patent 3 refers to WO2015177367A1, WO2014184234A1, and WO2016042172A1.

Through patent searching, S100 inhibitors and their respective IC50 values were collected from three different patents. In the patents, even though the inhibitory effect on every complex (the binding complex of S100A9 with hRAGE/Fc, TLR4/MD2, or hCD147/Fc) was measured through the change of resonance units (RU) in surface plasmon resonance (SPR) (Fritzson et al., 2014), IC50 was calculated through the AlphaScreen assay of several concentrations in only biotinylated hS100A9 complex with rhRAGE-Fc (Fritzson et al., 2014; Wellmar et al., 2015, 2016). Therefore, the predicted inhibitory effect of our model means competitive inhibition of S100A9-RAGE in this study. The assay method for IC50 was identical in the three patents. The total number of molecules collected was 266: 115 compounds from WO2011184234A1, 97 compounds from WO2011177367A1, and 54 compounds from WO2012042172A1. The three distinct scaffolds led to the structural diversity of the dataset which was confirmed through the principal component analysis (PCA) of patent molecules (Figure 2). To investigate a more reasonable decision boundary between the activity and inactivity of the inhibitory effect on S100A9, five datasets (SET01, SET02, SET03, SET04, and SET05) were generated with different thresholds of activity (respectively 4, 3, 2, 1, and 11.4 μM of IC50). Insufficient numbers of inactive molecules were compensated by decoys from the DUD-E database (Mysinger et al., 2012), in order to obtain the same size for each dataset (*N* = 402), with a ratio of 66.17% (*N* = 266) patent molecules and 33.83% (*N* = 136) inactive decoy molecules (see Table S1 and Datasheet 1 in Supplementary Materials for SMILES information of the dataset). The activity property was converted to a binominal value according to the threshold of each set for a dichotomous classification. In particular, the activity threshold of 11.4 μM in SET05 is the highest IC50 value among patent molecules, thus making every patent molecule active, and every decoy molecule inactive in SET05.

### Descriptor and Fingerprint Calculation

Useful descriptors provide a better understanding of the molecules, and are widely used to construct models to predict certain molecular properties (Glover and Kochenberger, 2006). In our study, 2,798 features were generated using PaDEL-Descriptor ver. 2.21 (PaDEL-Descriptor, Pharmaceutical Data Exploration Laboratory) (Yap, 2011). All kinds of 1D and 2D descriptors were calculated to produce 1,444 features. The remaining features are from three kinds of fingerprints: MACCSFP, 166 bits; PubChemFP, 881 bits; SubstructureFP, 307 bits.

### Dimensionality Reduction

To avoid the curse of dimensionality and to enhance the efficiency of the overall predicting process, we applied several strategies to greatly reduce the number of features. Notably, each bit of fingerprint was considered as a single feature in our study; thus, the optimal feature set comprises hybrid fingerprints and descriptors after the feature reduction process. By removing irrelevant bits from the original intact fingerprint, a hybrid fingerprint can achieve increased prediction accuracy as well as reduced computational cost (Williams, 2006; Nisius and Bajorath, 2009, 2010; Singla et al., 2013; Smieja and Warszycki, 2016; Warszycki et al., 2017).

#### Low-Variance Filter and High-Correlation Filter

In our pre-processing step, we applied two steps of filtering: the low-variance filter and the high-correlation filter. First, to avoid redundancy, features with low variance were removed after normalization. Among 2,797 features, 724 columns with zero variance were removed (Table 1) to obtain a small feature set without reducing the prediction performance. Second, the correlation between two random variables was ranked to obtain Kendall's Tau-a coefficient matrix. Features with strong dependency (τ > 0.9) were removed to ensure maximum dissimilarity between features (Ding and Peng, 2005). Here, 201 columns were removed, leaving 1,872 independent features that were de-normalized for further processing (Table 1).

**Table 1:** The number of features.

	**Initial features**	**Low-variance filter**	**Low-variance filter and high-correlation filter**
1D/2D descriptors	1,444	1,218	1,017
Fingerprints	1,354	855	855
MACCSFP	166	147	147
PubChemFP	881	598	598
SubstructureFP	307	110	110
Total	2,798	2,073	1,872

*The numbers of descriptors and bits of fingerprints generated initially, and the selected numbers of features after the removal of unnecessary features by certain filtration methods are listed. Note that each bit of a fingerprint was considered as a single feature*.

#### Correlation-Based Feature Subset Selection

In addition to the correlation filter, we used a correlation-based feature subset selection method (Hall, 1999) to obtain a compact number of features. Merit, composed of Pearson's correlation formula, is used to evaluate the correlation-based feature selection (CFS) algorithm. To determine subsets containing features that are highly correlated with the class but are uncorrelated with each other, the following merit is calculated along a search:

(1) $$\begin{array}{r} Merit_{s}=\frac{k\bar{r_{cf}}}{\sqrt{k+k\left(k-1\right)\bar{r_{ff}}}} \end{array}$$

where *Merit*_*s*_ is the heuristic merit of subset *S* containing *k* features, $\bar{r_{cf}}\;$is the average correlation with the class, and $\bar{r_{ff}}$ is the average inter-correlation. The subset with the highest merit is selected to obtain features with high predictive ability and low redundancy. Various search algorithms are applicable for improving the efficiency of feature selection (FS) methods. Herein we applied four different search algorithms: best first, genetic search, particle swarm optimization search, and subset size forward selection. To assess the effectiveness of the FS methods, two measurable indexes were selected: the rate of feature reduction and the merit of the best subset found. All calculations were performed in Weka software packages (Weka Environment for Knowledge Analysis ver. 3.6, The University of Waikato, Hamilton, New Zealand) (Hall et al., 2009).

#### Best First (BF)

Best first search is one of general algorithms for exploiting heuristic information to reduce search times. The general strategy assesses the merit of every candidate feature set exposed during the search, and then continues exploration along the direction of the highest merit (Kohavi and John, 1997). In our study, the search was terminated when an improved node was not found in the last 5 expansions. Also, backtracking was applied to reduce the size of the search space and to allow the algorithm to move toward a more promising subset (Freuder, 1988). Because the running times for the backward search starting from the full set of features could render the approach infeasible, especially if there are many features, forward selection was applied here to achieve cost-effectiveness.

#### Genetic Search (GS)

The genetic algorithm was first introduced by John Holland (Holland, 1992), and David Goldberg presented an application in 1989 (Goldberg, 1989) that triggered a wide variety of modifications and developments to genetic algorithms (Glover and Kochenberger, 2006). Genetic algorithms derive their name from the fact that they are inspired by the mechanism of natural selection, where the fittest individuals survive to the following generations (Man et al., 1996). Although the search method using genetic theory may result in higher computational costs than other methods, such as best first, it remains popular, because it is relatively insensitive to noise and is well-suited for problems where little knowledge is provided (Vafaie and De Jong, 1992). In this study, the total number of generations was 20, with 20 feature subsets in each generation. The probability of crossover and the mutation rate were set to 0.6 and 0.33, respectively.

#### Particle Swarm Optimization Search (PSO)

Particle swarm optimization (PSO), suggested by Kennedy and Eberhart in 1995 (Eberhart and Kennedy, 1995), is based on social-psychological principles. Because only a few lines of code and primitive mathematical operators are required, this method has been proved to be highly efficient for application to numerous areas (Shi, 2001). Herein we utilized the geometric particle swarm optimization (GPSO) (Moraglio et al., 2007), where a convex combination was applied to update the positions of particles. In GPSO, three convex weights *w*_1_, *w*_2_, and *w*_3_ are employed, where *w*_1_, *w*_2_, *w*_3_ > 0 and *w*_1_+*w*_2_+*w*_3_ = 1. The function of GPSO can be defined as:

$$\begin{array}{l} x_{i}=CX\left(\left(x_{i},w_{1}\right),\left(ĝ,w_{2}\right),\left(\hat{x_{i}},w_{3}\right)\right) \end{array}$$

where ĝ is the global optimum and $\hat{x_{i}}$ is the local optimum. Each convex weight represents the inertia weight (*w*_1_), social weight (*w*_2_), and individual weight (*w*_3_), which were set to 0.33, 0.33, and 0.34, respectively in our study. The number of particles in the search space and the number of populations in each generation were both set to 20.

#### Subset Size Forward Selection (SSFS)

Subset size forward selection (SSFS) is an extension of linear forward selection. Through this method, a compact feature set can be obtained from large-scale features with a relatively small number of instances (Gutlein et al., 2009). The optimal size was determined through 5-fold cross-validation with fixed-set linear forward selection, resulting in a reduced error compared to searching in a single training and test set. The number of top-ranked features forming a search space was set to 50.

### Machine Learning Classifiers

After selecting the optimal feature sets, three different classifiers (decision tee, random forest, and naïve Bayes) were applied to develop and determine the best classification model for S100 inhibitors. All ML processes and calculations were performed using the KNIME software.

#### Decision Tree (DT)

The decision tree classifier is a simple and widely comprehensive method that can be constructed relatively quickly compared to other well-known classifiers (Kotsiantis et al., 2007). The scalable parallelizable induction of decision trees (SPRINT) (Shafer et al., 1996), a modified form of the well-known C4.5 (Quinlan, 2014), was applied in this study so that the model can take a large-scale database as an input. The Gini index was measured to determine the root node, which is the best feature that divides the dataset. To avoid overfitting problems, we applied both pre-pruning and post-pruning strategies. For post-pruning process, minimum descriptor length (MDL) pruning was applied here (Rissanen, 1978).

#### Random Forest (RF)

Random forest (Breiman, 2001) was developed by introducing bootstrap aggregating to decision tree. Trees are built with randomly sampled features to form a forest, and the most voted tree is selected as the optimal classifier. This ensemble learning method can handle high-dimensional data with numerous features. In addition, it is less susceptible to noise and builds a robust model, often outperforming other classifiers (Verikas et al., 2011; Khuri et al., 2017). In this study, features were evaluated based on the information gain ratio to obtain the best splits.

#### Naïve Bayes

Along with decision trees, naïve Bayes is one of the most popular machine learning methods for classification models. Unlike the canonical Bayesian method, naïve Bayes assumes that all features are independent of each other. Although this “naïve” assumption rarely fits in practice, it has been verified to perform reasonably well in various situations, without the requirement of independence between features (Domingos and Pazzani, 1997). This occurs because the strong false assumption may lead to reduced overfitting (Domingos, 2012). Another advantage of this method is its simplicity and low computational cost (Kotsiantis et al., 2007), which allows one to search in very large databases with high efficiency.

### Parameter Optimization

To achieve the best performance, several parameters were optimized prior to the development of predictive models. For each optimization, 10-fold cross-validation was performed with the training set (65% of the original dataset), where the optimal parameters exhibiting the largest area under the curve (AUC) of receiver operating characteristics (ROC) curves were exported to construct the model. The optimized parameters and AUC values are listed in Table 2.

**Table 2:** The optimized parameters for random forest models and the AUC of ROC values of test set prediction.

**Applied feature selector**	**Dataset**	**maxDepth^**a**^**	**numTrees^**b**^**	**AUC of ROC**
BF	SET01	5	52	0.971
	SET02	6	42	0.961
	SET03	10	215	0.956
	SET04	10	203	0.912
	SET05	2	15	1
GS	SET01	3	82	0.932
	SET02	10	164	0.935
	SET03	10	112	0.948
	SET04	6	105	0.867
	SET05	3	36	1
PSOS	SET01	8	76	0.952
	SET02	5	45	0.915
	SET03	9	185	0.952
	SET04	10	84	0.882
	SET05	4	13	1
SSFS	SET01	7	97	0.967
	SET02	6	59	0.966
	SET03	9	236	0.963
	SET04	8	245	0.896
	SET05	2	50	1
None	SET01	5	25	0.954
	SET02	7	96	0.941
	SET03	7	124	0.949
	SET04	7	60	0.872
	SET05	5	79	1

### Model Validation

To assess the prediction performance of the models, two validation methods were employed: (i) evaluation by test set (35% of the original dataset) and (ii) 10-fold cross-validation of the training set. The AUC of ROC curve and the Matthews correlation coefficient (MCC) were calculated to obtain the top models among every combination of feature selectors and ML classifiers. The MCC value can be defined as:

$$\begin{array}{l} MCC=\frac{TP\times TN-FP\times FN}{\sqrt{\left(TP+FP\right)\left(TP+FN\right)\left(TN+FP\right)\left(TN+FN\right)}}, \end{array}$$

where TP, TN, FP, and FN refers to true positive, true negative, false positive, and false negative.

### *In silico* Screening of the eMolecules Database

Over past decade, large drug discovery companies have been actively applying high-throughput screening (HTS) to search potent hit molecules (Stahura and Bajorath, 2004; Reddy et al., 2007). However, HTS often demands prior validation and preparation time as well as great expense and facilities. To aid or complement HTS, the VS method should have the ability to select only a small number of potent molecules from a huge database. Thus, the top models that we have chosen previously were further evaluated by *in silico* screening of a large-scale dataset (*N* = 6,447,184) from the eMolecules database (http://www.emolecules.com/).

#### Screening Library

eMolecules provides almost eight million unique compound structures along with the information of vendors of the respective molecules assembled from more than 150 suppliers and manufacturers (Williams, 2008). Many studies have successfully discovered potential hits by screening molecules from this database (Bisignano et al., 2015; Lenselink et al., 2016; Shehata et al., 2016). In our study, 6,447,184 molecules were collected for screening, and split into 33 subsets in order to reduce computing (memory) burdens (Table 3). Features of each subset molecules could be generated based on the optimal features of the top models. First, the upper class of necessary descriptors were calculated, because only the upper class of descriptors can be selected rather than each single feature in PaDEL-Descriptor. Then, using KNIME software, the features needed were chosen to generate the exact same kind of feature set, which was used in top model building.

**Table 3:** The number of molecules from eMolecules database in each subset.

**Subset**	**Number of molecules**
Subset01–subset09	100,000
Subset10–subset18	200,000
Subset19–subset32	250,000
Subset33	247,184
Total	6,447,184

#### Prediction and Identification of Hits

Each subset with each respective feature set was then used as an input to the random forest predictor, which was built through the learning of patent molecules. Then, the predictor was used to assign possibility as S100A9 inhibitors among the screened molecules. Only molecules with a higher probability than 0.9 of being active than were selected. Overlapped molecules from the consensus of eight top models were collected to obtain the final hits.

#### Prediction of ADME Properties

Since poor pharmacokinetic profiles and high potential of toxicity are one of the main reasons of failure in drug development, it is crucial to consider such absorption, distribution, metabolism, excretion (ADME) properties in advance to encourage further assays and clinical trials of final hits. Thus, we predicted several drug-likeness and ADME properties of hit molecules using the QikProp module of Maestro 11.4 (Schrodinger Release 2017-4: QikProp, Schrödinger, LLC, New York, NY, 2017). QikProp computes pharmaceutically relevant properties of molecules to help eliminate those with unsatisfactory ADMET profiles. Here, we generated computational properties to ensure the drug-likeness of hits, including molecular weight (MW), LogP, hydrogen bond donor, hydrogen bond acceptor, number of N and O, polar surface area (PSA), and violation of Lipinski's rule of five as well as Jorgensen's rule of three. Also, the apparent Caco-2 cell permeability and MDCK cell permeability was also calculated to investigate intestinal absorption and oral absorption abilities.

## Results and Discussion

### Reasonable Compression of Features for Predictive Models

In order to compare the performances before and after FS, we could consider predictive power and cost-effectiveness. The efficiency of each feature selection method was evaluated by calculating two measurements: the rate of feature reduction, and the merit.

#### Feature Reduction

**Figure 3:**
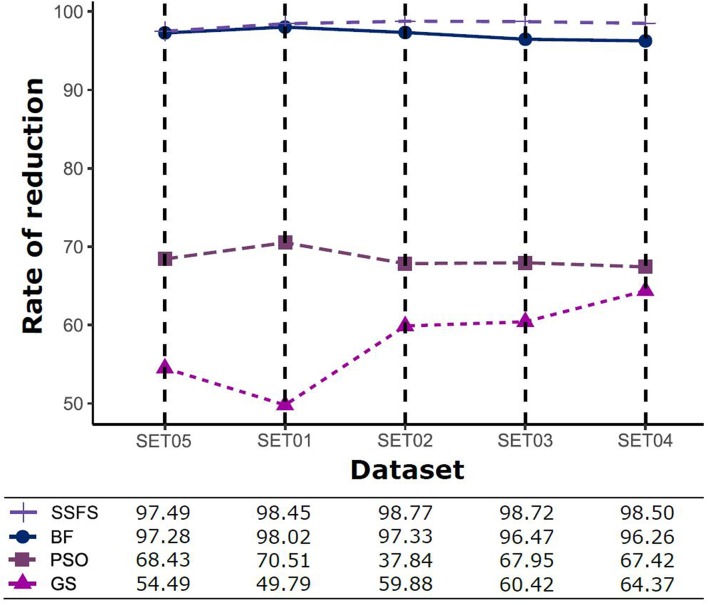
The rates of feature reduction. The reduction rate is the ratio between the number of features removed after FS method and the original number of features before FS method.

Feature reduction can play an important role in model building due to its ability to greatly reduce computational burden and to increase classification accuracy. Herein the cost-reducing effect of each FS method was evaluated through feature reduction ability. After two serial filtrations which removed 926 features from 2,798 original features, we applied CFS with four different search methods to further obtain a compact and optimal feature sets. The reduction ability of each FS method was evaluated and compared to determine optimal approaches. The selected number of features after each FS method is presented in Table 4. The rates of feature reduction are also shown in Figure 3, which are the number of excluded features divided by the number of features before CFS.

**Table 4:** The number of selected features after each FS method.

	**BF**	**GS**	**PSO**	**SSFS**
SET05	51	852	591	47
SET01	37	940	552	29
SET02	50	751	602	23
SET03	66	741	600	24
SET04	70	667	610	28

**Figure 4:**
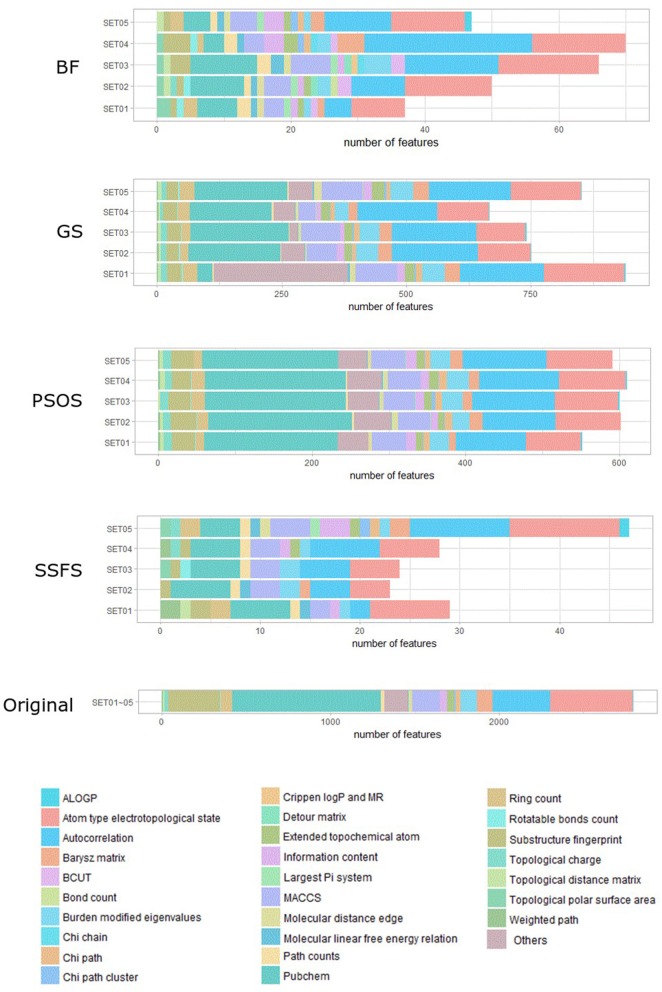
The composition of each feature set. The number of each kind of descriptor and fingerprint bit after each FS method is shown here. SET0N refers to the different IC50 threshold (SET01:4 μM; SET02:3 μM; SET03:2 μM; SET04:1 μM; SET05:11.4 μM). Note that the maximum value of horizontal axis of the graph differs between each FS method.

As shown in Table 4 and Figure 3, BF and SSFS excluded most of the features with over 96% removal in all five datasets. However, a relatively high number of features remained after GS or PSO, and especially, GS showed the least consistency between subsets (49.786%~64.37%). When comparing between BF and SSFS, the actual number of features is less through SSFS than BF, yet the rates of reduction are similar. In SET03, the number of features remaining after SSFS was 36.36% (*N* = 24) of that after BF (*N* = 66). Thus, SSFS is expected to achieve the greatest effectiveness regarding cost reduction, and since the number of features selected is also small enough in BF, it is also expected to have a high efficiency similar to SSFS. The composition of each feature set is shown in Figure 4. See Table S2 in Supplementary Materials for detailed information of the selected features. Due to the large number of original features, autocorrelation (e.g., ATS, AATS, ATSC), Pubchem fingerprint (e.g., PubchemFPxxx), and atom type electrotopological state (e.g., SpMax1_Bhm) could also show the highest relative frequency ratio among 63 descriptor types of 2,798 original features. In addition, with the three type descriptors, burden modified eigenvalues, molecular linear free energy relation, path count, MACCS fingerprint, and substructure fingerprint were commonly chosen through four FS methods. Because fragmented fingerprints and burden modified eigenvalues have relatively large number of original features (96–489 features), molecular linear free energy relation (with 6 features) and path count (with 22 features) are more impact per feature than other descriptors but the descriptors could not exist in every subset (5 subsets × 4 FS method).

#### Merit

**Figure 5:**
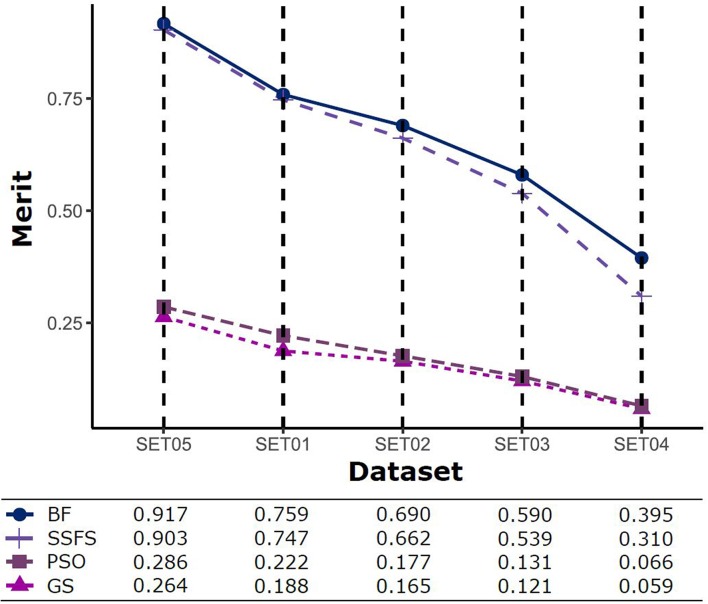
The merits of feature sets after feature selection method in each dataset with different IC50 threshold.

The predictive performance of a model strongly depends on the usefulness of the features. After feature selection, the remaining features may not fully represent the original features. Therefore, the merit of a feature set is measured as shown in Figure 5 to determine which FS method produce the best discriminative ability for model building. Despite this ability, the merit value itself does not consider the size of the dataset and a standard of a “high enough” merit value cannot be defined. Only a comparison between methods with the same dataset is valid and therefore, as described later, we further examined the effects on classification accuracy. A general observation is that the merit improved with an increase in the activity threshold. When every compound from a same resource is classified into the same class, it seems that the merit value tends to be enhanced, as shown in Figure 5, where the merit was the highest in SET05 among all datasets. The merits of BF and SSFS were higher than those of GS and PSO in every dataset, although they decreased rapidly (0.917–0.395 and 0.903–0.31, respectively) as the range of activity narrowed. GS and PSO selected feature sets with relatively poor merits, lower than 0.3 in every dataset, and almost near to zero in SET04. The results indicate that BF and SSFS achieve efficiency as well as enhance the predictive ability of the model, whereas GS and PSO barely improve the prediction ability.

### Evaluation of Classification Performance

To assess the performance of the classification models, two validation approaches, external validation using the test set (35% of initial dataset) and internal 10-fold cross-validation, were used to acquire the AUC of ROC curve and MCC. Effectiveness of 5 × 3 × 4 models: (1) five type activity thresholds between activeness and inactiveness, (2) three FS methods (selectors), and (3) four ML methods (classifiers) were evaluated. The 60 models were also compared with the models without a CFS process as control groups to evaluate the effectiveness of FS on the classification performance. Mean values of measurements in each dataset were calculated to better focus on the comparison between FS methods. The control group, where FS was not treated, is labeled as “none.” Every dataset used in all models contain identical molecules but differently assigned activity.

#### AUC of ROC

**Figure 6:**
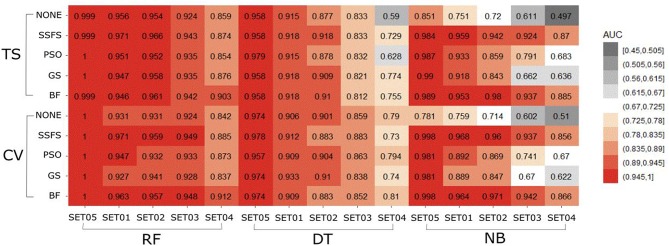
Heat-map depicting the AUC of ROC curve of the classification models.

The AUC values of ROC curve of each model are illustrated in Figure 6. Generally, AUC declined as the IC50 threshold narrowed. Nevertheless, the RF models produced the highest AUC values in all combinations of activity thresholds and the FS methods in both external test set validation and 10-fold cross-validation. On the other hand, the AUC values were dramatically reduced as the activity threshold narrowed in NB or DT models, especially when built without feature selection process. This indicates that RF models have the most robust predictive ability among classifiers, showing a constantly high AUC ranging from 0.859 to 1 and from 0.839 to 1 in test set validation and cross-validation, respectively. Regarding FS methods, BF or SSFS exhibited relatively higher AUC than PSO or GS, as well as none (without CFS methodology). In addition, they produced the highest AUC when built with the RF classifier. The NB models appears to get the largest benefit from BF and SSFS methods, achieving substantial increase compared to the model without CFS process. However, GS or PSO methods could not greatly enhance the AUC values of NB models, producing only a slight increase compared to the model built without them, especially when the activity threshold was low. This suggests that RF models built with BF or SSFS feature selection methods have strong possibility to be the optimal model and exhibit the greatest robustness.

#### MCC

**Figure 7:**
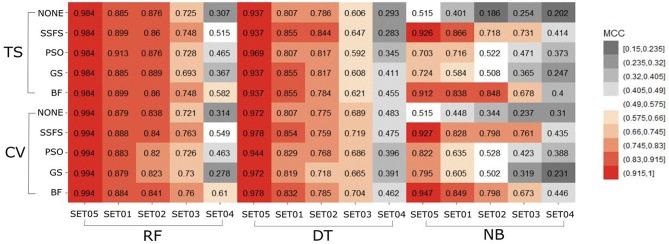
Heat-map depicting the MCC of the classification models.

In general, the MCC values exhibited similar tendencies to the AUC (Figure 7). Here also, RF achieved the highest MCC for every combination except for the cross-validation result of models applying GS or no feature selector with SET04. The overall MCC values of RF classifier with other datasets except for SET04 were reasonably high, ranging from 0.693 to 0.984 in external test set validation, and from 0.721 to 0.994 in 10-fold cross-validation. Among FS methods, BF and SSFS also achieved the best performance for all combinations. In particular, they exhibited enhanced MCC values when combined with the RF classifier. On the other hand, the NB classifier with the GS or PSO feature selector exhibited considerably lower values compared to other methods, and a rapid decline could be seen as the IC50 threshold narrowed. Even when combined with BF or SSFS, the NB models resulted in relatively low MCC compared to the RF or DT models.

In summary, “RF classifier + BF selector” or “RF classifier + SSFS selector” under their optimal hyperparameters presented the best predictive ability. Obviously, RF was more distinguished than other classifiers with a robust performance in all IC50 thresholds. BF and SSFS enhanced the classification performance, obtaining higher AUC and MCC values than other selectors. It is thus observed that the IC50 activity threshold has non-negligible influences on prediction performance. As the threshold narrowed, the accuracy and MCC values declined without any exception, implying the toughness of distinguishing between patent molecules with low IC50 values. Nevertheless, models built with low activity threshold may lead to the discovery of highly potent molecules selectively. Among all IC50 thresholds (SET01 to SET05), 1 μM (SET04) was excluded to generate the Top models: four IC50 activity thresholds (11.4, 4, 3, and 2 μM) and two feature selectors (BF and SSFS) under the optimal RF classifier. Through the consensus vote of the top 8 models, potential S100A9 inhibitors could be obtained.

#### Quality, Cost, and Effectiveness of Screening Hits

**Figure 8:**
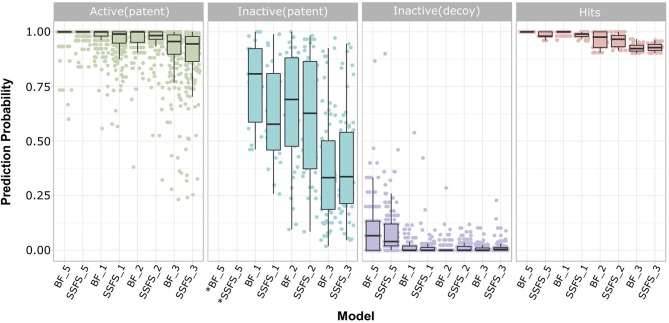
Prediction probability of top 8 models on patent molecules, decoy molecules, and hits. Each label of horizontal axis represents each top random forest model. The FS method and the dataset (SET0N) used in the model is indicated as “FS method_N”. For example, SSFS_3 refers to the random forest model built with the feature set chosen by SSFS with dataset SET03. *Note that every patent molecules were considered as active in BF_5 and SSFS_5.

Ligand-based virtual screening was performed using a large-scale dataset (*N* = 6,447,184) derived from the eMolecules database. We finally obtained 46 potential S100A9 inhibitors through unanimous votes from top models (hit rate = 0.000713%). The 2D structures of hits are presented in Table S3. Notably, the prediction probabilities of selected hits were similarly high compared with patent molecules, ranging from 0.902 to 1 with little differential between models (Figure 8). In order to qualify the hit compounds, their structure novelty also was evaluated. For this purpose, the Tanimoto similarity between each hit compound and the nearest neighbor was presented (Table 5).

**Table 5:** Drug-likeness, ADME parameters prediction for 46 hits using QikProp and their Tanimoto similarity between the nearest neighbor.

**Molecule index**	**MW^a^**	**LogPo/w^b^**	**dHB^c^**	**aHB^d^**	**No. N&O^e^**	**PSA^f^**	**Lo5^g^**	**Jo3^h^**	**Caco2^i^**	**MDCK^j^**	**Sim^k^**
1	438.81	4.64	2	5.25	6	83.28	0	1	883.32	6500.64	0.723
2	447.52	1.88	1	10.25	8	110.03	0	0	169.49	217.42	0.733
3	475.58	2.45	1	10.25	8	107.08	0	0	212.80	254.04	0.725
4	459.55	3.35	2	9	7	109.44	0	1	252.94	277.07	0.709
5	357.35	2.21	1	6.5	6	79.76	0	0	662.83	1251.4	0.803
6	475.54	2.06	2	12	10	152.60	0	0	74.61	50.07	0.686
7	369.37	2.66	2	5.5	7	112.07	0	0	123.62	120.63	0.823
8	489.56	2.35	2	12	10	152.60	0	1	74.59	50.05	0.685
9	463.57	2.06	3	9.5	9	138.29	0	0	31.69	26.88	0.763
10	399.25	2.08	1.25	7.75	7	102.73	0	0	310.52	651.41	0.831
11	394.81	2.23	1.25	7.75	7	102.66	0	0	338.55	628.45	0.757
12	376.82	1.93	1.25	7.75	7	103.25	0	0	371.75	355.25	0.767
13	394.81	2.10	1.25	7.75	7	103.88	0	0	336.23	499.24	0.757
14	449.54	2.32	3	9.5	9	134.36	0	0	78.22	41.92	0.711
15	463.57	2.57	2	9.75	8	110.80	0	0	245.98	297.80	0.708
16	396.39	3.56	1.25	5.25	6	93.24	0	1	414.29	1915.26	0.727
17	378.42	0.73	3	10	8	136.31	0	0	55.49	35.38	0.747
18	408.45	0.61	2	11.75	10	140.34	0	0	89.51	46.28	0.738
19	388.46	2.35	2	6.5	7	120.67	0	0	135.00	195.05	0.633
20	392.47	3.46	2	5.25	6	88.54	0	0	274.13	938.46	0.663
21	379.41	0.81	3	9.25	8	135.31	0	0	48.47	31.86	0.833
22	488.49	4.53	1	8.7	8	90.37	0	2	1243.59	3476.5	0.697
23	374.43	3.01	2.25	5.75	6	96.24	0	0	317.78	825.75	0.718
24	376.86	2.93	2.25	5.75	6	95.44	0	0	309.55	1326.69	0.721
25	376.86	2.92	2.25	5.75	6	95.45	0	0	344.43	1241.09	0.721
26	360.41	2.61	2.25	5.75	6	94.32	0	0	309.07	1006.01	0.721
27	424.44	3.73	2.25	5.75	6	98.39	0	0	240.41	1826.54	0.704
28	410.41	3.34	2.25	5.75	6	94.30	0	0	309.14	2445.21	0.706
29	394.81	1.97	1.25	7.75	7	101.41	0	0	336.23	484.57	0.757
30	397.52	2.51	2	8	6	90.69	0	0	823.95	875.59	0.753
31	378.46	2.15	2	7.7	7	110.06	0	0	286.99	254.97	0.776
32	382.82	2.67	1	6.75	8	111.06	0	0	226.09	236.12	0.783
33	427.46	0.74	1	11	10	129.95	0	0	146.33	120.44	0.747
34	410.41	2.18	2	9	7	115.79	0	0	223.42	411.72	0.744
35	410.41	2.15	2	9	7	116.40	0	0	182.76	361.39	0.744
36	357.79	1.93	2	7	6	92.65	0	0	276.14	510.66	0.828
37	357.79	1.93	2	7	6	93.28	0	0	259.64	489.39	0.828
38	357.79	1.86	2	7	6	93.79	0	0	240.46	428.65	0.828
39	371.81	2.37	1	7.5	6	81.06	0	0	544.41	1089.44	0.780
40	374.43	3.67	1.25	5.75	6	84.30	0	1	922.94	2328.24	0.750
41	370.79	1.97	2	6.5	7	111.17	0	0	122.26	206.45	0.759
42	399.87	3.14	1	7.5	6	82.38	0	0	625.21	1265.93	0.791
43	412.80	2.35	1.25	7.75	7	101.92	0	0	323.45	793.95	0.757
44	398.40	4.57	2	4.5	6	80.90	0	1	1275.28	3912.14	0.671
45	379.42	2.24	2	8.5	6	101.90	0	0	319.42	418.38	0.759
46	348.39	0.82	2	9.5	7	114.57	0	0	141.61	101.70	0.783
Standard value^l^	130.0–725.0	−2.0–6.5	0.0–6.0	2.0–20.0	2–15	7.0–200.0	Maximum is 4	Maximum is 3	<25 poor, >500 great	<25 poor, >500 great	

a *Molecular weight*.

b *Octanol/water partition coefficient*.

c *Number of HB donors*.

d *Number of HB acceptors*.

e *Number of N and O atoms*.

f *Polar surface area*.

g *Number of violation of Lipinski's rule of five*.

h *Number of violation of Jorgensen's rule of five*.

i *Apparent Caco-2 cell permeability (nm/s)*.

j *Apparent MDCK cell permeability (nm/s)*.

k *Tanimoto coefficient of the entry between the nearest neighbor among 266 active molecules from patents*.

l *Standard values from 95% of known drugs based on results of Qikprop*.

In the view of structural novelty, our virtual screening could certainly guarantee similarity, such as the level of recent generative model-based *de novo* design (Popova et al., 2018). Our hits not only retain the structural diversity of active molecules, but also exhibit differentiation from patents, thereby suggesting our models' ability to elicit novel S100A9 inhibitors (Figure 2). Furthermore, our model is economical in the view of cost. The overall screening process including feature generation of the 6 M size library took ca. 161 h under 1 CPU and 8 GB memory condition for being to show 40 times faster than the screening using S100A9 docking models in the same computing resource. It proved strong cost-reduction ability and efficiency enough to apply to the real-world drug R&D.

In sequence, binding mode of the hit compounds was compared with known S100A9 inhibitors, 266 dataset under in-house docking model. For the docking simulations, homodimer of the mutant S100A9 (C3S) was gain from PDB 5I8N code (Chang et al., 2016). The S100A9 inhibitors were docked to S100A9-RAGE V dining domain to share the common region surrounded by Glu52 (at the hinge between H2 and H3), Arg85 (at H4), and Trp88 (at H4) in Figures S1–S3. 46 hit compounds also presented similar binding modes: (1) pi-pi or pi-cation interaction with residues at H4 (e.g., Trp88, Arg85) or (2) hydrogen bonding with hinge (e.g., Glu52 or Asn55) in Figures S4, S5 to add promising evidence of the hit compounds. Finally, since poor pharmacokinetic profiles and high potential toxicity are likely to fail in clinical trials, it is also crucial to predict such properties in advance to encourage further *in vivo* validation of hit molecules. We calculated the molecular parameters regarding drug-likeness and ADME properties to ensure that the hit compounds are suitable for further drug development processes (Table 5). Hopefully, all predicted values of 46 molecules are within the acceptable range. Neither Lipinski's rule of five nor Jorgensen's rule of three was violated by almost all hits. Even though we did not implement any physicochemical predictor into our model, the physicochemical property of the dataset could be transferred into screening hits through a structure-property relationship. If our model can be linked with a powerful inverse design model, we can expect our model can also provide powerful predictability with a physicochemical property range.

## Conclusion

In summary, through extensive validation of 60 models built from multi-scaffold ligand information, we optimized the machine learning classifier as well as the feature selector to obtain highly predictive classification models for identifying S100A9 inhibitors. Unlike many other reports employing only several kinds of descriptors or a whole bits of fingerprint, we combined various kinds of descriptors with a hybrid fingerprint to generate a compact and effective feature set. Ultimately, this high efficiency allowed us to further obtain 47 hits from over six million compounds through the consensus vote of models within a week, indicating the high cost-reduction ability of the models. In addition, our study is the first example of reasonable classification models for S100A9 inhibitors. Regarding the clinical importance of S100A9, as well as the difficulty of generating models for its unique characteristics, we expect that our study will further aid in developing the first S100A9 agents and guide new paths of curing diverse diseases, including Alzheimer's disease and other neurodegenerative diseases.

## Data Availability Statement

The raw data supporting the conclusions of this manuscript will be made available by the authors, without undue reservation, to any qualified researcher.

## Author Contributions

MK and S-YL conceived and designed the study at their grant based research projects. Under the designed study, SK and JL built their models, validated them and acquired *in-silico* hits through their models. MK and JL wrote the manuscript. MK and SP revised the manuscript. All the authors read and approved the final manuscript.

### Conflict of Interest

The authors declare that the research was conducted in the absence of any commercial or financial relationships that could be construed as a potential conflict of interest.

## Supplementary Material

DataSheet 1 The 2D-structure of Dataset in Table S1.
