# Supplementary figures and images for: Development of Predictive Models for Identifying Potential S100A9 Inhibitors Based on Machine Learning Methods (part 2 of 2)
Source: Front Chem. 2019 Nov 25;7:779. doi: 10.3389/fchem.2019.00779 (PMC6886474; doi:10.3389/fchem.2019.00779)

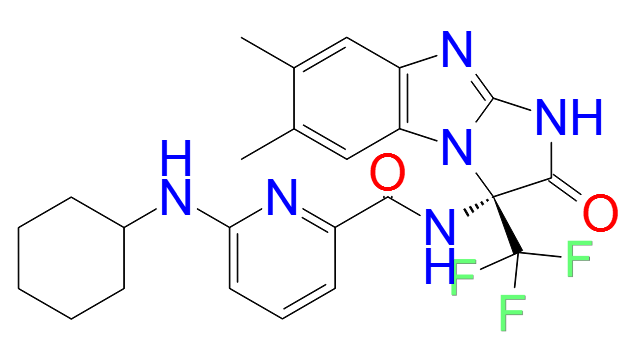

Supplement: DataSheet 1 — The 2D-structure of Dataset in Table S1. [file Data_Sheet_1.ZIP › Dataset19.png]

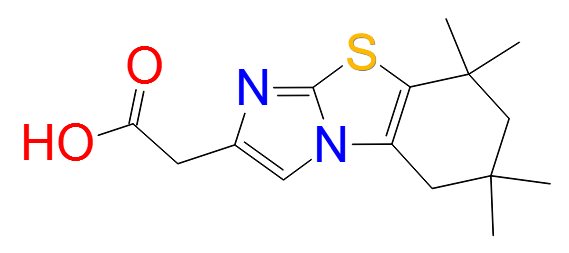

Supplement: DataSheet 1 — The 2D-structure of Dataset in Table S1. [file Data_Sheet_1.ZIP › Dataset190.png]

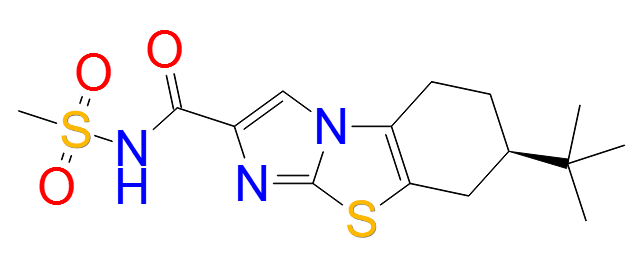

Supplement: DataSheet 1 — The 2D-structure of Dataset in Table S1. [file Data_Sheet_1.ZIP › Dataset191.png]

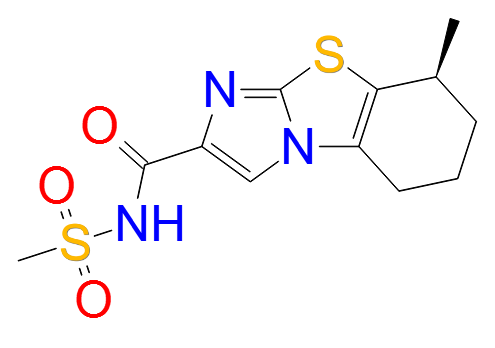

Supplement: DataSheet 1 — The 2D-structure of Dataset in Table S1. [file Data_Sheet_1.ZIP › Dataset192.png]

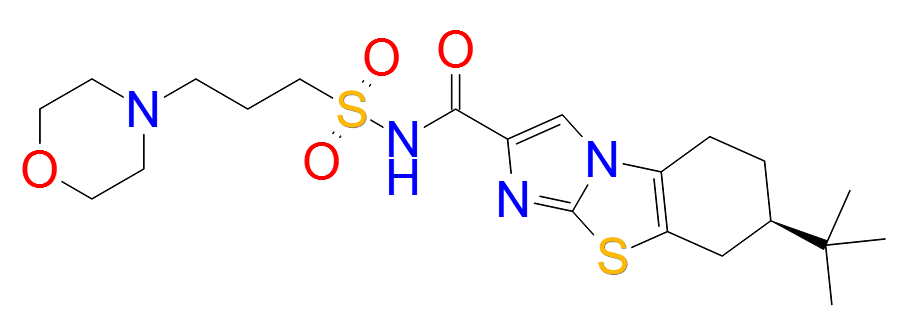

Supplement: DataSheet 1 — The 2D-structure of Dataset in Table S1. [file Data_Sheet_1.ZIP › Dataset193.png]

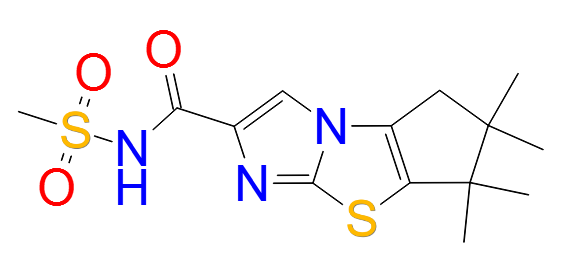

Supplement: DataSheet 1 — The 2D-structure of Dataset in Table S1. [file Data_Sheet_1.ZIP › Dataset194.png]

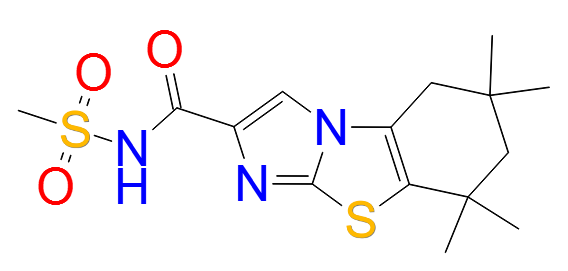

Supplement: DataSheet 1 — The 2D-structure of Dataset in Table S1. [file Data_Sheet_1.ZIP › Dataset195.png]

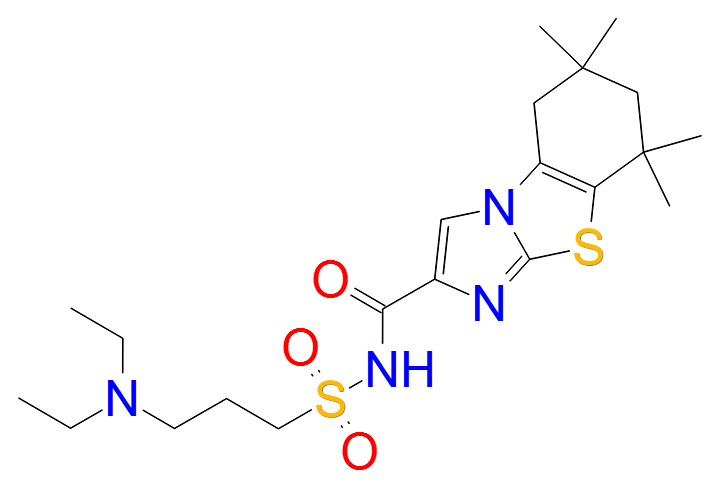

Supplement: DataSheet 1 — The 2D-structure of Dataset in Table S1. [file Data_Sheet_1.ZIP › Dataset196.png]

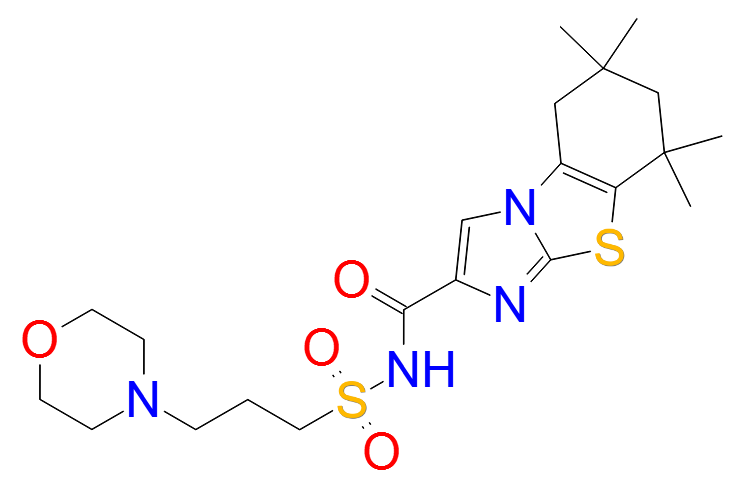

Supplement: DataSheet 1 — The 2D-structure of Dataset in Table S1. [file Data_Sheet_1.ZIP › Dataset197.png]

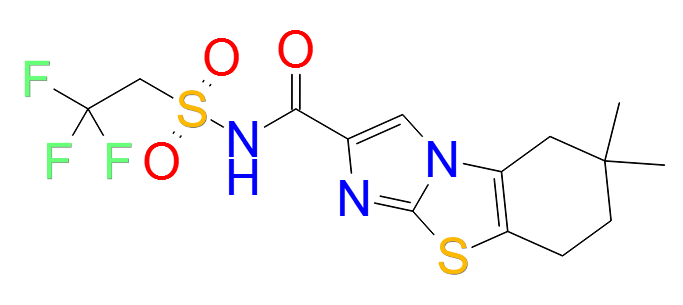

Supplement: DataSheet 1 — The 2D-structure of Dataset in Table S1. [file Data_Sheet_1.ZIP › Dataset198.png]

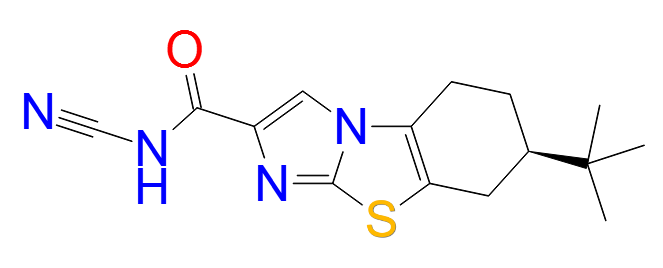

Supplement: DataSheet 1 — The 2D-structure of Dataset in Table S1. [file Data_Sheet_1.ZIP › Dataset199.png]

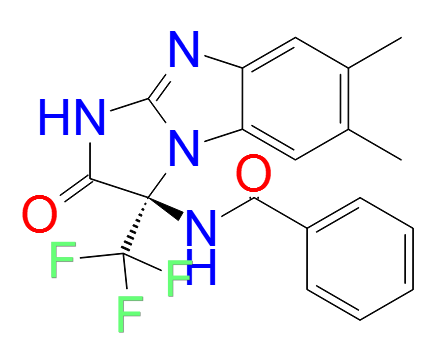

Supplement: DataSheet 1 — The 2D-structure of Dataset in Table S1. [file Data_Sheet_1.ZIP › Dataset2.png]

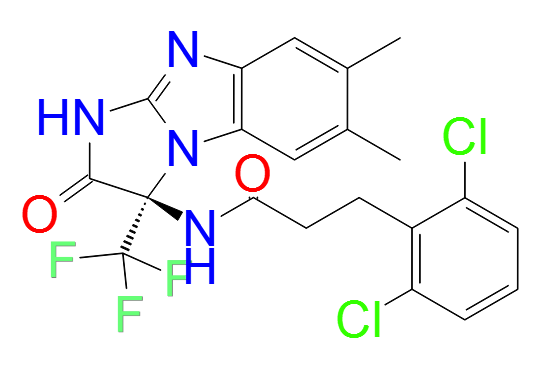

Supplement: DataSheet 1 — The 2D-structure of Dataset in Table S1. [file Data_Sheet_1.ZIP › Dataset20.png]

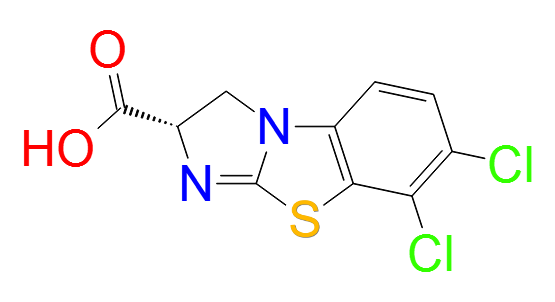

Supplement: DataSheet 1 — The 2D-structure of Dataset in Table S1. [file Data_Sheet_1.ZIP › Dataset200.png]

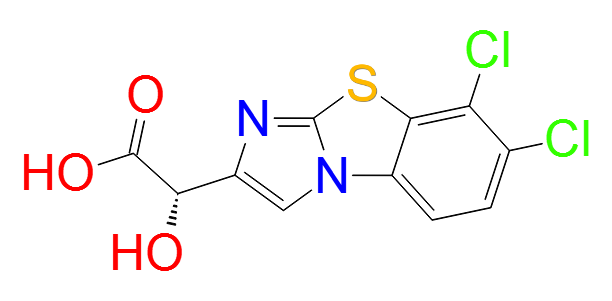

Supplement: DataSheet 1 — The 2D-structure of Dataset in Table S1. [file Data_Sheet_1.ZIP › Dataset201.png]

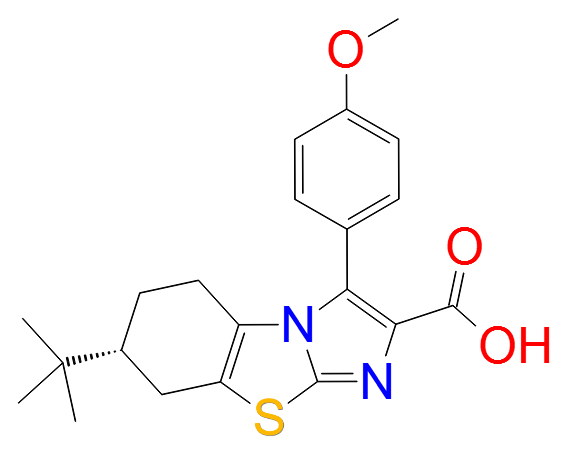

Supplement: DataSheet 1 — The 2D-structure of Dataset in Table S1. [file Data_Sheet_1.ZIP › Dataset202.png]

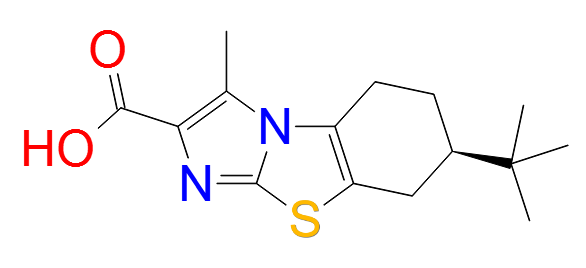

Supplement: DataSheet 1 — The 2D-structure of Dataset in Table S1. [file Data_Sheet_1.ZIP › Dataset203.png]

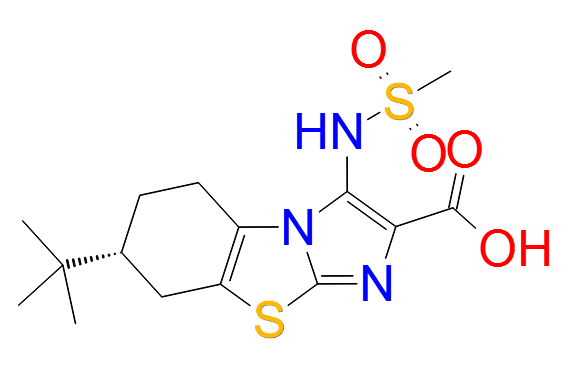

Supplement: DataSheet 1 — The 2D-structure of Dataset in Table S1. [file Data_Sheet_1.ZIP › Dataset204.png]

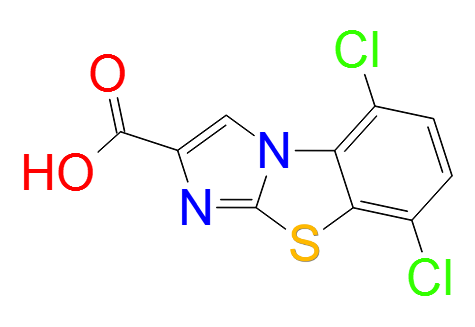

Supplement: DataSheet 1 — The 2D-structure of Dataset in Table S1. [file Data_Sheet_1.ZIP › Dataset205.png]

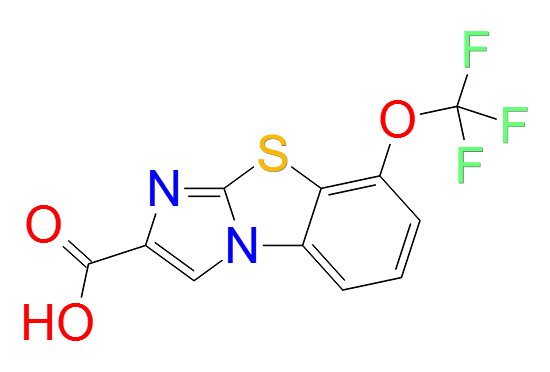

Supplement: DataSheet 1 — The 2D-structure of Dataset in Table S1. [file Data_Sheet_1.ZIP › Dataset206.png]

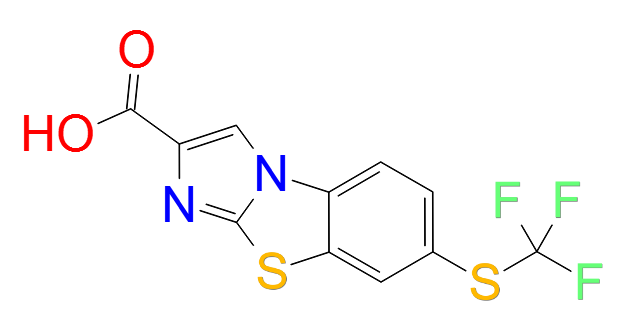

Supplement: DataSheet 1 — The 2D-structure of Dataset in Table S1. [file Data_Sheet_1.ZIP › Dataset207.png]

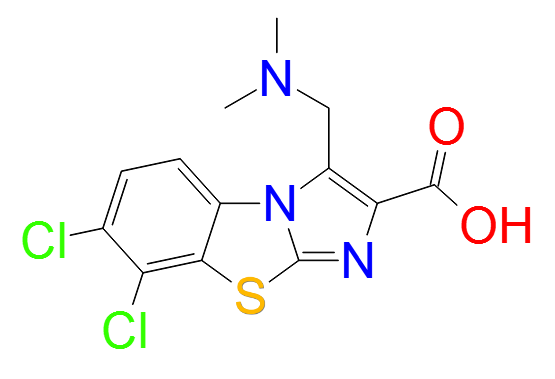

Supplement: DataSheet 1 — The 2D-structure of Dataset in Table S1. [file Data_Sheet_1.ZIP › Dataset208.png]

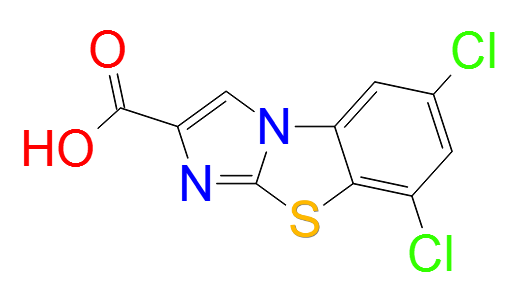

Supplement: DataSheet 1 — The 2D-structure of Dataset in Table S1. [file Data_Sheet_1.ZIP › Dataset209.png]

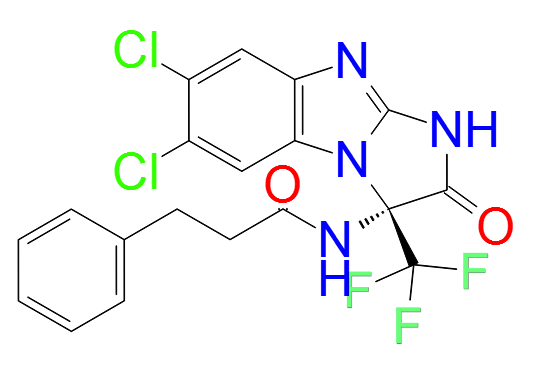

Supplement: DataSheet 1 — The 2D-structure of Dataset in Table S1. [file Data_Sheet_1.ZIP › Dataset21.png]

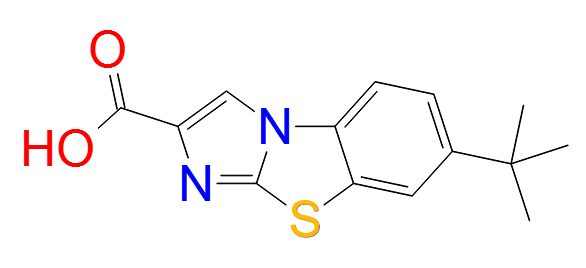

Supplement: DataSheet 1 — The 2D-structure of Dataset in Table S1. [file Data_Sheet_1.ZIP › Dataset210.png]

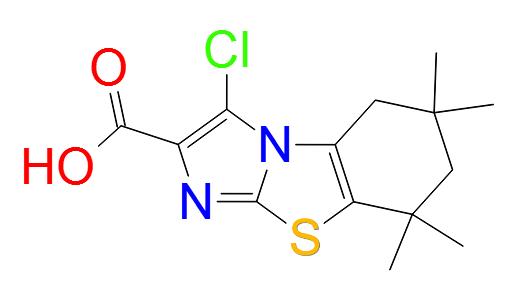

Supplement: DataSheet 1 — The 2D-structure of Dataset in Table S1. [file Data_Sheet_1.ZIP › Dataset211.png]

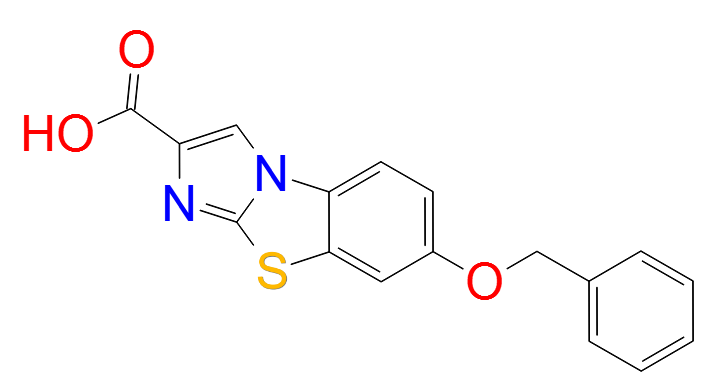

Supplement: DataSheet 1 — The 2D-structure of Dataset in Table S1. [file Data_Sheet_1.ZIP › Dataset212.png]

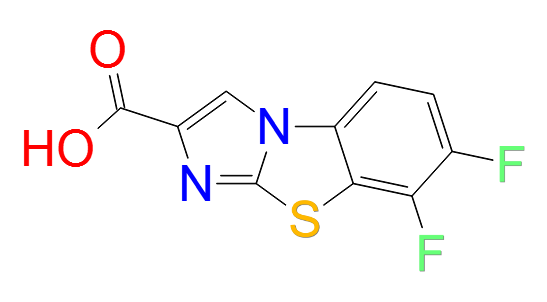

Supplement: DataSheet 1 — The 2D-structure of Dataset in Table S1. [file Data_Sheet_1.ZIP › Dataset213.png]

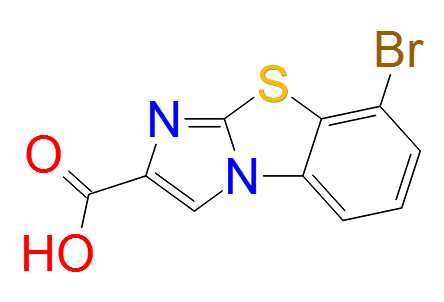

Supplement: DataSheet 1 — The 2D-structure of Dataset in Table S1. [file Data_Sheet_1.ZIP › Dataset214.png]

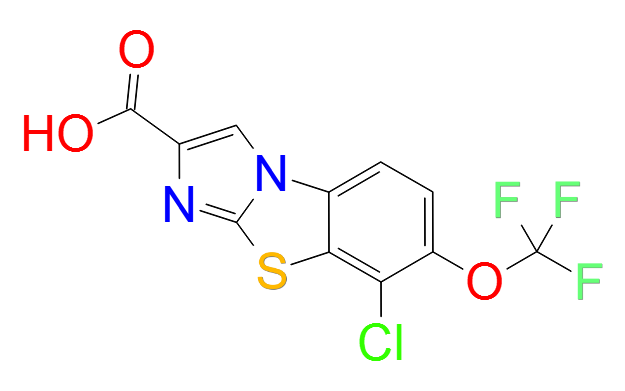

Supplement: DataSheet 1 — The 2D-structure of Dataset in Table S1. [file Data_Sheet_1.ZIP › Dataset215.png]

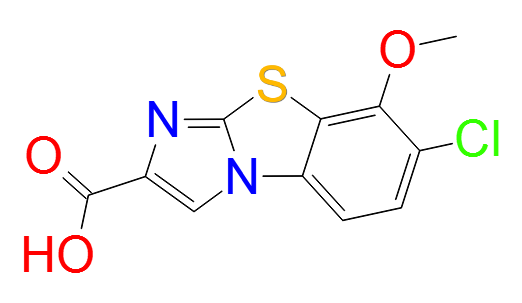

Supplement: DataSheet 1 — The 2D-structure of Dataset in Table S1. [file Data_Sheet_1.ZIP › Dataset216.png]

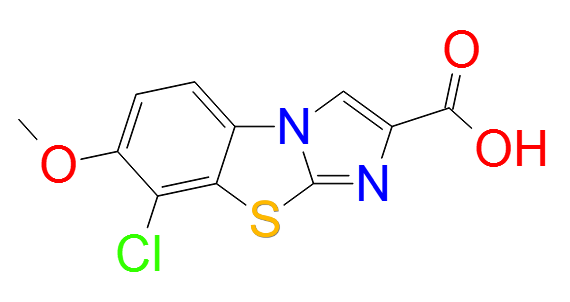

Supplement: DataSheet 1 — The 2D-structure of Dataset in Table S1. [file Data_Sheet_1.ZIP › Dataset217.png]

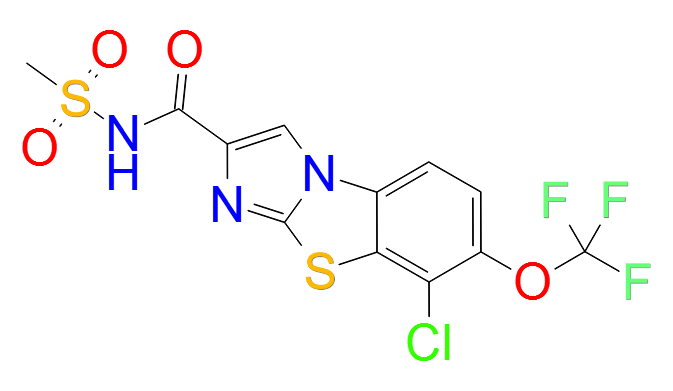

Supplement: DataSheet 1 — The 2D-structure of Dataset in Table S1. [file Data_Sheet_1.ZIP › Dataset218.png]

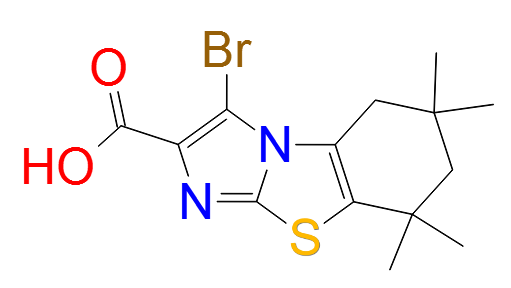

Supplement: DataSheet 1 — The 2D-structure of Dataset in Table S1. [file Data_Sheet_1.ZIP › Dataset219.png]

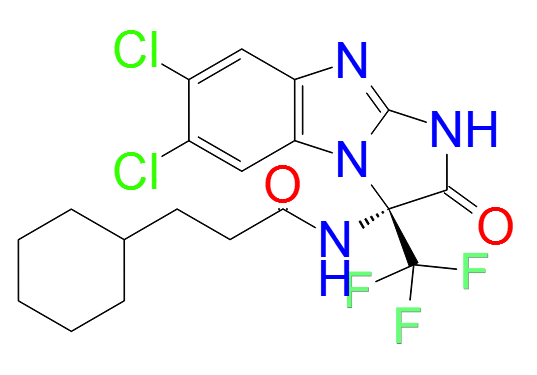

Supplement: DataSheet 1 — The 2D-structure of Dataset in Table S1. [file Data_Sheet_1.ZIP › Dataset22.png]

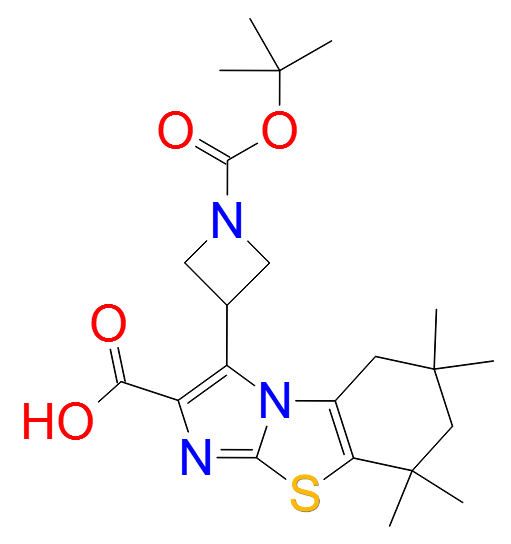

Supplement: DataSheet 1 — The 2D-structure of Dataset in Table S1. [file Data_Sheet_1.ZIP › Dataset220.png]

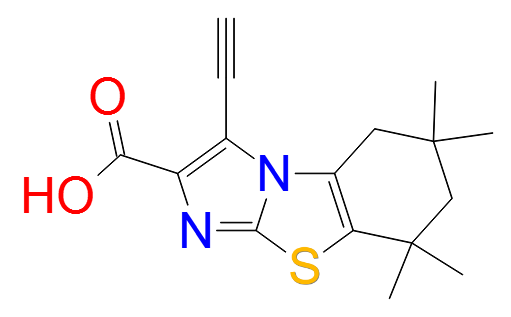

Supplement: DataSheet 1 — The 2D-structure of Dataset in Table S1. [file Data_Sheet_1.ZIP › Dataset221.png]

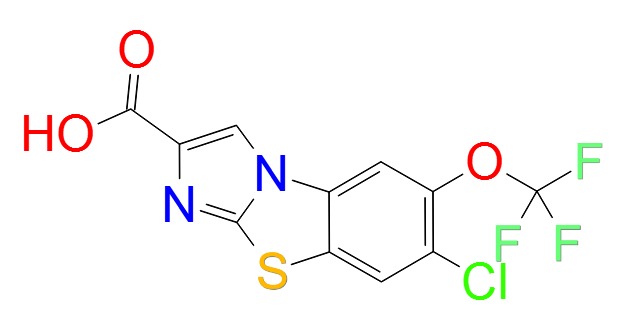

Supplement: DataSheet 1 — The 2D-structure of Dataset in Table S1. [file Data_Sheet_1.ZIP › Dataset222.png]

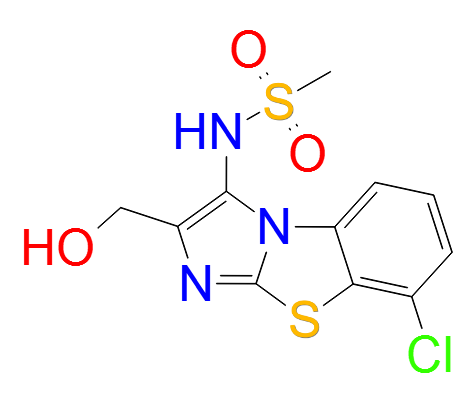

Supplement: DataSheet 1 — The 2D-structure of Dataset in Table S1. [file Data_Sheet_1.ZIP › Dataset223.png]

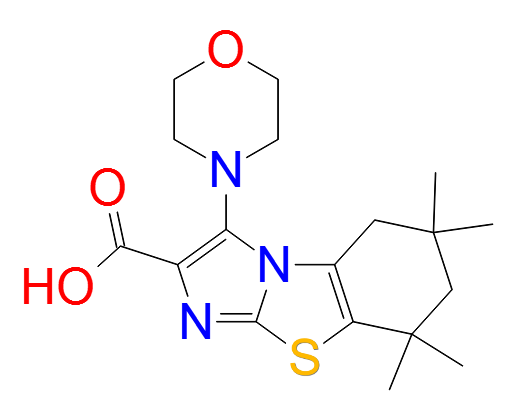

Supplement: DataSheet 1 — The 2D-structure of Dataset in Table S1. [file Data_Sheet_1.ZIP › Dataset224.png]

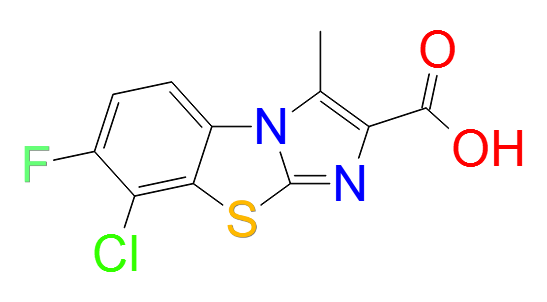

Supplement: DataSheet 1 — The 2D-structure of Dataset in Table S1. [file Data_Sheet_1.ZIP › Dataset225.png]

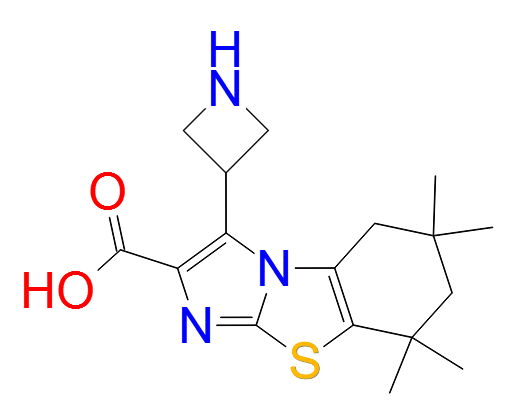

Supplement: DataSheet 1 — The 2D-structure of Dataset in Table S1. [file Data_Sheet_1.ZIP › Dataset226.png]

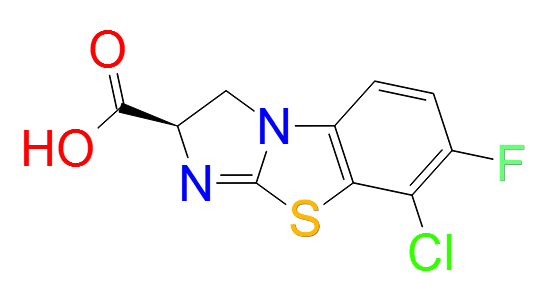

Supplement: DataSheet 1 — The 2D-structure of Dataset in Table S1. [file Data_Sheet_1.ZIP › Dataset227.png]

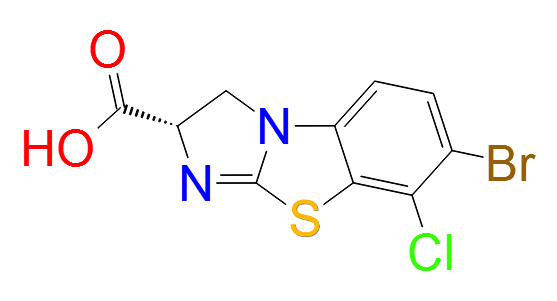

Supplement: DataSheet 1 — The 2D-structure of Dataset in Table S1. [file Data_Sheet_1.ZIP › Dataset228.png]

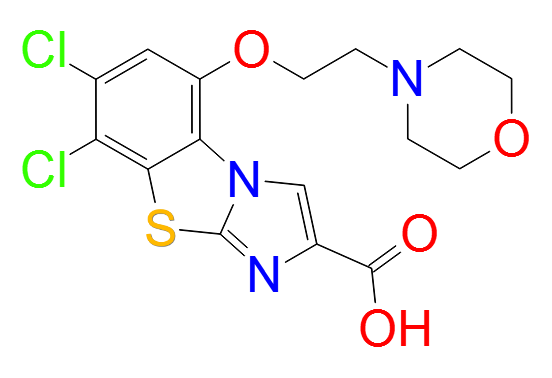

Supplement: DataSheet 1 — The 2D-structure of Dataset in Table S1. [file Data_Sheet_1.ZIP › Dataset229.png]

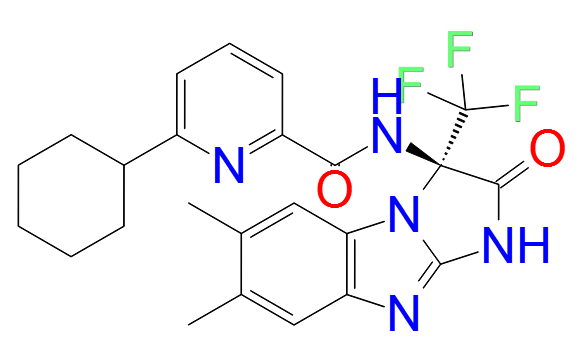

Supplement: DataSheet 1 — The 2D-structure of Dataset in Table S1. [file Data_Sheet_1.ZIP › Dataset23.png]

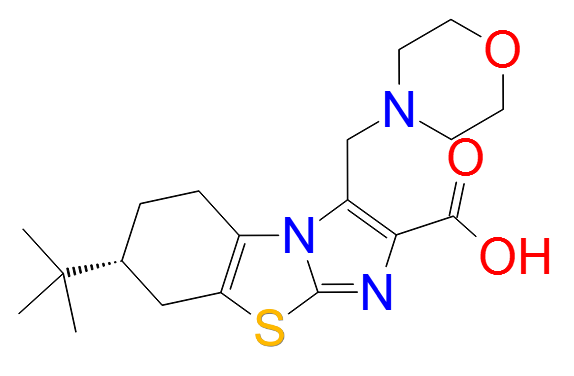

Supplement: DataSheet 1 — The 2D-structure of Dataset in Table S1. [file Data_Sheet_1.ZIP › Dataset230.png]

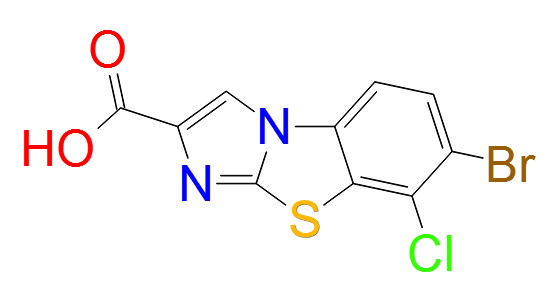

Supplement: DataSheet 1 — The 2D-structure of Dataset in Table S1. [file Data_Sheet_1.ZIP › Dataset231.png]

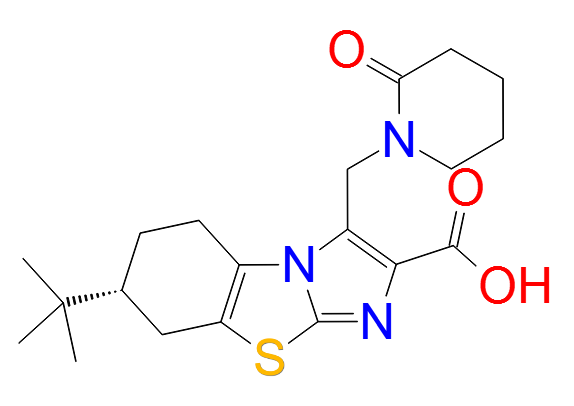

Supplement: DataSheet 1 — The 2D-structure of Dataset in Table S1. [file Data_Sheet_1.ZIP › Dataset232.png]

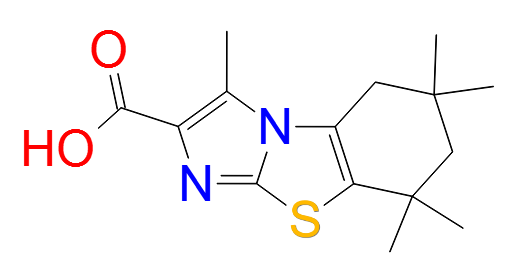

Supplement: DataSheet 1 — The 2D-structure of Dataset in Table S1. [file Data_Sheet_1.ZIP › Dataset233.png]

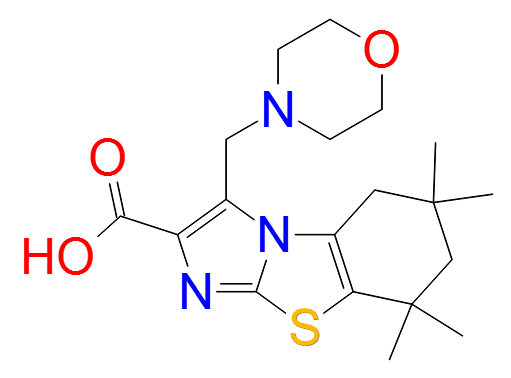

Supplement: DataSheet 1 — The 2D-structure of Dataset in Table S1. [file Data_Sheet_1.ZIP › Dataset234.png]

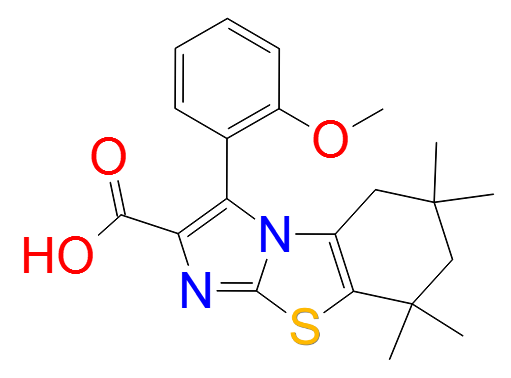

Supplement: DataSheet 1 — The 2D-structure of Dataset in Table S1. [file Data_Sheet_1.ZIP › Dataset235.png]

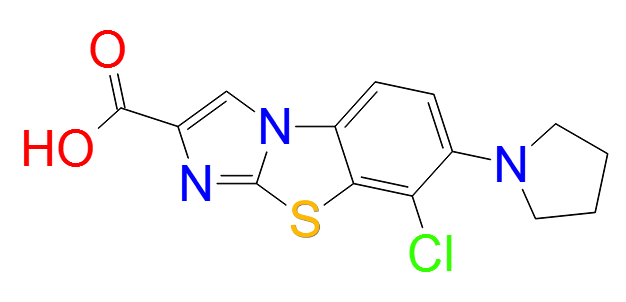

Supplement: DataSheet 1 — The 2D-structure of Dataset in Table S1. [file Data_Sheet_1.ZIP › Dataset236.png]

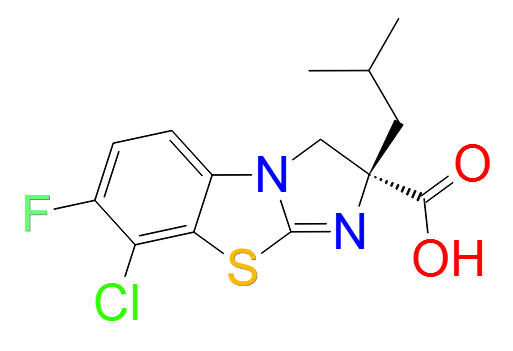

Supplement: DataSheet 1 — The 2D-structure of Dataset in Table S1. [file Data_Sheet_1.ZIP › Dataset237.png]

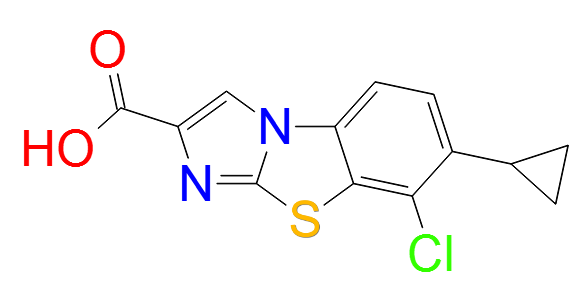

Supplement: DataSheet 1 — The 2D-structure of Dataset in Table S1. [file Data_Sheet_1.ZIP › Dataset238.png]

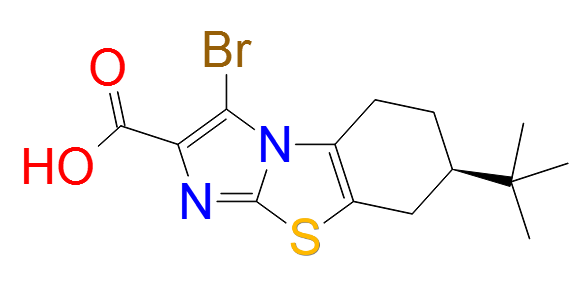

Supplement: DataSheet 1 — The 2D-structure of Dataset in Table S1. [file Data_Sheet_1.ZIP › Dataset239.png]

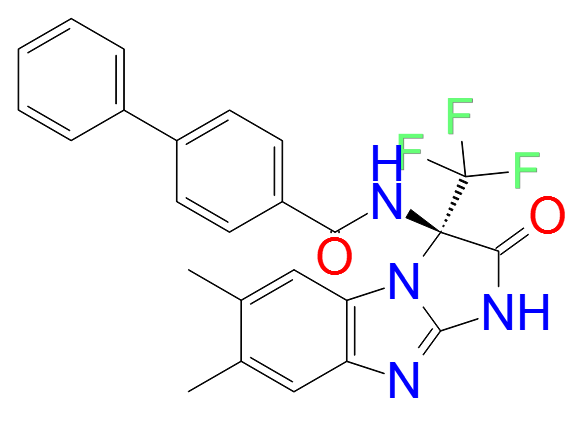

Supplement: DataSheet 1 — The 2D-structure of Dataset in Table S1. [file Data_Sheet_1.ZIP › Dataset24.png]

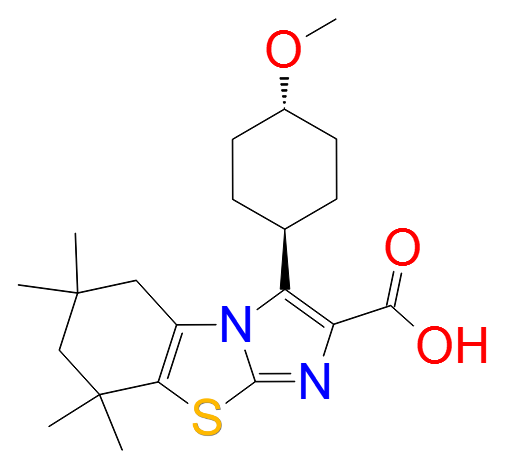

Supplement: DataSheet 1 — The 2D-structure of Dataset in Table S1. [file Data_Sheet_1.ZIP › Dataset240.png]

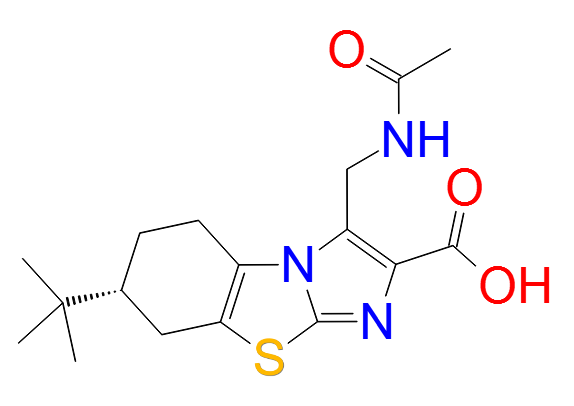

Supplement: DataSheet 1 — The 2D-structure of Dataset in Table S1. [file Data_Sheet_1.ZIP › Dataset241.png]

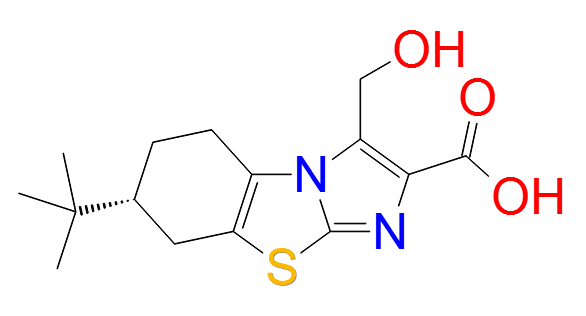

Supplement: DataSheet 1 — The 2D-structure of Dataset in Table S1. [file Data_Sheet_1.ZIP › Dataset242.png]

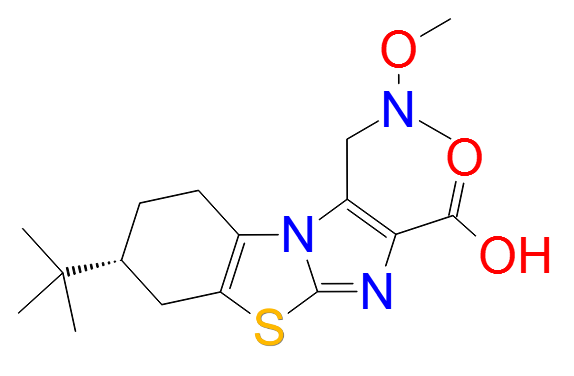

Supplement: DataSheet 1 — The 2D-structure of Dataset in Table S1. [file Data_Sheet_1.ZIP › Dataset243.png]

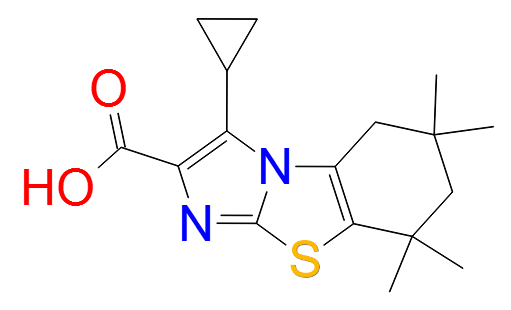

Supplement: DataSheet 1 — The 2D-structure of Dataset in Table S1. [file Data_Sheet_1.ZIP › Dataset244.png]

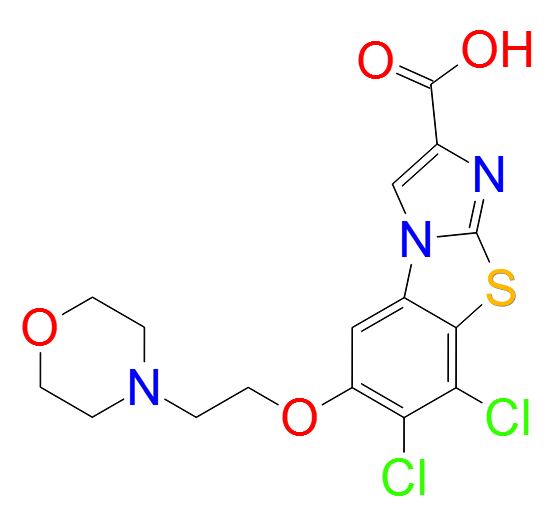

Supplement: DataSheet 1 — The 2D-structure of Dataset in Table S1. [file Data_Sheet_1.ZIP › Dataset245.png]

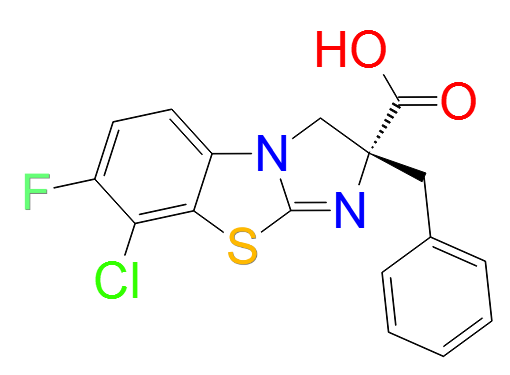

Supplement: DataSheet 1 — The 2D-structure of Dataset in Table S1. [file Data_Sheet_1.ZIP › Dataset246.png]

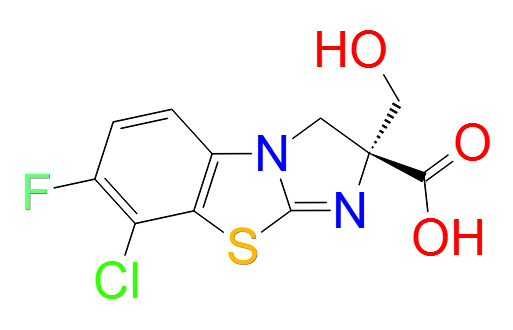

Supplement: DataSheet 1 — The 2D-structure of Dataset in Table S1. [file Data_Sheet_1.ZIP › Dataset247.png]

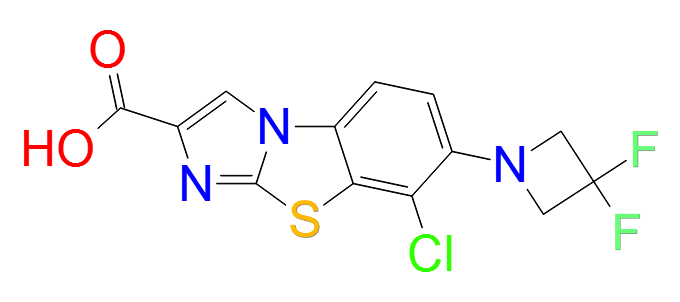

Supplement: DataSheet 1 — The 2D-structure of Dataset in Table S1. [file Data_Sheet_1.ZIP › Dataset248.png]

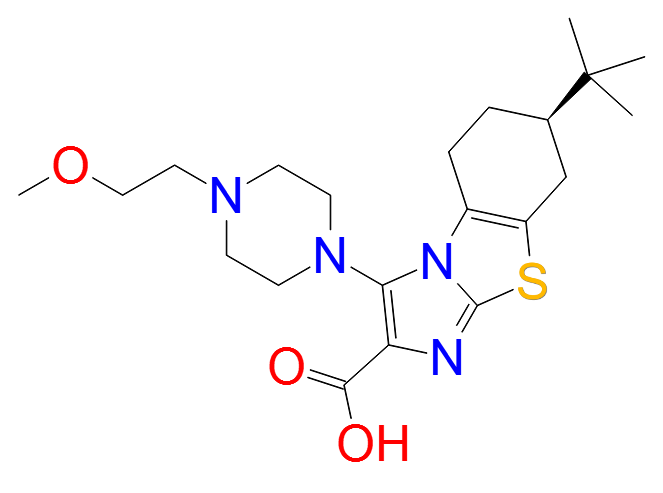

Supplement: DataSheet 1 — The 2D-structure of Dataset in Table S1. [file Data_Sheet_1.ZIP › Dataset249.png]

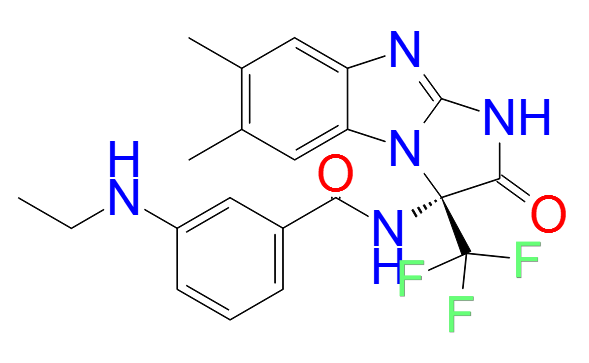

Supplement: DataSheet 1 — The 2D-structure of Dataset in Table S1. [file Data_Sheet_1.ZIP › Dataset25.png]

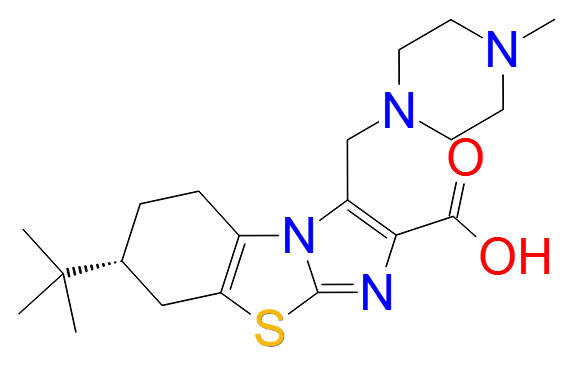

Supplement: DataSheet 1 — The 2D-structure of Dataset in Table S1. [file Data_Sheet_1.ZIP › Dataset250.png]

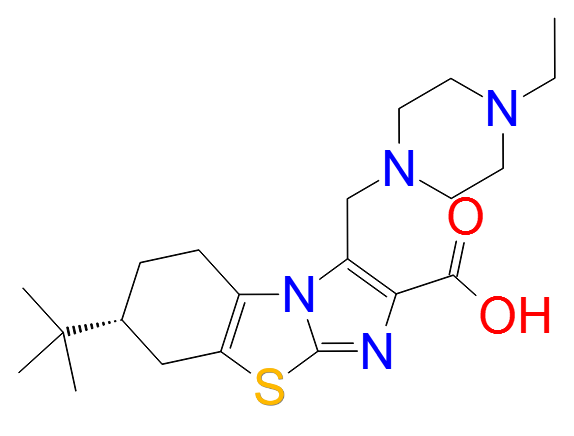

Supplement: DataSheet 1 — The 2D-structure of Dataset in Table S1. [file Data_Sheet_1.ZIP › Dataset251.png]

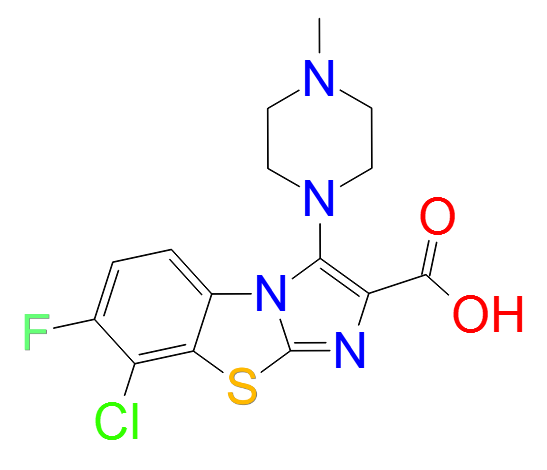

Supplement: DataSheet 1 — The 2D-structure of Dataset in Table S1. [file Data_Sheet_1.ZIP › Dataset252.png]

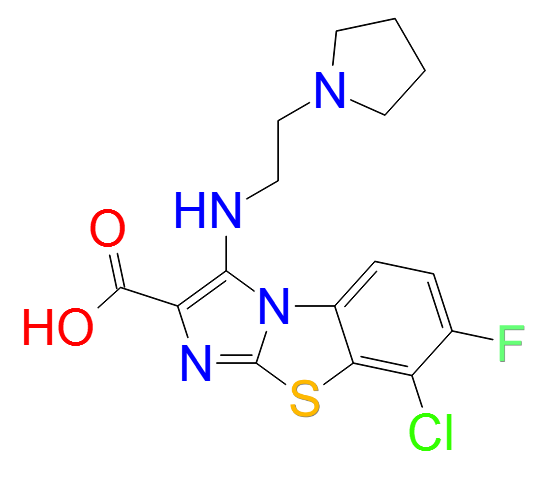

Supplement: DataSheet 1 — The 2D-structure of Dataset in Table S1. [file Data_Sheet_1.ZIP › Dataset253.png]

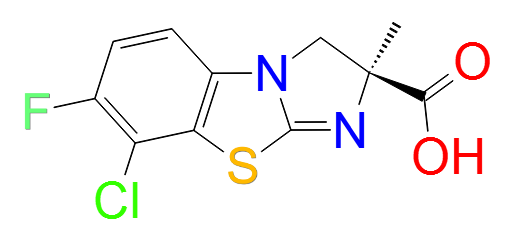

Supplement: DataSheet 1 — The 2D-structure of Dataset in Table S1. [file Data_Sheet_1.ZIP › Dataset254.png]

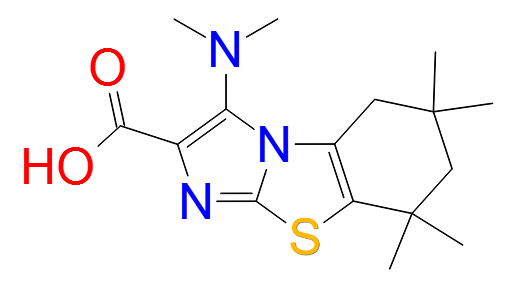

Supplement: DataSheet 1 — The 2D-structure of Dataset in Table S1. [file Data_Sheet_1.ZIP › Dataset255.png]

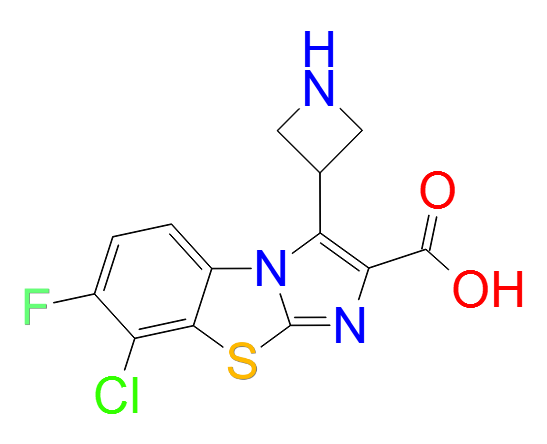

Supplement: DataSheet 1 — The 2D-structure of Dataset in Table S1. [file Data_Sheet_1.ZIP › Dataset256.png]

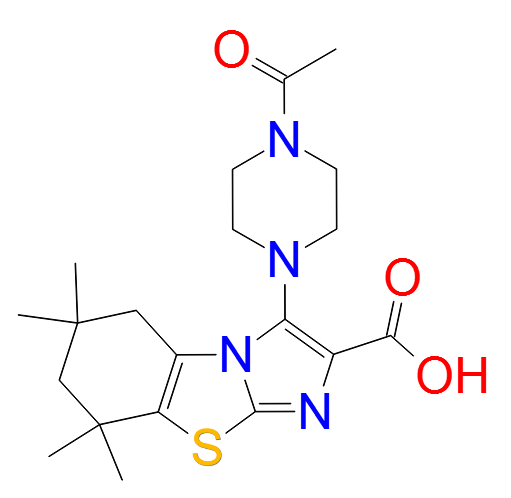

Supplement: DataSheet 1 — The 2D-structure of Dataset in Table S1. [file Data_Sheet_1.ZIP › Dataset257.png]

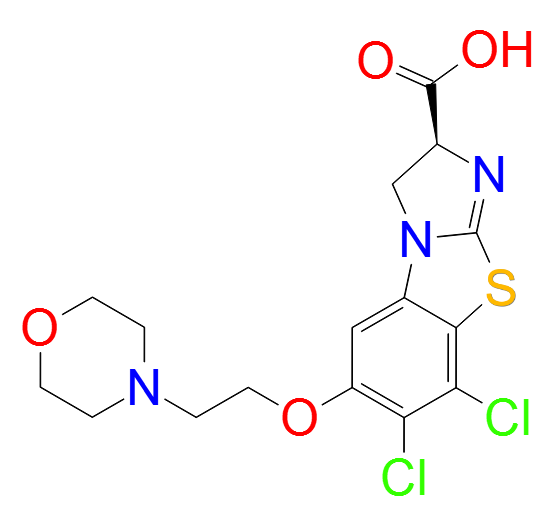

Supplement: DataSheet 1 — The 2D-structure of Dataset in Table S1. [file Data_Sheet_1.ZIP › Dataset258.png]

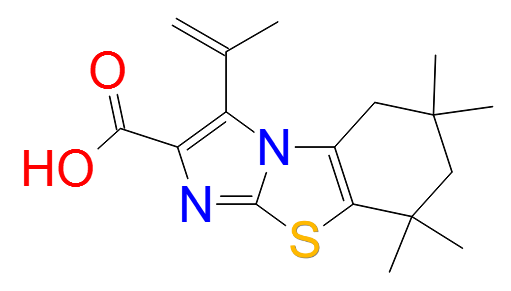

Supplement: DataSheet 1 — The 2D-structure of Dataset in Table S1. [file Data_Sheet_1.ZIP › Dataset259.png]

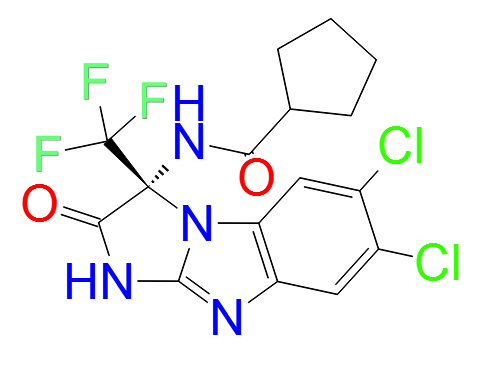

Supplement: DataSheet 1 — The 2D-structure of Dataset in Table S1. [file Data_Sheet_1.ZIP › Dataset26.png]

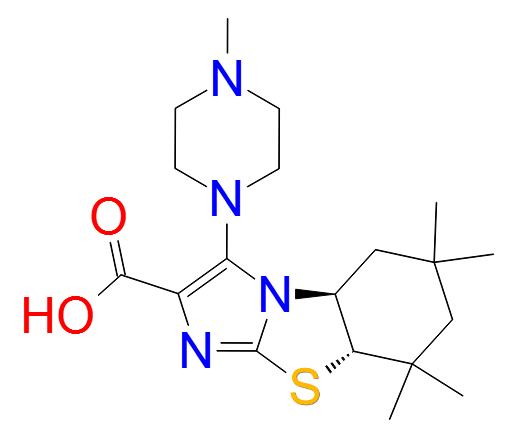

Supplement: DataSheet 1 — The 2D-structure of Dataset in Table S1. [file Data_Sheet_1.ZIP › Dataset260.png]

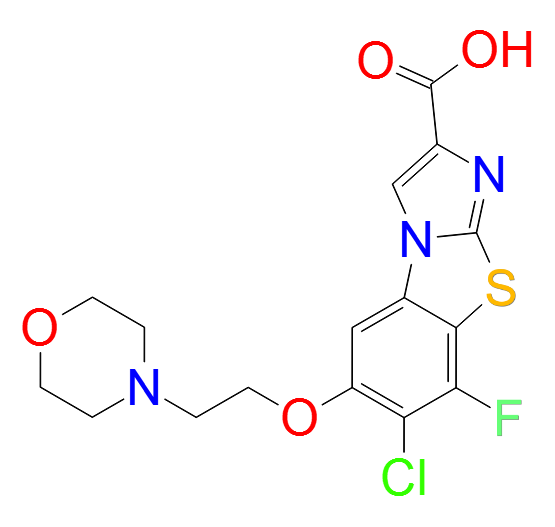

Supplement: DataSheet 1 — The 2D-structure of Dataset in Table S1. [file Data_Sheet_1.ZIP › Dataset261.png]

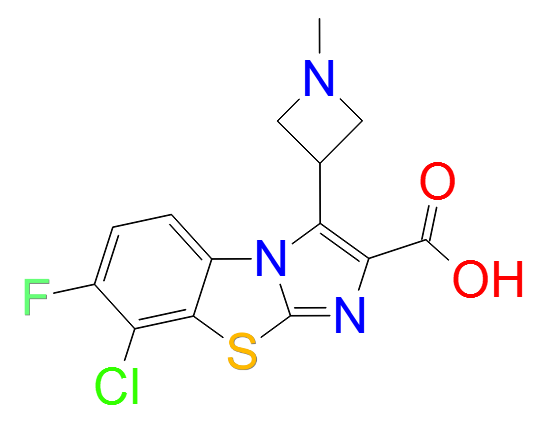

Supplement: DataSheet 1 — The 2D-structure of Dataset in Table S1. [file Data_Sheet_1.ZIP › Dataset262.png]

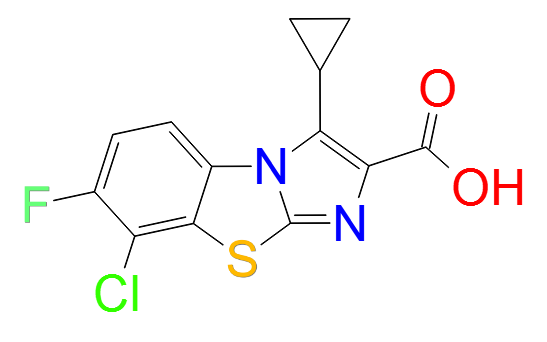

Supplement: DataSheet 1 — The 2D-structure of Dataset in Table S1. [file Data_Sheet_1.ZIP › Dataset263.png]

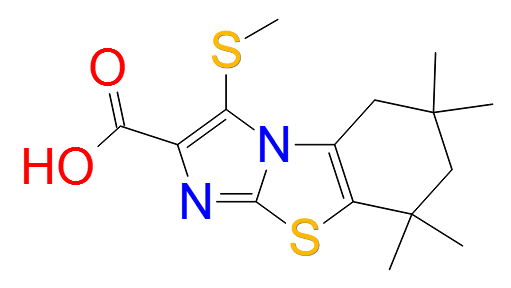

Supplement: DataSheet 1 — The 2D-structure of Dataset in Table S1. [file Data_Sheet_1.ZIP › Dataset264.png]

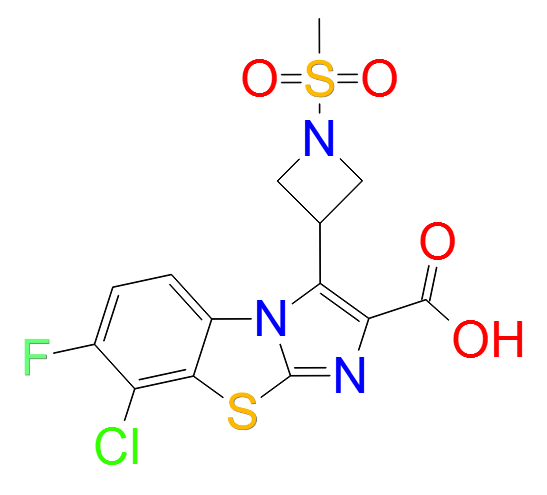

Supplement: DataSheet 1 — The 2D-structure of Dataset in Table S1. [file Data_Sheet_1.ZIP › Dataset265.png]

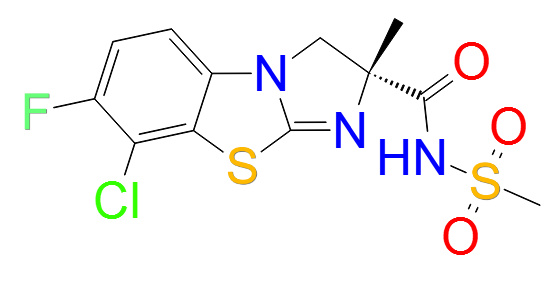

Supplement: DataSheet 1 — The 2D-structure of Dataset in Table S1. [file Data_Sheet_1.ZIP › Dataset266.png]

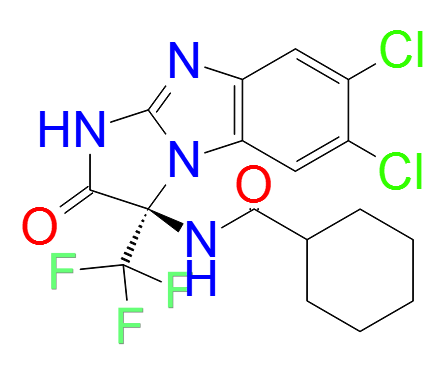

Supplement: DataSheet 1 — The 2D-structure of Dataset in Table S1. [file Data_Sheet_1.ZIP › Dataset27.png]

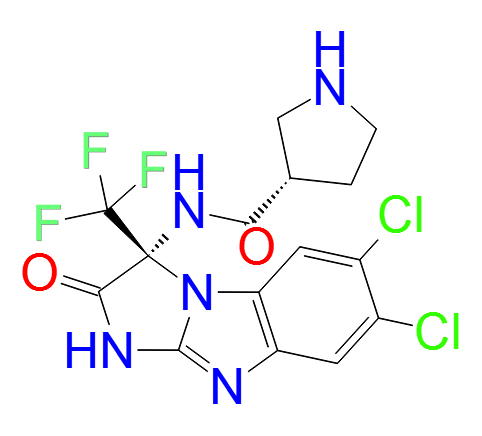

Supplement: DataSheet 1 — The 2D-structure of Dataset in Table S1. [file Data_Sheet_1.ZIP › Dataset28.png]

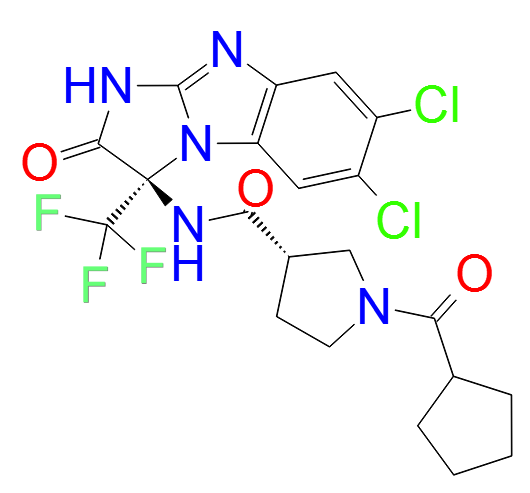

Supplement: DataSheet 1 — The 2D-structure of Dataset in Table S1. [file Data_Sheet_1.ZIP › Dataset29.png]

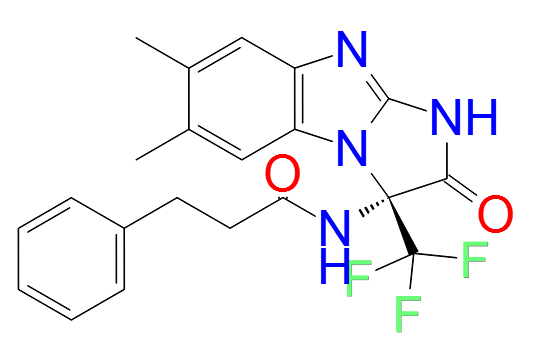

Supplement: DataSheet 1 — The 2D-structure of Dataset in Table S1. [file Data_Sheet_1.ZIP › Dataset3.png]

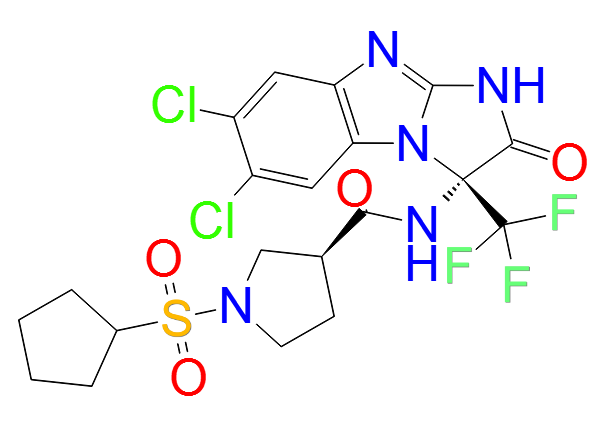

Supplement: DataSheet 1 — The 2D-structure of Dataset in Table S1. [file Data_Sheet_1.ZIP › Dataset30.png]

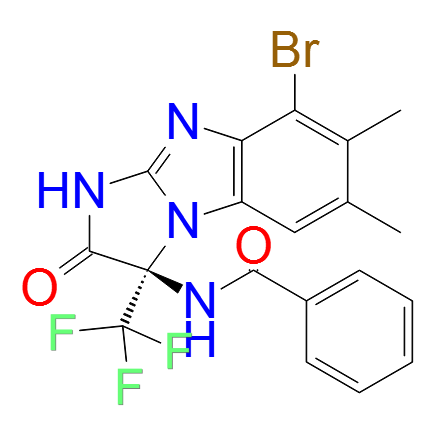

Supplement: DataSheet 1 — The 2D-structure of Dataset in Table S1. [file Data_Sheet_1.ZIP › Dataset31.png]

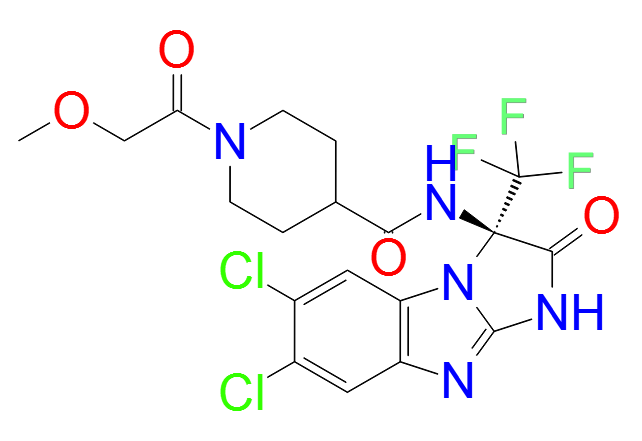

Supplement: DataSheet 1 — The 2D-structure of Dataset in Table S1. [file Data_Sheet_1.ZIP › Dataset32.png]

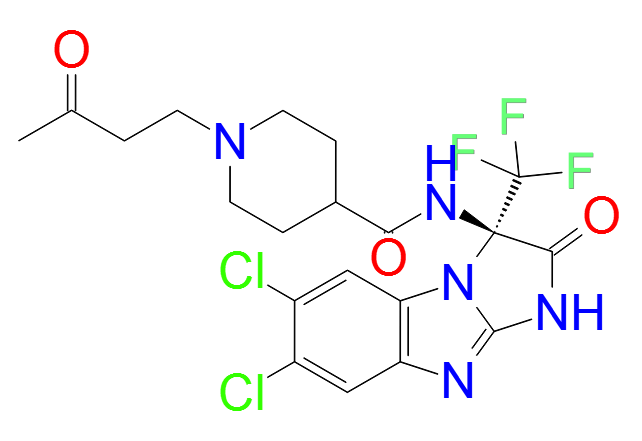

Supplement: DataSheet 1 — The 2D-structure of Dataset in Table S1. [file Data_Sheet_1.ZIP › Dataset33.png]

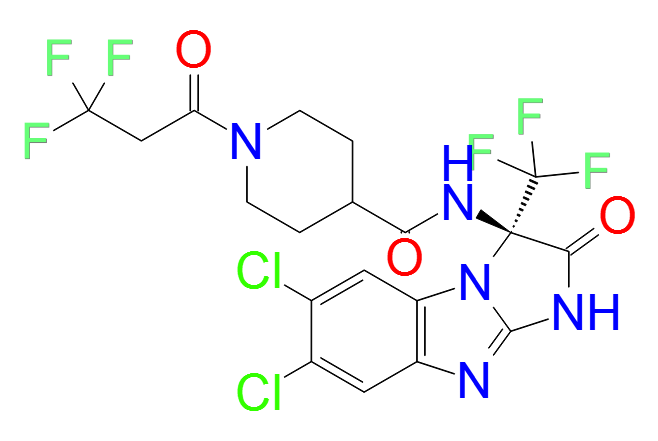

Supplement: DataSheet 1 — The 2D-structure of Dataset in Table S1. [file Data_Sheet_1.ZIP › Dataset34.png]

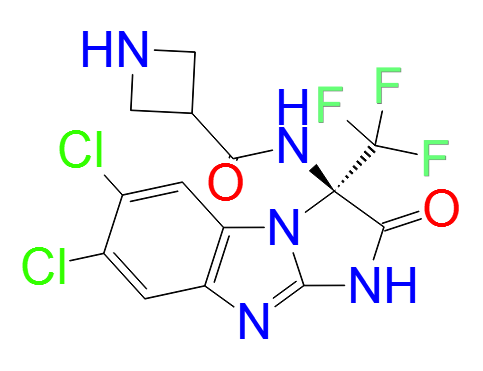

Supplement: DataSheet 1 — The 2D-structure of Dataset in Table S1. [file Data_Sheet_1.ZIP › Dataset35.png]

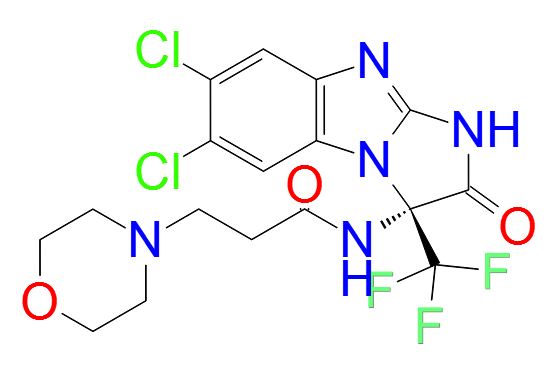

Supplement: DataSheet 1 — The 2D-structure of Dataset in Table S1. [file Data_Sheet_1.ZIP › Dataset36.png]

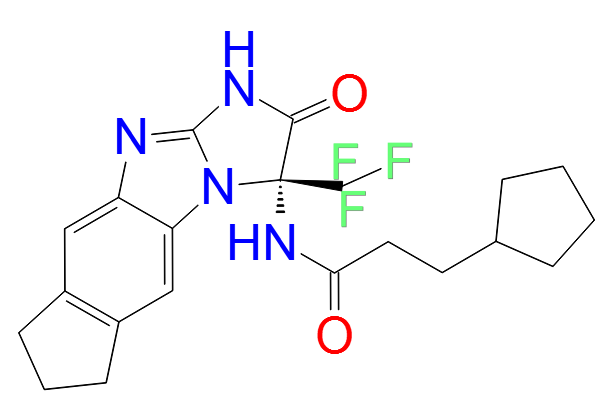

Supplement: DataSheet 1 — The 2D-structure of Dataset in Table S1. [file Data_Sheet_1.ZIP › Dataset37.png]

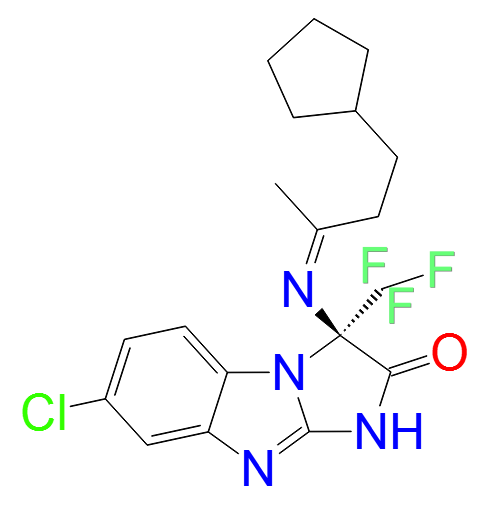

Supplement: DataSheet 1 — The 2D-structure of Dataset in Table S1. [file Data_Sheet_1.ZIP › Dataset38.png]

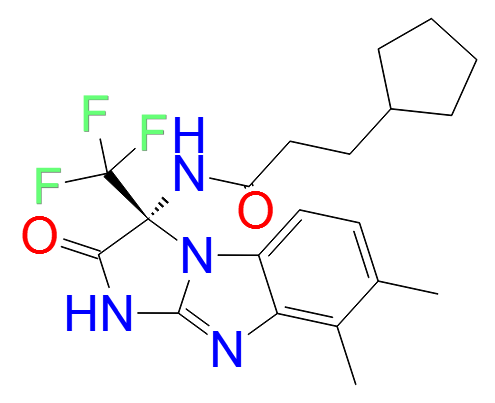

Supplement: DataSheet 1 — The 2D-structure of Dataset in Table S1. [file Data_Sheet_1.ZIP › Dataset39.png]
