# Supplementary figures and images for: Development of Predictive Models for Identifying Potential S100A9 Inhibitors Based on Machine Learning Methods (part 1 of 2)
Source: Front Chem. 2019 Nov 25;7:779. doi: 10.3389/fchem.2019.00779 (PMC6886474; doi:10.3389/fchem.2019.00779)

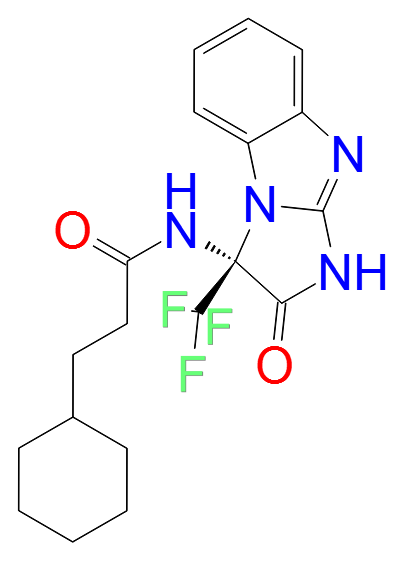

Supplement: DataSheet 1 — The 2D-structure of Dataset in Table S1. [file Data_Sheet_1.ZIP › Dataset1.png]

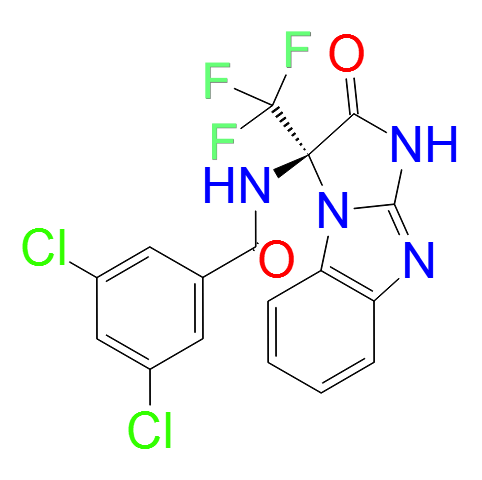

Supplement: DataSheet 1 — The 2D-structure of Dataset in Table S1. [file Data_Sheet_1.ZIP › Dataset10.png]

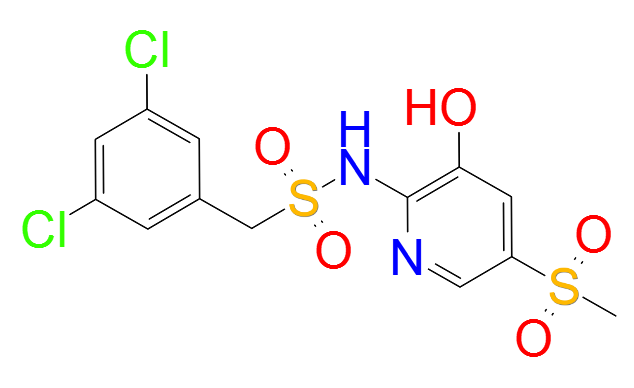

Supplement: DataSheet 1 — The 2D-structure of Dataset in Table S1. [file Data_Sheet_1.ZIP › Dataset100.png]

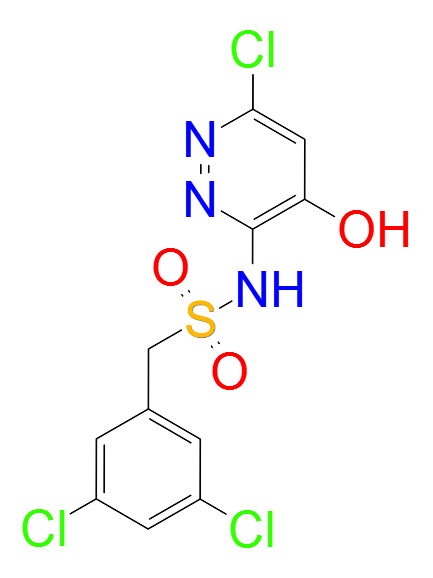

Supplement: DataSheet 1 — The 2D-structure of Dataset in Table S1. [file Data_Sheet_1.ZIP › Dataset101.png]

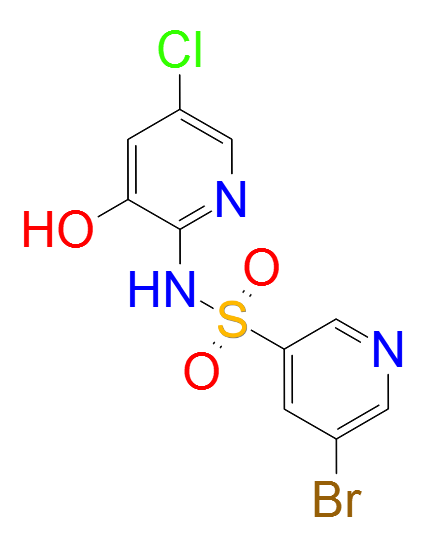

Supplement: DataSheet 1 — The 2D-structure of Dataset in Table S1. [file Data_Sheet_1.ZIP › Dataset102.png]

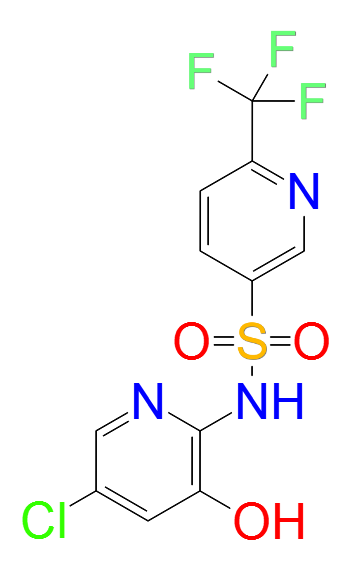

Supplement: DataSheet 1 — The 2D-structure of Dataset in Table S1. [file Data_Sheet_1.ZIP › Dataset103.png]

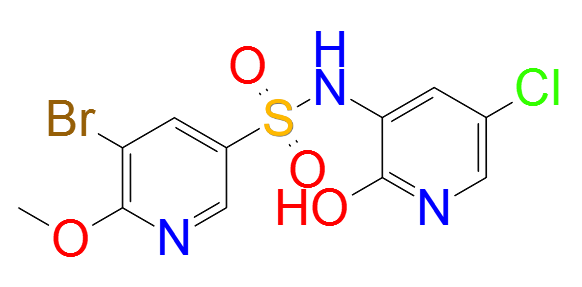

Supplement: DataSheet 1 — The 2D-structure of Dataset in Table S1. [file Data_Sheet_1.ZIP › Dataset104.png]

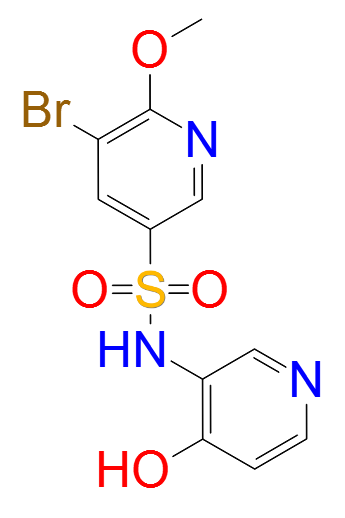

Supplement: DataSheet 1 — The 2D-structure of Dataset in Table S1. [file Data_Sheet_1.ZIP › Dataset105.png]

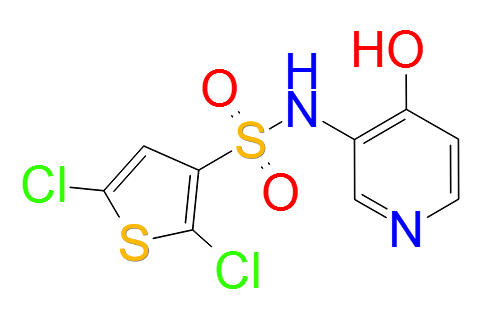

Supplement: DataSheet 1 — The 2D-structure of Dataset in Table S1. [file Data_Sheet_1.ZIP › Dataset106.png]

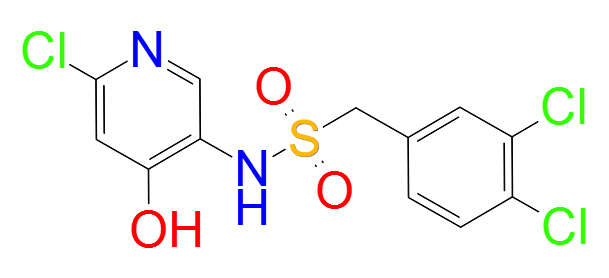

Supplement: DataSheet 1 — The 2D-structure of Dataset in Table S1. [file Data_Sheet_1.ZIP › Dataset107.png]

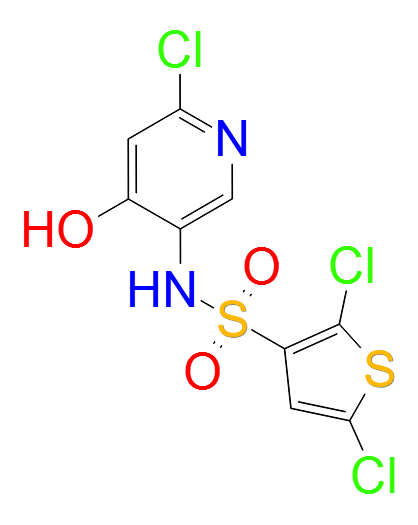

Supplement: DataSheet 1 — The 2D-structure of Dataset in Table S1. [file Data_Sheet_1.ZIP › Dataset108.png]

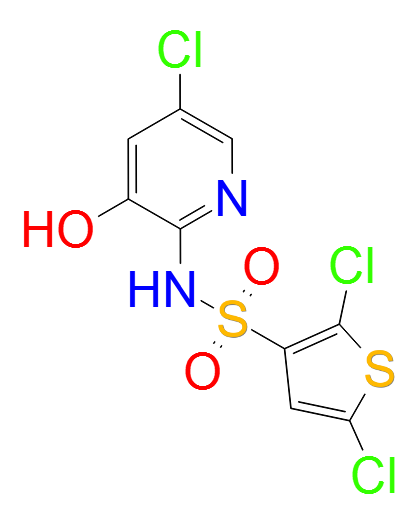

Supplement: DataSheet 1 — The 2D-structure of Dataset in Table S1. [file Data_Sheet_1.ZIP › Dataset109.png]

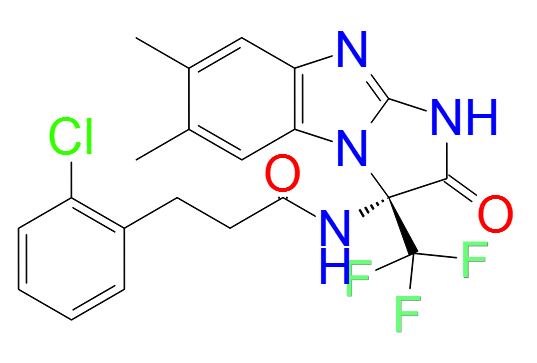

Supplement: DataSheet 1 — The 2D-structure of Dataset in Table S1. [file Data_Sheet_1.ZIP › Dataset11.png]

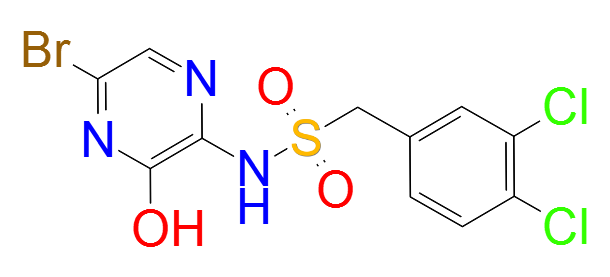

Supplement: DataSheet 1 — The 2D-structure of Dataset in Table S1. [file Data_Sheet_1.ZIP › Dataset110.png]

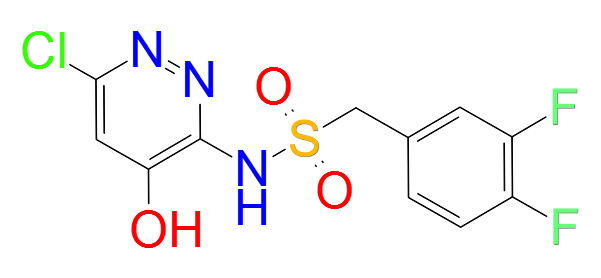

Supplement: DataSheet 1 — The 2D-structure of Dataset in Table S1. [file Data_Sheet_1.ZIP › Dataset111.png]

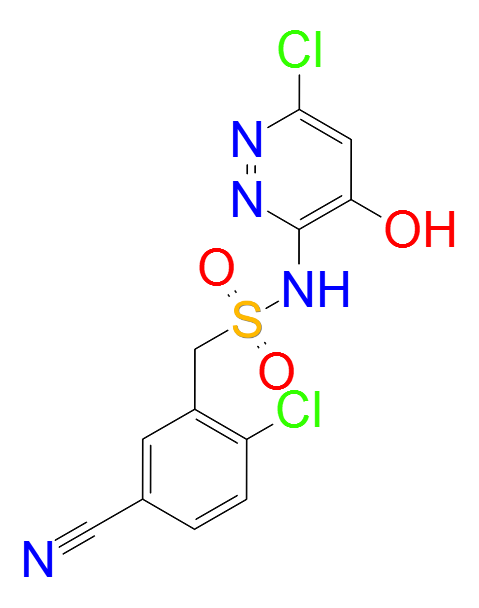

Supplement: DataSheet 1 — The 2D-structure of Dataset in Table S1. [file Data_Sheet_1.ZIP › Dataset112.png]

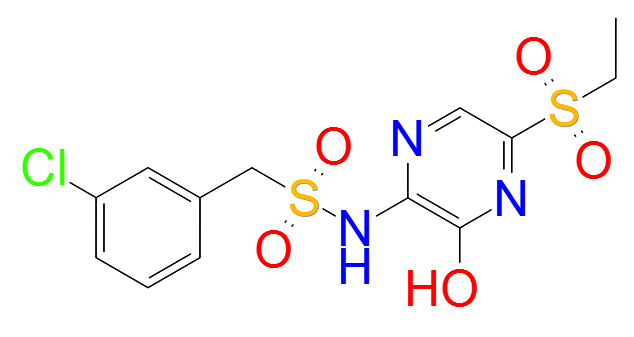

Supplement: DataSheet 1 — The 2D-structure of Dataset in Table S1. [file Data_Sheet_1.ZIP › Dataset113.png]

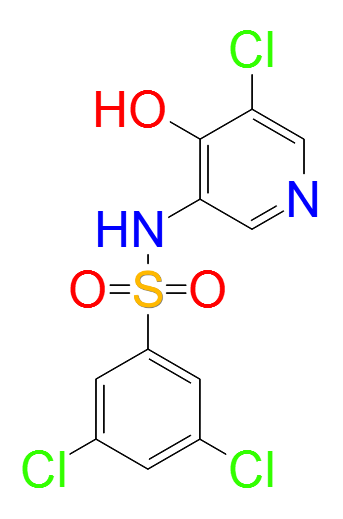

Supplement: DataSheet 1 — The 2D-structure of Dataset in Table S1. [file Data_Sheet_1.ZIP › Dataset114.png]

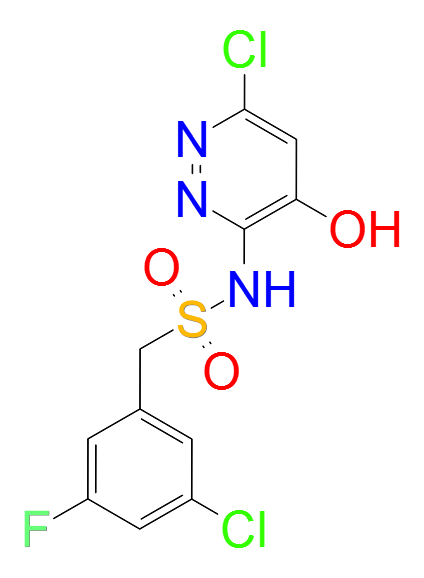

Supplement: DataSheet 1 — The 2D-structure of Dataset in Table S1. [file Data_Sheet_1.ZIP › Dataset115.png]

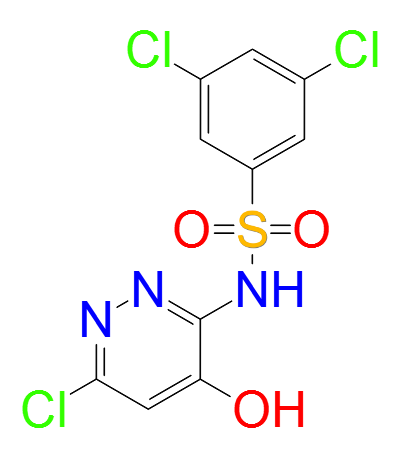

Supplement: DataSheet 1 — The 2D-structure of Dataset in Table S1. [file Data_Sheet_1.ZIP › Dataset116.png]

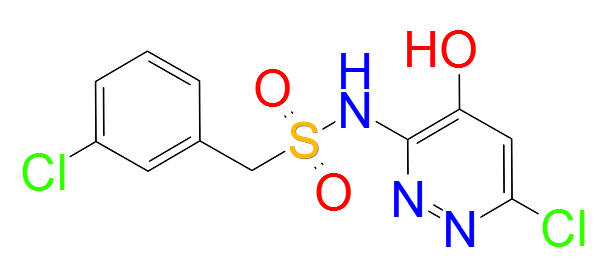

Supplement: DataSheet 1 — The 2D-structure of Dataset in Table S1. [file Data_Sheet_1.ZIP › Dataset117.png]

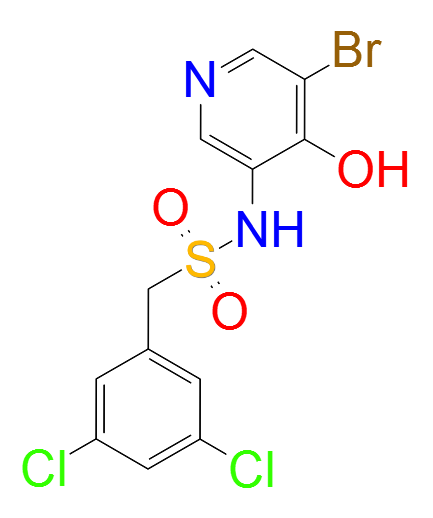

Supplement: DataSheet 1 — The 2D-structure of Dataset in Table S1. [file Data_Sheet_1.ZIP › Dataset118.png]

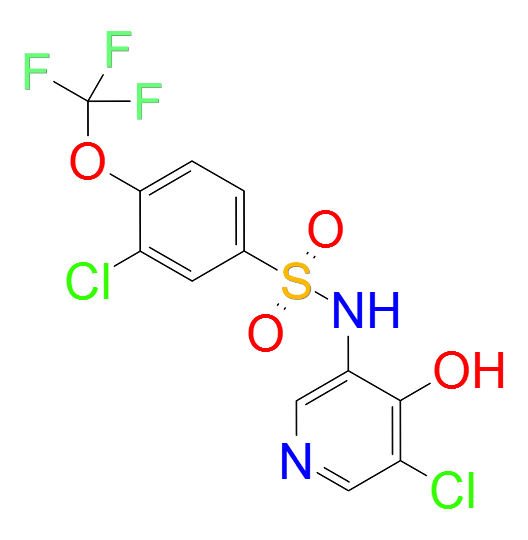

Supplement: DataSheet 1 — The 2D-structure of Dataset in Table S1. [file Data_Sheet_1.ZIP › Dataset119.png]

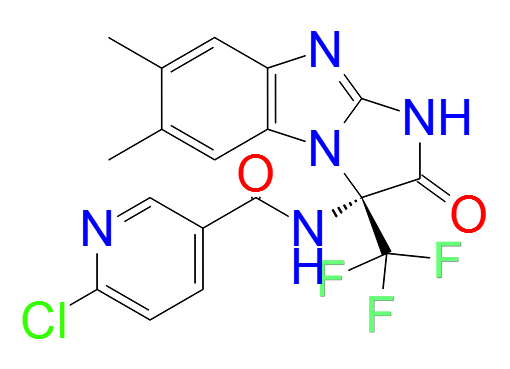

Supplement: DataSheet 1 — The 2D-structure of Dataset in Table S1. [file Data_Sheet_1.ZIP › Dataset12.png]

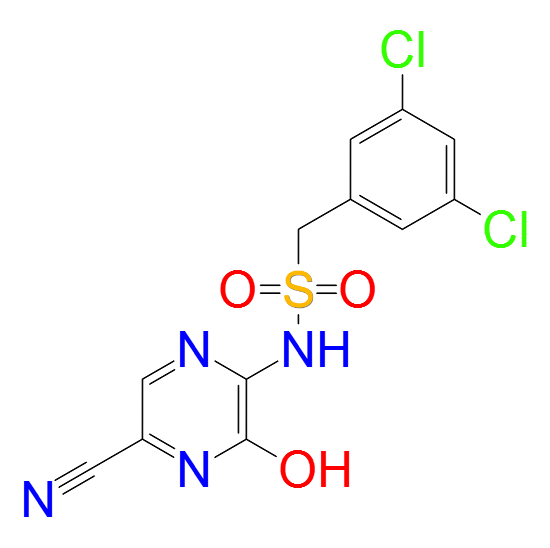

Supplement: DataSheet 1 — The 2D-structure of Dataset in Table S1. [file Data_Sheet_1.ZIP › Dataset120.png]

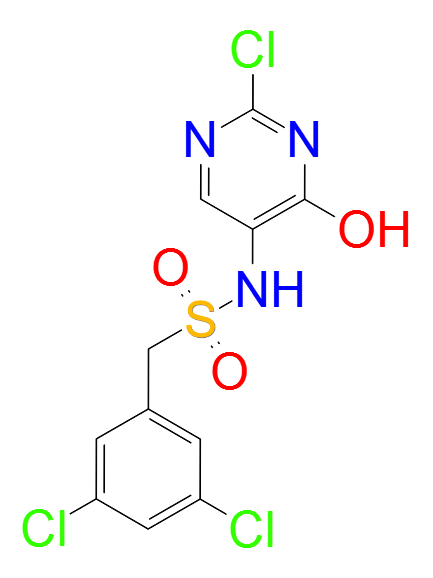

Supplement: DataSheet 1 — The 2D-structure of Dataset in Table S1. [file Data_Sheet_1.ZIP › Dataset121.png]

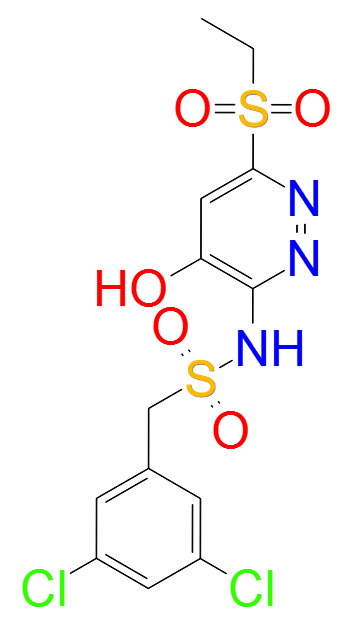

Supplement: DataSheet 1 — The 2D-structure of Dataset in Table S1. [file Data_Sheet_1.ZIP › Dataset122.png]

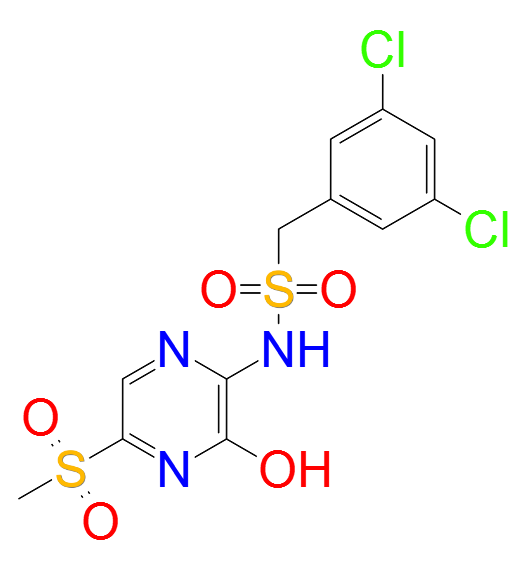

Supplement: DataSheet 1 — The 2D-structure of Dataset in Table S1. [file Data_Sheet_1.ZIP › Dataset123.png]

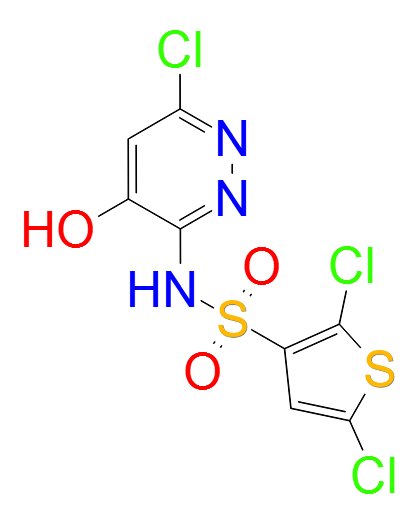

Supplement: DataSheet 1 — The 2D-structure of Dataset in Table S1. [file Data_Sheet_1.ZIP › Dataset124.png]

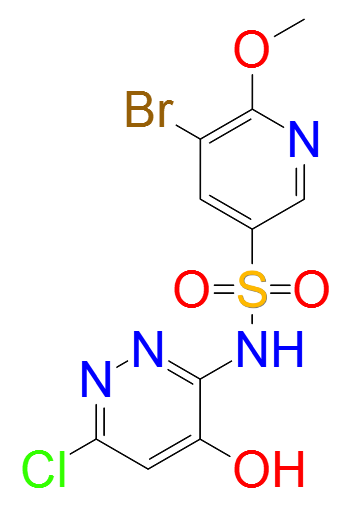

Supplement: DataSheet 1 — The 2D-structure of Dataset in Table S1. [file Data_Sheet_1.ZIP › Dataset125.png]

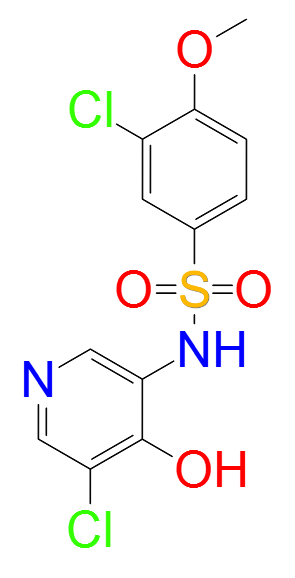

Supplement: DataSheet 1 — The 2D-structure of Dataset in Table S1. [file Data_Sheet_1.ZIP › Dataset126.png]

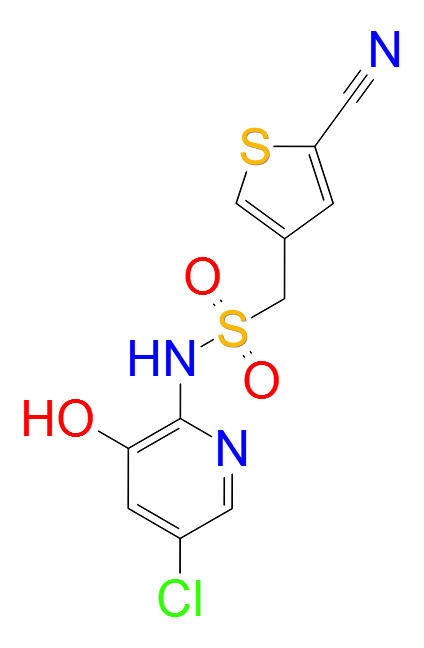

Supplement: DataSheet 1 — The 2D-structure of Dataset in Table S1. [file Data_Sheet_1.ZIP › Dataset127.png]

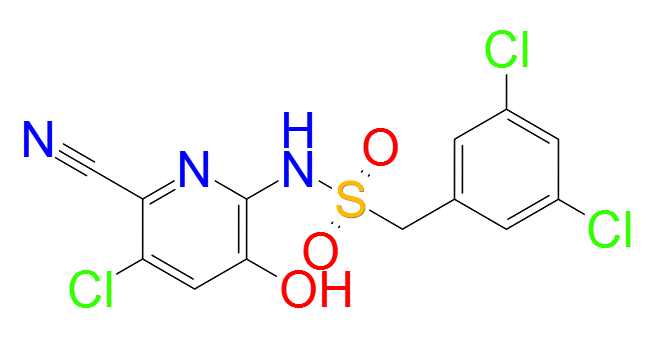

Supplement: DataSheet 1 — The 2D-structure of Dataset in Table S1. [file Data_Sheet_1.ZIP › Dataset128.png]

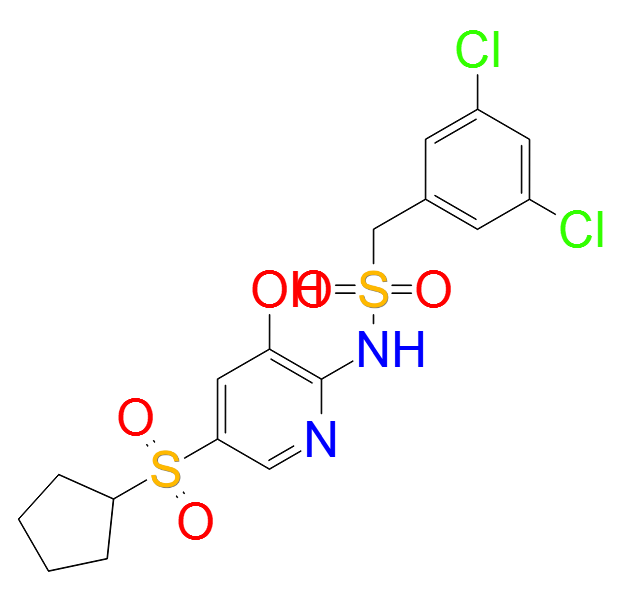

Supplement: DataSheet 1 — The 2D-structure of Dataset in Table S1. [file Data_Sheet_1.ZIP › Dataset129.png]

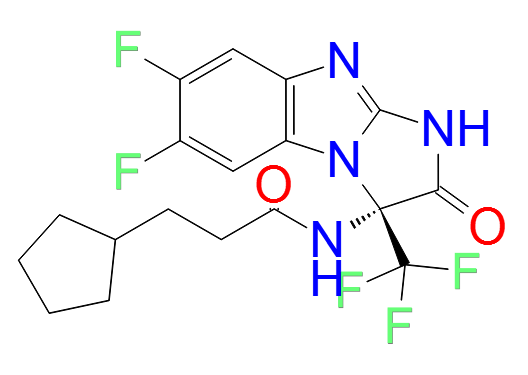

Supplement: DataSheet 1 — The 2D-structure of Dataset in Table S1. [file Data_Sheet_1.ZIP › Dataset13.png]

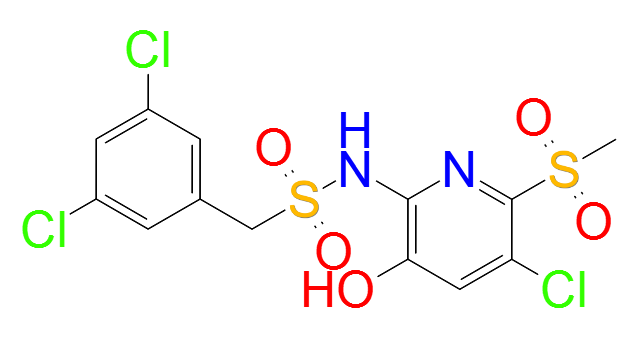

Supplement: DataSheet 1 — The 2D-structure of Dataset in Table S1. [file Data_Sheet_1.ZIP › Dataset130.png]

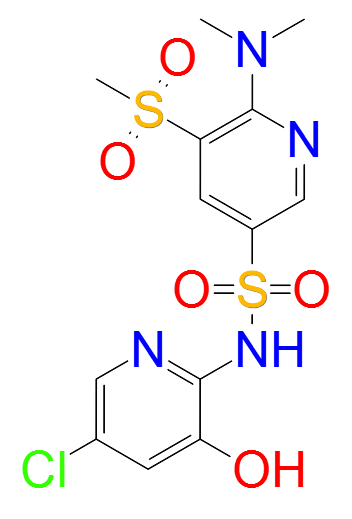

Supplement: DataSheet 1 — The 2D-structure of Dataset in Table S1. [file Data_Sheet_1.ZIP › Dataset131.png]

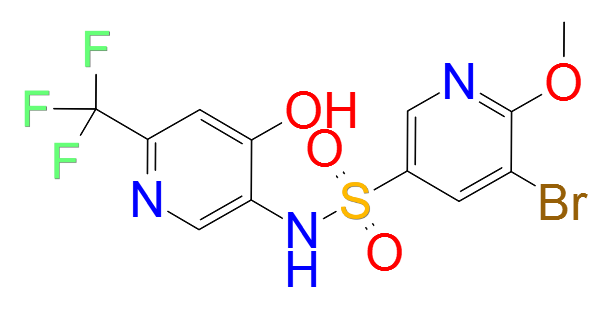

Supplement: DataSheet 1 — The 2D-structure of Dataset in Table S1. [file Data_Sheet_1.ZIP › Dataset132.png]

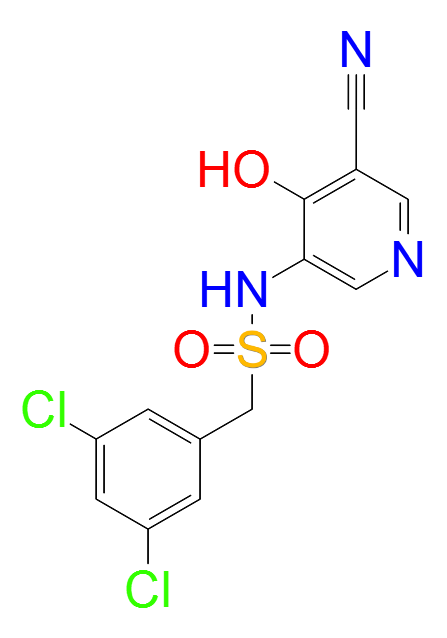

Supplement: DataSheet 1 — The 2D-structure of Dataset in Table S1. [file Data_Sheet_1.ZIP › Dataset133.png]

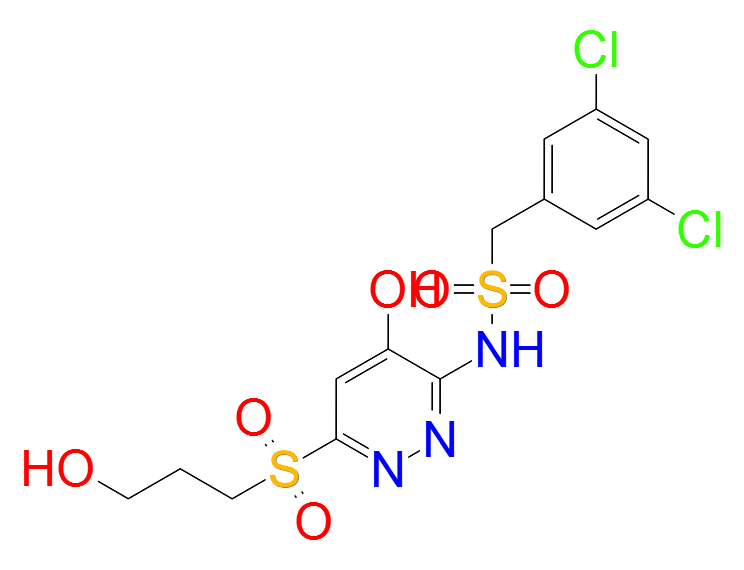

Supplement: DataSheet 1 — The 2D-structure of Dataset in Table S1. [file Data_Sheet_1.ZIP › Dataset134.png]

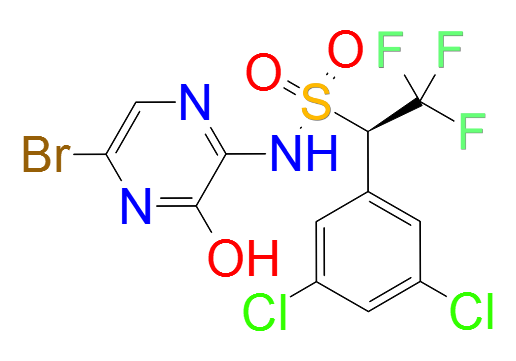

Supplement: DataSheet 1 — The 2D-structure of Dataset in Table S1. [file Data_Sheet_1.ZIP › Dataset135.png]

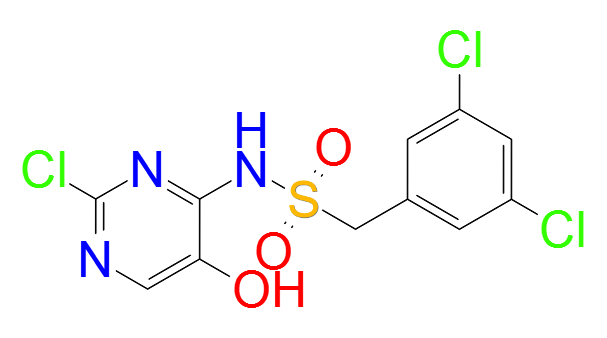

Supplement: DataSheet 1 — The 2D-structure of Dataset in Table S1. [file Data_Sheet_1.ZIP › Dataset136.png]

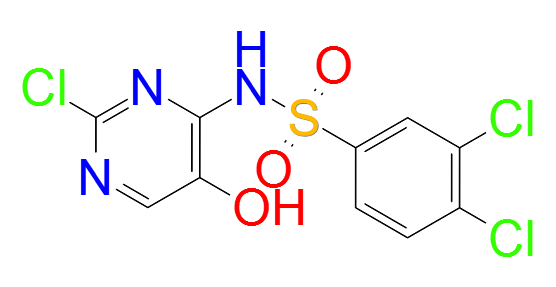

Supplement: DataSheet 1 — The 2D-structure of Dataset in Table S1. [file Data_Sheet_1.ZIP › Dataset137.png]

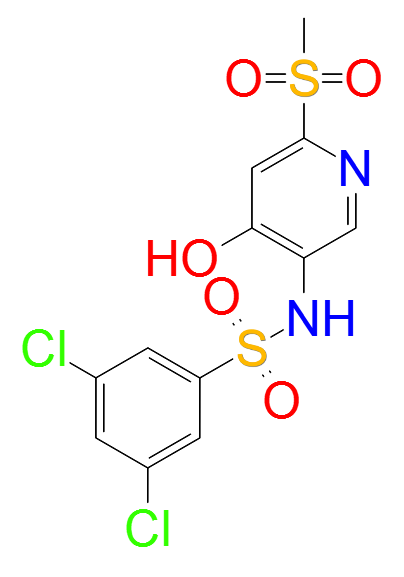

Supplement: DataSheet 1 — The 2D-structure of Dataset in Table S1. [file Data_Sheet_1.ZIP › Dataset138.png]

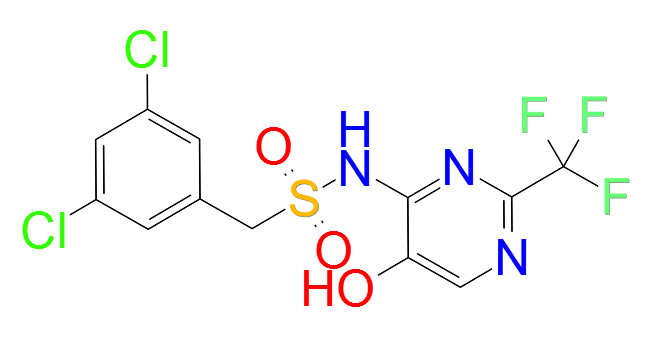

Supplement: DataSheet 1 — The 2D-structure of Dataset in Table S1. [file Data_Sheet_1.ZIP › Dataset139.png]

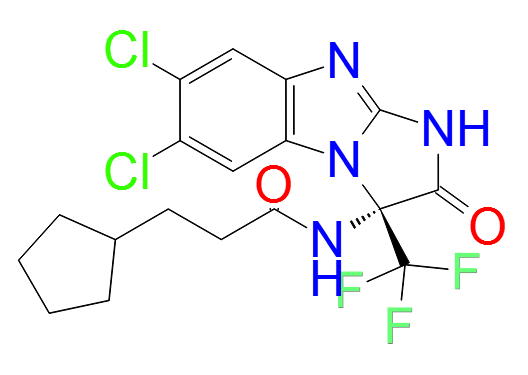

Supplement: DataSheet 1 — The 2D-structure of Dataset in Table S1. [file Data_Sheet_1.ZIP › Dataset14.png]

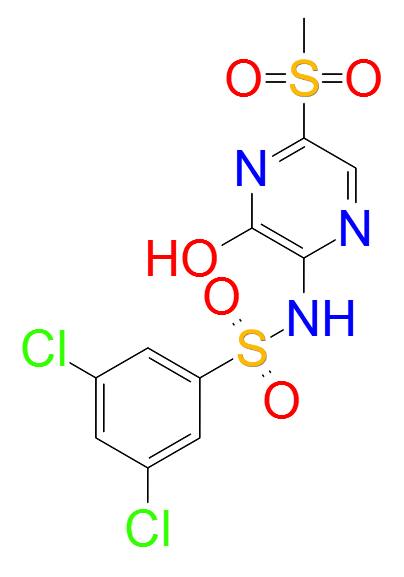

Supplement: DataSheet 1 — The 2D-structure of Dataset in Table S1. [file Data_Sheet_1.ZIP › Dataset140.png]

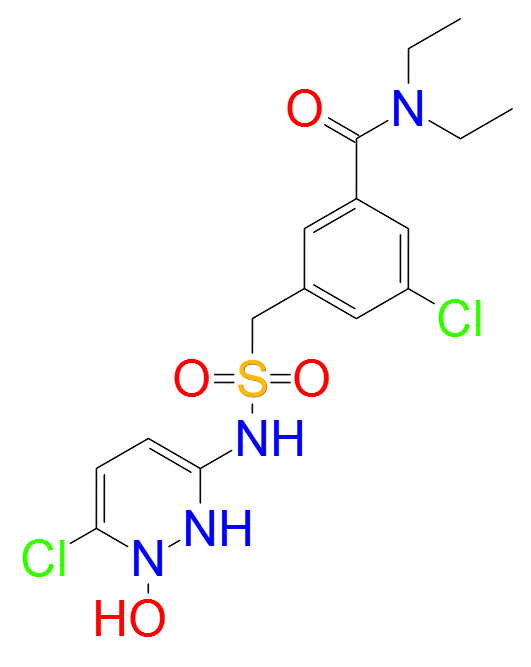

Supplement: DataSheet 1 — The 2D-structure of Dataset in Table S1. [file Data_Sheet_1.ZIP › Dataset141.png]

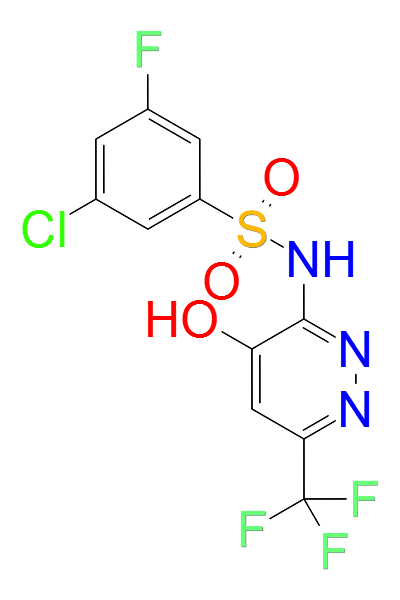

Supplement: DataSheet 1 — The 2D-structure of Dataset in Table S1. [file Data_Sheet_1.ZIP › Dataset142.png]

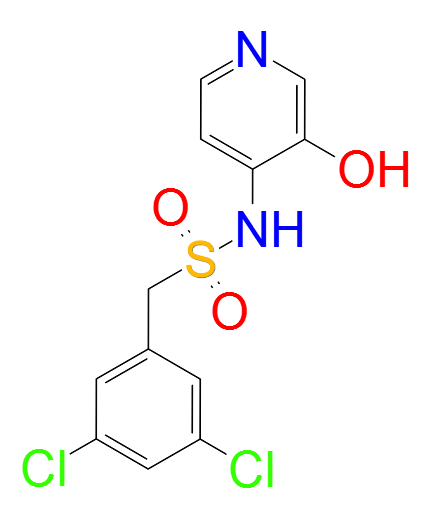

Supplement: DataSheet 1 — The 2D-structure of Dataset in Table S1. [file Data_Sheet_1.ZIP › Dataset143.png]

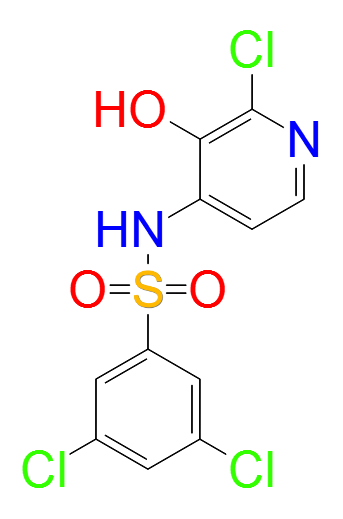

Supplement: DataSheet 1 — The 2D-structure of Dataset in Table S1. [file Data_Sheet_1.ZIP › Dataset144.png]

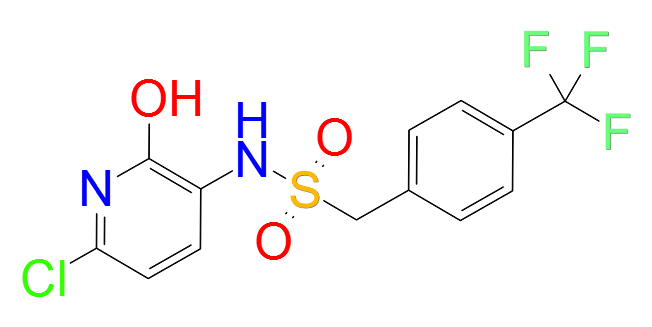

Supplement: DataSheet 1 — The 2D-structure of Dataset in Table S1. [file Data_Sheet_1.ZIP › Dataset145.png]

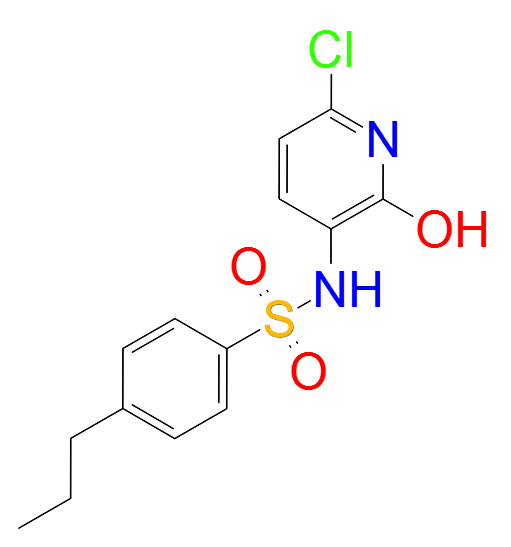

Supplement: DataSheet 1 — The 2D-structure of Dataset in Table S1. [file Data_Sheet_1.ZIP › Dataset146.png]

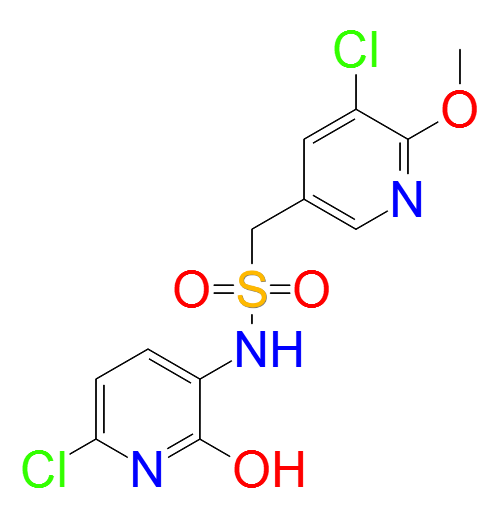

Supplement: DataSheet 1 — The 2D-structure of Dataset in Table S1. [file Data_Sheet_1.ZIP › Dataset147.png]

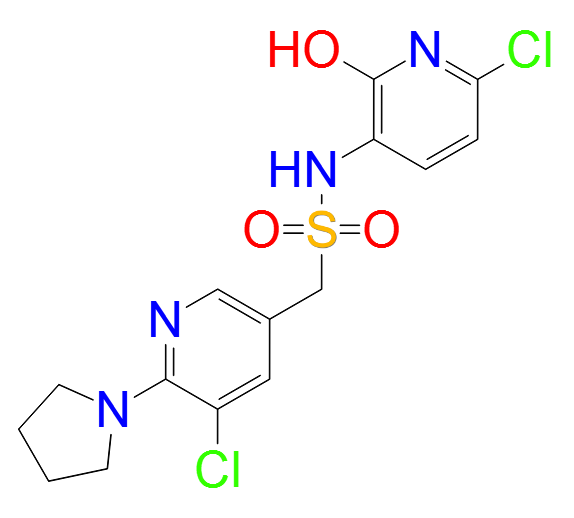

Supplement: DataSheet 1 — The 2D-structure of Dataset in Table S1. [file Data_Sheet_1.ZIP › Dataset148.png]

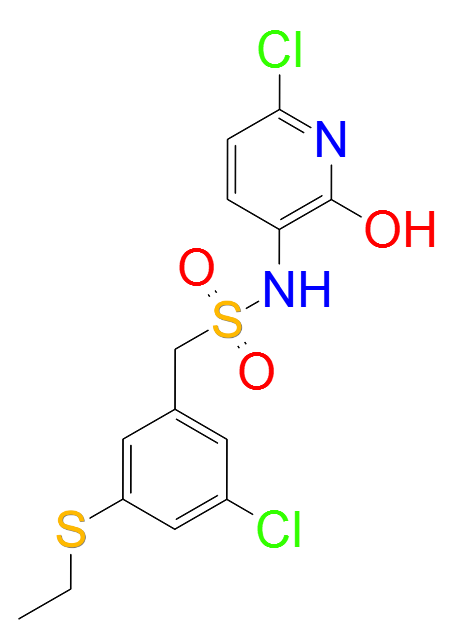

Supplement: DataSheet 1 — The 2D-structure of Dataset in Table S1. [file Data_Sheet_1.ZIP › Dataset149.png]

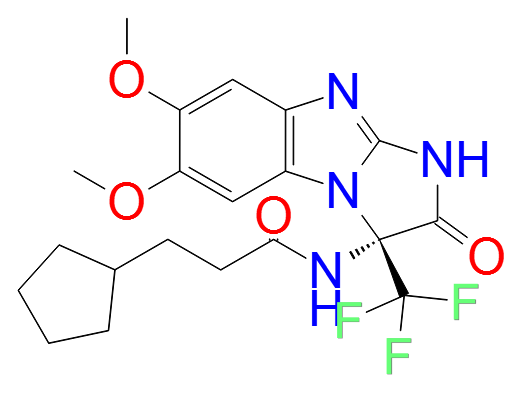

Supplement: DataSheet 1 — The 2D-structure of Dataset in Table S1. [file Data_Sheet_1.ZIP › Dataset15.png]

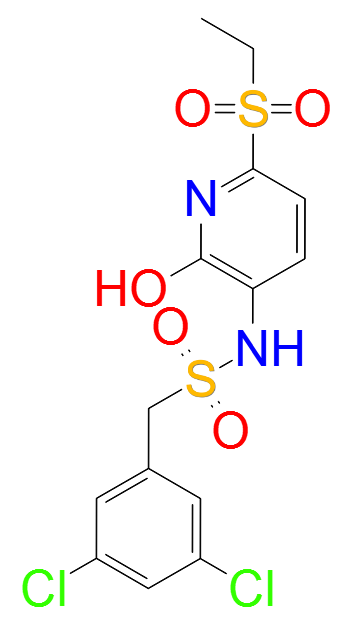

Supplement: DataSheet 1 — The 2D-structure of Dataset in Table S1. [file Data_Sheet_1.ZIP › Dataset150.png]

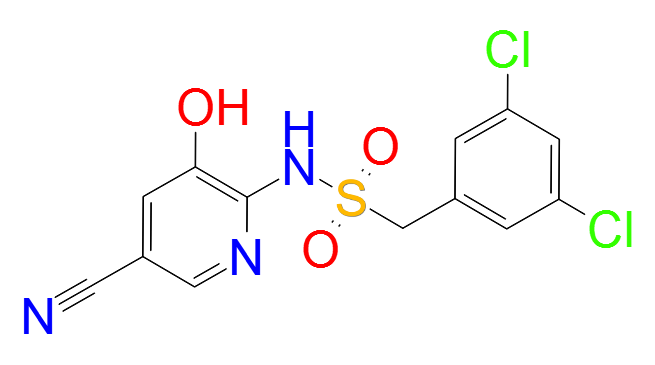

Supplement: DataSheet 1 — The 2D-structure of Dataset in Table S1. [file Data_Sheet_1.ZIP › Dataset151.png]

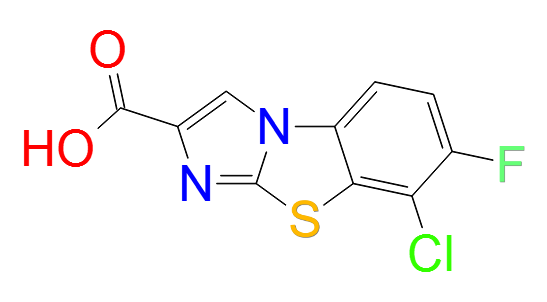

Supplement: DataSheet 1 — The 2D-structure of Dataset in Table S1. [file Data_Sheet_1.ZIP › Dataset152.png]

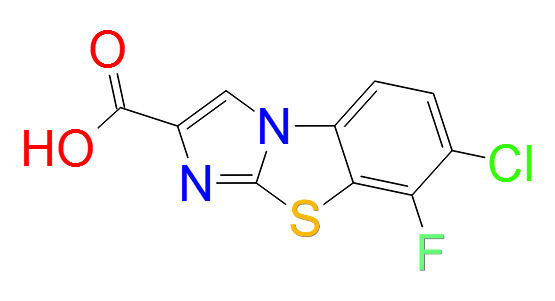

Supplement: DataSheet 1 — The 2D-structure of Dataset in Table S1. [file Data_Sheet_1.ZIP › Dataset153.png]

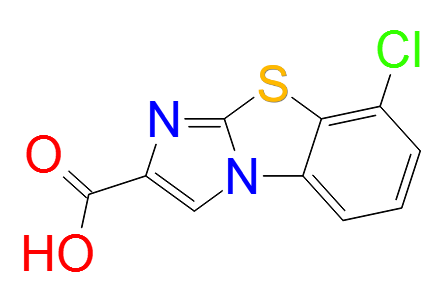

Supplement: DataSheet 1 — The 2D-structure of Dataset in Table S1. [file Data_Sheet_1.ZIP › Dataset154.png]

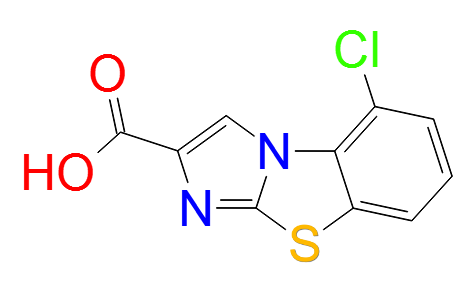

Supplement: DataSheet 1 — The 2D-structure of Dataset in Table S1. [file Data_Sheet_1.ZIP › Dataset155.png]

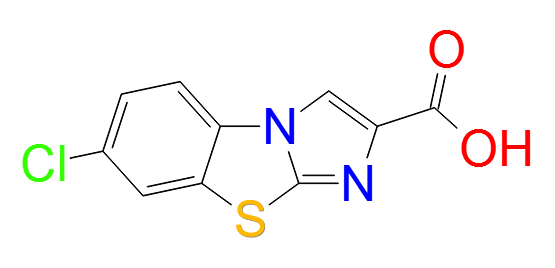

Supplement: DataSheet 1 — The 2D-structure of Dataset in Table S1. [file Data_Sheet_1.ZIP › Dataset156.png]

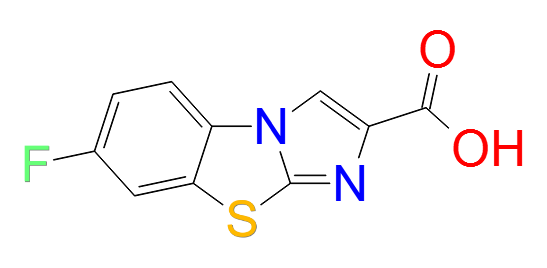

Supplement: DataSheet 1 — The 2D-structure of Dataset in Table S1. [file Data_Sheet_1.ZIP › Dataset157.png]

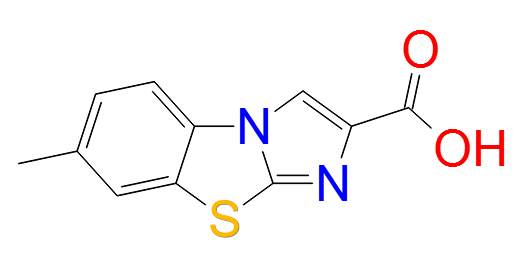

Supplement: DataSheet 1 — The 2D-structure of Dataset in Table S1. [file Data_Sheet_1.ZIP › Dataset158.png]

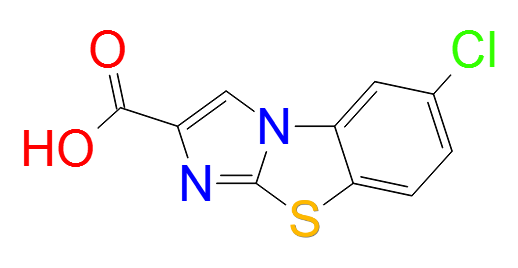

Supplement: DataSheet 1 — The 2D-structure of Dataset in Table S1. [file Data_Sheet_1.ZIP › Dataset159.png]

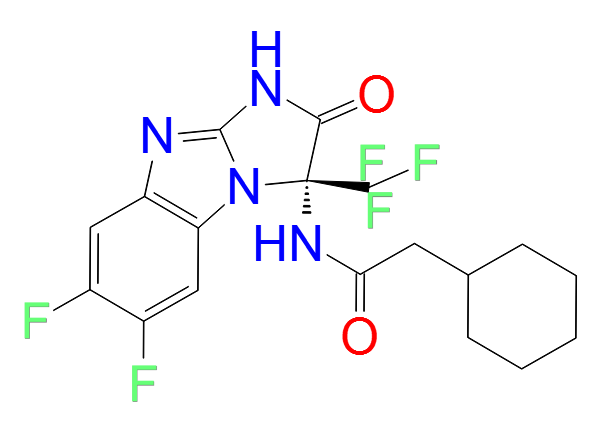

Supplement: DataSheet 1 — The 2D-structure of Dataset in Table S1. [file Data_Sheet_1.ZIP › Dataset16.png]

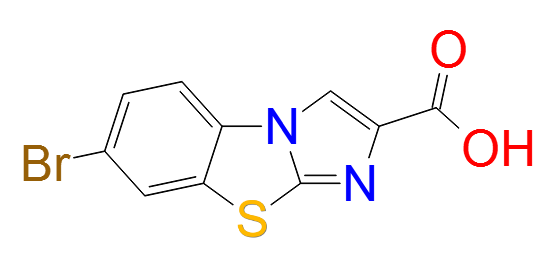

Supplement: DataSheet 1 — The 2D-structure of Dataset in Table S1. [file Data_Sheet_1.ZIP › Dataset160.png]

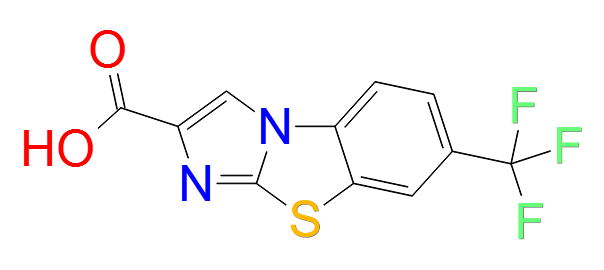

Supplement: DataSheet 1 — The 2D-structure of Dataset in Table S1. [file Data_Sheet_1.ZIP › Dataset161.png]

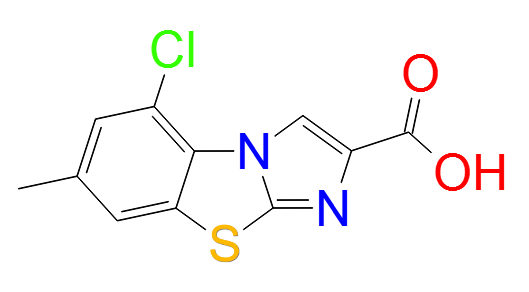

Supplement: DataSheet 1 — The 2D-structure of Dataset in Table S1. [file Data_Sheet_1.ZIP › Dataset162.png]

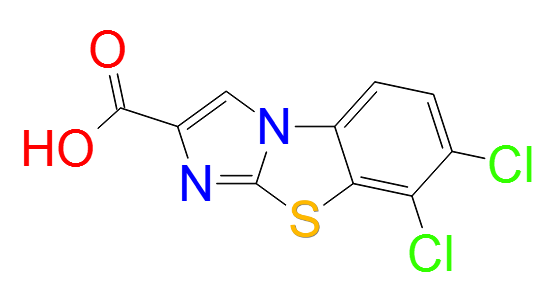

Supplement: DataSheet 1 — The 2D-structure of Dataset in Table S1. [file Data_Sheet_1.ZIP › Dataset163.png]

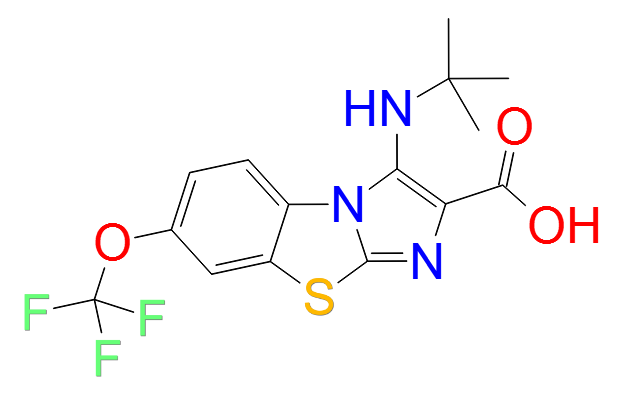

Supplement: DataSheet 1 — The 2D-structure of Dataset in Table S1. [file Data_Sheet_1.ZIP › Dataset164.png]

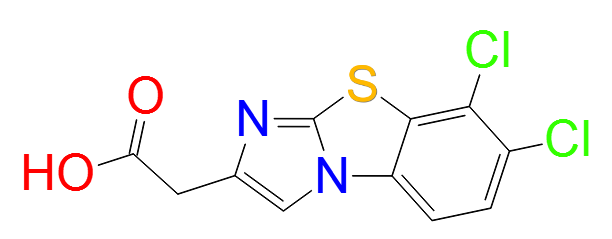

Supplement: DataSheet 1 — The 2D-structure of Dataset in Table S1. [file Data_Sheet_1.ZIP › Dataset165.png]

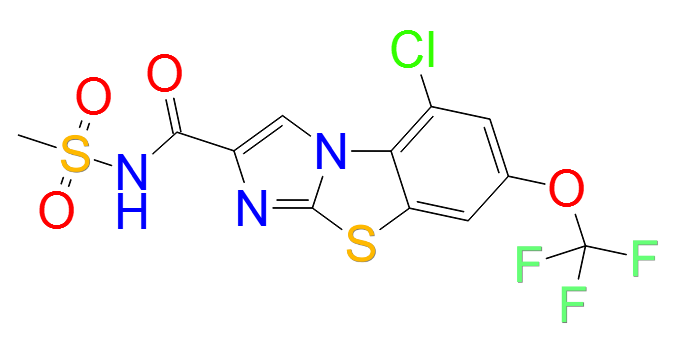

Supplement: DataSheet 1 — The 2D-structure of Dataset in Table S1. [file Data_Sheet_1.ZIP › Dataset166.png]

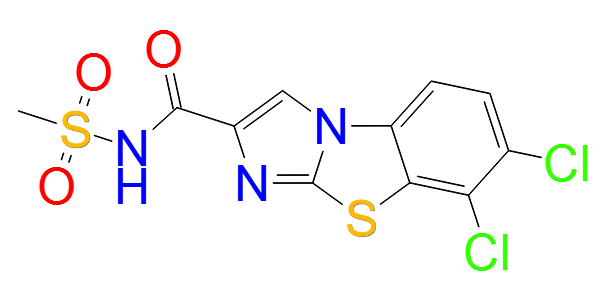

Supplement: DataSheet 1 — The 2D-structure of Dataset in Table S1. [file Data_Sheet_1.ZIP › Dataset167.png]

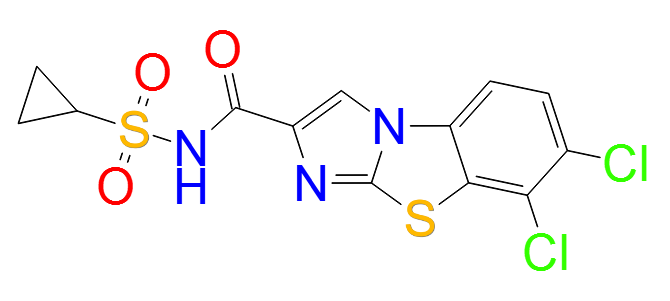

Supplement: DataSheet 1 — The 2D-structure of Dataset in Table S1. [file Data_Sheet_1.ZIP › Dataset168.png]

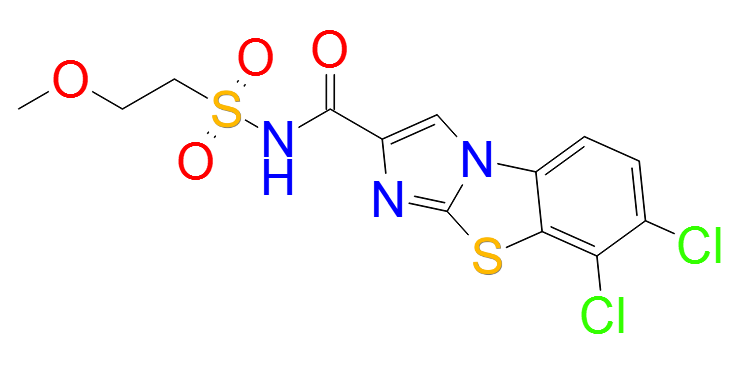

Supplement: DataSheet 1 — The 2D-structure of Dataset in Table S1. [file Data_Sheet_1.ZIP › Dataset169.png]

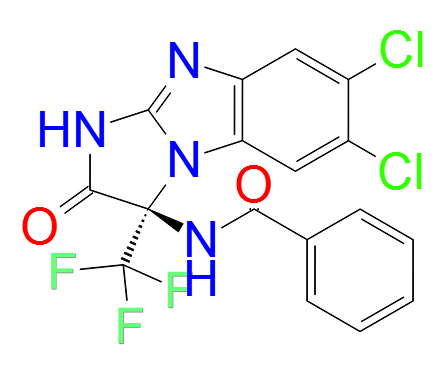

Supplement: DataSheet 1 — The 2D-structure of Dataset in Table S1. [file Data_Sheet_1.ZIP › Dataset17.png]

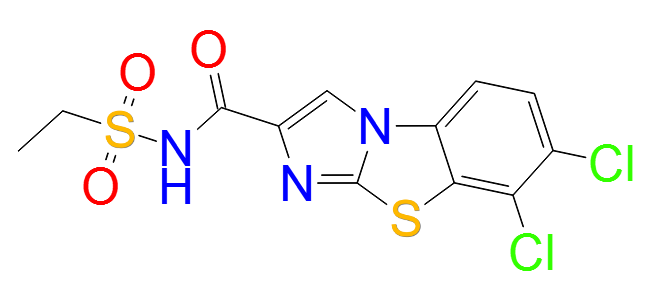

Supplement: DataSheet 1 — The 2D-structure of Dataset in Table S1. [file Data_Sheet_1.ZIP › Dataset170.png]

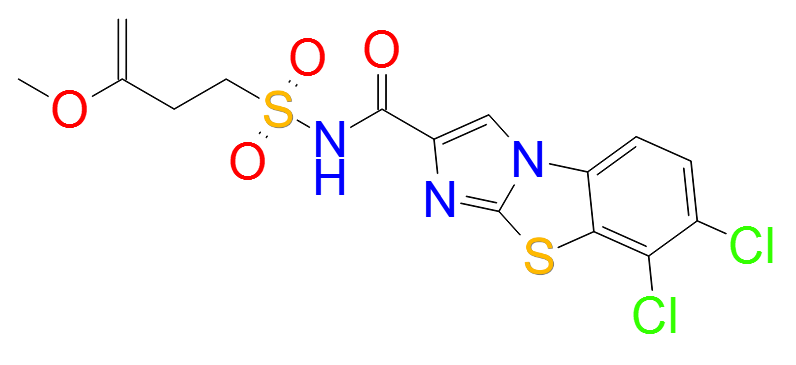

Supplement: DataSheet 1 — The 2D-structure of Dataset in Table S1. [file Data_Sheet_1.ZIP › Dataset171.png]

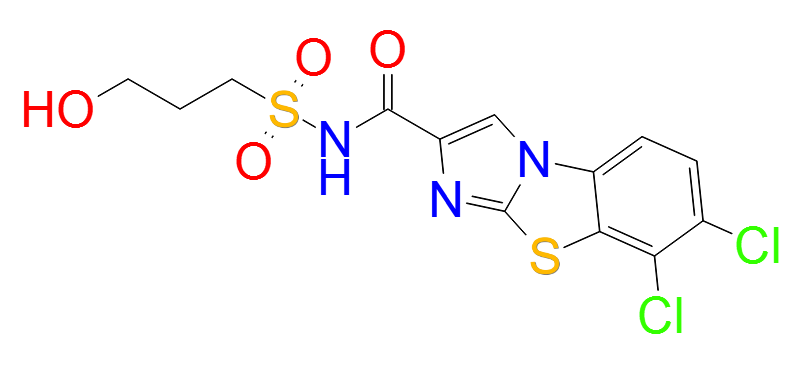

Supplement: DataSheet 1 — The 2D-structure of Dataset in Table S1. [file Data_Sheet_1.ZIP › Dataset172.png]

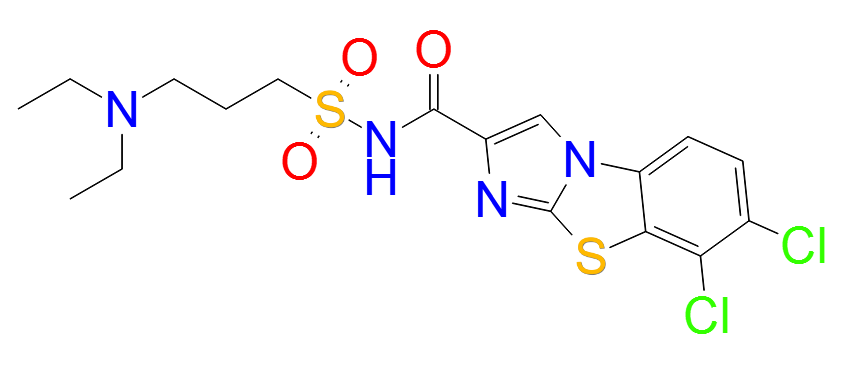

Supplement: DataSheet 1 — The 2D-structure of Dataset in Table S1. [file Data_Sheet_1.ZIP › Dataset173.png]

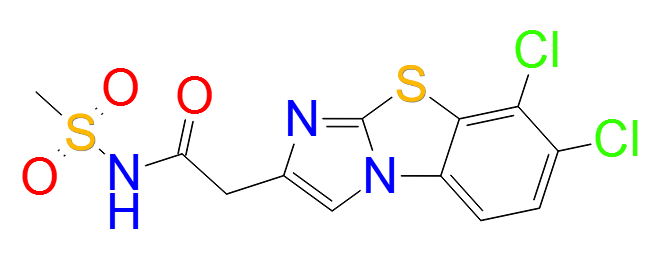

Supplement: DataSheet 1 — The 2D-structure of Dataset in Table S1. [file Data_Sheet_1.ZIP › Dataset174.png]

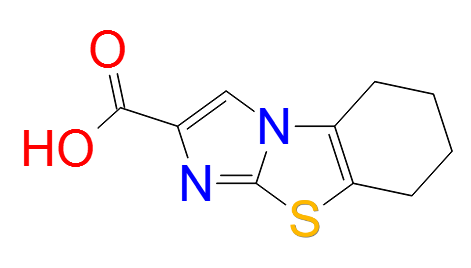

Supplement: DataSheet 1 — The 2D-structure of Dataset in Table S1. [file Data_Sheet_1.ZIP › Dataset175.png]

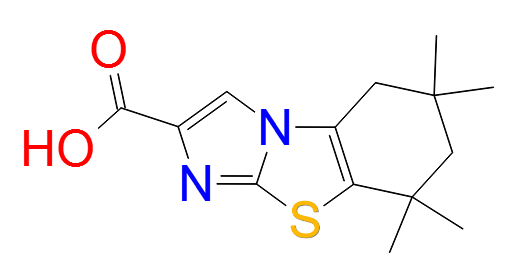

Supplement: DataSheet 1 — The 2D-structure of Dataset in Table S1. [file Data_Sheet_1.ZIP › Dataset176.png]

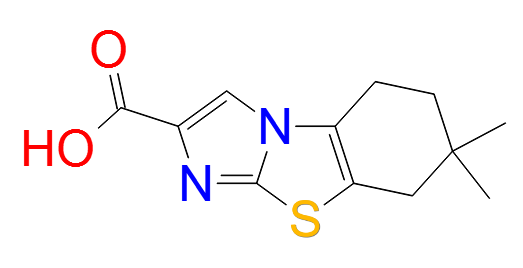

Supplement: DataSheet 1 — The 2D-structure of Dataset in Table S1. [file Data_Sheet_1.ZIP › Dataset177.png]

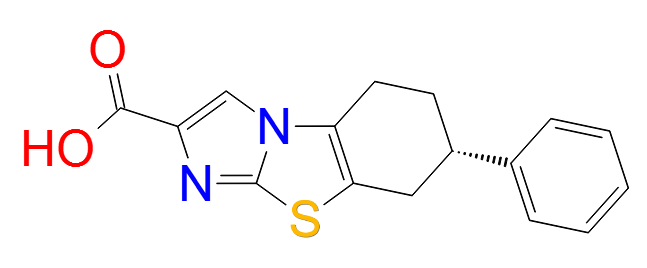

Supplement: DataSheet 1 — The 2D-structure of Dataset in Table S1. [file Data_Sheet_1.ZIP › Dataset178.png]

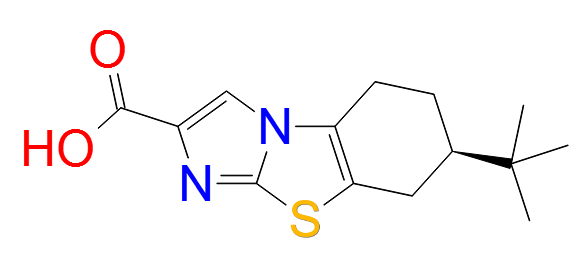

Supplement: DataSheet 1 — The 2D-structure of Dataset in Table S1. [file Data_Sheet_1.ZIP › Dataset179.png]

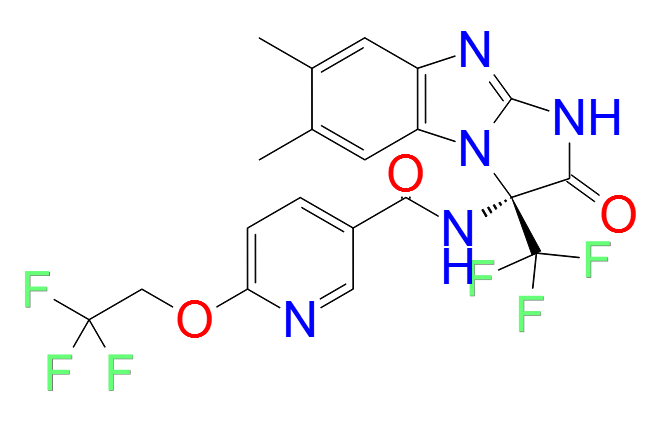

Supplement: DataSheet 1 — The 2D-structure of Dataset in Table S1. [file Data_Sheet_1.ZIP › Dataset18.png]

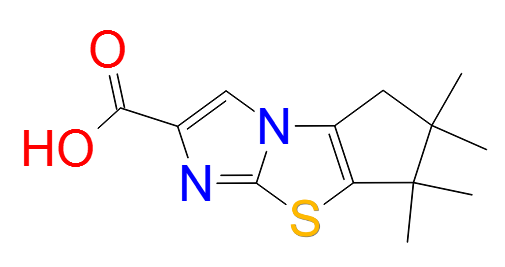

Supplement: DataSheet 1 — The 2D-structure of Dataset in Table S1. [file Data_Sheet_1.ZIP › Dataset180.png]

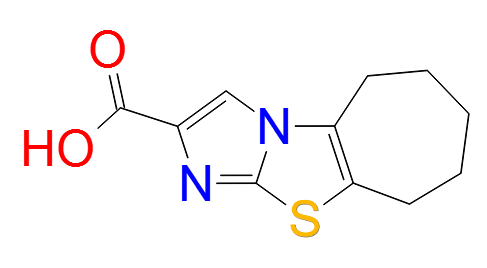

Supplement: DataSheet 1 — The 2D-structure of Dataset in Table S1. [file Data_Sheet_1.ZIP › Dataset181.png]

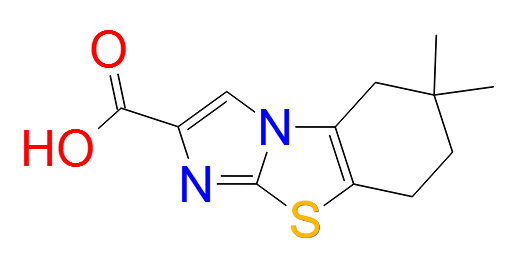

Supplement: DataSheet 1 — The 2D-structure of Dataset in Table S1. [file Data_Sheet_1.ZIP › Dataset182.png]

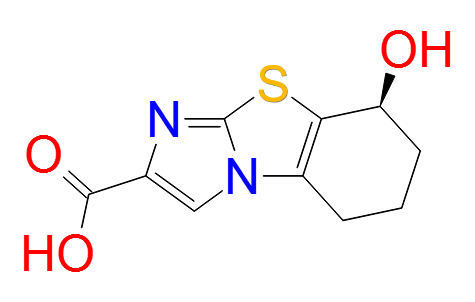

Supplement: DataSheet 1 — The 2D-structure of Dataset in Table S1. [file Data_Sheet_1.ZIP › Dataset183.png]

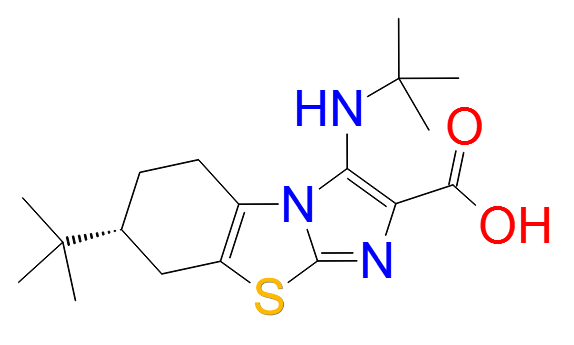

Supplement: DataSheet 1 — The 2D-structure of Dataset in Table S1. [file Data_Sheet_1.ZIP › Dataset184.png]

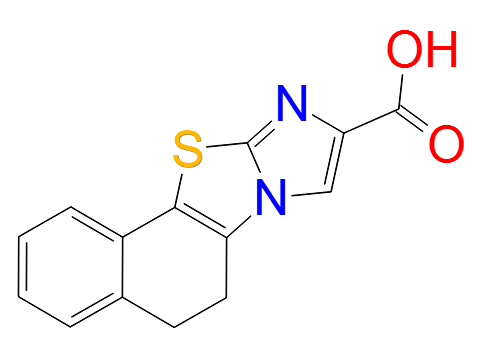

Supplement: DataSheet 1 — The 2D-structure of Dataset in Table S1. [file Data_Sheet_1.ZIP › Dataset185.png]

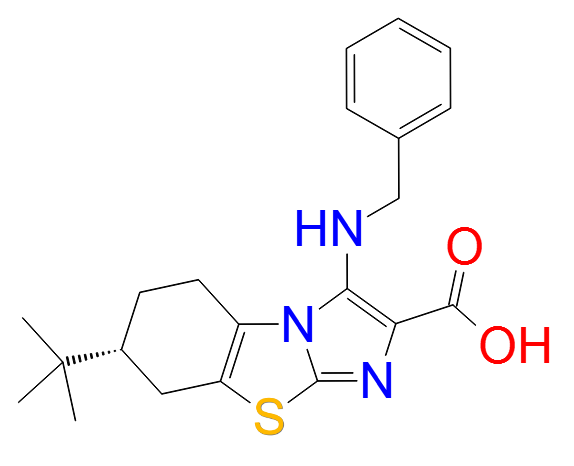

Supplement: DataSheet 1 — The 2D-structure of Dataset in Table S1. [file Data_Sheet_1.ZIP › Dataset186.png]

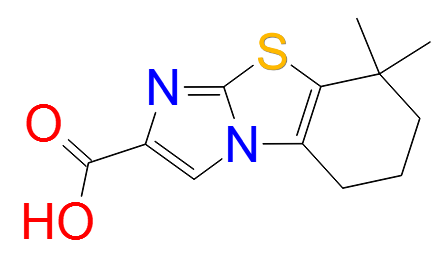

Supplement: DataSheet 1 — The 2D-structure of Dataset in Table S1. [file Data_Sheet_1.ZIP › Dataset187.png]

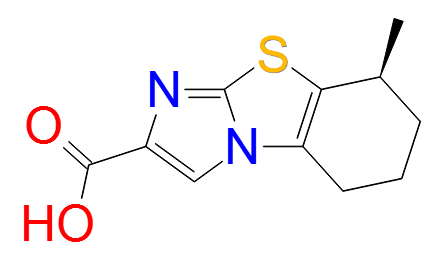

Supplement: DataSheet 1 — The 2D-structure of Dataset in Table S1. [file Data_Sheet_1.ZIP › Dataset188.png]

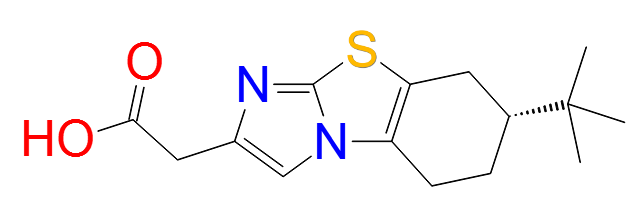

Supplement: DataSheet 1 — The 2D-structure of Dataset in Table S1. [file Data_Sheet_1.ZIP › Dataset189.png]
