# Supplementary material for: Development of Predictive Models for Identifying Potential S100A9 Inhibitors Based on Machine Learning Methods
Source: Front Chem. 2019 Nov 25;7:779. doi: 10.3389/fchem.2019.00779 (PMC6886474; doi:10.3389/fchem.2019.00779)
Supplement: Supplementary file 2 [file Table_1.docx]

**Supplementary Materials**

**Table S1.** The SMILES information of the dataset composed of active molecules (Compound 1~266) and decoys (Compound 267~402).

| Compound | SMILES |
| --- | --- |
| 1 | FC(F)(F)[C@]1(NC(=O)CCC2CCCCC2)C(=O)Nc2n1c1c(n2)cccc1 |
| 2 | FC(F)(F)[C@]1(NC(=O)c2ccccc2)C(=O)Nc2n1c1c(n2)cc(C)c(C)c1 |
| 3 | FC(F)(F)[C@]1(NC(=O)CCc2ccccc2)C(=O)Nc2n1c1c(n2)cc(C)c(C)c1 |
| 4 | FC(F)(F)[C@]1(NC(=O)CCC2CCCC2)C(=O)Nc2n1c1c(n2)cc(C)c(C)c1 |
| 5 | FC(F)(F)[C@]1(NC(=O)CCC2CCCC2)C(=O)Nc2n1c1c(n2)cccc1 |
| 6 | FC(F)(F)[C@]1(NC(=O)c2cc(OC)cc(OC)c2)C(=O)Nc2n1c1c(n2)cccc1 |
| 7 | FC(F)(F)[C@]1(NC(=O)CCC2CCCCC2)C(=O)Nc2n1c1c(n2)cc(C)c(C)c1 |
| 8 | FC(F)(F)[C@]1(NC(=O)CCCC2CCCC2)C(=O)Nc2n1c1c(n2)cc(C)c(C)c1 |
| 9 | FC(F)(F)[C@]1(NC(=O)CC2CCCCC2)C(=O)Nc2n1c1c(n2)cc(C)c(C)c1 |
| 10 | Clc1cc(Cl)cc(C(=O)N[C@@]2(C(F)(F)F)C(=O)Nc3n2c2c(n3)cccc2)c1 |
| 11 | Clc1c(CCC(=O)N[C@@]2(C(F)(F)F)C(=O)Nc3n2c2c(n3)cc(C)c(C)c2)cccc1 |
| 12 | Clc1ncc(C(=O)N[C@@]2(C(F)(F)F)C(=O)Nc3n2c2c(n3)cc(C)c(C)c2)cc1 |
| 13 | FC(F)(F)[C@]1(NC(=O)CCC2CCCC2)C(=O)Nc2n1c1c(n2)cc(F)c(F)c1 |
| 14 | Clc1c(Cl)cc2nc3n([C@](NC(=O)CCC4CCCC4)(C(F)(F)F)C(=O)N3)c2c1 |
| 15 | FC(F)(F)[C@]1(NC(=O)CCC2CCCC2)C(=O)Nc2n1c1c(n2)cc(OC)c(OC)c1 |
| 16 | FC(F)(F)[C@]1(NC(=O)CC2CCCCC2)C(=O)Nc2n1c1c(n2)cc(F)c(F)c1 |
| 17 | Clc1c(Cl)cc2nc3n([C@](NC(=O)c4ccccc4)(C(F)(F)F)C(=O)N3)c2c1 |
| 18 | FC(F)(F)COc1ncc(C(=O)N[C@@]2(C(F)(F)F)C(=O)Nc3n2c2c(n3)cc(C)c(C)c2)cc1 |
| 19 | FC(F)(F)[C@]1(NC(=O)c2nc(NC3CCCCC3)ccc2)C(=O)Nc2n1c1c(n2)cc(C)c(C)c1 |
| 20 | Clc1c(CCC(=O)N[C@@]2(C(F)(F)F)C(=O)Nc3n2c2c(n3)cc(C)c(C)c2)c(Cl)ccc1 |
| 21 | Clc1c(Cl)cc2nc3n([C@](NC(=O)CCc4ccccc4)(C(F)(F)F)C(=O)N3)c2c1 |
| 22 | Clc1c(Cl)cc2nc3n([C@](NC(=O)CCC4CCCCC4)(C(F)(F)F)C(=O)N3)c2c1 |
| 23 | FC(F)(F)[C@]1(NC(=O)c2nc(C3CCCCC3)ccc2)C(=O)Nc2n1c1c(n2)cc(C)c(C)c1 |
| 24 | FC(F)(F)[C@]1(NC(=O)c2ccc(-c3ccccc3)cc2)C(=O)Nc2n1c1c(n2)cc(C)c(C)c1 |
| 25 | FC(F)(F)[C@]1(NC(=O)c2cc(NCC)ccc2)C(=O)Nc2n1c1c(n2)cc(C)c(C)c1 |
| 26 | Clc1c(Cl)cc2nc3n([C@](NC(=O)C4CCCC4)(C(F)(F)F)C(=O)N3)c2c1 |
| 27 | Clc1c(Cl)cc2nc3n([C@](NC(=O)C4CCCCC4)(C(F)(F)F)C(=O)N3)c2c1 |
| 28 | Clc1c(Cl)cc2nc3n([C@](NC(=O)[C@@H]4CNCC4)(C(F)(F)F)C(=O)N3)c2c1 |
| 29 | Clc1c(Cl)cc2nc3n([C@](NC(=O)[C@@H]4CN(C(=O)C5CCCC5)CC4)(C(F)(F)F)C(=O)N3)c2c1 |
| 30 | Clc1c(Cl)cc2nc3n([C@](NC(=O)[C@@H]4CN(S(=O)(=O)C5CCCC5)CC4)(C(F)(F)F)C(=O)N3)c2c1 |
| 31 | Brc1c(C)c(C)cc2n3[C@](NC(=O)c4ccccc4)(C(F)(F)F)C(=O)Nc3nc12 |
| 32 | Clc1c(Cl)cc2nc3n([C@](NC(=O)C4CCN(C(=O)COC)CC4)(C(F)(F)F)C(=O)N3)c2c1 |
| 33 | Clc1c(Cl)cc2nc3n([C@](NC(=O)C4CCN(CCC(=O)C)CC4)(C(F)(F)F)C(=O)N3)c2c1 |
| 34 | Clc1c(Cl)cc2nc3n([C@](NC(=O)C4CCN(C(=O)CC(F)(F)F)CC4)(C(F)(F)F)C(=O)N3)c2c1 |
| 35 | Clc1c(Cl)cc2nc3n([C@](NC(=O)C4CNC4)(C(F)(F)F)C(=O)N3)c2c1 |
| 36 | Clc1c(Cl)cc2nc3n([C@](NC(=O)CCN4CCOCC4)(C(F)(F)F)C(=O)N3)c2c1 |
| 37 | FC(F)(F)[C@]1(NC(=O)CCC2CCCC2)C(=O)Nc2n1c1c(n2)cc2c(c1)CCC2 |
| 38 | Clc1cc2nc3n([C@](/N=C(/CCC4CCCC4)\C)(C(F)(F)F)C(=O)N3)c2cc1 |
| 39 | FC(F)(F)[C@]1(NC(=O)CCC2CCCC2)C(=O)Nc2n1c1c(c(C)c(C)cc1)n2 |
| 40 | FC(F)(F)[C@]1(NC(=O)CCC2CCCC2)C(=O)Nc2n1c1c(c(C)ccc1)n2 |
| 41 | Clc1c(Cl)cc2nc3n([C@](NC(=O)c4nc(NCC)ccc4)(C(F)(F)F)C(=O)N3)c2c1 |
| 42 | Clc1c(Cl)cc2nc3n([C@](NC(=O)c4nc(Cl)ccc4)(C(F)(F)F)C(=O)N3)c2c1 |
| 43 | Clc1c(Cl)cc2nc3n([C@](NC(=O)CN4CCOCC4)(C(F)(F)F)C(=O)N3)c2c1 |
| 44 | FC(F)(F)[C@]1(NC(=O)CCC2CCCC2)C(=O)Nc2n1c1c(c(F)c(F)cc1)n2 |
| 45 | Clc1c(Cl)ccc2n3[C@](NC(=O)CCC4CCCC4)(C(F)(F)F)C(=O)Nc3nc12 |
| 46 | Clc1c(Cl)cc2nc3n([C@](NC(=O)c4cc(OCC(F)(F)F)ccc4)(C(F)(F)F)C(=O)N3)c2c1 |
| 47 | Clc1c(Cl)cc2nc3n([C@](NC(=O)c4nc(N(C)C)ccc4)(C(F)(F)F)C(=O)N3)c2c1 |
| 48 | Clc1c(Cl)cc2nc3n([C@](NC(=O)CC[C@@H]4NCCCC4)(C(F)(F)F)C(=O)N3)c2c1 |
| 49 | Clc1c2nc3n([C@](NC(=O)CCC4CCCC4)(C(F)(F)F)C(=O)N3)c2cc(Cl)c1 |
| 50 | Clc1c2nc3n([C@](NC(=O)CCC4CCCC4)(C(F)(F)F)C(=O)N3)c2ccc1 |
| 51 | Clc1c(Cl)cc2nc3n([C@](NC(=O)C4CN(C(=O)C)C4)(C(F)(F)F)C(=O)N3)c2c1 |
| 52 | Clc1c(Cl)cc2nc3n([C@](NC(=O)C4CN(c5ncccc5)C4)(C(F)(F)F)C(=O)N3)c2c1 |
| 53 | Clc1c(Cl)cc2nc3n([C@](NC(=O)c4nc(NC5CCCCC5)ccc4)(C(F)(F)F)C(=O)N3)c2c1 |
| 54 | Clc1c(Cl)cc2nc3n([C@](NC(=O)c4cc(Cl)ccc4)(C(F)(F)F)C(=O)N3)c2c1 |
| 55 | FC(F)(F)[C@]1(NC(=O)CCC2CCCC2)C(=O)Nc2n1c1c(c(F)cc(F)c1)n2 |
| 56 | Clc1c(Cl)cc2nc3n([C@](NC(=O)C4CC5(C4)CNC5)(C(F)(F)F)C(=O)N3)c2c1 |
| 57 | Clc1c(Cl)cc2nc3n([C@](NC(=O)[C@@H]4NCCC4)(C(F)(F)F)C(=O)N3)c2c1 |
| 58 | Clc1c(Cl)cc2nc3n([C@](NC(=O)c4cc(Cl)cc(Cl)c4)(C(F)(F)F)C(=O)N3)c2c1 |
| 59 | Clc1c(Cl)cc2nc3n([C@](NC(=O)c4nc(NCCOC)ccc4)(C(F)(F)F)C(=O)N3)c2c1 |
| 60 | Clc1c(Cl)cc2nc3n([C@](NC(=O)c4cc(C#N)ccc4)(C(F)(F)F)C(=O)N3)c2c1 |
| 61 | Clc1c(Cl)cc2nc3n([C@](NC(=O)c4nc(N(CCOC)C)ccc4)(C(F)(F)F)C(=O)N3)c2c1 |
| 62 | Clc1c(Cl)cc2nc3n([C@](NC(=O)c4nc(NCCO)ccc4)(C(F)(F)F)C(=O)N3)c2c1 |
| 63 | Clc1c(Cl)cc2nc3n([C@](NC(=O)c4nc(C(=O)OC)ccc4)(C(F)(F)F)C(=O)N3)c2c1 |
| 64 | Brc1cc(C(F)(F)F)c2nc3n([C@](NC(=O)CCC4CCCC4)(C(F)(F)F)C(=O)N3)c2c1 |
| 65 | Clc1c(C#N)c2nc3n([C@](NC(=O)c4nc(N[C@H](COC)C)ccc4)(C(F)(F)F)C(=O)N3)c2cc1 |
| 66 | Clc1c(Cl)cc2nc3n([C@](NC(=O)c4nc(NCCN(C)C)ccc4)(C(F)(F)F)C(=O)N3)c2c1 |
| 67 | Clc1c(Cl)cc2nc3n([C@](NC(=O)c4nc(CO)ccc4)(C(F)(F)F)C(=O)N3)c2c1 |
| 68 | FC(F)(F)[C@]1(NC(=O)[C@@H](O)c2ccccc2)C(=O)Nc2n1c1c(n2)cc(C)c(C)c1 |
| 69 | FC(F)(F)[C@]1(NC(=O)[C@@H](OC)c2ccccc2)C(=O)Nc2n1c1c(n2)cc(C)c(C)c1 |
| 70 | Clc1c(C#N)c2nc3n([C@](NC(=O)c4[nH]c(NCCOC)cc4)(C(F)(F)F)C(=O)N3)c2cc1 |
| 71 | FC(F)(F)[C@]1(NC(=O)c2nc(NCCOC)sc2)C(=O)Nc2n1c1c(n2)cccc1 |
| 72 | O=C(N[C@@]1(C(C)(C)C)C(=O)Nc2n1c1c(n2)cc(C)c(C)c1)[C@@H](C)c1ccccc1 |
| 73 | O=C(N[C@@]1(C(C)(C)C)C(=O)Nc2n1c1c(n2)cc(C)c(C)c1)[C@H](CC1CCCC1)C |
| 74 | S(=O)(=O)(C)N1[C@H](C(=O)N[C@@]2(C(F)(F)F)C(=O)Nc3n2c2c(n3)cc(C)c(C)c2)CCC1 |
| 75 | Clc1c(Cl)cc2nc3n([C@](NC(=O)C4(C)CNC4)(C(F)(F)F)C(=O)N3)c2c1 |
| 76 | Clc1c(Cl)ccc2n3[C@](NC(=O)CCC4(O)CCCC4)(C(F)(F)F)C(=O)Nc3nc12 |
| 77 | Brc1c2nc3n([C@](NC(=O)CCC4CCCC4)(C(F)(F)F)C(=O)N3)c2cc(Cl)c1 |
| 78 | Brc1c(F)c2nc3n([C@](NC(=O)CCC4CCCC4)(C(F)(F)F)C(=O)N3)c2cc1 |
| 79 | Clc1cc(C(=O)C)c2nc3n([C@](NC(=O)CCC4CCCC4)(C(F)(F)F)C(=O)N3)c2c1 |
| 80 | Clc1c(OC)cc2nc3n([C@](NC(=O)CCC4CCCC4)(C(F)(F)F)C(=O)N3)c2c1 |
| 81 | FC(F)(F)[C@]1(NC(=O)CCC2CCCC2)C(=O)Nc2n1c1c(c(-c3cnc(OC)cc3)ccc1)n2 |
| 82 | FC(F)(F)[C@]1(NC(=O)CCC2CCCC2)C(=O)Nc2n1c1c(c(-c3cc(OC)ccc3)ccc1)n2 |
| 83 | FC(F)(F)[C@]1(NC(=O)CCC2CCCC2)C(=O)Nc2n1c1c(c(-c3ccc(C(C)(C)C)cc3)ccc1)n2 |
| 84 | FC(F)(F)[C@]1(NC(=O)CCC2CCCC2)C(=O)Nc2n1c1c(c(-c3ccc(NO)cc3)ccc1)n2 |
| 85 | FC(F)(F)[C@]1(NC(=O)CCC2CCCC2)C(=O)Nc2n1c1c(c(-c3ccccc3)ccc1)n2 |
| 86 | FC(F)(F)[C@]1(NC(=O)CCC2CCCC2)C(=O)Nc2n1c1c(c(-c3c(O)cccc3)ccc1)n2 |
| 87 | FC(F)(F)[C@]1(NC(=O)CCC2CCCC2)C(=O)Nc2n1c1c(c(/C(=N\N=NC)/N)ccc1)n2 |
| 88 | FC(F)(F)Oc1c2nc3n([C@](NC(=O)CCC4CCCC4)(C(F)(F)F)C(=O)N3)c2ccc1 |
| 89 | S(=O)(=O)(Nc1c2nc3n([C@](NC(=O)CCC4CCCC4)(C(F)(F)F)C(=O)N3)c2ccc1)C1CC1 |
| 90 | S(=O)(=O)(NC(=O)c1c2nc3n([C@](NC(=O)CCC4CCCC4)(C(F)(F)F)C(=O)N3)c2ccc1)C |
| 91 | Clc1ccc(-c2c3nc4n([C@](NC(=O)CCC5CCCC5)(C(F)(F)F)C(=O)N4)c3ccc2)cc1 |
| 92 | FC(F)(F)[C@]1(NC(=O)CCC2CCCC2)C(=O)Nc2n1c1c(c(-c3ccc(C)cc3)ccc1)n2 |
| 93 | Clc1c(Cl)ccc(-c2c3nc4n([C@](NC(=O)CCC5CCCC5)(C(F)(F)F)C(=O)N4)c3ccc2)c1 |
| 94 | FC(F)(F)[C@]1(NC(=O)CCC)C(=O)Nc2n1c1c(c(-c3ccc(OC)cc3)ccc1)n2 |
| 95 | Clc1c(-c2c3nc4n([C@](NC(=O)CCC5CCCC5)(C(F)(F)F)C(=O)N4)c3ccc2)ccc(C(=O)O)c1 |
| 96 | S(C)c1ccc(-c2c3nc4n([C@](NC(=O)CCC5CCCC5)(C(F)(F)F)C(=O)N4)c3ccc2)cc1 |
| 97 | Clc1c(Cl)cccc1-c1c2nc3n([C@](NC(=O)c4nc(NCC(C)C)sc4)(C(F)(F)F)C(=O)N3)c2ccc1 |
| 98 | Clc1cc(Cl)cc(CS(=O)(=O)Nc2c(O)cc(Cl)cn2)c1 |
| 99 | Brc1c(Cl)ncc(S(=O)(=O)Nc2c(O)cc(C(C)C)cn2)c1 |
| 100 | Clc1cc(Cl)cc(CS(=O)(=O)Nc2c(O)cc(S(=O)(=O)C)cn2)c1 |
| 101 | Clc1nnc(NS(=O)(=O)Cc2cc(Cl)cc(Cl)c2)c(O)c1 |
| 102 | Brc1cc(S(=O)(=O)Nc2c(O)cc(Cl)cn2)cnc1 |
| 103 | Clc1cc(O)c(NS(=O)(=O)c2cnc(C(F)(F)F)cc2)nc1 |
| 104 | Brc1c(OC)ncc(S(=O)(=O)Nc2c(O)ncc(Cl)c2)c1 |
| 105 | Brc1c(OC)ncc(S(=O)(=O)Nc2c(O)ccnc2)c1 |
| 106 | Clc1c(S(=O)(=O)Nc2c(O)ccnc2)cc(Cl)s1 |
| 107 | Clc1c(Cl)ccc(CS(=O)(=O)Nc2c(O)cc(Cl)nc2)c1 |
| 108 | Clc1c(S(=O)(=O)Nc2c(O)cc(Cl)nc2)cc(Cl)s1 |
| 109 | Clc1c(S(=O)(=O)Nc2c(O)cc(Cl)cn2)cc(Cl)s1 |
| 110 | Brc1nc(O)c(NS(=O)(=O)Cc2cc(Cl)c(Cl)cc2)nc1 |
| 111 | Clc1nnc(NS(=O)(=O)Cc2cc(F)c(F)cc2)c(O)c1 |
| 112 | Clc1c(CS(=O)(=O)Nc2c(O)cc(Cl)nn2)cc(C#N)cc1 |
| 113 | Clc1cc(CS(=O)(=O)Nc2c(O)nc(S(=O)(=O)CC)cn2)ccc1 |
| 114 | Clc1c(O)c(NS(=O)(=O)c2cc(Cl)cc(Cl)c2)cnc1 |
| 115 | Clc1nnc(NS(=O)(=O)Cc2cc(Cl)cc(F)c2)c(O)c1 |
| 116 | Clc1nnc(NS(=O)(=O)c2cc(Cl)cc(Cl)c2)c(O)c1 |
| 117 | Clc1nnc(NS(=O)(=O)Cc2cc(Cl)ccc2)c(O)c1 |
| 118 | Brc1c(O)c(NS(=O)(=O)Cc2cc(Cl)cc(Cl)c2)cnc1 |
| 119 | Clc1c(OC(F)(F)F)ccc(S(=O)(=O)Nc2c(O)c(Cl)cnc2)c1 |
| 120 | Clc1cc(Cl)cc(CS(=O)(=O)Nc2c(O)nc(C#N)cn2)c1 |
| 121 | Clc1nc(O)c(NS(=O)(=O)Cc2cc(Cl)cc(Cl)c2)cn1 |
| 122 | Clc1cc(Cl)cc(CS(=O)(=O)Nc2c(O)cc(S(=O)(=O)CC)nn2)c1 |
| 123 | Clc1cc(Cl)cc(CS(=O)(=O)Nc2c(O)nc(S(=O)(=O)C)cn2)c1 |
| 124 | Clc1c(S(=O)(=O)Nc2c(O)cc(Cl)nn2)cc(Cl)s1 |
| 125 | Brc1c(OC)ncc(S(=O)(=O)Nc2c(O)cc(Cl)nn2)c1 |
| 126 | Clc1c(OC)ccc(S(=O)(=O)Nc2c(O)c(Cl)cnc2)c1 |
| 127 | Clc1cc(O)c(NS(=O)(=O)Cc2cc(C#N)sc2)nc1 |
| 128 | Clc1c(C#N)nc(NS(=O)(=O)Cc2cc(Cl)cc(Cl)c2)c(O)c1 |
| 129 | Clc1cc(Cl)cc(CS(=O)(=O)Nc2c(O)cc(S(=O)(=O)C3CCCC3)cn2)c1 |
| 130 | Clc1c(S(=O)(=O)C)nc(NS(=O)(=O)Cc2cc(Cl)cc(Cl)c2)c(O)c1 |
| 131 | Clc1cc(O)c(NS(=O)(=O)c2cc(S(=O)(=O)C)c(N(C)C)nc2)nc1 |
| 132 | Brc1c(OC)ncc(S(=O)(=O)Nc2c(O)cc(C(F)(F)F)nc2)c1 |
| 133 | Clc1cc(Cl)cc(CS(=O)(=O)Nc2c(O)c(C#N)cnc2)c1 |
| 134 | Clc1cc(Cl)cc(CS(=O)(=O)Nc2c(O)cc(S(=O)(=O)CCCO)nn2)c1 |
| 135 | Brc1nc(O)c(NS(=O)(=O)[C@@H](C(F)(F)F)c2cc(Cl)cc(Cl)c2)nc1 |
| 136 | Clc1nc(NS(=O)(=O)Cc2cc(Cl)cc(Cl)c2)c(O)cn1 |
| 137 | Clc1nc(NS(=O)(=O)c2cc(Cl)c(Cl)cc2)c(O)cn1 |
| 138 | Clc1cc(Cl)cc(S(=O)(=O)Nc2c(O)cc(S(=O)(=O)C)nc2)c1 |
| 139 | Clc1cc(Cl)cc(CS(=O)(=O)Nc2c(O)cnc(C(F)(F)F)n2)c1 |
| 140 | Clc1cc(Cl)cc(S(=O)(=O)Nc2c(O)nc(S(=O)(=O)C)cn2)c1 |
| 141 | Clc1cc(C(=O)N(CC)CC)cc(CS(=O)(=O)NC=2NN(O)C(Cl)=CC=2)c1 |
| 142 | Clc1cc(S(=O)(=O)Nc2c(O)cc(C(F)(F)F)nn2)cc(F)c1 |
| 143 | Clc1cc(Cl)cc(CS(=O)(=O)Nc2c(O)cncc2)c1 |
| 144 | Clc1c(O)c(NS(=O)(=O)c2cc(Cl)cc(Cl)c2)ccn1 |
| 145 | Clc1nc(O)c(NS(=O)(=O)Cc2ccc(C(F)(F)F)cc2)cc1 |
| 146 | Clc1nc(O)c(NS(=O)(=O)c2ccc(CCC)cc2)cc1 |
| 147 | Clc1c(OC)ncc(CS(=O)(=O)Nc2c(O)nc(Cl)cc2)c1 |
| 148 | Clc1c(N2CCCC2)ncc(CS(=O)(=O)Nc2c(O)nc(Cl)cc2)c1 |
| 149 | Clc1nc(O)c(NS(=O)(=O)Cc2cc(Cl)cc(SCC)c2)cc1 |
| 150 | Clc1cc(Cl)cc(CS(=O)(=O)Nc2c(O)nc(S(=O)(=O)CC)cc2)c1 |
| 151 | Clc1cc(Cl)cc(CS(=O)(=O)Nc2c(O)cc(C#N)cn2)c1 |
| 152 | Clc1c(F)ccc2-n3c(Sc12)nc(C(=O)O)c3 |
| 153 | Clc1c(F)c2Sc3n(-c2cc1)cc(C(=O)O)n3 |
| 154 | Clc1c2Sc3n(-c2ccc1)cc(C(=O)O)n3 |
| 155 | Clc1c2-n3c(Sc2ccc1)nc(C(=O)O)c3 |
| 156 | Clc1cc2Sc3n(-c2cc1)cc(C(=O)O)n3 |
| 157 | Fc1cc2Sc3n(-c2cc1)cc(C(=O)O)n3 |
| 158 | O=C(O)c1nc2Sc3c(-n2c1)ccc(C)c3 |
| 159 | Clc1cc2-n3c(Sc2cc1)nc(C(=O)O)c3 |
| 160 | Brc1cc2Sc3n(-c2cc1)cc(C(=O)O)n3 |
| 161 | FC(F)(F)c1cc2Sc3n(-c2cc1)cc(C(=O)O)n3 |
| 162 | Clc1c2-n3c(Sc2cc(C)c1)nc(C(=O)O)c3 |
| 163 | Clc1c(Cl)ccc2-n3c(Sc12)nc(C(=O)O)c3 |
| 164 | FC(F)(F)Oc1cc2Sc3n(c(NC(C)(C)C)c(C(=O)O)n3)-c2cc1 |
| 165 | Clc1c(Cl)ccc2-n3c(Sc12)nc(CC(=O)O)c3 |
| 166 | Clc1c2-n3c(Sc2cc(OC(F)(F)F)c1)nc(C(=O)NS(=O)(=O)C)c3 |
| 167 | Clc1c(Cl)ccc2-n3c(Sc12)nc(C(=O)NS(=O)(=O)C)c3 |
| 168 | Clc1c(Cl)ccc2-n3c(Sc12)nc(C(=O)NS(=O)(=O)C1CC1)c3 |
| 169 | Clc1c(Cl)ccc2-n3c(Sc12)nc(C(=O)NS(=O)(=O)CCOC)c3 |
| 170 | Clc1c(Cl)ccc2-n3c(Sc12)nc(C(=O)NS(=O)(=O)CC)c3 |
| 171 | Clc1c(Cl)ccc2-n3c(Sc12)nc(C(=O)NS(=O)(=O)CCC(OC)=C)c3 |
| 172 | Clc1c(Cl)ccc2-n3c(Sc12)nc(C(=O)NS(=O)(=O)CCCO)c3 |
| 173 | Clc1c(Cl)ccc2-n3c(Sc12)nc(C(=O)NS(=O)(=O)CCCN(CC)CC)c3 |
| 174 | Clc1c(Cl)ccc2-n3c(Sc12)nc(CC(=O)NS(=O)(=O)C)c3 |
| 175 | O=C(O)c1nc2SC3=C(n2c1)CCCC3 |
| 176 | O=C(O)c1nc2SC=3C(C)(C)CC(C)(C)CC=3n2c1 |
| 177 | O=C(O)c1nc2SC3=C(n2c1)CCC(C)(C)C3 |
| 178 | O=C(O)c1nc2SC3=C(n2c1)CC[C@H](c1ccccc1)C3 |
| 179 | O=C(O)c1nc2SC3=C(n2c1)CC[C@@H](C(C)(C)C)C3 |
| 180 | O=C(O)c1nc2SC=3C(C)(C)C(C)(C)CC=3n2c1 |
| 181 | O=C(O)c1nc2SC3=C(n2c1)CCCCC3 |
| 182 | O=C(O)c1nc2SC3=C(n2c1)CC(C)(C)CC3 |
| 183 | O=C(O)c1nc2SC=3[C@@H](O)CCCC=3n2c1 |
| 184 | O=C(O)c1c(NC(C)(C)C)n2c(SC3=C2CC[C@@H](C(C)(C)C)C3)n1 |
| 185 | O=C(O)c1nc2SC=3c4c(cccc4)CCC=3n2c1 |
| 186 | O=C(O)c1c(NCc2ccccc2)n2c(SC3=C2CC[C@@H](C(C)(C)C)C3)n1 |
| 187 | O=C(O)c1nc2SC=3C(C)(C)CCCC=3n2c1 |
| 188 | O=C(O)c1nc2SC=3[C@@H](C)CCCC=3n2c1 |
| 189 | O=C(O)Cc1nc2SC3=C(n2c1)CC[C@@H](C(C)(C)C)C3 |
| 190 | O=C(O)Cc1nc2SC=3C(C)(C)CC(C)(C)CC=3n2c1 |
| 191 | S(=O)(=O)(NC(=O)c1nc2SC3=C(n2c1)CC[C@@H](C(C)(C)C)C3)C |
| 192 | S(=O)(=O)(NC(=O)c1nc2SC=3[C@@H](C)CCCC=3n2c1)C |
| 193 | S(=O)(=O)(NC(=O)c1nc2SC3=C(n2c1)CC[C@@H](C(C)(C)C)C3)CCCN1CCOCC1 |
| 194 | S(=O)(=O)(NC(=O)c1nc2SC=3C(C)(C)C(C)(C)CC=3n2c1)C |
| 195 | S(=O)(=O)(NC(=O)c1nc2SC=3C(C)(C)CC(C)(C)CC=3n2c1)C |
| 196 | S(=O)(=O)(NC(=O)c1nc2SC=3C(C)(C)CC(C)(C)CC=3n2c1)CCCN(CC)CC |
| 197 | S(=O)(=O)(NC(=O)c1nc2SC=3C(C)(C)CC(C)(C)CC=3n2c1)CCCN1CCOCC1 |
| 198 | S(=O)(=O)(NC(=O)c1nc2SC3=C(n2c1)CC(C)(C)CC3)CC(F)(F)F |
| 199 | O=C(NC#N)c1nc2SC3=C(n2c1)CC[C@@H](C(C)(C)C)C3 |
| 200 | Clc1c(Cl)ccc2N3C(Sc12)=N[C@H](C(=O)O)C3 |
| 201 | Clc1c(Cl)ccc2-n3c(Sc12)nc([C@H](O)C(=O)O)c3 |
| 202 | O=C(O)c1c(-c2ccc(OC)cc2)n2c(SC3=C2CC[C@@H](C(C)(C)C)C3)n1 |
| 203 | O=C(O)c1c(C)n2c(SC3=C2CC[C@@H](C(C)(C)C)C3)n1 |
| 204 | S(=O)(=O)(Nc1c(C(=O)O)nc2SC3=C(n12)CC[C@@H](C(C)(C)C)C3)C |
| 205 | Clc1c2Sc3n(-c2c(Cl)cc1)cc(C(=O)O)n3 |
| 206 | FC(F)(F)Oc1c2Sc3n(-c2ccc1)cc(C(=O)O)n3 |
| 207 | S(C(F)(F)F)c1cc2Sc3n(-c2cc1)cc(C(=O)O)n3 |
| 208 | Clc1c(Cl)ccc2-n3c(CN(C)C)c(C(=O)O)nc3Sc12 |
| 209 | Clc1c2Sc3n(-c2cc(Cl)c1)cc(C(=O)O)n3 |
| 210 | O=C(O)c1nc2Sc3c(-n2c1)ccc(C(C)(C)C)c3 |
| 211 | Clc1c(C(=O)O)nc2SC=3C(C)(C)CC(C)(C)CC=3n12 |
| 212 | O=C(O)c1nc2Sc3c(-n2c1)ccc(OCc1ccccc1)c3 |
| 213 | Fc1c(F)ccc2-n3c(Sc12)nc(C(=O)O)c3 |
| 214 | Brc1c2Sc3n(-c2ccc1)cc(C(=O)O)n3 |
| 215 | Clc1c(OC(F)(F)F)ccc2-n3c(Sc12)nc(C(=O)O)c3 |
| 216 | Clc1c(OC)c2Sc3n(-c2cc1)cc(C(=O)O)n3 |
| 217 | Clc1c(OC)ccc2-n3c(Sc12)nc(C(=O)O)c3 |
| 218 | Clc1c(OC(F)(F)F)ccc2-n3c(Sc12)nc(C(=O)NS(=O)(=O)C)c3 |
| 219 | Brc1c(C(=O)O)nc2SC=3C(C)(C)CC(C)(C)CC=3n12 |
| 220 | O(C(C)(C)C)C(=O)N1CC(c2c(C(=O)O)nc3SC=4C(C)(C)CC(C)(C)CC=4n23)C1 |
| 221 | O=C(O)c1c(C#C)n2c(SC=3C(C)(C)CC(C)(C)CC2=3)n1 |
| 222 | Clc1c(OC(F)(F)F)cc2-n3c(Sc2c1)nc(C(=O)O)c3 |
| 223 | Clc1c2Sc3n(c(NS(=O)(=O)C)c(CO)n3)-c2ccc1 |
| 224 | O=C(O)c1c(N2CCOCC2)n2c(SC=3C(C)(C)CC(C)(C)CC2=3)n1 |
| 225 | Clc1c(F)ccc2-n3c(C)c(C(=O)O)nc3Sc12 |
| 226 | O=C(O)c1c(C2CNC2)n2c(SC=3C(C)(C)CC(C)(C)CC2=3)n1 |
| 227 | Clc1c(F)ccc2N3C(Sc12)=N[C@@H](C(=O)O)C3 |
| 228 | Brc1c(Cl)c2SC3=N[C@H](C(=O)O)CN3c2cc1 |
| 229 | Clc1c(Cl)cc(OCCN2CCOCC2)c2-n3c(Sc12)nc(C(=O)O)c3 |
| 230 | O=C(O)c1c(CN2CCOCC2)n2c(SC3=C2CC[C@@H](C(C)(C)C)C3)n1 |
| 231 | Brc1c(Cl)c2Sc3n(-c2cc1)cc(C(=O)O)n3 |
| 232 | O=C(O)c1c(CN2C(=O)CCCC2)n2c(SC3=C2CC[C@@H](C(C)(C)C)C3)n1 |
| 233 | O=C(O)c1c(C)n2c(SC=3C(C)(C)CC(C)(C)CC2=3)n1 |
| 234 | O=C(O)c1c(CN2CCOCC2)n2c(SC=3C(C)(C)CC(C)(C)CC2=3)n1 |
| 235 | O=C(O)c1c(-c2c(OC)cccc2)n2c(SC=3C(C)(C)CC(C)(C)CC2=3)n1 |
| 236 | Clc1c(N2CCCC2)ccc2-n3c(Sc12)nc(C(=O)O)c3 |
| 237 | Clc1c(F)ccc2N3C(Sc12)=N[C@](C(=O)O)(CC(C)C)C3 |
| 238 | Clc1c(C2CC2)ccc2-n3c(Sc12)nc(C(=O)O)c3 |
| 239 | Brc1c(C(=O)O)nc2SC3=C(n12)CC[C@@H](C(C)(C)C)C3 |
| 240 | O=C(O)c1c(C2CCC(OC)CC2)n2c(SC=3C(C)(C)CC(C)(C)CC2=3)n1 |
| 241 | O=C(NCc1c(C(=O)O)nc2SC3=C(n12)CC[C@@H](C(C)(C)C)C3)C |
| 242 | O=C(O)c1c(CO)n2c(SC3=C2CC[C@@H](C(C)(C)C)C3)n1 |
| 243 | O=C(O)c1c(CN(OC)C)n2c(SC3=C2CC[C@@H](C(C)(C)C)C3)n1 |
| 244 | O=C(O)c1c(C2CC2)n2c(SC=3C(C)(C)CC(C)(C)CC2=3)n1 |
| 245 | Clc1c(Cl)c2Sc3n(-c2cc1OCCN1CCOCC1)cc(C(=O)O)n3 |
| 246 | Clc1c(F)ccc2N3C(Sc12)=N[C@](C(=O)O)(Cc1ccccc1)C3 |
| 247 | Clc1c(F)ccc2N3C(Sc12)=N[C@@](C(=O)O)(CO)C3 |
| 248 | Clc1c(N2CC(F)(F)C2)ccc2-n3c(Sc12)nc(C(=O)O)c3 |
| 249 | O=C(O)c1c(N2CCN(CCOC)CC2)n2c(SC3=C2CC[C@@H](C(C)(C)C)C3)n1 |
| 250 | O=C(O)c1c(CN2CCN(C)CC2)n2c(SC3=C2CC[C@@H](C(C)(C)C)C3)n1 |
| 251 | O=C(O)c1c(CN2CCN(CC)CC2)n2c(SC3=C2CC[C@@H](C(C)(C)C)C3)n1 |
| 252 | Clc1c(F)ccc2-n3c(N4CCN(C)CC4)c(C(=O)O)nc3Sc12 |
| 253 | Clc1c(F)ccc2-n3c(NCCN4CCCC4)c(C(=O)O)nc3Sc12 |
| 254 | Clc1c(F)ccc2N3C(Sc12)=N[C@@](C(=O)O)(C)C3 |
| 255 | O=C(O)c1c(N(C)C)n2c(SC=3C(C)(C)CC(C)(C)CC2=3)n1 |
| 256 | Clc1c(F)ccc2-n3c(c(C(=O)O)nc3Sc12)C1CNC1 |
| 257 | O=C(C)N1CCN(c2c(C(=O)O)nc3SC=4C(C)(C)CC(C)(C)CC=4n23)CC1 |
| 258 | Clc1c(Cl)c2SC3=N[C@H](C(=O)O)CN3c2cc1OCCN1CCOCC1 |
| 259 | O=C(O)c1c(C(=C)C)n2c(SC=3C(C)(C)CC(C)(C)CC2=3)n1 |
| 260 | O=C(O)c1c(N2CCN(C)CC2)n2[C@@H]3[C@H](C(C)(C)CC(C)(C)C3)Sc2n1 |
| 261 | Clc1c(F)c2Sc3n(-c2cc1OCCN1CCOCC1)cc(C(=O)O)n3 |
| 262 | Clc1c(F)ccc2-n3c(c(C(=O)O)nc3Sc12)C1CN(C)C1 |
| 263 | Clc1c(F)ccc2-n3c(c(C(=O)O)nc3Sc12)C1CC1 |
| 264 | S(C)c1c(C(=O)O)nc2SC=3C(C)(C)CC(C)(C)CC=3n12 |
| 265 | Clc1c(F)ccc2-n3c(c(C(=O)O)nc3Sc12)C1CN(S(=O)(=O)C)C1 |
| 266 | Clc1c(F)ccc2N3C(Sc12)=N[C@](C(=O)NS(=O)(=O)C)(C)C3 |
| 267 | O=c1oc2ccc(C)c(c2c3c14)c(=O)oc3c5c(c4O)cccc5O[C@@H]6[C@H]([C@H](O)[C@@H](O)[C@@H](O6)C)O[C@@H]7[C@@H](O)[C@H](OC)[C@@H](O)[C@@H](O7)C |
| 268 | N12[C@H]3NS(=O)(=O)N[C@H]4N(S1(=O)=O)[C@H](CC4)NS(=O)(=O)N[C@@H]2CC3 |
| 269 | OC\C=C(C)\CCC=C(C)C |
| 270 | C1CCCCCCCCCc(c12)c(nc(=S)[nH]2)-c3nc(=S)[nH]c(c34)CCCCCCCCCC4 |
| 271 | c1cccc(c12)c3c(cccc3)n2Cn4c(cccc5)c5c(c46)cccc6 |
| 272 | C1CCCCCC[N+]1(C)CCCC[N+]2(C)CCCCCCC2 |
| 273 | Clc1c(Cl)c(Cl)c(O)c(Cl)c1Cl |
| 274 | Clc1c(Cl)c(Cl)c(Cl)c(Cl)c1CSc2nc(O)nc(n2)[S-] |
| 275 | c1cc(Cl)ccc1-c(c2Cl)c(Cl)nc(n2)/C=C/c3ccc(Cl)cc3 |
| 276 | C1CN(C)CCN1c(cc(n2)C)n(c23)nc(n3)-c4ccc(Cl)cc4 |
| 277 | [O-]c1c(OC)cc(c2c13)CC[N+](C)(C)[C@@H]2Cc4cc(c(O)cc4)Oc5cc6c(cc5OC)CC[N+](C)(C)[C@@H]6Cc7ccc(O3)cc7 |
| 278 | c1cc(O)c(O)cc1C[C@H]2[N+](C)(C)CCc(c23)cc(O)c([O-])c3 |
| 279 | CNC[C@@H](SC)c1cc(O)c(O)cc1 |
| 280 | Oc1cc(O)cc(c12)O[C@H](c3cc(O)c(O)cc3)[C@@H](O)[C@@H]2c4c(O)cc(O)c(c45)C[C@@H](O)[C@@H](O5)c6cc(O)c(O)cc6 |
| 281 | CC(C)=CCc(c1O)c(OC(C)(C)C=C2)c2c(c13)occ(c3=O)-c4cc(O)c(O)cc4 |
| 282 | O=C(O)CCc(c1OC)c(OC(C)(C)C=C2)c2c(c13)OC(C)(C)C=C3 |
| 283 | c1cc(O)cc(CC2)c1[C@H](CC3)[C@H]2[C@H]([C@@]34C)CC[C@H]4OC(=O)CCc5ccccc5 |
| 284 | c1cc(O)cc(CC2)c1[C@H](CC3)[C@H]2[C@H]([C@@]34C)CC[C@H]4OC(=O)CCC5CCCC5 |
| 285 | O=C1CC[C@@H]([C@]12C)[C@@H]3[C@@H](CC2)c4c(CC3)cc(cc4)OP(=O)([O-])[O-] |
| 286 | O=C1/C(=N/O)C[C@@H]([C@]12C)[C@@H]3[C@@H](CC2)c4c(CC3)cc(cc4)OCC(C)=C |
| 287 | c1cc(C(C)C)cc(c1[C@@]23C)CC[C@@H]2[C@](C)(CN)CCC3 |
| 288 | [O-]c1c[n+](no1)-c2ccc(C)cc2 |
| 289 | n1oc([O-])c[n+]1CCCC[n+]2cc([O-])on2 |
| 290 | C1CCCCN1CC[n+](cc2)cc(c3C)c2c(C)c(c34)[nH]c5c4cc(O)cc5 |
| 291 | c1cccc(s2)c1c(=O)c(c23)c(ccc3CO)NCCN4CCCCC4 |
| 292 | o1c(=O)c(C(=O)O)cc(c12)cc(cc2OC)CN3CCCCC3 |
| 293 | C1CCCCN1Cc2c(O)c(cc(c2)C)CN3CCCCC3 |
| 294 | CC(C)(C)CC(C)(C)c1cc(c(O)c(c1)O)CN2CCCCC2 |
| 295 | c1cccc(O)c1CNCc2c(O)cccc2 |
| 296 | C[N+]([O-])(C)Cc1cc(ccc1O)-c2ccccc2 |
| 297 | CC(C)(C)CC(C)(C)c1cc(C[N+]([O-])(C)C)c(O)cc1 |
| 298 | c1ccccc1C(O)(c2c(CN(C)C)cc(C(F)(F)F)cc2)c3ccccc3 |
| 299 | Oc1ccc(C(C)(C)C)cc1CN(c2ccccc2)Cc3cc(C(C)(C)C)ccc3O |
| 300 | CN(C)Cc1c(cc(cc1)C(C)(C)C)-c2c(CN(C)C)ccc(c2)C(C)(C)C |
| 301 | CN(C)Cc1c(O)c(CN(C)C)cc(c1)C(C)(C)C |
| 302 | Oc1c(Br)cc(C(C)(C)C)cc1CN(C)Cc2cc(C(C)(C)C)cc(Br)c2O |
| 303 | Oc1c(C)cc(C(C)(C)C)cc1CNC2CCCCC2 |
| 304 | c1c(C)c(N)cc(N)c1Cc2c(N)cc(N)c(c2)C |
| 305 | c1c(C)cc(C)c(O)c1Cc2c(O)c(ccc2)Cc3c(O)c(C)cc(c3)C |
| 306 | c1c(C)cc(C(C)(C)C)c(O)c1Cc2c(C)c(C)c(c(C)c2C)Cc3c(O)c(C(C)(C)C)cc(c3)C |
| 307 | Cc1c(Cl)cc(C(C)(C)C)c(O)c1Cc2c(O)c(C(C)(C)C)cc(Cl)c2C |
| 308 | CC(C)(C)c1c(O)ccc(c1)O |
| 309 | Oc1c(C)ccc(c1C)C2(CCN(C)CC2)c3c(C)c(O)c(C)cc3 |
| 310 | OCC(C)(CO)\N=C\c1c(O)c(I)cc(I)c1 |
| 311 | NCc1c(CO)cnc(C)c1O |
| 312 | CN(C)Cc1c(CN(C)C)c(C)[nH]c1C |
| 313 | c1c(Br)c(O)c(Br)cc1CN(C2CCCCC2)Cc3cc(Br)c(O)c(Br)c3 |
| 314 | C1CCCCC1N(CO2)Cc(c3C(=O)O)c2c(O)c(c34)OCN(C4)C5CCCCC5 |
| 315 | C1CCCCC1N(C2CCCCC2)Cc3c(O)c(C(=O)C)ccc3O |
| 316 | O=C(O)c1c(C(=O)O)c(O)cc(c12)C(=O)c3c(C2=O)c(O)c(C(=O)C)c(c3O)CC |
| 317 | c1cccc(c12)CC[N@+](C)(C2)CCN[N+]3(C)CCCCC3 |
| 318 | C[N+](C)(C)CCN[N@+]1(C)CCCc(c12)cccc2 |
| 319 | C1CC[C@@H](C)CN1C[C@@H](O)CN2CCCc(c23)cccc3 |
| 320 | c1cccc(c12)CCN[C@@H]2CC(C3)=C(CC)CN4CCc(c5[C@H]34)cc(OC)c(c5)OC |
| 321 | CN(C)CCC=C1c(cccc2)c2C=Cc(c13)cccc3 |
| 322 | CN(C)CC/C=C\1c(cccc2)c2Sc(c13)ccc(Cl)c3 |
| 323 | C1CCCCC12N(CCC2)CCCN3c(cccc4)c4Sc(c35)ccc(Cl)c5 |
| 324 | C1CN(C)CCN1CCCN2c(cccc3)c3Sc(c24)ccc(c4)C(F)(F)F |
| 325 | C1CCCN(C)[C@H]1CCN2c(cccc3)c3Sc(c24)ccc(c4)OC |
| 326 | C1CCCN(C)[C@H]1CCN2c(cccc3)c3Sc(c24)ccc(c4)[S@](=O)C |
| 327 | CCN(CC)CCN1c(cccc2)c2Sc3ccc(c4c13)cccc4 |
| 328 | C1CC(C)CCN1CC(=O)N2c(cccc3)c3Sc(c24)cc5c(c4)cccc5 |
| 329 | CN(C)C[C@@H]1C(=O)[C@H](CN(C)C)CCC1 |
| 330 | CN(C)C[C@H](C1=O)CCC(=C12)OC3=C(C(=O)[C@H](CC3)CN(C)C)C2c4cc(Cl)c(Cl)cc4 |
| 331 | c1cc(N(C)C)ccc1[C@@H](C#N)N2C(c3ccc(cc3)N(C)C)N(CCC2)[C@@H](C#N)c4ccc(cc4)N(C)C |
| 332 | C1CCCN(C)[C@H]1CCN2CCCCC2 |
| 333 | CC(C)(C)C(CC1)CCC12CCN(CC2)CCCN(C)C |
| 334 | CC(C)(C)C(CC1)CCC12CCN(CC2)CCCN3CCOCC3 |
| 335 | C1COCCN1CN(C2=O)C(=O)[C@H]3[C@H]([C@@H]4[C@@H]23)[C@@H]5[C@H]6[C@H]([C@H]4C=C5)C(=O)N(C6=O)CN7CCOCC7 |
| 336 | C1COCCN1Cn2nnc(c23)cccc3 |
| 337 | c1cc(OC)ccc1C[C@H](O)CN2CCOCC2 |
| 338 | C1COCCN1Cc2c(O)c(O)c(cc2)CN3CCOCC3 |
| 339 | c1c(Cl)ccc(Cl)c1SCCCN2CCNCC2 |
| 340 | CC(C)(C)c1cc(C)cc(C(C)(C)C)c1OCCCN2CCOCC2 |
| 341 | [S-]C(=S)NCC[NH+]1CCNCC1 |
| 342 | CC[NH+](CC)COC(=S)[S-] |
| 343 | C1CC1CN(CN2)CN=C2SCc3cc(Cl)cc(c34)COCO4 |
| 344 | O1COc(c12)cc(O)c(c2)[C@H](Nc3ncccn3)c4ccc(cc4)OC |
| 345 | C1CNCCN1C(c(c2)ccc(c23)OCO3)N4CCNCC4 |
| 346 | O1COc(c12)ccc(c2)/C=N/[C@@H]3[C@@H](O)[C@H](O)[C@@H](CO)O[C@H]3O |
| 347 | O[C@@H]1[C@@H](O)[C@H](CO)O[C@@H]([C@H]1O)N2CCN(CC2)[C@@H]3O[C@H](CO)[C@@H](O)[C@H](O)[C@H]3O |
| 348 | NC(=O)OCc1c(C(=O)C(=C(C2=O)C)OC)c2n(c13)C[C@@H]4[C@H]3N4C |
| 349 | O=C(O)[C@@H]1[C@H](C(=O)O)[C@@H](C2)[C@@H]([C@H](Br)[C@H]12)N3C(=O)CCC3=O |
| 350 | O=C(O)[C@@]12C(=O)[C@]3(C(=O)O)C[C@@](C2)(C(=O)O)C(=O)[C@](C1)(C3)C(=O)O |
| 351 | O=C(O)[C@](Br)(C)[C@H](Br)C(=O)O |
| 352 | O=C(O)C(F)(F)C(F)(F)C(F)(F)C(F)(F)C(=O)O |
| 353 | Cl[C@@]12[C@H](C(=O)O)[C@H](C(=O)O)[C@@](Cl)(C1(Cl)Cl)C(Cl)=C2Cl |
| 354 | O=C(O)C[C@@]12C(C)(C)[C@@H](C[C@@H]1Cl)CC2 |
| 355 | O=C(O)CC1(CC(=O)O)C[C@H](C)CCC1 |
| 356 | O=C(O)CN(CC)CC(=O)O |
| 357 | O=C(O)CN(C(C)C)CC(=O)O |
| 358 | NC(=O)N[C@@H](C(=O)O)CC(=O)O |
| 359 | O=C(O)CC(C(=O)O)CC(=O)O |
| 360 | NC(=O)C[S@@](=O)C[C@H](N)C(=O)O |
| 361 | O=P([O-])([O-])CC[C@@H](C(=O)[O-])[NH3+] |
| 362 | O=C(O)[C@H]([C@@H]12)[C@@H](N)[C@H](C1)C=C2 |
| 363 | NCC(=O)[C@@H](O)[C@H](O)[C@H](O)CO |
| 364 | O=C(O)[C@@H](O)[C@@H](O)[C@H](O)[C@H](O)CO |
| 365 | OC[C@@H](O)[C@@H](O)[C@@H](O)[C@H]1[C@@H](O)[C@@H](C(O1)=O)O |
| 366 | NC(=[NH2+])SCC[C@@]1(CC)C(=O)[N-]C(=O)NC1=O |
| 367 | CN(C)CS/C(N)=N/[C@@H](C(Cl)(Cl)Cl)O |
| 368 | O[C@@H]1[C@@H](O)[C@H](O)[C@@H](O)[C@H](O)[C@H]1O[C@@H]2[C@@H]([NH3+])C[C@H]([C@@H](O2)C)/N=C(/C([O-])=O)N |
| 369 | O=C(O)CC[C@@H](C)[C@@H]1CC[C@@H]([C@]12C)[C@@H]3[C@@H](C[C@@H]2O)[C@]4(C)[C@@H](CC3)C[C@@H](O)CC4 |
| 370 | C1C[C@H](C2(C)C)C[C@H]([C@@]12C)OC(=O)CSC#N |
| 371 | C1C[C@H](C2(C)C)C[C@H]([C@@]12C)OCC([N+](=O)[O-])(C)C |
| 372 | O=C(O)[C@H]1[C@@H](C(=O)O)[C@@](C=C2)(C[C@H](O)CC3)[C@]3(C)C([C@@]124)=CC[C@@]5(C)[C@H]4CC[C@@H]5C(=O)C |
| 373 | O=P([O-])([O-])OCC(=O)[C@]1(O)[C@@H](C)C[C@@H]([C@]12C)[C@H]3[C@](F)([C@@H](C2)O)[C@@]4(C)C(CC3)=CC(=O)C=C4 |
| 374 | C1CC(=O)C=C(CC2)[C@]1(C)[C@H]([C@H](C3)O)[C@H]2[C@H]([C@@]34C)CC[C@@]4(O)C(=O)COC(=O)C5CCCC5 |
| 375 | C1CC(=O)C=C(CC2)[C@]1(C)[C@H]([C@H](C3)O)[C@H]2[C@H]([C@@]34C)CC[C@@]4(O)C(=O)COC(=O)[C@@H]5CN(C)CCC5 |
| 376 | C1OC(=O)C=C1[C@@H](CC2)[C@@](C)(C[C@@H]3O)[C@]2(O)[C@H]([C@@H]3[C@]45CO)CC[C@@]4(O)C[C@H](C[C@H]5O)O[C@@H]6[C@@H](O)[C@H](O)[C@@H](O)[C@@H](O6)C |
| 377 | C1C[C@@H](O)[C@@](C)(CO)[C@H]([C@@]1([C@@]234)C)CC[C@H]2C[C@@H](C3)[C@@](O)(CC4)COC(=O)CN |
| 378 | O=C1OCC=C1[C@@H](CC2)[C@@]3(C)CC[C@@H]([C@@H]4[C@]23O)[C@]5(C)[C@@H](CC4)C[C@H](CC5)O[C@@H]6O[C@H](CO)[C@@H](O)[C@H](O)[C@H]6O |
| 379 | C1C[C@H](O)C[C@H](CC2)[C@@]1(C)[C@H](CC3)[C@H]2[C@](O)([C@@]34C)C[C@@H](O)[C@H]4C5=CC(=O)OC5 |
| 380 | FC(F)(F)[C@@H](O)CC(=O)[C@@H]1CC[C@@H]([C@]12C)[C@@H]3[C@@H](CC2)[C@]4(C)[C@@H](CC3)C[C@@H](O)CC4 |
| 381 | C1C(C)=CC(=O)[C@H]2[C@H]1C[C@@H]([C@]23C)[C@@H]4[C@@H](CC3=O)[C@]5(C)[C@@H](CC4)C[C@H](CC5)OC(=O)C |
| 382 | CC(=O)OC(CC1)=CC([C@]1(C)[C@]23S)=CC[C@H]2[C@H]4[C@](C)(CC3=O)C(=O)CC4 |
| 383 | CC(=O)[C@]1(O)CC[C@@H]([C@]12C)[C@@H]3[C@@H]([C@H](C2)O)[C@@]4(C)C([C@@H](C3)C)=CC(=O)CC4 |
| 384 | OCC(=O)[C@](O)(CC1)[C@](C)([C@@]12O)CC[C@@H]3[C@@H]2CCC=4[C@@]3(C)CCC(=O)C4 |
| 385 | O=C(O)CC(=CC1)[C@@](C)(CC2=O)[C@@H]1[C@H]([C@@H]2[C@]34C)CCC3=CC(=O)CC4 |
| 386 | C1CC(=O)C=C(CC2)[C@]1(C)[C@H]([C@H](C3)O)[C@H]2[C@H]([C@@]34C)CC[C@H]4c5nc(N)sc5 |
| 387 | C1CC(=O)C=C(CC2)[C@]1(C)[C@H](CC3)[C@H]2[C@H]([C@@]34C)CC[C@H]4C(=O)COS(=O)(=O)c5ccc(Br)cc5 |
| 388 | C1CC(=O)C=C(CC2)[C@]1(C)[C@H](CC3)[C@H]2[C@H]([C@@]34C)CC[C@H]4C(=O)Cn5cnc(c56)c(=S)[nH]cn6 |
| 389 | C1CC(=O)C=C(CC2)[C@]1(C)[C@H](CC3)[C@H]2[C@H]([C@@]34C)CC[C@H]4C(=O)Cn5cnc(c56)c(SC)ncn6 |
| 390 | C1CC(=O)C=C(CC2)[C@@H]1[C@H](CC3)[C@H]2[C@H]([C@@]34C)CC[C@H]4OC(O)(C5=O)C(=O)c(c56)cccc6 |
| 391 | CC(=O)O[C@@H](CC1)[C@@](C)(CC2)[C@@H]1[C@H]([C@@H]2[C@]34C)CCC3=C[C@H]([C@@H](C4)OC(=O)C)C5SCCS5 |
| 392 | N1C[C@@H](C)CC[C@]12[C@H](C)[C@H]3[C@]4(C)[C@@H](C[C@H]3O2)[C@@H]5[C@@H](CC4)[C@]6(C)C(=CC5)C[C@H](CC6)OC(=O)C |
| 393 | O=C(O)[C@@H](C)[C@@H]1CC[C@@H]([C@]12C)[C@@H]3[C@@H](CC2)[C@]4(C)C(=CC3)C[C@H](CC4)OC(=O)C |
| 394 | CC(=O)O[C@@H](CC1)CC(=CC2)[C@@]1(C)[C@H](CC3)[C@H]2[C@H]([C@@]34C)CC[C@H]4c5cc(C(F)(F)F)n(n5)C(=O)C |
| 395 | C1C[C@H](CC)[C@@](C)(CC2)[C@@H]1[C@H]([C@@H]2[C@@]34C)CC=C3C[C@@H]([C@H](C4)N)OC(=O)C |
| 396 | OCCN(CCO)[C@@H]1CC[C@@H]([C@]12C)[C@@H]3[C@@H](CC2)[C@]4(C)C(=CC3)C[C@@H](O)CC4 |
| 397 | CN(C)CCC(=O)[C@]1(O)CC[C@@H]([C@]12C)[C@@H]3[C@@H](CC2)[C@]4(C)C(=CC3)C[C@@H](O)CC4 |
| 398 | C1C[C@](C)(O)[C@@](C)(CC2)[C@@H]1[C@H]([C@@H]2[C@@]34C)CC=C3N5C(CC4)=NCC5 |
| 399 | O=C(O)C(=O)[C@@]12[C@@H]3N(CC2)CC=4[C@@H](C3)[C@@H]([C@H]1N)[C@H](OCC4)CC(=O)O |
| 400 | O=C(O)C[C@@H]1OCC=C(CN([C@H]2C3)CC4)[C@H]3[C@@H]1[C@@H]([C@]245)Nc6c5cc(OC)c(c6)OC |
| 401 | COc(c1)c(OC)cc2c1N([C@@H]3[C@]245)C(=O)C[C@H]6[C@@H]3[C@@H]7C(CCO6)=CN([C@H]4C7)CC5 |
| 402 | CC1=CCCC(C)(C)[C@]12c3c4c(C2)c5c(c(O)c4c(O)cc3OC)C(=O)[C@]6(O)[C@@](O)([C@H]5O)CC(O)=C(C6=O)C(=O)N |

**Table S2.** The list of selected features through each feature selector (BF, GS, PSOS, SSFS) with each IC50 threshold (SET01~SET05).

| BF | SET01 | ATSC4v,AATSC8i,AATSC2s,GATS5s,VR1_Dze,BCUTw-1h,SpMax1_Bhm,nHBint5,nHdsCH,SHBint2, minHBa,minHBint2,  mindO,maxHBint2,gmin,ETA_dEpsilon_D,ZMIC3,nAtomP,MDEO-22,MLFER_A,piPC3, R_TpiPCTPC,nT6Ring,n4HeteroRing,  JGI9,WTPT-4,WTPT-5,MACCSFP95,MACCSFP106,MACCSFP148,PubchemFP376,PubchemFP418,PubchemFP461,  PubchemFP583,PubchemFP609,PubchemFP730,SubFP288 |
| --- | --- | --- |
|  | **SET02** | AATS5p,AATS7p,ATSC4v,ATSC8p,ATSC8i,AATSC2s,GATS1c,GATS5p,BCUTw-1h,BCUTp-1h,nBondsD,SpMax1_Bhm,  SpMax1_Bhp,SpMAD_Dt,nHBint5,nHdsCH,naasC,SHBint2,SHCsatu,minHBd,minHBa,minHBint2,minHBint5,mindO,  maxHBint2,maxdO,gmin,ETA_dEpsilon_D,MIC0,nAtomP,MDEO-22,MLFER_A,R_TpiPCTPC,JGI6,VR2_D,TopoPSA,  WTPT-5,MACCSFP72,MACCSFP95,MACCSFP106,MACCSFP148,PubchemFP376,PubchemFP378,PubchemFP418,  PubchemFP449,PubchemFP461,PubchemFP494,PubchemFP747,PubchemFP768,SubFP62 |
|  | **SET03** | AATS7p,AATS8s,ATSC4v,ATSC8v,ATSC8i,AATSC1m,AATSC8i,AATSC2s,MATS7c,MATS1s,GATS1c,GATS3c,GATS1e,  GATS6e,BCUTw-1h,BCUTp-1h,SpMax1_Bhm,SpMax6_Bhm,SpMax1_Bhp,SpMax2_Bhp,SCH-5,CrippenLogP,VE3_Dt,  nHBint5,nHdsCH,nHCsatu,naasC,SHBd,SwHBa,SHCsatu,SHother,minHBd,minHBint2,minHBint5,maxHBa,maxHBint2,maxdO,  gmin,MIC0,nAtomP,MDEO-22,MLFER_A,MLFER_BH,piPC8,R_TpiPCTPC,JGI2,JGI5,JGI9,TopoPSA,WTPT-5,  MACCSFP49,MACCSFP72,MACCSFP95,MACCSFP106,MACCSFP111,MACCSFP148,PubchemFP192,PubchemFP353,  PubchemFP376,PubchemFP418,PubchemFP438,PubchemFP461,PubchemFP494,PubchemFP566,PubchemFP768,PubchemFP818 |
|  | **SET04** | AATS3e,AATS5i,AATS7i,AATS3s,ATSC3c,ATSC8c,ATSC8v,ATSC3p,ATSC8p,ATSC6i,AATSC1m,AATSC2m,AATSC1v,  AATSC3v,AATSC8p,AATSC3i,AATSC8i,MATS7c,MATS4e,MATS1s,GATS2c,GATS5p,GATS8i,GATS2s,GATS4s,VE3_DzZ,  VR2_DzZ,VE1_Dzi,VE3_Dzi,BCUTp-1h,SpMax1_Bhm,SpMin2_Bhm,SCH-7,SPC-5,AVP-0,SHBint7,SHBint9,SHCsatu,  SaasC,minHBd,minHBint2,minHBint6,minsssCH,minsOH,minssO,maxHBa,maxHBint2,maxaaaC,meanI,ETA_AlphaP,  ETA_dBeta,MIC0,ZMIC1,ZMIC3,MLFER_BH,piPC8,R_TpiPCTPC,n6HeteroRing,GGI2,JGI2,JGI6,JGI7,WTPT-5,  MACCSFP77,MACCSFP97,MACCSFP111,PubchemFP450,PubchemFP768,PubchemFP831,SubFP150 |
|  | **SET05** | ALogP,AATS8s,ATSC7c,ATSC1v,AATSC4m,AATSC5m,AATSC8i,AATSC2s,MATS5c,MATS8s,GATS2c,VE3_Dzi,  SpMax1_Bhm,SpMin4_Bhm,SpMin1_Bhe,ASP-0,nHBint5,SHBint2,SsOH,minHBa,minHBint2,maxwHBa,maxHBint2,  maxaaN,maxdO,hmin,gmin,ETA_dEpsilon_D,IC1,IC2,ZMIC4,nAtomP,MDEC-22,MDEO-12,MLFER_BH,piPC3,R_TpiPCTPC,  nTRing,JGI2,JGI6,TopoPSA,MACCSFP95,MACCSFP97,MACCSFP106,MACCSFP121,MACCSFP148,PubchemFP192,  PubchemFP418,PubchemFP449,PubchemFP499,PubchemFP699 |
| GS | **SET01** | nAcid,ALogP,ALogp2,AMR,naAromAtom,nHeavyAtom,nH,nC,nN,nS,nCl,ATS1m,ATS3m,ATS5m,ATS6m,ATS7m,ATS6v,  ATS8v,ATS3e,ATS4e,ATS5e,ATS7e,ATS8e,ATS5p,ATS0s,ATS2s,ATS4s,ATS5s,ATS7s,AATS0m,AATS1m,AATS2m,AATS3m,  AATS4m,AATS5m,AATS6m,AATS0v,AATS3v,AATS5v,AATS6v,AATS8v,AATS0e,AATS1e,AATS2e,AATS3e,nP,nI,ATS4v,  AATS4e,AATS5e,AATS7e,AATS8e,AATS0p,AATS1p,AATS4p,AATS7p,AATS8p,AATS3i,AATS4i,AATS6i,AATS8i,AATS0s,  AATS2s,AATS3s,AATS4s,AATS5s,AATS8s,ATSC1c,ATSC2c,ATSC3c,ATSC4c,ATSC5c,ATSC6c,ATSC7c,ATSC8c,ATSC0m,  ATSC2m,ATSC3m,ATSC5m,ATSC6m,ATSC3v,ATSC6v,ATSC8v,ATSC1e,ATSC2e,ATSC5e,ATSC0p,ATSC2p,ATSC8p,ATSC0i,  ATSC2i,ATSC4i,ATSC0s,ATSC1s,ATSC2s,ATSC4s,ATSC6s,ATSC7s,AATSC0c,AATSC2c,AATSC4c,AATSC5c,AATSC8c,  AATSC0m,AATSC1m,AATSC3m,AATSC4m,AATSC5m,AATSC8m,AATSC0v,AATSC1v,AATSC2v,AATSC4v,AATSC5v,  AATSC6v,AATSC7v,AATSC8v,AATSC0e,AATSC1e,AATSC5e,AATSC6e,AATSC8e,AATSC3p,AATSC4p,AATSC5p,AATSC0i,  AATSC4i,AATSC7i,AATSC2s,AATSC6s,MATS2c,MATS6c,MATS3m,MATS4m,MATS6m,MATS4e,MATS6e,MATS7e,MATS8e,  MATS2p,MATS1i,MATS3i,MATS5i,MATS2s,MATS5s,MATS6s,MATS8s,GATS2c,GATS3c,GATS5c,GATS6c,GATS8c,GATS1m,  GATS2m,GATS4m,GATS5m,GATS6m,GATS8m,GATS1v,GATS3v,GATS4v,GATS5v,GATS6v,GATS8v,GATS2e,GATS4e,  GATS6e,GATS7e,GATS8e,GATS1p,GATS2p,GATS4p,GATS8p,GATS2i,GATS5i,GATS1s,GATS2s,GATS3s,GATS6s,GATS7s,  SpMAD_DzZ,SM1_DzZ,VR2_DzZ,SpDiam_Dzv,SM1_Dzv,VE1_Dzv,VE3_Dzv,VR2_Dzv,VR3_Dzv,SpMAD_Dze,VE1_Dze,  VE2_Dze,VE3_Dze,VR2_Dze,VE1_Dzp,VE2_Dzp,VR1_Dzp,VR3_Dzp,VE1_Dzi,VE3_Dzi,VR1_Dzi,VR2_Dzi,SpAbs_Dzs,  SpMAD_Dzs,VE1_Dzs,VE2_Dzs,VR1_Dzs,VR2_Dzs,VR3_Dzs,nBase,BCUTw-1l,BCUTw-1h,BCUTc-1h,BCUTp-1l,nBondsS2,  nBondsS3,nBondsD,nBondsT,SpMax2_Bhm,SpMax4_Bhm,SpMax5_Bhm,SpMin2_Bhm,SpMin3_Bhm,SpMin4_Bhm,  SpMin5_Bhm,SpMin6_Bhm,SpMin7_Bhm,SpMax2_Bhv,SpMax4_Bhv,SpMax5_Bhv,SpMax6_Bhv,SpMax7_Bhv,SpMin1_Bhv,  SpMin2_Bhv,SpMin3_Bhv,SpMin4_Bhv,SpMin5_Bhv,SpMin6_Bhv,SpMax2_Bhe,SpMax5_Bhe,SpMax6_Bhe,SpMax7_Bhe,  SpMax8_Bhe,SpMin1_Bhe,SpMin2_Bhe,SpMin3_Bhe,SpMin4_Bhe,SpMax1_Bhp,SpMax2_Bhp,SpMin3_Bhp,SpMin7_Bhp,  SpMin8_Bhp,SpMax2_Bhi,SpMax3_Bhi,SpMax7_Bhi,SpMin1_Bhi,SpMin2_Bhi,SpMin3_Bhi,SpMax1_Bhs,SpMax5_Bhs,  SpMax6_Bhs,SpMax8_Bhs,SpMin6_Bhs,SpMin8_Bhs,C1SP1,C2SP1,C2SP2,C1SP3,C2SP3,C3SP3,SCH-4,SCH-5,SCH-6,  SCH-7,VCH-3,VCH-6,SC-4,SC-5,VC-5,VC-6,SPC-5,VPC-4,VPC-5,ASP-1,ASP-2,ASP-6,ASP-7,VP-0,VP-3,VP-4,VP-5,  AVP-0,AVP-2,AVP-6,AVP-7,Sv,CrippenLogP,SpMax_Dt,SpMAD_Dt,VE1_Dt,VE3_Dt,VR1_Dt,VR3_Dt,ECCEN,nHBd,nHBa,  nwHBa,nHBint2,nHBint4,nHBint5,nHBint6,nHBint7,nHBint8,nHsOH,nHsSH,nHsNH2,nHsssNHp,nHdsCH,nHaaCH,nHCHnX,  nHCsatu,nHother,nssCH2,ndsCH,naaCH,nsssCH,ndssC,naaaC,nsNH3p,nssNH,nsssNHp,naasN,nssssNp,nssO,naaO,nsOm,ndsssP,  nsSH,naaS,nsCl,nsI,SHBd,SHBa,SHBint2,SHBint3,SHBint6,SHBint7,SHBint8,SHBint9,SHBint10,SHsOH,SHsNH2,SHssNH,  SHaaNH,SHsssNHp,SHtCH,SHdCH2,SHAvin,StCH,SsssCH,SdssC,SaasC,SssssC,SsNH3p,SsNH2,SssNH,StN,SdsN,SsssN,  SaasN,SssssNp,SsOH,SdO,SaaO,SsSH,SdS,SaaS,SdssS,SsCl,minHBd,minwHBd,minHBa,minwHBa,minHBint2,minHBint3,  minHBint5,minHBint6,minHsOH,minHsNH2,minHssNH,minHaaNH,minHsssNHp,minHtCH,minHdCH2,minHdsCH,minHAvin,  minsCH3,mindCH2,minssCH2,mintCH,mindsCH,mintsC,mindssC,minssssC,minsNH3p,minsNH2,minssNH,minaaNH,minaaN,  minaasN,minssssNp,minsOH,mindO,minssO,minaaO,minsF,mindsssP,mindS,minssS,minaaS,minddssS,minsCl,maxHBd,  maxwHBd,maxwHBa,maxHBint2,maxHBint3,maxHBint4,maxHBint5,maxHBint6,maxHBint7,maxHBint8,maxHBint9,  maxHsSH,maxHsNH2,maxHssNH,maxHaaNH,maxHsNH3p,maxHdCH2,maxHdsCH,maxHaaCH,maxHCsats,maxHCsatu,  maxsCH3,maxdCH2,maxtCH,maxaaCH,maxaasC,maxaaaC,maxsNH2,maxaaNH,maxtN,maxsssNHp,maxdsN,maxaaN,maxaasN,  maxsOH,maxdO,maxsF,maxdS,hmax,gmin,LipoaffinityIndex,MAXDN,DELS,ETA_Alpha,ETA_dAlpha_A,ETA_Epsilon_3,  ETA_Epsilon_4,ETA_dEpsilon_B,ETA_Psi_1,ETA_dPsi_B,ETA_Shape_Y,ETA_Beta_s,ETA_BetaP_s,ETA_Beta_ns,  ETA_BetaP_ns,ETA_dBetaP,ETA_Eta,ETA_Eta_F,ETA_EtaP_F,ETA_EtaP_L,ETA_EtaP_F_L,ETA_Eta_B,ETA_EtaP_B,FMF,  nHBAcc2,nHBAcc3,nHBAcc_Lipinski,IC2,TIC2,SIC1,SIC2,SIC3,CIC0,BIC2,BIC3,BIC4,BIC5,MIC0,MIC1,MIC3,ZMIC0,  ZMIC3,Kier1,Kier3,MDEC-11,MDEC-13,MDEC-14,MDEC-22,MDEC-24,MDEC-33,MDEC-44,MDEN-11,MDEN-12,  MDEN-13,MDEN-33,MLFER_A,MLFER_BH,MLFER_S,MLFER_L,piPC1,piPC2,piPC4,n3Ring,n4Ring,n6Ring,n7Ring,  n12Ring,nFRing,nF7Ring,nF11Ring,nF12Ring,nFG12Ring,nTRing,nT4Ring,nT5Ring,nT8Ring,nT10Ring,nTG12Ring,  nHeteroRing,n3HeteroRing,n4HeteroRing,n7HeteroRing,nF6HeteroRing,nF9HeteroRing,nF12HeteroRing,nT4HeteroRing,  nT7HeteroRing,nT8HeteroRing,nT9HeteroRing,nT11HeteroRing,nTG12HeteroRing,RotBFrac,nRotBt,LipinskiFailures,  topoDiameter,topoShape,GGI1,GGI3,GGI5,GGI6,GGI7,GGI8,GGI9,JGI2,JGI4,JGI5,JGI6,JGI9,SpMAD_D,VE1_D,VR1_D,  VR2_D,VR3_D,TopoPSA,MWC3,SRW7,SRW9,MW,WTPT-4,WTPT-5,MACCSFP8,MACCSFP11,MACCSFP16,MACCSFP17,  MACCSFP19,MACCSFP21,MACCSFP24,MACCSFP26,MACCSFP27,MACCSFP28,MACCSFP29,MACCSFP30,  MACCSFP32,MACCSFP39,MACCSFP40,MACCSFP42,MACCSFP43,MACCSFP45,MACCSFP46,MACCSFP49,  MACCSFP50,MACCSFP55,MACCSFP57,MACCSFP58,MACCSFP59,MACCSFP60,MACCSFP61,MACCSFP63,  MACCSFP65,MACCSFP68,MACCSFP69,MACCSFP70,MACCSFP73,MACCSFP74,MACCSFP75,MACCSFP77,  MACCSFP83,MACCSFP85,MACCSFP87,MACCSFP88,MACCSFP89,MACCSFP91,MACCSFP93,MACCSFP96,  MACCSFP97,MACCSFP98,MACCSFP99,MACCSFP100,MACCSFP102,MACCSFP104,MACCSFP106,MACCSFP108,  MACCSFP114,MACCSFP117,MACCSFP118,MACCSFP119,MACCSFP121,MACCSFP122,MACCSFP123,MACCSFP124,  MACCSFP126,MACCSFP129,MACCSFP130,MACCSFP132,MACCSFP133,MACCSFP135,MACCSFP136,MACCSFP137,  MACCSFP138,MACCSFP140,MACCSFP142,MACCSFP145,MACCSFP147,MACCSFP149,MACCSFP152,MACCSFP153,  MACCSFP154,MACCSFP155,MACCSFP156,MACCSFP159,MACCSFP160,MACCSFP162,MACCSFP163,MACCSFP165,  PubchemFP1,PubchemFP2,PubchemFP11,PubchemFP12,PubchemFP13,PubchemFP14,PubchemFP15,PubchemFP16,  PubchemFP19,PubchemFP20,PubchemFP23,PubchemFP24,PubchemFP25,PubchemFP30,PubchemFP34,PubchemFP37,  PubchemFP38,PubchemFP43,PubchemFP44,PubchemFP132,PubchemFP136,PubchemFP144,PubchemFP145,PubchemFP149,  PubchemFP150,PubchemFP152,PubchemFP153,PubchemFP183,PubchemFP185,PubchemFP186,PubchemFP188,PubchemFP190,  PubchemFP193,PubchemFP199,PubchemFP206,PubchemFP213,PubchemFP214,PubchemFP216,PubchemFP230,PubchemFP236,  PubchemFP255,PubchemFP256,PubchemFP257,PubchemFP258,PubchemFP285,PubchemFP287,PubchemFP293,PubchemFP300,  PubchemFP301,PubchemFP308,PubchemFP314,PubchemFP330,PubchemFP332,PubchemFP334,PubchemFP335,PubchemFP336,  PubchemFP337,PubchemFP338,PubchemFP339,PubchemFP341,PubchemFP346,PubchemFP347,PubchemFP349,PubchemFP351,  PubchemFP353,PubchemFP355,PubchemFP358,PubchemFP360,PubchemFP362,PubchemFP363,PubchemFP364,PubchemFP368,  PubchemFP370,PubchemFP371,PubchemFP373,PubchemFP376,PubchemFP377,PubchemFP378,PubchemFP379,PubchemFP380,  PubchemFP382,PubchemFP383,PubchemFP387,PubchemFP389,PubchemFP390,PubchemFP391,PubchemFP392,PubchemFP397,  PubchemFP398,PubchemFP404,PubchemFP405,PubchemFP411,PubchemFP414,PubchemFP416,PubchemFP418,PubchemFP420,  PubchemFP422,PubchemFP423,PubchemFP425,PubchemFP427,PubchemFP429,PubchemFP430,PubchemFP431,PubchemFP432,  PubchemFP435,PubchemFP437,PubchemFP438,PubchemFP440,PubchemFP441,PubchemFP442,PubchemFP445,PubchemFP446,  PubchemFP447,PubchemFP449,PubchemFP451,PubchemFP455,PubchemFP456,PubchemFP457,PubchemFP458,PubchemFP459,  PubchemFP460,PubchemFP461,PubchemFP462,PubchemFP473,PubchemFP477,PubchemFP479,PubchemFP480,PubchemFP481,  PubchemFP482,PubchemFP483,PubchemFP484,PubchemFP485,PubchemFP487,PubchemFP488,PubchemFP490,PubchemFP491,  PubchemFP493,PubchemFP495,PubchemFP497,PubchemFP498,PubchemFP500,PubchemFP501,PubchemFP504,PubchemFP505,  PubchemFP506,PubchemFP507,PubchemFP513,PubchemFP514,PubchemFP515,PubchemFP516,PubchemFP518,PubchemFP521,  PubchemFP522,PubchemFP523,PubchemFP526,PubchemFP528,PubchemFP532,PubchemFP533,PubchemFP534,PubchemFP544,  PubchemFP545,PubchemFP547,PubchemFP549,PubchemFP550,PubchemFP557,PubchemFP559,PubchemFP562,PubchemFP568,  PubchemFP571,PubchemFP573,PubchemFP577,PubchemFP580,PubchemFP581,PubchemFP582,PubchemFP588,PubchemFP592,  PubchemFP594,PubchemFP598,PubchemFP608,PubchemFP610,PubchemFP616,PubchemFP628,PubchemFP632,PubchemFP633,  PubchemFP634,PubchemFP635,PubchemFP639,PubchemFP642,PubchemFP643,PubchemFP644,PubchemFP645,PubchemFP650,  PubchemFP651,PubchemFP656,PubchemFP661,PubchemFP662,PubchemFP663,PubchemFP666,PubchemFP670,PubchemFP672,  PubchemFP679,PubchemFP682,PubchemFP687,PubchemFP688,PubchemFP694,PubchemFP698,PubchemFP700,PubchemFP704,  PubchemFP708,PubchemFP711,PubchemFP716,PubchemFP722,PubchemFP731,PubchemFP735,PubchemFP740,PubchemFP742,  PubchemFP743,PubchemFP748,PubchemFP750,PubchemFP753,PubchemFP755,PubchemFP765,PubchemFP771,PubchemFP772,  PubchemFP782,PubchemFP783,PubchemFP784,PubchemFP785,PubchemFP788,PubchemFP794,PubchemFP797,PubchemFP801,  PubchemFP803,PubchemFP804,PubchemFP810,PubchemFP834,PubchemFP836,PubchemFP839,PubchemFP840,PubchemFP842,  PubchemFP843,PubchemFP857,PubchemFP861,PubchemFP877,SubFP4,SubFP6,SubFP8,SubFP26,SubFP36,SubFP38,SubFP41,  SubFP49,SubFP52,SubFP63,SubFP68,SubFP72,SubFP74,SubFP96,SubFP133,SubFP169,SubFP170,SubFP172,SubFP180,  SubFP183,SubFP212,SubFP224,SubFP246,SubFP274,SubFP281,SubFP285,SubFP286,SubFP287,SubFP288,SubFP296 |
|  | **SET02** | nAcid,ALogp2,AMR,naAromAtom,nHeavyAtom,nH,nS,nCl,nBr,ATS0m,ATS1m,ATS5m,ATS6m,ATS6v,ATS8v,ATS3e,ATS4e,  ATS5e,ATS6e,ATS7e,ATS0p,ATS2p,ATS5p,ATS0s,ATS1s,ATS2s,ATS3s,ATS4s,ATS8s,AATS0m,AATS1m,AATS2m,AATS3m,  AATS5m,AATS6m,AATS8m,AATS0v,AATS3v,AATS6v,AATS0e,nP,ATS4v,AATS5e,AATS7e,AATS1p,AATS8p,AATS0i,  AATS2i,AATS4i,AATS6i,AATS7i,AATS1s,AATS3s,AATS5s,AATS6s,AATS7s,AATS8s,ATSC0c,ATSC1c,ATSC2c,ATSC3c,  ATSC5c,ATSC6c,ATSC8c,ATSC0m,ATSC3m,ATSC6m,ATSC0v,ATSC1v,ATSC2v,ATSC3v,ATSC5v,ATSC6v,ATSC7v,ATSC0e,  ATSC1e,ATSC5e,ATSC7e,ATSC1p,ATSC2p,ATSC3p,ATSC4p,ATSC8p,ATSC0i,ATSC2i,ATSC3i,ATSC4i,ATSC8i,ATSC1s,  ATSC2s,ATSC3s,ATSC4s,ATSC5s,ATSC6s,ATSC7s,AATSC0c,AATSC3c,AATSC6c,AATSC7c,AATSC8c,AATSC0m,AATSC1m,  AATSC2m,AATSC4m,AATSC7m,AATSC8m,AATSC0v,AATSC1v,AATSC3v,AATSC4v,AATSC7v,AATSC8v,AATSC0e,  AATSC1e,AATSC3e,AATSC7e,AATSC0p,AATSC1p,AATSC2p,AATSC3p,AATSC4p,AATSC8p,AATSC0i,AATSC1i,AATSC3i,  AATSC4i,AATSC2s,AATSC4s,AATSC7s,AATSC8s,MATS1c,MATS4c,MATS5c,MATS2m,MATS3m,MATS5m,MATS6m,  MATS8m,MATS2e,MATS5e,MATS6e,MATS7e,MATS2i,MATS3i,MATS5i,MATS7i,MATS1s,MATS2s,MATS3s,MATS5s,  MATS6s,MATS8s,GATS3c,GATS5c,GATS6c,GATS2m,GATS5m,GATS8m,GATS1v,GATS2v,GATS3v,GATS4v,GATS6v,  GATS7v,GATS8v,GATS1e,GATS2e,GATS5e,GATS7e,GATS8e,GATS1p,GATS6p,GATS2i,GATS3i,GATS5i,GATS7i,GATS1s,  GATS2s,GATS3s,GATS6s,GATS7s,SM1_DzZ,VR1_DzZ,VR2_DzZ,SpDiam_Dzv,SM1_Dzv,VE1_Dzv,VE3_Dzv,VR1_Dzv,  VR2_Dzv,VR3_Dzv,SpMax_Dze,SpMAD_Dze,VE2_Dze,VE3_Dze,SpMAD_Dzp,VE1_Dzp,VE2_Dzp,VR2_Dzp,VR3_Dzp,  SM1_Dzi,VE3_Dzi,SpAbs_Dzs,SpMAD_Dzs,VE1_Dzs,VE2_Dzs,VR1_Dzs,VR2_Dzs,nBase,BCUTw-1h,nBondsS2,  nBondsS3,nBondsD,nBondsT,nBondsM,SpMax4_Bhm,SpMax5_Bhm,SpMax8_Bhm,SpMin1_Bhm,SpMin2_Bhm,SpMin3_Bhm,  SpMin5_Bhm,SpMin7_Bhm,SpMax1_Bhv,SpMax2_Bhv,SpMax6_Bhv,SpMax7_Bhv,SpMin1_Bhv,SpMin2_Bhv,SpMin3_Bhv,  SpMin4_Bhv,SpMin6_Bhv,SpMin8_Bhv,SpMax2_Bhe,SpMax6_Bhe,SpMax7_Bhe,SpMin4_Bhe,SpMin5_Bhe,SpMin6_Bhe,  SpMin8_Bhe,SpMax1_Bhp,SpMax3_Bhp,SpMin1_Bhp,SpMin3_Bhp,SpMin7_Bhp,SpMin8_Bhp,SpMax1_Bhi,SpMax2_Bhi,  SpMax3_Bhi,SpMax4_Bhi,SpMax7_Bhi,SpMin3_Bhi,SpMax1_Bhs,SpMax6_Bhs,SpMax8_Bhs,SpMin4_Bhs,SpMin5_Bhs,  SpMin6_Bhs,SpMin8_Bhs,C1SP1,C2SP2,C3SP2,C1SP3,C2SP3,SCH-5,VCH-3,VCH-5,SC-3,SC-4,SC-6,VC-4,VC-5,VPC-4,  ASP-0,ASP-2,ASP-3,ASP-4,VP-0,VP-1,VP-4,VP-5,AVP-7,Sv,CrippenLogP,CrippenMR,SpMax_Dt,VE1_Dt,VE2_Dt,VE3_Dt,  VR3_Dt,ECCEN,nwHBa,nHBint2,nHBint4,nHBint7,nHBint8,nHBint10,nHsNH3p,nHCsatu,nsssCH,naasC,naaaC,nsNH2,nsssN,  naaO,nsOm,nsSH,naaS,ndssS,nsCl,SHBd,SHBa,SHBint3,SHBint5,SHBint7,SHsOH,SHssNH,SHsssNHp,SHdCH2,SHdsCH,  SsCH3,SdCH2,SssCH2,SaaCH,SdssC,SaasC,SaaaC,SaaNH,SsssNHp,SsOH,SssO,SdS,SssS,SsBr,SsI,minwHBa,minHBint2,  minHBint5,minHBint6,minHBint8,minHBint10,minHsSH,minHsNH3p,minHsssNHp,minHdsCH,minHother,minssCH2,mintCH,  mindsCH,mintsC,minaasC,minaaaC,minsNH3p,minssNH,mintN,minsssN,minaasN,minssssNp,mindO,minaaO,minsOm,minsF,  mindsssP,mindS,minddssS,maxHBd,maxHBa,maxwHBa,maxHBint2,maxHBint3,maxHBint5,maxHBint9,maxHsOH,maxHsNH2,  maxHssNH,maxHdCH2,maxHdsCH,maxHCsatu,maxssCH2,maxtCH,maxdsCH,maxaaCH,maxaaaC,maxsNH3p,maxtN,  maxsssNHp,maxsssN,maxsOH,maxdO,maxssO,maxdS,maxsBr,maxsI,gmin,LipoaffinityIndex,MAXDP,DELS,ETA_dAlpha_A,  ETA_Epsilon_3,ETA_dEpsilon_B,ETA_dEpsilon_D,ETA_Psi_1,ETA_Shape_X,ETA_Beta_s,ETA_dBeta,ETA_dBetaP,ETA_Eta,  ETA_Eta_F,ETA_EtaP_F,ETA_EtaP_L,ETA_EtaP_B_RC,fragC,nHBAcc2,nHBAcc_Lipinski,HybRatio,IC0,IC1,IC2,TIC1,TIC2,  SIC0,SIC1,SIC2,SIC3,SIC4,BIC3,BIC4,ZMIC0,ZMIC1,ZMIC3,Kier3,MLogP,MDEO-12,MDEN-23,MDEN-33,MLFER_A,  piPC3,PetitjeanNumber,n4Ring,n6Ring,nFRing,nF10Ring,nFG12Ring,nTRing,nT12Ring,nTG12Ring,nHeteroRing,  nF6HeteroRing,nF8HeteroRing,nF11HeteroRing,nF12HeteroRing,nT6HeteroRing,nT11HeteroRing,nT12HeteroRing,nRotB,  RotBFrac,nRotBt,LipinskiFailures,topoDiameter,GGI2,GGI3,GGI4,GGI6,GGI7,JGI2,JGI3,JGI6,JGI8,JGI10,JGT,VE3_D,VR1_D,  VR2_D,VR3_D,SRW7,SRW9,WTPT-2,WTPT-3,WTPT-4,WTPT-5,MACCSFP8,MACCSFP13,MACCSFP16,MACCSFP17,  MACCSFP19,MACCSFP23,MACCSFP26,MACCSFP29,MACCSFP30,MACCSFP32,MACCSFP33,MACCSFP36,  MACCSFP37,MACCSFP39,MACCSFP42,MACCSFP45,MACCSFP48,MACCSFP51,MACCSFP55,MACCSFP58,  MACCSFP61,MACCSFP64,MACCSFP65,MACCSFP69,MACCSFP73,MACCSFP77,MACCSFP83,MACCSFP84,  MACCSFP86,MACCSFP88,MACCSFP90,MACCSFP91,MACCSFP95,MACCSFP98,MACCSFP99,MACCSFP101,  MACCSFP103,MACCSFP106,MACCSFP109,MACCSFP116,MACCSFP120,MACCSFP121,MACCSFP122,MACCSFP123,  MACCSFP125,MACCSFP126,MACCSFP127,MACCSFP129,MACCSFP131,MACCSFP135,MACCSFP139,MACCSFP142,  MACCSFP145,MACCSFP147,MACCSFP154,MACCSFP156,MACCSFP157,MACCSFP158,MACCSFP161,MACCSFP162,  MACCSFP163,PubchemFP1,PubchemFP2,PubchemFP12,PubchemFP17,PubchemFP19,PubchemFP21,PubchemFP33,  PubchemFP34,PubchemFP37,PubchemFP117,PubchemFP129,PubchemFP144,PubchemFP146,PubchemFP148,PubchemFP150,  PubchemFP152,PubchemFP153,PubchemFP178,PubchemFP180,PubchemFP182,PubchemFP186,PubchemFP187,PubchemFP189,  PubchemFP190,PubchemFP191,PubchemFP200,PubchemFP213,PubchemFP214,PubchemFP216,PubchemFP217,PubchemFP229,  PubchemFP236,PubchemFP258,PubchemFP261,PubchemFP293,PubchemFP300,PubchemFP305,PubchemFP327,PubchemFP332,  PubchemFP338,PubchemFP341,PubchemFP346,PubchemFP349,PubchemFP357,PubchemFP358,PubchemFP359,PubchemFP364,  PubchemFP365,PubchemFP368,PubchemFP371,PubchemFP374,PubchemFP376,PubchemFP377,PubchemFP378,PubchemFP379,  PubchemFP381,PubchemFP385,PubchemFP386,PubchemFP388,PubchemFP389,PubchemFP390,PubchemFP391,PubchemFP392,  PubchemFP399,PubchemFP406,PubchemFP412,PubchemFP414,PubchemFP416,PubchemFP431,PubchemFP434,PubchemFP437,  PubchemFP439,PubchemFP445,PubchemFP452,PubchemFP453,PubchemFP457,PubchemFP461,PubchemFP466,PubchemFP470,  PubchemFP472,PubchemFP473,PubchemFP474,PubchemFP480,PubchemFP483,PubchemFP494,PubchemFP495,PubchemFP497,  PubchemFP499,PubchemFP504,PubchemFP508,PubchemFP515,PubchemFP520,PubchemFP523,PubchemFP528,PubchemFP529,  PubchemFP531,PubchemFP532,PubchemFP538,PubchemFP541,PubchemFP544,PubchemFP545,PubchemFP546,PubchemFP547,  PubchemFP549,PubchemFP550,PubchemFP554,PubchemFP556,PubchemFP560,PubchemFP565,PubchemFP568,PubchemFP573,  PubchemFP576,PubchemFP577,PubchemFP580,PubchemFP581,PubchemFP583,PubchemFP584,PubchemFP585,PubchemFP591,  PubchemFP592,PubchemFP597,PubchemFP602,PubchemFP609,PubchemFP610,PubchemFP611,PubchemFP615,PubchemFP621,  PubchemFP624,PubchemFP630,PubchemFP634,PubchemFP635,PubchemFP637,PubchemFP643,PubchemFP645,PubchemFP647,  PubchemFP650,PubchemFP653,PubchemFP658,PubchemFP660,PubchemFP663,PubchemFP664,PubchemFP665,PubchemFP668,  PubchemFP669,PubchemFP674,PubchemFP677,PubchemFP679,PubchemFP686,PubchemFP694,PubchemFP696,PubchemFP697,  PubchemFP701,PubchemFP703,PubchemFP709,PubchemFP719,PubchemFP720,PubchemFP724,PubchemFP727,PubchemFP730,  PubchemFP731,PubchemFP735,PubchemFP739,PubchemFP741,PubchemFP742,PubchemFP745,PubchemFP746,PubchemFP750,  PubchemFP763,PubchemFP764,PubchemFP771,PubchemFP773,PubchemFP779,PubchemFP784,PubchemFP785,PubchemFP792,  PubchemFP793,PubchemFP797,PubchemFP800,PubchemFP803,PubchemFP810,PubchemFP814,PubchemFP836,PubchemFP863,  PubchemFP864,PubchemFP878,SubFP5,SubFP13,SubFP15,SubFP19,SubFP21,SubFP26,SubFP27,SubFP35,SubFP36,SubFP41,  SubFP54,SubFP62,SubFP84,SubFP86,SubFP96,SubFP125,SubFP133,SubFP171,SubFP235,SubFP279,SubFP281,SubFP282,  SubFP285,SubFP287,SubFP288 |
|  | **SET03** | nAcid,ALogP,ALogp2,AMR,naAromAtom,nHeavyAtom,nH,nC,nN,nS,nX,ATS0m,ATS1m,ATS4m,ATS7m,ATS8m,ATS7v,ATS8v,ATS1e,ATS3e,ATS4e,ATS5e,ATS6e,ATS7e,ATS8e,ATS0p,ATS2p,ATS0s,ATS2s,ATS3s,ATS4s,ATS5s,ATS6s,ATS7s,AATS0m,AATS5m,AATS0v,AATS4v,AATS6v,AATS7v,AATS0e,AATS1e,AATS2e,AATS3e,nP,ATS4v,AATS5e,AATS6e,AATS7e,AATS8e,AATS0p,AATS1p,AATS3p,AATS4p,AATS6p,AATS7p,AATS8p,AATS3i,AATS4i,AATS6i,AATS0s,AATS2s,AATS4s,AATS8s,ATSC1c,ATSC4c,ATSC5c,ATSC8c,ATSC0m,ATSC1m,ATSC2m,ATSC3m,ATSC7m,ATSC8m,ATSC0v,ATSC4v,ATSC8v,ATSC0e,ATSC2e,ATSC3e,ATSC7e,ATSC0p,ATSC1p,ATSC3p,ATSC5p,ATSC0i,ATSC3i,ATSC4i,ATSC5i,ATSC6i,ATSC0s,ATSC1s,ATSC2s,ATSC6s,ATSC7s,AATSC0c,AATSC1c,AATSC2c,AATSC5c,AATSC8c,AATSC0m,AATSC2m,AATSC3m,AATSC4m,AATSC5m,AATSC1v,AATSC2v,AATSC3v,AATSC4v,AATSC5v,AATSC7v,AATSC0e,AATSC1e,AATSC2e,AATSC4e,AATSC5e,AATSC1p,AATSC4p,AATSC7p,AATSC0i,AATSC1i,AATSC2i,AATSC4i,AATSC6i,AATSC8i,AATSC1s,AATSC2s,AATSC3s,AATSC4s,AATSC6s,AATSC7s,MATS1c,MATS5c,MATS7c,MATS1m,MATS2m,MATS3m,MATS5m,MATS6m,MATS1e,MATS3e,MATS6e,MATS7e,MATS8e,MATS2i,MATS5i,MATS6i,MATS1s,MATS2s,MATS7s,GATS4c,GATS5c,GATS6c,GATS7c,GATS2m,GATS3m,GATS4m,GATS5m,GATS8m,GATS1v,GATS3v,GATS4v,GATS5v,GATS7v,GATS8v,GATS1e,GATS3e,GATS6e,GATS8e,GATS1p,GATS3p,GATS5p,GATS7p,GATS8p,GATS1i,GATS4i,GATS1s,GATS2s,GATS4s,GATS6s,GATS8s,SpMAD_DzZ,SM1_DzZ,VE1_DzZ,VR1_DzZ,SpDiam_Dzv,SpMAD_Dzv,SM1_Dzv,VE1_Dzv,VR1_Dzv,VR3_Dzv,SpMax_Dze,SpMAD_Dze,VE3_Dze,VR3_Dze,SpMAD_Dzp,VE2_Dzp,VR2_Dzp,VR3_Dzp,VE3_Dzi,VR1_Dzi,VE1_Dzs,VE2_Dzs,VR1_Dzs,VR2_Dzs,nBase,BCUTw-1l,BCUTc-1l,BCUTp-1h,nBondsD,nBondsD2,nBondsT,nBondsM,SpMax4_Bhm,SpMax6_Bhm,SpMin1_Bhm,SpMin4_Bhm,SpMin5_Bhm,SpMin6_Bhm,SpMin7_Bhm,SpMin8_Bhm,SpMax1_Bhv,SpMax2_Bhv,SpMax3_Bhv,SpMax5_Bhv,SpMin1_Bhv,SpMin3_Bhv,SpMin4_Bhv,SpMax3_Bhe,SpMax5_Bhe,SpMax6_Bhe,SpMin3_Bhe,SpMin4_Bhe,SpMin5_Bhe,SpMax1_Bhp,SpMax2_Bhp,SpMin2_Bhp,SpMin7_Bhp,SpMin8_Bhp,SpMax2_Bhi,SpMax3_Bhi,SpMax7_Bhi,SpMin1_Bhi,SpMin2_Bhi,SpMax1_Bhs,SpMax2_Bhs,SpMax4_Bhs,SpMax7_Bhs,SpMin1_Bhs,SpMin2_Bhs,SpMin5_Bhs,SpMin6_Bhs,SpMin7_Bhs,SpMin8_Bhs,C1SP1,C1SP2,C2SP2,C3SP2,C3SP3,SCH-3,SCH-6,VCH-5,VCH-6,VCH-7,SC-5,SC-6,VC-3,VC-4,VPC-4,VPC-5,ASP-1,ASP-4,ASP-6,ASP-7,VP-0,VP-4,VP-5,VP-6,AVP-2,AVP-3,AVP-7,CrippenMR,SpMAD_Dt,VE1_Dt,VE2_Dt,VE3_Dt,VR1_Dt,VR2_Dt,VR3_Dt,ECCEN,nHBint3,nHBint4,nHBint7,nHBint8,nHBint9,nHBint10,nHsSH,nHsssNHp,nHtCH,nHdsCH,nHCHnX,nHCsats,nHother,ntCH,ndsCH,naaCH,ntsC,naasC,nssNH,nssssNp,nsOH,nsF,ndsssP,nssS,naaS,ndssS,nsBr,SHBd,SHBa,SHBint4,SHBint8,SHBint9,SHBint10,SHsNH2,SHaaNH,SHtCH,SHdCH2,SHdsCH,SHother,SsCH3,StCH,SaaCH,SdssC,SaaaC,SsNH3p,SsssNHp,SaaN,SsOH,SssO,SdS,SaaS,SsBr,SsI,minwHBa,minHBint5,minHBint6,minHBint10,minHsssNHp,minHdsCH,minHCsatu,minssCH2,mindsCH,minaasC,minsNH3p,minsNH2,mintN,minsssNHp,minaasN,mindO,minsF,minsSH,maxHBd,maxwHBd,maxHBa,maxwHBa,maxHBint2,maxHBint5,maxHssNH,maxHsssNHp,maxHdsCH,maxHAvin,maxsCH3,maxssCH2,maxtCH,maxdsCH,maxaaCH,maxsNH3p,maxtN,maxaaN,maxsOH,maxdO,maxssO,maxaaO,maxdS,maxsBr,gmin,DELS,ETA_dAlpha_A,ETA_Epsilon_4,ETA_dEpsilon_B,ETA_dEpsilon_D,ETA_dPsi_B,ETA_Shape_X,ETA_Beta,ETA_BetaP_s,ETA_dBeta,ETA_dBetaP,ETA_BetaP_ns_d,ETA_Eta,ETA_EtaP_F,ETA_EtaP_L,ETA_EtaP_F_L,ETA_Eta_B_RC,ETA_EtaP_B_RC,nHBAcc_Lipinski,nHBDon,nHBDon_Lipinski,HybRatio,IC0,IC1,IC2,TIC2,SIC2,SIC4,MIC1,ZMIC0,nAtomLC,MDEC-22,MDEO-22,MDEN-12,MDEN-13,MDEN-23,MLFER_BH,TPC,R_TpiPCTPC,n4Ring,nFRing,nF6Ring,nF10Ring,nFG12Ring,nT10Ring,n3HeteroRing,n6HeteroRing,n7HeteroRing,nF6HeteroRing,nF7HeteroRing,nF8HeteroRing,nF12HeteroRing,nFG12HeteroRing,nT6HeteroRing,nT8HeteroRing,nT11HeteroRing,RotBtFrac,GGI2,GGI3,GGI4,GGI5,GGI7,JGI1,JGI2,JGI3,JGI8,JGI9,JGI10,JGT,VE1_D,VE2_D,VE3_D,VR1_D,VR2_D,VR3_D,SRW3,SRW7,WTPT-3,WTPT-5,XLogP,MACCSFP8,MACCSFP23,MACCSFP26,MACCSFP29,MACCSFP32,MACCSFP33,MACCSFP37,MACCSFP39,MACCSFP42,MACCSFP43,MACCSFP49,MACCSFP52,MACCSFP55,MACCSFP57,MACCSFP60,MACCSFP64,MACCSFP65,MACCSFP69,MACCSFP73,MACCSFP75,MACCSFP84,MACCSFP87,MACCSFP88,MACCSFP89,MACCSFP91,MACCSFP92,MACCSFP95,MACCSFP99,MACCSFP101,MACCSFP102,MACCSFP103,MACCSFP108,MACCSFP110,MACCSFP116,MACCSFP117,MACCSFP120,MACCSFP122,MACCSFP123,MACCSFP125,MACCSFP126,MACCSFP127,MACCSFP134,MACCSFP135,MACCSFP138,MACCSFP146,MACCSFP147,MACCSFP156,MACCSFP163,MACCSFP164,PubchemFP1,PubchemFP2,PubchemFP11,PubchemFP12,PubchemFP14,PubchemFP15,PubchemFP17,PubchemFP19,PubchemFP20,PubchemFP21,PubchemFP24,PubchemFP33,PubchemFP34,PubchemFP37,PubchemFP39,PubchemFP44,PubchemFP45,PubchemFP115,PubchemFP131,PubchemFP132,PubchemFP143,PubchemFP157,PubchemFP160,PubchemFP179,PubchemFP180,PubchemFP182,PubchemFP186,PubchemFP189,PubchemFP190,PubchemFP192,PubchemFP193,PubchemFP199,PubchemFP214,PubchemFP227,PubchemFP230,PubchemFP257,PubchemFP294,PubchemFP297,PubchemFP298,PubchemFP300,PubchemFP301,PubchemFP328,PubchemFP337,PubchemFP349,PubchemFP353,PubchemFP355,PubchemFP356,PubchemFP357,PubchemFP361,PubchemFP362,PubchemFP365,PubchemFP366,PubchemFP368,PubchemFP375,PubchemFP377,PubchemFP378,PubchemFP379,PubchemFP382,PubchemFP385,PubchemFP387,PubchemFP391,PubchemFP393,PubchemFP395,PubchemFP396,PubchemFP398,PubchemFP401,PubchemFP406,PubchemFP407,PubchemFP411,PubchemFP412,PubchemFP425,PubchemFP430,PubchemFP431,PubchemFP434,PubchemFP435,PubchemFP449,PubchemFP453,PubchemFP456,PubchemFP468,PubchemFP471,PubchemFP476,PubchemFP481,PubchemFP483,PubchemFP484,PubchemFP485,PubchemFP487,PubchemFP488,PubchemFP490,PubchemFP494,PubchemFP495,PubchemFP502,PubchemFP504,PubchemFP506,PubchemFP507,PubchemFP511,PubchemFP520,PubchemFP523,PubchemFP526,PubchemFP530,PubchemFP532,PubchemFP534,PubchemFP537,PubchemFP538,PubchemFP539,PubchemFP543,PubchemFP546,PubchemFP547,PubchemFP559,PubchemFP563,PubchemFP566,PubchemFP568,PubchemFP569,PubchemFP570,PubchemFP573,PubchemFP575,PubchemFP578,PubchemFP579,PubchemFP580,PubchemFP589,PubchemFP598,PubchemFP602,PubchemFP604,PubchemFP605,PubchemFP606,PubchemFP608,PubchemFP611,PubchemFP612,PubchemFP613,PubchemFP614,PubchemFP616,PubchemFP617,PubchemFP620,PubchemFP622,PubchemFP623,PubchemFP628,PubchemFP632,PubchemFP634,PubchemFP641,PubchemFP645,PubchemFP651,PubchemFP654,PubchemFP660,PubchemFP662,PubchemFP665,PubchemFP666,PubchemFP668,PubchemFP672,PubchemFP680,PubchemFP681,PubchemFP682,PubchemFP683,PubchemFP685,PubchemFP687,PubchemFP696,PubchemFP699,PubchemFP701,PubchemFP702,PubchemFP703,PubchemFP705,PubchemFP706,PubchemFP709,PubchemFP711,PubchemFP712,PubchemFP716,PubchemFP721,PubchemFP722,PubchemFP725,PubchemFP731,PubchemFP736,PubchemFP738,PubchemFP739,PubchemFP747,PubchemFP757,PubchemFP758,PubchemFP761,PubchemFP771,PubchemFP773,PubchemFP783,PubchemFP784,PubchemFP788,PubchemFP791,PubchemFP798,PubchemFP801,PubchemFP806,PubchemFP809,PubchemFP810,PubchemFP812,PubchemFP818,PubchemFP828,PubchemFP830,PubchemFP834,PubchemFP837,PubchemFP841,PubchemFP845,PubchemFP861,PubchemFP877,PubchemFP878,SubFP1,SubFP5,SubFP12,SubFP18,SubFP19,SubFP23,SubFP32,SubFP36,SubFP49,SubFP52,SubFP76,SubFP84,SubFP99,SubFP109,SubFP120,SubFP166,SubFP169,SubFP172,SubFP173,SubFP179,SubFP188,SubFP214,SubFP224,SubFP246,SubFP274,SubFP281,SubFP283,SubFP296,SubFP303 |
|  | **SET04** | ALogP,AMR,naAromAtom,nH,nC,nN,nO,nF,nCl,nBr,nX,ATS1m,ATS2m,ATS3m,ATS4m,ATS5m,ATS8m,ATS7v,ATS8v,ATS1e,ATS7e,ATS8e,ATS2p,ATS5p,ATS1s,ATS2s,ATS3s,ATS6s,ATS7s,AATS0m,AATS1m,AATS2m,AATS4m,AATS5m,AATS7m,AATS8m,AATS3v,AATS4v,AATS0e,AATS1e,AATS2e,AATS3e,nP,nI,ATS4v,AATS4e,AATS0p,AATS1p,AATS3p,AATS4p,AATS5p,AATS8p,AATS0i,AATS1i,AATS5i,AATS7i,AATS1s,AATS2s,AATS3s,AATS6s,AATS7s,AATS8s,ATSC0c,ATSC2c,ATSC3c,ATSC4c,ATSC5c,ATSC7c,ATSC8c,ATSC0m,ATSC1m,ATSC2m,ATSC3m,ATSC4m,ATSC7m,ATSC8m,ATSC0v,ATSC4v,ATSC0e,ATSC1e,ATSC2e,ATSC3e,ATSC4e,ATSC7e,ATSC0p,ATSC2p,ATSC3p,ATSC4p,ATSC5p,ATSC7p,ATSC8p,ATSC2i,ATSC5i,ATSC8i,ATSC0s,ATSC4s,ATSC8s,AATSC0c,AATSC1c,AATSC5c,AATSC6c,AATSC1m,AATSC2m,AATSC3m,AATSC5m,AATSC0v,AATSC4v,AATSC8v,AATSC2e,AATSC4e,AATSC5e,AATSC7e,AATSC0p,AATSC5p,AATSC7p,AATSC8p,AATSC0i,AATSC2i,AATSC3i,AATSC6i,AATSC7i,AATSC2s,AATSC6s,AATSC7s,AATSC8s,MATS1c,MATS2c,MATS3c,MATS1m,MATS3m,MATS4m,MATS6m,MATS7m,MATS8m,MATS1e,MATS3e,MATS7e,MATS8e,MATS2p,MATS1i,MATS2i,MATS3i,MATS5i,MATS7i,MATS2s,MATS5s,MATS6s,GATS3c,GATS7c,GATS8c,GATS1m,GATS2m,GATS5m,GATS2v,GATS4v,GATS5v,GATS6v,GATS4e,GATS5e,GATS1p,GATS2p,GATS4p,GATS5p,GATS6p,GATS1i,GATS3i,GATS5i,GATS7i,GATS8i,GATS3s,GATS6s,SpMAD_DzZ,SM1_DzZ,VR2_DzZ,VE2_Dzv,VE3_Dzv,VR1_Dzv,SM1_Dze,VE1_Dze,VE2_Dze,VE3_Dze,VR1_Dze,VR3_Dzp,SM1_Dzi,VE2_Dzi,VE3_Dzi,VR2_Dzi,VE1_Dzs,BCUTw-1l,BCUTc-1h,BCUTp-1l,BCUTp-1h,nBondsS2,nBondsD,nBondsD2,nBondsT,SpMax2_Bhm,SpMax3_Bhm,SpMax4_Bhm,SpMax6_Bhm,SpMax8_Bhm,SpMin1_Bhm,SpMin3_Bhm,SpMax5_Bhv,SpMax7_Bhv,SpMin3_Bhv,SpMin6_Bhv,SpMax2_Bhe,SpMax7_Bhe,SpMin2_Bhe,SpMin8_Bhe,SpMax2_Bhp,SpMax1_Bhi,SpMin2_Bhi,SpMin8_Bhi,SpMax2_Bhs,SpMax3_Bhs,SpMax4_Bhs,SpMax6_Bhs,SpMax7_Bhs,SpMin1_Bhs,SpMin3_Bhs,SpMin6_Bhs,SpMin8_Bhs,C1SP1,C1SP2,C2SP2,C3SP2,SCH-4,SCH-5,SCH-6,VCH-7,SC-5,VC-3,SPC-5,SP-2,ASP-0,ASP-4,VP-6,AVP-0,AVP-3,AVP-4,AVP-6,Sv,CrippenLogP,VE1_Dt,VE2_Dt,VE3_Dt,VR3_Dt,ECCEN,nwHBd,nwHBa,nHBint3,nHBint7,nHBint8,nHsSH,nHsssNHp,nHdCH2,nHCHnX,nHother,ndsCH,naaCH,nsssCH,ndssC,nssssC,nssNH,naaNH,naaN,nsssN,naaO,nsOm,ndssS,nddssS,nsCl,nsBr,nsI,SHBa,SHBint3,SHBint4,SHBint6,SHBint10,SHaaNH,SHsNH3p,SHtCH,SHdCH2,SHdsCH,SHCsats,SdCH2,SssCH2,StsC,SaaaC,SssssC,SsNH3p,SsNH2,StN,SsssN,SssO,SdsssP,SssS,SaaS,SdssS,SsCl,minHBd,minHBa,minwHBa,minHBint3,minHBint4,minHBint5,minHBint6,minHBint7,minHsSH,minHsNH3p,minHsssNHp,minHCHnX,minHCsatu,minHAvin,mindsCH,minssssC,minsNH3p,minsNH2,mintN,mindsN,minsssN,minsOH,mindO,minssO,mindsssP,minsSH,minssS,mindssS,minsCl,maxHBd,maxHBint2,maxHBint7,maxHBint9,maxHsOH,maxHsSH,maxHsNH3p,maxHdCH2,maxHdsCH,maxHCsats,maxHAvin,maxdCH2,maxssCH2,maxtsC,maxssssC,maxtN,maxaaN,maxsssN,maxssssNp,LipoaffinityIndex,MAXDN,DELS,ETA_AlphaP,ETA_dAlpha_A,ETA_Epsilon_3,ETA_Epsilon_4,ETA_Epsilon_5,ETA_dEpsilon_D,ETA_Psi_1,ETA_Shape_P,ETA_Shape_X,ETA_Beta_s,ETA_BetaP_s,ETA_Beta_ns,ETA_BetaP_ns,ETA_dBeta,ETA_dBetaP,ETA_Beta_ns_d,ETA_Eta,ETA_Eta_F_L,ETA_EtaP_B_RC,nHBAcc3,nHBDon,nHBDon_Lipinski,HybRatio,IC0,IC2,SIC0,SIC1,BIC2,BIC3,BIC5,MIC0,MIC1,ZMIC4,Kier2,nAtomP,MDEC-23,MDEC-33,MDEO-12,MDEO-22,MDEN-11,MDEN-12,MLFER_S,piPC2,piPC3,piPC4,piPC8,nRing,n4Ring,n6Ring,n7Ring,n12Ring,nF6Ring,nF8Ring,nF11Ring,nT4Ring,nT6Ring,nT8Ring,nT10Ring,nT12Ring,n6HeteroRing,n7HeteroRing,nF7HeteroRing,nF10HeteroRing,nF11HeteroRing,nF12HeteroRing,nFG12HeteroRing,nT4HeteroRing,nT6HeteroRing,nT10HeteroRing,nT11HeteroRing,RotBtFrac,topoDiameter,GGI6,GGI8,JGI3,JGI6,JGI10,SpMAD_D,VE1_D,VR2_D,TopoPSA,MWC3,SRW5,WTPT-2,WTPT-4,WTPT-5,XLogP,MACCSFP13,MACCSFP16,MACCSFP24,MACCSFP28,MACCSFP30,MACCSFP33,MACCSFP46,MACCSFP49,MACCSFP50,MACCSFP51,MACCSFP53,MACCSFP54,MACCSFP57,MACCSFP65,MACCSFP78,MACCSFP79,MACCSFP82,MACCSFP87,MACCSFP90,MACCSFP94,MACCSFP95,MACCSFP97,MACCSFP98,MACCSFP106,MACCSFP116,MACCSFP123,MACCSFP124,MACCSFP127,MACCSFP128,MACCSFP137,MACCSFP145,MACCSFP152,MACCSFP159,MACCSFP163,PubchemFP1,PubchemFP15,PubchemFP19,PubchemFP21,PubchemFP130,PubchemFP145,PubchemFP149,PubchemFP153,PubchemFP159,PubchemFP160,PubchemFP178,PubchemFP179,PubchemFP189,PubchemFP192,PubchemFP217,PubchemFP234,PubchemFP255,PubchemFP256,PubchemFP257,PubchemFP261,PubchemFP287,PubchemFP292,PubchemFP293,PubchemFP298,PubchemFP300,PubchemFP301,PubchemFP327,PubchemFP329,PubchemFP330,PubchemFP332,PubchemFP336,PubchemFP339,PubchemFP340,PubchemFP341,PubchemFP346,PubchemFP348,PubchemFP358,PubchemFP363,PubchemFP364,PubchemFP368,PubchemFP372,PubchemFP373,PubchemFP381,PubchemFP383,PubchemFP389,PubchemFP393,PubchemFP400,PubchemFP418,PubchemFP423,PubchemFP425,PubchemFP432,PubchemFP434,PubchemFP437,PubchemFP439,PubchemFP443,PubchemFP445,PubchemFP450,PubchemFP452,PubchemFP453,PubchemFP459,PubchemFP462,PubchemFP466,PubchemFP472,PubchemFP473,PubchemFP474,PubchemFP477,PubchemFP483,PubchemFP484,PubchemFP493,PubchemFP495,PubchemFP499,PubchemFP504,PubchemFP514,PubchemFP515,PubchemFP520,PubchemFP524,PubchemFP528,PubchemFP531,PubchemFP536,PubchemFP537,PubchemFP541,PubchemFP544,PubchemFP547,PubchemFP551,PubchemFP553,PubchemFP555,PubchemFP556,PubchemFP564,PubchemFP575,PubchemFP577,PubchemFP581,PubchemFP584,PubchemFP585,PubchemFP587,PubchemFP592,PubchemFP595,PubchemFP602,PubchemFP603,PubchemFP607,PubchemFP611,PubchemFP617,PubchemFP621,PubchemFP636,PubchemFP640,PubchemFP641,PubchemFP642,PubchemFP644,PubchemFP645,PubchemFP646,PubchemFP650,PubchemFP652,PubchemFP654,PubchemFP655,PubchemFP659,PubchemFP665,PubchemFP666,PubchemFP667,PubchemFP668,PubchemFP669,PubchemFP674,PubchemFP676,PubchemFP677,PubchemFP679,PubchemFP686,PubchemFP697,PubchemFP709,PubchemFP712,PubchemFP714,PubchemFP715,PubchemFP720,PubchemFP729,PubchemFP730,PubchemFP734,PubchemFP739,PubchemFP741,PubchemFP742,PubchemFP743,PubchemFP746,PubchemFP750,PubchemFP758,PubchemFP760,PubchemFP761,PubchemFP763,PubchemFP764,PubchemFP767,PubchemFP769,PubchemFP777,PubchemFP785,PubchemFP790,PubchemFP791,PubchemFP797,PubchemFP798,PubchemFP800,PubchemFP801,PubchemFP810,PubchemFP816,PubchemFP818,PubchemFP822,PubchemFP831,PubchemFP833,PubchemFP836,PubchemFP841,PubchemFP864,PubchemFP877,SubFP15,SubFP16,SubFP19,SubFP23,SubFP27,SubFP32,SubFP41,SubFP49,SubFP74,SubFP75,SubFP76,SubFP86,SubFP88,SubFP96,SubFP125,SubFP129,SubFP133,SubFP136,SubFP150,SubFP173,SubFP179,SubFP182,SubFP188,SubFP214,SubFP275,SubFP281,SubFP283,SubFP285,SubFP287 |
|  | **SET05** | nAcid,ALogP,ALogp2,naAromAtom,nHeavyAtom,nH,nC,nN,nO,nS,ATS0m,ATS1m,ATS2m,ATS5m,ATS6m,ATS7m,ATS8m,ATS6v,ATS7v,ATS8v,ATS3e,ATS4e,ATS6e,ATS7e,ATS8e,ATS0p,ATS5p,ATS0s,ATS4s,ATS5s,ATS7s,AATS2m,AATS3m,AATS4m,AATS5m,AATS6m,AATS0v,AATS4v,AATS5v,AATS7v,AATS8v,AATS0e,AATS1e,nP,AATS4e,AATS7e,AATS8e,AATS0p,AATS2p,AATS3p,AATS4p,AATS6p,AATS8p,AATS1i,AATS4i,AATS8i,AATS0s,AATS1s,AATS3s,AATS4s,AATS5s,AATS6s,AATS8s,ATSC1c,ATSC2c,ATSC3c,ATSC6c,ATSC8c,ATSC0m,ATSC1m,ATSC2m,ATSC3m,ATSC4m,ATSC5m,ATSC8m,ATSC1v,ATSC2v,ATSC3v,ATSC5v,ATSC6v,ATSC7v,ATSC8v,ATSC1e,ATSC6e,ATSC8e,ATSC0p,ATSC6p,ATSC7p,ATSC0i,ATSC2i,ATSC4i,ATSC6i,ATSC1s,ATSC2s,ATSC3s,ATSC4s,ATSC5s,ATSC6s,ATSC7s,AATSC0c,AATSC1c,AATSC5c,AATSC6c,AATSC4m,AATSC7m,AATSC0v,AATSC1v,AATSC2v,AATSC3v,AATSC5v,AATSC6v,AATSC7v,AATSC8v,AATSC0e,AATSC2e,AATSC7e,AATSC8e,AATSC1p,AATSC2p,AATSC4p,AATSC8p,AATSC1i,AATSC6i,AATSC7i,AATSC1s,AATSC2s,AATSC4s,AATSC5s,AATSC6s,AATSC8s,MATS2c,MATS4c,MATS6c,MATS7c,MATS3m,MATS6m,MATS8m,MATS3e,MATS4e,MATS5e,MATS6e,MATS7e,MATS8e,MATS2p,MATS1i,MATS3i,MATS4i,MATS7i,MATS1s,MATS6s,MATS7s,MATS8s,GATS6c,GATS8c,GATS2m,GATS4m,GATS6m,GATS8m,GATS1v,GATS2v,GATS4v,GATS5v,GATS6v,GATS8v,GATS1e,GATS5e,GATS6e,GATS8e,GATS1p,GATS2i,GATS4i,GATS5i,GATS1s,GATS2s,GATS3s,SpMAD_DzZ,SM1_DzZ,VE3_DzZ,VR1_DzZ,VR2_DzZ,SpDiam_Dzv,SM1_Dzv,VE1_Dzv,VE3_Dzv,VR1_Dzv,VR2_Dzv,VR3_Dzv,SpMax_Dze,SpMAD_Dze,VE2_Dze,VE3_Dze,VR1_Dze,VR2_Dze,VE1_Dzp,VE2_Dzp,VR3_Dzp,SM1_Dzi,VE1_Dzi,VE2_Dzi,VR1_Dzi,VR2_Dzi,SpAbs_Dzs,SpMAD_Dzs,VE1_Dzs,VE2_Dzs,VR2_Dzs,BCUTw-1l,BCUTw-1h,BCUTc-1h,nBondsS2,nBondsS3,nBondsD,nBondsT,SpMax1_Bhm,SpMax5_Bhm,SpMax7_Bhm,SpMax8_Bhm,SpMin2_Bhm,SpMin4_Bhm,SpMin7_Bhm,SpMin8_Bhm,SpMax1_Bhv,SpMax2_Bhv,SpMax3_Bhv,SpMax4_Bhv,SpMax6_Bhv,SpMax7_Bhv,SpMin2_Bhv,SpMin3_Bhv,SpMin4_Bhv,SpMin5_Bhv,SpMin6_Bhv,SpMin7_Bhv,SpMax5_Bhe,SpMax7_Bhe,SpMax8_Bhe,SpMin3_Bhe,SpMin5_Bhe,SpMin6_Bhe,SpMax1_Bhp,SpMax2_Bhp,SpMax3_Bhp,SpMin1_Bhp,SpMin3_Bhp,SpMin7_Bhp,SpMax2_Bhi,SpMax4_Bhi,SpMax7_Bhi,SpMin1_Bhi,SpMin2_Bhi,SpMin3_Bhi,SpMax1_Bhs,SpMax4_Bhs,SpMax5_Bhs,SpMax6_Bhs,SpMax8_Bhs,SpMin2_Bhs,SpMin6_Bhs,C2SP1,C2SP2,C1SP3,C2SP3,SCH-4,SCH-7,VCH-5,VCH-6,VCH-7,SC-3,SC-5,VC-3,VC-4,VC-5,SPC-4,SPC-5,VPC-4,VPC-5,ASP-4,ASP-6,VP-0,VP-4,AVP-0,AVP-2,AVP-3,AVP-6,AVP-7,CrippenLogP,VE2_Dt,VE3_Dt,VR1_Dt,VR3_Dt,ECCEN,nHBd,nHBa,nwHBa,nHBint2,nHBint4,nHBint5,nHBint7,nHsSH,nHaaNH,nHsNH3p,nHsssNHp,nHdsCH,nHaaCH,nHCHnX,nHCsatu,nHAvin,nHother,nssCH2,ndsCH,naaCH,nsssCH,ntsC,ndssC,nsNH3p,nssNH,nsssNHp,naaN,nsssN,naasN,nssssNp,ndO,nsOm,nsSH,nsCl,nsBr,nsI,SHBa,SHBint2,SHBint7,SHBint9,SHsOH,SHsNH2,SHssNH,SHtCH,SHCsats,SHCsatu,SHAvin,SdsCH,SaaCH,SdssC,SaasC,SssssC,SsNH2,SssNH,StN,SsssN,SaasN,SsOm,SdsssP,SsSH,SaaS,SdssS,SddssS,SsCl,SsBr,SsI,minHBd,minHBa,minwHBa,minHBint2,minHBint3,minHBint6,minHsOH,minHssNH,minHaaNH,minHtCH,minHdCH2,minHAvin,minsCH3,mindCH2,minssCH2,mintCH,minsssCH,mindssC,minsNH2,minssNH,minaaNH,mindsN,minaaN,minaasN,minssssNp,mindO,minssO,minaaO,mindsssP,mindS,minssS,minaaS,mindssS,minddssS,minsCl,maxHBd,maxHBa,maxwHBa,maxHBint2,maxHBint3,maxHBint4,maxHBint5,maxHBint6,maxHBint7,maxHBint9,maxHsOH,maxHsNH2,maxHssNH,maxHaaNH,maxHdsCH,maxHaaCH,maxHCsats,maxssCH2,maxtCH,maxssssC,maxsNH2,maxssNH,maxaaNH,maxsssNHp,maxdsN,maxaaN,maxaasN,maxdO,maxssO,maxdS,maxsBr,maxsI,meanI,hmax,gmin,LipoaffinityIndex,MAXDN,MAXDP,DELS,ETA_Alpha,ETA_Epsilon_2,ETA_Epsilon_3,ETA_Epsilon_4,ETA_Epsilon_5,ETA_dEpsilon_B,ETA_Psi_1,ETA_dPsi_B,ETA_Shape_Y,ETA_Beta_s,ETA_BetaP_s,ETA_BetaP_ns,ETA_dBeta,ETA_dBetaP,ETA_Eta,ETA_Eta_F,ETA_EtaP_F,ETA_Eta_L,ETA_EtaP_L,ETA_Eta_F_L,ETA_EtaP_F_L,ETA_Eta_B,ETA_EtaP_B,ETA_Eta_B_RC,ETA_EtaP_B_RC,nHBAcc,nHBAcc3,nHBDon,IC1,TIC1,TIC2,SIC0,SIC1,SIC2,SIC3,CIC0,BIC2,BIC4,BIC5,MIC0,MIC1,MIC3,ZMIC2,ZMIC3,ZMIC4,Kier1,Kier3,nAtomP,nAtomLAC,MLogP,MDEC-11,MDEC-12,MDEC-13,MDEC-14,MDEC-24,MDEC-33,MDEC-44,MDEO-11,MDEO-22,MDEN-11,MDEN-12,MDEN-13,MDEN-23,MDEN-33,MLFER_A,MLFER_BH,MLFER_S,piPC1,piPC8,R_TpiPCTPC,n3Ring,n4Ring,n6Ring,n8Ring,n12Ring,nFRing,nF8Ring,nF10Ring,nF11Ring,nF12Ring,nFG12Ring,nTRing,nT4Ring,nT5Ring,nT7Ring,nT8Ring,nT9Ring,nT10Ring,n3HeteroRing,n4HeteroRing,n5HeteroRing,nF6HeteroRing,nF9HeteroRing,nF10HeteroRing,nF12HeteroRing,nT4HeteroRing,nT7HeteroRing,nT9HeteroRing,nT10HeteroRing,nT12HeteroRing,nTG12HeteroRing,nRotB,nRotBt,topoRadius,topoShape,GGI5,GGI6,GGI7,GGI8,GGI9,JGI1,JGI3,JGI4,JGI6,JGT,SpMAD_D,VE1_D,VE2_D,VE3_D,VR1_D,VR2_D,VR3_D,TopoPSA,MWC3,MWC6,SRW7,MW,WTPT-4,MACCSFP8,MACCSFP11,MACCSFP13,MACCSFP16,MACCSFP17,MACCSFP21,MACCSFP23,MACCSFP24,MACCSFP27,MACCSFP29,MACCSFP30,MACCSFP32,MACCSFP33,MACCSFP37,MACCSFP39,MACCSFP40,MACCSFP42,MACCSFP45,MACCSFP49,MACCSFP50,MACCSFP51,MACCSFP54,MACCSFP57,MACCSFP58,MACCSFP59,MACCSFP60,MACCSFP61,MACCSFP64,MACCSFP65,MACCSFP66,MACCSFP68,MACCSFP69,MACCSFP70,MACCSFP71,MACCSFP72,MACCSFP73,MACCSFP74,MACCSFP76,MACCSFP83,MACCSFP84,MACCSFP85,MACCSFP87,MACCSFP91,MACCSFP92,MACCSFP93,MACCSFP95,MACCSFP97,MACCSFP98,MACCSFP100,MACCSFP103,MACCSFP104,MACCSFP105,MACCSFP106,MACCSFP108,MACCSFP109,MACCSFP112,MACCSFP113,MACCSFP118,MACCSFP119,MACCSFP123,MACCSFP124,MACCSFP125,MACCSFP126,MACCSFP127,MACCSFP129,MACCSFP131,MACCSFP132,MACCSFP135,MACCSFP136,MACCSFP137,MACCSFP140,MACCSFP142,MACCSFP145,MACCSFP148,MACCSFP149,MACCSFP153,MACCSFP154,MACCSFP155,MACCSFP160,MACCSFP161,MACCSFP162,MACCSFP164,MACCSFP165,PubchemFP1,PubchemFP2,PubchemFP3,PubchemFP11,PubchemFP12,PubchemFP13,PubchemFP14,PubchemFP15,PubchemFP16,PubchemFP19,PubchemFP20,PubchemFP24,PubchemFP25,PubchemFP30,PubchemFP34,PubchemFP37,PubchemFP38,PubchemFP44,PubchemFP46,PubchemFP118,PubchemFP132,PubchemFP143,PubchemFP144,PubchemFP145,PubchemFP148,PubchemFP152,PubchemFP153,PubchemFP157,PubchemFP164,PubchemFP178,PubchemFP184,PubchemFP186,PubchemFP187,PubchemFP234,PubchemFP237,PubchemFP256,PubchemFP257,PubchemFP259,PubchemFP286,PubchemFP292,PubchemFP293,PubchemFP294,PubchemFP298,PubchemFP299,PubchemFP301,PubchemFP327,PubchemFP334,PubchemFP335,PubchemFP340,PubchemFP342,PubchemFP370,PubchemFP373,PubchemFP377,PubchemFP378,PubchemFP379,PubchemFP386,PubchemFP387,PubchemFP392,PubchemFP416,PubchemFP418,PubchemFP420,PubchemFP429,PubchemFP434,PubchemFP437,PubchemFP442,PubchemFP445,PubchemFP452,PubchemFP453,PubchemFP456,PubchemFP461,PubchemFP462,PubchemFP464,PubchemFP468,PubchemFP471,PubchemFP472,PubchemFP474,PubchemFP479,PubchemFP484,PubchemFP485,PubchemFP487,PubchemFP491,PubchemFP493,PubchemFP495,PubchemFP499,PubchemFP502,PubchemFP504,PubchemFP513,PubchemFP514,PubchemFP520,PubchemFP528,PubchemFP531,PubchemFP532,PubchemFP541,PubchemFP542,PubchemFP544,PubchemFP545,PubchemFP548,PubchemFP551,PubchemFP556,PubchemFP564,PubchemFP576,PubchemFP577,PubchemFP581,PubchemFP582,PubchemFP587,PubchemFP590,PubchemFP592,PubchemFP595,PubchemFP598,PubchemFP602,PubchemFP603,PubchemFP605,PubchemFP609,PubchemFP610,PubchemFP611,PubchemFP621,PubchemFP622,PubchemFP623,PubchemFP641,PubchemFP643,PubchemFP644,PubchemFP650,PubchemFP651,PubchemFP653,PubchemFP665,PubchemFP672,PubchemFP677,PubchemFP679,PubchemFP682,PubchemFP685,PubchemFP686,PubchemFP690,PubchemFP691,PubchemFP693,PubchemFP695,PubchemFP696,PubchemFP699,PubchemFP700,PubchemFP703,PubchemFP704,PubchemFP705,PubchemFP710,PubchemFP711,PubchemFP713,PubchemFP714,PubchemFP716,PubchemFP719,PubchemFP720,PubchemFP725,PubchemFP727,PubchemFP730,PubchemFP739,PubchemFP741,PubchemFP742,PubchemFP746,PubchemFP747,PubchemFP749,PubchemFP750,PubchemFP755,PubchemFP756,PubchemFP758,PubchemFP760,PubchemFP763,PubchemFP764,PubchemFP765,PubchemFP767,PubchemFP769,PubchemFP772,PubchemFP776,PubchemFP777,PubchemFP779,PubchemFP780,PubchemFP783,PubchemFP785,PubchemFP790,PubchemFP799,PubchemFP800,PubchemFP801,PubchemFP814,PubchemFP819,PubchemFP821,PubchemFP830,PubchemFP836,PubchemFP860,PubchemFP861,PubchemFP878,SubFP14,SubFP19,SubFP21,SubFP26,SubFP41,SubFP49,SubFP62,SubFP68,SubFP75,SubFP86,SubFP88,SubFP96,SubFP129,SubFP133,SubFP181,SubFP183,SubFP214,SubFP224,SubFP237,SubFP278,SubFP279,SubFP283,SubFP288,SubFP296 |
| PSO | **SET01** | nAcid,ALogp2,AMR,nAromBond,nO,nS,nBr,nX,ATS0m,ATS1m,ATS3m,ATS7m,ATS6v,ATS1e,ATS4e,ATS5e,ATS6e,ATS7e,ATS8e,ATS2s,ATS3s,ATS5s,AATS2v,AATS3v,AATS2e,AATS6e,AATS8e,AATS5p,AATS6p,AATS0s,AATS1s,ATSC7c,ATSC1m,ATSC4m,ATSC8m,ATSC0v,ATSC8v,ATSC6e,ATSC0i,ATSC1i,ATSC6s,AATSC1c,AATSC4c,AATSC6c,AATSC7c,AATSC8c,AATSC1m,AATSC0v,AATSC1v,AATSC4v,AATSC6v,AATSC2e,AATSC5e,AATSC5p,AATSC0i,AATSC1i,AATSC5i,AATSC8i,AATSC1s,AATSC2s,AATSC5s,MATS3c,MATS6c,MATS7c,MATS3m,MATS4m,MATS3e,MATS5e,MATS6e,MATS7e,MATS1i,MATS3i,MATS7i,MATS2s,MATS3s,MATS5s,MATS6s,MATS7s,GATS1c,GATS6c,GATS1m,GATS7m,GATS1v,GATS4v,GATS7v,GATS8v,GATS2e,GATS5e,GATS2p,GATS3p,GATS8p,GATS3i,GATS5i,GATS6i,GATS7i,GATS8i,GATS1s,GATS2s,GATS5s,SM1_DzZ,VR1_DzZ,SM1_Dze,VR1_Dze,VR2_Dze,VR1_Dzp,SpAbs_Dzs,SpMAD_Dzs,SM1_Dzs,nBase,BCUTw-1h,BCUTp-1l,nBondsS2,nBondsM,SpMax3_Bhm,SpMax4_Bhm,SpMax5_Bhm,SpMax6_Bhm,SpMax7_Bhm,SpMin2_Bhm,SpMin4_Bhm,SpMin5_Bhm,SpMax2_Bhv,SpMax8_Bhv,SpMin1_Bhv,SpMin5_Bhv,SpMin6_Bhv,SpMin8_Bhv,SpMax2_Bhe,SpMax3_Bhe,SpMin2_Bhe,SpMin4_Bhe,SpMin8_Bhe,SpMax3_Bhi,SpMax4_Bhi,SpMax3_Bhs,SpMax7_Bhs,SpMin3_Bhs,SpMin7_Bhs,C1SP1,C1SP2,C2SP2,C3SP2,C3SP3,SCH-4,SCH-6,SCH-7,VCH-5,SC-3,SC-4,SC-5,VC-3,VC-5,VC-6,VPC-4,SP-2,ASP-2,ASP-4,ASP-6,VP-1,VP-2,VP-5,AVP-7,CrippenLogP,SpMax_Dt,SpMAD_Dt,VR2_Dt,ECCEN,nHBd,nwHBa,nHsNH2,nHaaCH,nHCsats,nHother,nsCH3,nssCH2,ntCH,naaCH,nsssCH,naaaC,naaNH,naaN,nsssN,ndO,nssO,naaO,nsCl,SHBd,SHBint3,SHBint5,SHssNH,SHCsatu,SHAvin,SaaCH,SaaaC,SdsN,SaaN,SsssN,SsOH,SsF,SdS,SsBr,minHBa,minHBint2,minHBint5,minHBint8,minHBint9,minHssNH,minHdsCH,minHaaCH,minHCsatu,minHAvin,minssCH2,minaaCH,minaasC,minsNH3p,mintN,minsssNHp,minaasN,mindO,minssO,minsF,mindsssP,mindS,maxHBd,maxHBa,maxwHBa,maxHsNH2,maxHssNH,maxHdsCH,maxHaaCH,maxsCH3,maxaaCH,maxaaaC,maxsOH,maxdO,maxssO,maxsCl,gmin,LipoaffinityIndex,ETA_Alpha,ETA_dEpsilon_B,ETA_dEpsilon_D,ETA_dPsi_B,ETA_Shape_P,ETA_dBetaP,ETA_Eta_L,ETA_Eta_B_RC,ETA_EtaP_B_RC,fragC,HybRatio,IC0,IC1,IC2,TIC1,TIC2,SIC3,SIC4,BIC2,BIC3,MIC0,MIC1,ZMIC1,Kier2,nAtomLAC,MDEC-22,MDEC-23,MDEO-12,MDEN-23,PetitjeanNumber,nFRing,nF6Ring,nF10Ring,nF12Ring,nFG12Ring,nTRing,nTG12Ring,n5HeteroRing,nT6HeteroRing,nT9HeteroRing,nT11HeteroRing,RotBtFrac,topoRadius,topoDiameter,GGI2,GGI3,GGI4,GGI5,GGI7,JGI2,JGI3,JGI5,JGI6,JGI8,JGI10,VE2_D,VE3_D,VR1_D,VR3_D,MWC3,MWC6,WTPT-2,WTPT-4,WTPT-5,XLogP,MACCSFP8,MACCSFP11,MACCSFP16,MACCSFP25,MACCSFP29,MACCSFP32,MACCSFP33,MACCSFP37,MACCSFP38,MACCSFP39,MACCSFP42,MACCSFP52,MACCSFP55,MACCSFP57,MACCSFP58,MACCSFP60,MACCSFP66,MACCSFP69,MACCSFP71,MACCSFP76,MACCSFP77,MACCSFP79,MACCSFP91,MACCSFP95,MACCSFP96,MACCSFP98,MACCSFP101,MACCSFP106,MACCSFP108,MACCSFP116,MACCSFP118,MACCSFP119,MACCSFP120,MACCSFP121,MACCSFP122,MACCSFP126,MACCSFP137,MACCSFP139,MACCSFP143,MACCSFP145,MACCSFP148,MACCSFP154,MACCSFP158,MACCSFP162,MACCSFP165,PubchemFP1,PubchemFP2,PubchemFP3,PubchemFP13,PubchemFP14,PubchemFP18,PubchemFP19,PubchemFP21,PubchemFP24,PubchemFP33,PubchemFP37,PubchemFP44,PubchemFP45,PubchemFP116,PubchemFP129,PubchemFP143,PubchemFP146,PubchemFP160,PubchemFP180,PubchemFP186,PubchemFP190,PubchemFP191,PubchemFP192,PubchemFP200,PubchemFP206,PubchemFP214,PubchemFP219,PubchemFP229,PubchemFP258,PubchemFP259,PubchemFP286,PubchemFP297,PubchemFP330,PubchemFP333,PubchemFP339,PubchemFP340,PubchemFP343,PubchemFP346,PubchemFP349,PubchemFP358,PubchemFP360,PubchemFP362,PubchemFP364,PubchemFP366,PubchemFP368,PubchemFP376,PubchemFP380,PubchemFP382,PubchemFP386,PubchemFP391,PubchemFP392,PubchemFP394,PubchemFP401,PubchemFP403,PubchemFP406,PubchemFP412,PubchemFP414,PubchemFP416,PubchemFP418,PubchemFP420,PubchemFP425,PubchemFP431,PubchemFP432,PubchemFP437,PubchemFP443,PubchemFP445,PubchemFP446,PubchemFP447,PubchemFP450,PubchemFP455,PubchemFP457,PubchemFP458,PubchemFP466,PubchemFP470,PubchemFP474,PubchemFP475,PubchemFP478,PubchemFP482,PubchemFP487,PubchemFP495,PubchemFP496,PubchemFP497,PubchemFP499,PubchemFP501,PubchemFP504,PubchemFP507,PubchemFP509,PubchemFP514,PubchemFP515,PubchemFP522,PubchemFP523,PubchemFP524,PubchemFP531,PubchemFP534,PubchemFP535,PubchemFP536,PubchemFP545,PubchemFP547,PubchemFP549,PubchemFP551,PubchemFP555,PubchemFP559,PubchemFP564,PubchemFP566,PubchemFP569,PubchemFP575,PubchemFP577,PubchemFP581,PubchemFP584,PubchemFP587,PubchemFP591,PubchemFP602,PubchemFP606,PubchemFP607,PubchemFP608,PubchemFP612,PubchemFP614,PubchemFP615,PubchemFP618,PubchemFP620,PubchemFP621,PubchemFP628,PubchemFP630,PubchemFP638,PubchemFP639,PubchemFP641,PubchemFP650,PubchemFP653,PubchemFP654,PubchemFP655,PubchemFP656,PubchemFP657,PubchemFP660,PubchemFP670,PubchemFP673,PubchemFP675,PubchemFP677,PubchemFP681,PubchemFP684,PubchemFP686,PubchemFP688,PubchemFP689,PubchemFP698,PubchemFP700,PubchemFP706,PubchemFP709,PubchemFP716,PubchemFP720,PubchemFP721,PubchemFP722,PubchemFP725,PubchemFP738,PubchemFP741,PubchemFP746,PubchemFP757,PubchemFP758,PubchemFP764,PubchemFP772,PubchemFP773,PubchemFP774,PubchemFP779,PubchemFP782,PubchemFP784,PubchemFP788,PubchemFP790,PubchemFP791,PubchemFP810,PubchemFP813,PubchemFP814,PubchemFP818,PubchemFP825,PubchemFP860,PubchemFP861,SubFP1,SubFP5,SubFP6,SubFP12,SubFP16,SubFP21,SubFP23,SubFP27,SubFP35,SubFP52,SubFP62,SubFP76,SubFP133,SubFP135,SubFP143,SubFP150,SubFP153,SubFP171,SubFP172,SubFP174,SubFP181,SubFP182,SubFP184,SubFP201,SubFP212,SubFP274,SubFP280,SubFP283,SubFP294,SubFP303 |
|  | **SET02** | nAcid,nAromBond,nO,nS,nX,ATS0m,ATS7m,ATS6v,ATS1e,ATS3e,ATS5e,ATS6e,ATS7e,ATS8e,ATS0p,ATS1s,ATS2s,ATS5s,AATS2m,AATS0v,AATS7v,AATS0e,AATS2e,AATS3e,ATS4v,AATS4e,AATS6e,AATS7e,AATS8e,AATS0p,AATS6p,AATS8p,AATS6i,AATS7i,AATS0s,AATS6s,ATSC0c,ATSC2c,ATSC3c,ATSC2m,ATSC4m,ATSC8m,ATSC0v,ATSC7v,ATSC0e,ATSC3e,ATSC4e,ATSC0i,ATSC1i,ATSC3i,ATSC2s,ATSC8s,AATSC0c,AATSC4c,AATSC7c,AATSC1m,AATSC6m,AATSC0v,AATSC1v,AATSC6v,AATSC1e,AATSC2e,AATSC5e,AATSC7e,AATSC3p,AATSC5p,AATSC7p,AATSC8p,AATSC0i,AATSC1i,AATSC5i,AATSC8i,AATSC1s,AATSC3s,AATSC5s,AATSC6s,MATS5c,MATS6c,MATS3m,MATS4m,MATS1i,MATS2s,MATS4s,MATS6s,GATS1c,GATS6c,GATS7c,GATS1m,GATS5m,GATS7m,GATS8v,GATS5e,GATS3p,GATS2i,GATS3i,GATS6i,GATS7i,GATS8i,GATS2s,GATS5s,SM1_DzZ,VR1_DzZ,SpDiam_Dzv,SM1_Dzv,VE3_Dzv,VR3_Dzv,SpMAD_Dze,SM1_Dze,VE1_Dze,VR1_Dze,VR2_Dze,VR1_Dzp,VR2_Dzp,VR3_Dzp,VE3_Dzi,SpAbs_Dzs,SM1_Dzs,nBase,BCUTw-1h,nBondsS2,nBondsS3,nBondsD,nBondsM,SpMax1_Bhm,SpMax3_Bhm,SpMax4_Bhm,SpMax5_Bhm,SpMin2_Bhm,SpMin4_Bhm,SpMin5_Bhm,SpMin6_Bhm,SpMax3_Bhv,SpMax8_Bhv,SpMin1_Bhv,SpMax2_Bhe,SpMax5_Bhe,SpMax3_Bhp,SpMin1_Bhp,SpMin2_Bhp,SpMax2_Bhi,SpMax4_Bhi,SpMin2_Bhi,SpMax5_Bhs,SpMax7_Bhs,SpMin7_Bhs,C1SP1,C1SP2,C2SP2,C3SP2,C1SP3,C3SP3,SCH-4,SCH-6,SCH-7,VCH-3,VCH-5,VCH-7,SC-3,SC-5,VC-3,VC-5,VC-6,SPC-5,SP-2,ASP-0,ASP-2,ASP-4,ASP-6,VP-0,VP-1,VP-2,VP-3,VP-5,CrippenLogP,CrippenMR,SpMax_Dt,SpMAD_Dt,VE2_Dt,VR3_Dt,ECCEN,nHBd,nHBint5,nHsSH,nHsNH3p,nHsssNHp,nHaaCH,nHCHnX,nsCH3,naaCH,nsssCH,naasC,nsssN,ndO,naaS,ndssS,nsCl,SHBd,SHBa,SHBint3,SHBint6,SHsSH,SHsNH2,SHCsatu,SaaCH,SsssCH,SaaaC,SssssC,SsssNHp,SaaN,SsssN,SsOH,SsOm,SsF,SaaS,SsBr,minHBa,minHBint2,minHBint5,minHBint6,minHBint8,minHBint10,minHaaNH,minHsssNHp,minHdsCH,minHaaCH,minHCsatu,minHAvin,minssCH2,mintsC,minaasC,minsNH2,minssNH,mintN,minaasN,mindO,minssO,minsOm,mindsssP,minsI,maxHBd,maxHBa,maxwHBa,maxHBint2,maxHBint5,maxHBint6,maxHsOH,maxHsNH2,maxHssNH,maxHdCH2,maxHCsatu,maxHAvin,maxsCH3,maxdCH2,maxtCH,maxdsCH,maxaaCH,maxaaaC,maxtN,maxssssNp,maxdO,maxaaO,gmin,LipoaffinityIndex,MAXDN,MAXDP,ETA_Epsilon_4,ETA_dEpsilon_B,ETA_dPsi_B,ETA_Shape_X,ETA_Eta_F_L,ETA_EtaP_F_L,ETA_Eta_B_RC,ETA_EtaP_B_RC,fragC,nHBAcc3,nHBDon,HybRatio,TIC1,SIC1,SIC3,SIC4,BIC3,BIC4,MIC0,MIC1,ZMIC2,Kier2,nAtomP,MDEC-13,MDEC-22,MDEC-23,MDEC-33,MDEO-11,MDEO-12,MDEN-22,MDEN-23,MLFER_A,piPC3,piPC4,piPC8,PetitjeanNumber,nFRing,nF6Ring,nF11Ring,nFG12Ring,nTRing,nT12Ring,nTG12Ring,nHeteroRing,n5HeteroRing,n8HeteroRing,nF12HeteroRing,nT9HeteroRing,nT11HeteroRing,nT12HeteroRing,RotBtFrac,LipinskiFailures,topoShape,GGI2,GGI4,GGI5,GGI7,JGI1,JGI2,JGI3,JGI5,JGI6,JGI8,VE1_D,VE2_D,VR2_D,MWC3,MWC6,SRW5,SRW7,WTPT-2,WTPT-4,WTPT-5,XLogP,MACCSFP8,MACCSFP11,MACCSFP32,MACCSFP33,MACCSFP39,MACCSFP52,MACCSFP55,MACCSFP57,MACCSFP59,MACCSFP64,MACCSFP65,MACCSFP68,MACCSFP75,MACCSFP79,MACCSFP80,MACCSFP81,MACCSFP84,MACCSFP88,MACCSFP95,MACCSFP96,MACCSFP98,MACCSFP101,MACCSFP103,MACCSFP106,MACCSFP108,MACCSFP110,MACCSFP112,MACCSFP115,MACCSFP116,MACCSFP121,MACCSFP122,MACCSFP123,MACCSFP126,MACCSFP135,MACCSFP147,MACCSFP148,MACCSFP155,MACCSFP157,MACCSFP158,MACCSFP162,MACCSFP163,MACCSFP165,PubchemFP14,PubchemFP17,PubchemFP19,PubchemFP21,PubchemFP23,PubchemFP33,PubchemFP37,PubchemFP129,PubchemFP146,PubchemFP149,PubchemFP157,PubchemFP160,PubchemFP182,PubchemFP186,PubchemFP189,PubchemFP190,PubchemFP192,PubchemFP193,PubchemFP199,PubchemFP206,PubchemFP213,PubchemFP217,PubchemFP227,PubchemFP229,PubchemFP230,PubchemFP258,PubchemFP261,PubchemFP297,PubchemFP331,PubchemFP332,PubchemFP339,PubchemFP340,PubchemFP346,PubchemFP349,PubchemFP351,PubchemFP353,PubchemFP355,PubchemFP358,PubchemFP363,PubchemFP364,PubchemFP366,PubchemFP367,PubchemFP376,PubchemFP380,PubchemFP386,PubchemFP390,PubchemFP391,PubchemFP392,PubchemFP394,PubchemFP401,PubchemFP403,PubchemFP404,PubchemFP406,PubchemFP407,PubchemFP412,PubchemFP414,PubchemFP416,PubchemFP418,PubchemFP420,PubchemFP425,PubchemFP431,PubchemFP437,PubchemFP442,PubchemFP443,PubchemFP450,PubchemFP451,PubchemFP452,PubchemFP454,PubchemFP466,PubchemFP470,PubchemFP471,PubchemFP472,PubchemFP475,PubchemFP478,PubchemFP480,PubchemFP482,PubchemFP483,PubchemFP485,PubchemFP486,PubchemFP487,PubchemFP489,PubchemFP494,PubchemFP495,PubchemFP499,PubchemFP503,PubchemFP507,PubchemFP509,PubchemFP521,PubchemFP523,PubchemFP529,PubchemFP535,PubchemFP536,PubchemFP537,PubchemFP538,PubchemFP539,PubchemFP545,PubchemFP551,PubchemFP554,PubchemFP555,PubchemFP559,PubchemFP567,PubchemFP568,PubchemFP574,PubchemFP575,PubchemFP577,PubchemFP581,PubchemFP586,PubchemFP587,PubchemFP591,PubchemFP596,PubchemFP602,PubchemFP603,PubchemFP607,PubchemFP608,PubchemFP610,PubchemFP614,PubchemFP615,PubchemFP618,PubchemFP620,PubchemFP623,PubchemFP628,PubchemFP629,PubchemFP630,PubchemFP637,PubchemFP639,PubchemFP641,PubchemFP650,PubchemFP654,PubchemFP655,PubchemFP657,PubchemFP661,PubchemFP665,PubchemFP668,PubchemFP673,PubchemFP675,PubchemFP677,PubchemFP680,PubchemFP682,PubchemFP683,PubchemFP684,PubchemFP686,PubchemFP688,PubchemFP689,PubchemFP705,PubchemFP710,PubchemFP713,PubchemFP721,PubchemFP722,PubchemFP724,PubchemFP730,PubchemFP738,PubchemFP740,PubchemFP741,PubchemFP747,PubchemFP749,PubchemFP756,PubchemFP758,PubchemFP760,PubchemFP761,PubchemFP762,PubchemFP763,PubchemFP764,PubchemFP772,PubchemFP773,PubchemFP779,PubchemFP785,PubchemFP789,PubchemFP790,PubchemFP802,PubchemFP806,PubchemFP808,PubchemFP810,PubchemFP811,PubchemFP813,PubchemFP814,PubchemFP817,PubchemFP819,PubchemFP824,PubchemFP825,PubchemFP827,PubchemFP831,PubchemFP835,PubchemFP845,PubchemFP860,PubchemFP861,PubchemFP872,PubchemFP878,SubFP2,SubFP12,SubFP16,SubFP21,SubFP32,SubFP52,SubFP57,SubFP62,SubFP74,SubFP86,SubFP99,SubFP100,SubFP103,SubFP135,SubFP143,SubFP150,SubFP171,SubFP174,SubFP181,SubFP183,SubFP184,SubFP201,SubFP212,SubFP229,SubFP235,SubFP246,SubFP278,SubFP279,SubFP280,SubFP281,SubFP282,SubFP286,SubFP287,SubFP303 |
|  | **SET03** | nAcid,ALogp2,AMR,nAromBond,nO,nF,nBr,nX,ATS0m,ATS7m,ATS6v,ATS7v,ATS8v,ATS6e,ATS7e,ATS8e,ATS1s,ATS2s,ATS5s,ATS8s,AATS3m,AATS8m,AATS0v,AATS4v,AATS7v,AATS2e,AATS3e,AATS4e,AATS6e,AATS7e,AATS8e,AATS6p,AATS7i,AATS0s,AATS1s,AATS6s,AATS7s,ATSC0c,ATSC2c,ATSC7c,ATSC4m,ATSC8m,ATSC0v,ATSC8v,ATSC0e,ATSC2e,ATSC6e,ATSC8e,ATSC1i,ATSC3i,ATSC4i,ATSC6i,ATSC7i,ATSC1s,ATSC2s,AATSC0c,AATSC4c,AATSC6c,AATSC7c,AATSC1m,AATSC2m,AATSC3m,AATSC4m,AATSC5m,AATSC6m,AATSC0v,AATSC4v,AATSC1e,AATSC2e,AATSC5e,AATSC7e,AATSC1p,AATSC3p,AATSC5p,AATSC7p,AATSC0i,AATSC1i,AATSC5i,AATSC7i,AATSC8i,AATSC1s,AATSC2s,AATSC6s,MATS3c,MATS5c,MATS6c,MATS4e,MATS1i,MATS2s,MATS4s,MATS6s,GATS1c,GATS3c,GATS4c,GATS6c,GATS8c,GATS1m,GATS7m,GATS8m,GATS2v,GATS4v,GATS7v,GATS8v,GATS2e,GATS5e,GATS3p,GATS2i,GATS3i,GATS4i,GATS5i,GATS6i,GATS8i,GATS2s,GATS3s,GATS5s,VR1_DzZ,SM1_Dzv,VE1_Dzv,VR3_Dzv,SM1_Dze,VE1_Dze,VR1_Dze,VR2_Dze,VR1_Dzp,VE3_Dzi,VR2_Dzi,SpAbs_Dzs,SM1_Dzs,nBase,BCUTw-1h,nBondsS2,nBondsD,nBondsM,SpMax1_Bhm,SpMax2_Bhm,SpMax3_Bhm,SpMax4_Bhm,SpMax5_Bhm,SpMax8_Bhm,SpMin1_Bhm,SpMin4_Bhm,SpMin5_Bhm,SpMax8_Bhv,SpMin3_Bhv,SpMin7_Bhv,SpMax2_Bhe,SpMax3_Bhe,SpMin2_Bhe,SpMin3_Bhe,SpMin4_Bhe,SpMin8_Bhe,SpMax3_Bhp,SpMin2_Bhp,SpMin4_Bhp,SpMax3_Bhi,SpMax4_Bhi,SpMin1_Bhi,SpMin2_Bhi,SpMax6_Bhs,SpMin7_Bhs,C1SP1,C2SP1,C1SP2,C2SP2,C3SP2,C3SP3,SCH-4,SCH-6,SCH-7,VCH-5,VCH-6,SC-3,SC-5,VC-3,VC-5,SPC-4,SPC-5,VPC-4,VPC-5,SP-2,ASP-2,ASP-4,ASP-6,VP-0,VP-1,VP-3,AVP-4,AVP-7,CrippenLogP,CrippenMR,SpMax_Dt,SpMAD_Dt,VE2_Dt,ECCEN,nHBd,nwHBa,nHBint4,nHBint10,nHsNH3p,nHsssNHp,nHdCH2,nHaaCH,nHCsatu,nsCH3,nssCH2,nsssCH,ntsC,nssNH,nsssN,ndO,nsSH,naaS,ndssS,nsCl,SHBd,SwHBd,SHBa,SHBint4,SHsSH,SHsNH3p,SHCsatu,SssCH2,SaaCH,SsssCH,SsNH3p,SsssNHp,SdsN,SaaN,SdS,SaaS,SsBr,minHBd,minwHBa,minHBint2,minHBint3,minHBint5,minHBint6,minHBint8,minHssNH,minHaaNH,minHCHnX,minHCsatu,minHAvin,minssCH2,minaaCH,mintsC,minaasC,mindsN,minaasN,minsOH,mindO,minaaO,minsOm,minsF,maxHBd,maxHBa,maxwHBa,maxHBint5,maxHsOH,maxHsSH,maxHssNH,maxHCsatu,maxsCH3,maxdCH2,maxtCH,maxaaCH,maxsssCH,maxaaaC,maxtN,maxdsN,maxdO,maxssO,maxaaO,gmin,LipoaffinityIndex,MAXDP,ETA_dEpsilon_D,ETA_dPsi_B,ETA_Shape_X,ETA_dBetaP,ETA_Eta_L,ETA_EtaP_L,ETA_EtaP_F_L,ETA_Eta_B,ETA_Eta_B_RC,ETA_EtaP_B_RC,fragC,HybRatio,IC0,IC2,TIC1,SIC4,BIC3,BIC4,MIC0,MIC1,MIC3,ZMIC0,ZMIC2,nAtomP,MDEC-22,MDEC-23,MDEO-12,MDEO-22,MDEN-13,MLFER_E,piPC2,piPC3,piPC8,n3Ring,n4Ring,n5Ring,n7Ring,nFRing,nF6Ring,nF11Ring,nF12Ring,nFG12Ring,nTRing,nT11Ring,nT12Ring,nTG12Ring,n5HeteroRing,nT7HeteroRing,nT9HeteroRing,nT11HeteroRing,nT12HeteroRing,RotBtFrac,LipinskiFailures,GGI2,GGI4,GGI5,GGI7,JGI1,JGI2,JGI3,JGI5,JGI6,JGI10,VE1_D,VR1_D,VR3_D,MWC6,SRW5,SRW9,MW,XLogP,MACCSFP11,MACCSFP19,MACCSFP23,MACCSFP26,MACCSFP29,MACCSFP30,MACCSFP32,MACCSFP33,MACCSFP37,MACCSFP39,MACCSFP47,MACCSFP57,MACCSFP61,MACCSFP64,MACCSFP65,MACCSFP71,MACCSFP75,MACCSFP76,MACCSFP79,MACCSFP86,MACCSFP88,MACCSFP91,MACCSFP94,MACCSFP95,MACCSFP100,MACCSFP101,MACCSFP103,MACCSFP106,MACCSFP116,MACCSFP121,MACCSFP122,MACCSFP126,MACCSFP130,MACCSFP135,MACCSFP141,MACCSFP148,MACCSFP149,MACCSFP156,MACCSFP158,MACCSFP162,MACCSFP165,PubchemFP1,PubchemFP2,PubchemFP14,PubchemFP15,PubchemFP16,PubchemFP17,PubchemFP19,PubchemFP21,PubchemFP34,PubchemFP37,PubchemFP43,PubchemFP46,PubchemFP146,PubchemFP149,PubchemFP150,PubchemFP157,PubchemFP180,PubchemFP186,PubchemFP190,PubchemFP191,PubchemFP192,PubchemFP193,PubchemFP199,PubchemFP200,PubchemFP213,PubchemFP214,PubchemFP217,PubchemFP227,PubchemFP229,PubchemFP234,PubchemFP258,PubchemFP259,PubchemFP261,PubchemFP286,PubchemFP293,PubchemFP297,PubchemFP329,PubchemFP331,PubchemFP332,PubchemFP340,PubchemFP346,PubchemFP348,PubchemFP349,PubchemFP352,PubchemFP353,PubchemFP355,PubchemFP358,PubchemFP362,PubchemFP363,PubchemFP364,PubchemFP366,PubchemFP375,PubchemFP376,PubchemFP379,PubchemFP380,PubchemFP386,PubchemFP390,PubchemFP391,PubchemFP401,PubchemFP406,PubchemFP412,PubchemFP413,PubchemFP414,PubchemFP416,PubchemFP418,PubchemFP420,PubchemFP425,PubchemFP431,PubchemFP432,PubchemFP434,PubchemFP437,PubchemFP442,PubchemFP443,PubchemFP450,PubchemFP454,PubchemFP457,PubchemFP468,PubchemFP474,PubchemFP475,PubchemFP482,PubchemFP485,PubchemFP487,PubchemFP491,PubchemFP494,PubchemFP495,PubchemFP499,PubchemFP503,PubchemFP507,PubchemFP508,PubchemFP520,PubchemFP524,PubchemFP529,PubchemFP530,PubchemFP531,PubchemFP535,PubchemFP536,PubchemFP537,PubchemFP542,PubchemFP547,PubchemFP549,PubchemFP554,PubchemFP555,PubchemFP556,PubchemFP570,PubchemFP575,PubchemFP577,PubchemFP581,PubchemFP585,PubchemFP586,PubchemFP587,PubchemFP591,PubchemFP598,PubchemFP602,PubchemFP605,PubchemFP606,PubchemFP608,PubchemFP609,PubchemFP614,PubchemFP615,PubchemFP620,PubchemFP624,PubchemFP628,PubchemFP632,PubchemFP636,PubchemFP637,PubchemFP638,PubchemFP639,PubchemFP641,PubchemFP650,PubchemFP653,PubchemFP655,PubchemFP658,PubchemFP660,PubchemFP661,PubchemFP665,PubchemFP671,PubchemFP673,PubchemFP675,PubchemFP677,PubchemFP678,PubchemFP679,PubchemFP681,PubchemFP682,PubchemFP688,PubchemFP689,PubchemFP694,PubchemFP697,PubchemFP698,PubchemFP701,PubchemFP705,PubchemFP713,PubchemFP717,PubchemFP721,PubchemFP722,PubchemFP738,PubchemFP741,PubchemFP748,PubchemFP749,PubchemFP751,PubchemFP760,PubchemFP773,PubchemFP774,PubchemFP779,PubchemFP782,PubchemFP785,PubchemFP788,PubchemFP789,PubchemFP790,PubchemFP791,PubchemFP798,PubchemFP802,PubchemFP810,PubchemFP813,PubchemFP814,PubchemFP816,PubchemFP819,PubchemFP822,PubchemFP825,PubchemFP827,PubchemFP837,PubchemFP860,PubchemFP861,PubchemFP872,SubFP2,SubFP12,SubFP15,SubFP28,SubFP35,SubFP52,SubFP86,SubFP103,SubFP135,SubFP143,SubFP150,SubFP153,SubFP172,SubFP174,SubFP181,SubFP183,SubFP188,SubFP201,SubFP209,SubFP212,SubFP229,SubFP246,SubFP274,SubFP279,SubFP284,SubFP287,SubFP294,SubFP298,SubFP303 |
|  | **SET04** | nAcid,ALogP,AMR,naAromAtom,nH,nC,nN,nO,nCl,ATS2m,ATS3m,ATS4m,ATS5m,ATS8m,ATS6v,ATS8v,ATS1e,ATS7e,ATS8e,ATS0s,ATS1s,ATS2s,ATS3s,ATS4s,ATS7s,AATS0m,AATS1m,AATS2m,AATS3m,AATS7m,AATS8m,AATS0v,AATS7v,AATS8v,AATS1e,AATS2e,AATS3e,nI,AATS5e,AATS7e,AATS0p,AATS6p,AATS8p,AATS7i,AATS4s,AATS8s,ATSC3c,ATSC7c,ATSC4m,ATSC6m,ATSC7m,ATSC0v,ATSC8v,ATSC0e,ATSC3e,ATSC4e,ATSC3i,AATSC1m,AATSC3m,AATSC5m,AATSC7m,AATSC1v,AATSC4v,AATSC2e,AATSC3e,AATSC5e,AATSC6e,AATSC4p,AATSC6p,AATSC1i,AATSC2i,AATSC3i,AATSC4i,AATSC5i,AATSC6i,AATSC8i,AATSC1s,AATSC2s,AATSC5s,MATS1c,MATS4c,MATS7c,MATS8m,MATS1i,MATS3i,MATS5i,MATS2s,MATS4s,MATS6s,MATS7s,GATS1c,GATS3c,GATS4c,GATS6c,GATS7c,GATS1m,GATS4v,GATS8v,GATS2e,GATS5e,GATS8p,GATS4i,GATS6i,GATS7i,GATS8i,GATS1s,GATS2s,GATS3s,GATS4s,GATS5s,GATS6s,GATS7s,SM1_DzZ,SpDiam_Dzv,SM1_Dzv,VE1_Dzv,SpMAD_Dze,SM1_Dze,VR1_Dze,VR2_Dze,VR1_Dzp,VE2_Dzi,VE3_Dzi,VR2_Dzi,SpAbs_Dzs,SM1_Dzs,nBase,BCUTw-1l,BCUTw-1h,BCUTp-1h,nBondsS2,nBondsS3,nBondsD,nBondsM,SpMax1_Bhm,SpMax3_Bhm,SpMax4_Bhm,SpMax5_Bhm,SpMax6_Bhm,SpMin4_Bhm,SpMin5_Bhm,SpMin7_Bhm,SpMax4_Bhv,SpMax6_Bhv,SpMax7_Bhv,SpMax8_Bhv,SpMin7_Bhv,SpMax2_Bhe,SpMax3_Bhe,SpMax7_Bhe,SpMax8_Bhe,SpMin8_Bhe,SpMin3_Bhp,SpMax1_Bhi,SpMax3_Bhi,SpMax7_Bhi,SpMin2_Bhi,SpMax3_Bhs,SpMax5_Bhs,SpMin1_Bhs,SpMin4_Bhs,SpMin5_Bhs,C1SP2,C2SP2,C3SP2,C3SP3,SCH-4,SCH-5,SCH-6,SCH-7,VCH-5,VCH-6,VCH-7,SC-3,SC-4,SC-5,VC-3,VC-5,SPC-4,SP-2,SP-5,ASP-0,ASP-1,ASP-4,ASP-5,VP-0,VP-1,VP-5,AVP-1,AVP-7,CrippenLogP,CrippenMR,SpMax_Dt,VE1_Dt,ECCEN,nHBint3,nHBint4,nHBint5,nHBint9,nHsNH3p,nHsssNHp,nHdCH2,nHCHnX,nHCsatu,nHother,ndCH2,naasC,nssNH,nsssNHp,naaN,ndO,nsSH,ndssS,SHBd,SHBint3,SHBint6,SHBint9,SHBint10,SHsOH,SHtCH,SHdsCH,SHother,SsssCH,StsC,SaaaC,SsNH3p,StN,SsssNHp,SaaN,SdS,SaaS,SddssS,SsCl,minHBd,minHBa,minwHBa,minHBint2,minHBint5,minHBint8,minHsSH,minHaaNH,minHsssNHp,minHtCH,minHdsCH,minHCHnX,minHCsats,minHCsatu,mintCH,minsssCH,mintsC,minaaaC,minaaN,minaasN,minssssNp,mindO,minssO,minsF,mindsssP,mindS,minaaS,maxHBd,maxHBa,maxwHBa,maxHBint3,maxHBint5,maxHBint9,maxHsOH,maxHdCH2,maxHaaCH,maxsCH3,maxdsCH,maxaaCH,maxsssCH,maxaasC,maxsNH3p,maxtN,maxssO,maxsSH,maxsI,hmax,MAXDN,DELS,ETA_dEpsilon_B,ETA_dEpsilon_D,ETA_dPsi_B,ETA_BetaP_s,ETA_BetaP_ns,ETA_dBeta,ETA_dBetaP,ETA_BetaP_ns_d,ETA_Eta_F,ETA_EtaP_F_L,ETA_Eta_B_RC,ETA_EtaP_B_RC,fragC,nHBAcc_Lipinski,nHBDon_Lipinski,HybRatio,IC2,IC4,TIC2,SIC0,SIC1,SIC4,MIC0,MIC1,ZMIC0,ZMIC2,Kier2,MLogP,MDEC-13,MDEC-14,MDEC-23,MDEC-33,MDEO-12,MDEN-13,MDEN-23,MLFER_L,piPC2,piPC8,PetitjeanNumber,nFRing,nF6Ring,nF9Ring,nF10Ring,nF11Ring,nFG12Ring,nT9Ring,nT12Ring,nTG12Ring,nHeteroRing,n5HeteroRing,nF9HeteroRing,nF12HeteroRing,nT6HeteroRing,nT9HeteroRing,nT11HeteroRing,nT12HeteroRing,nRotB,GGI2,GGI3,GGI4,GGI5,GGI7,GGI8,JGI2,JGI3,JGI7,SpMAD_D,VE1_D,VE2_D,VR1_D,VR2_D,VR3_D,TopoPSA,SRW7,WTPT-2,WTPT-5,XLogP,MACCSFP11,MACCSFP16,MACCSFP21,MACCSFP23,MACCSFP29,MACCSFP40,MACCSFP42,MACCSFP52,MACCSFP55,MACCSFP56,MACCSFP57,MACCSFP58,MACCSFP64,MACCSFP65,MACCSFP69,MACCSFP76,MACCSFP79,MACCSFP83,MACCSFP88,MACCSFP89,MACCSFP91,MACCSFP95,MACCSFP96,MACCSFP97,MACCSFP103,MACCSFP106,MACCSFP107,MACCSFP110,MACCSFP118,MACCSFP120,MACCSFP121,MACCSFP123,MACCSFP126,MACCSFP128,MACCSFP136,MACCSFP137,MACCSFP144,MACCSFP148,MACCSFP154,MACCSFP155,MACCSFP158,MACCSFP163,MACCSFP165,PubchemFP2,PubchemFP12,PubchemFP17,PubchemFP21,PubchemFP34,PubchemFP37,PubchemFP43,PubchemFP44,PubchemFP45,PubchemFP117,PubchemFP145,PubchemFP146,PubchemFP148,PubchemFP150,PubchemFP160,PubchemFP164,PubchemFP180,PubchemFP184,PubchemFP189,PubchemFP190,PubchemFP191,PubchemFP192,PubchemFP193,PubchemFP195,PubchemFP199,PubchemFP213,PubchemFP217,PubchemFP219,PubchemFP229,PubchemFP258,PubchemFP261,PubchemFP286,PubchemFP294,PubchemFP297,PubchemFP300,PubchemFP305,PubchemFP329,PubchemFP330,PubchemFP332,PubchemFP335,PubchemFP337,PubchemFP339,PubchemFP340,PubchemFP341,PubchemFP350,PubchemFP352,PubchemFP353,PubchemFP358,PubchemFP361,PubchemFP363,PubchemFP364,PubchemFP368,PubchemFP371,PubchemFP372,PubchemFP374,PubchemFP376,PubchemFP380,PubchemFP382,PubchemFP386,PubchemFP389,PubchemFP391,PubchemFP392,PubchemFP393,PubchemFP396,PubchemFP397,PubchemFP399,PubchemFP407,PubchemFP412,PubchemFP418,PubchemFP420,PubchemFP421,PubchemFP425,PubchemFP431,PubchemFP437,PubchemFP445,PubchemFP450,PubchemFP452,PubchemFP455,PubchemFP456,PubchemFP458,PubchemFP464,PubchemFP465,PubchemFP471,PubchemFP473,PubchemFP474,PubchemFP475,PubchemFP476,PubchemFP482,PubchemFP486,PubchemFP488,PubchemFP489,PubchemFP491,PubchemFP495,PubchemFP499,PubchemFP500,PubchemFP503,PubchemFP506,PubchemFP507,PubchemFP508,PubchemFP509,PubchemFP515,PubchemFP523,PubchemFP531,PubchemFP533,PubchemFP540,PubchemFP542,PubchemFP544,PubchemFP546,PubchemFP548,PubchemFP551,PubchemFP552,PubchemFP562,PubchemFP563,PubchemFP566,PubchemFP568,PubchemFP571,PubchemFP575,PubchemFP576,PubchemFP581,PubchemFP585,PubchemFP591,PubchemFP596,PubchemFP602,PubchemFP606,PubchemFP608,PubchemFP610,PubchemFP611,PubchemFP614,PubchemFP615,PubchemFP628,PubchemFP636,PubchemFP647,PubchemFP650,PubchemFP654,PubchemFP656,PubchemFP657,PubchemFP658,PubchemFP660,PubchemFP665,PubchemFP671,PubchemFP672,PubchemFP675,PubchemFP677,PubchemFP679,PubchemFP684,PubchemFP688,PubchemFP689,PubchemFP690,PubchemFP698,PubchemFP704,PubchemFP705,PubchemFP708,PubchemFP713,PubchemFP714,PubchemFP715,PubchemFP716,PubchemFP721,PubchemFP730,PubchemFP736,PubchemFP740,PubchemFP749,PubchemFP760,PubchemFP771,PubchemFP772,PubchemFP773,PubchemFP779,PubchemFP781,PubchemFP783,PubchemFP793,PubchemFP798,PubchemFP802,PubchemFP805,PubchemFP810,PubchemFP813,PubchemFP814,PubchemFP816,PubchemFP828,PubchemFP831,PubchemFP840,PubchemFP843,PubchemFP845,PubchemFP860,PubchemFP872,SubFP16,SubFP32,SubFP62,SubFP88,SubFP96,SubFP120,SubFP135,SubFP139,SubFP150,SubFP153,SubFP166,SubFP171,SubFP180,SubFP183,SubFP188,SubFP200,SubFP212,SubFP246,SubFP274,SubFP279,SubFP281,SubFP282,SubFP286,SubFP294,SubFP299 |
|  | **SET05** | nAcid,nAromBond,nH,nC,nO,nX,ATS2m,ATS3m,ATS5m,ATS7m,ATS7v,ATS8v,ATS1e,ATS3e,ATS6e,ATS7e,ATS8e,ATS0p,ATS1s,ATS2s,ATS5s,AATS2m,AATS5m,AATS8m,AATS0v,AATS3v,AATS7v,AATS1e,AATS2e,AATS4e,AATS6e,AATS7e,AATS8e,AATS0p,AATS6p,AATS0i,AATS0s,AATS3s,AATS4s,AATS7s,ATSC7c,ATSC8c,ATSC4m,ATSC6m,ATSC8m,ATSC0v,ATSC1v,ATSC0e,ATSC2e,ATSC4e,ATSC6e,ATSC8e,ATSC0p,ATSC2p,ATSC7p,ATSC8p,ATSC0i,ATSC2i,ATSC3i,ATSC4i,ATSC8s,AATSC2c,AATSC1m,AATSC3m,AATSC6m,AATSC0v,AATSC4v,AATSC7v,AATSC2e,AATSC3e,AATSC7e,AATSC0p,AATSC5p,AATSC1i,AATSC3i,AATSC5i,AATSC6i,AATSC7i,AATSC1s,AATSC2s,AATSC4s,AATSC5s,AATSC6s,AATSC8s,MATS1c,MATS2c,MATS5c,MATS6c,MATS4m,MATS6m,MATS8m,MATS5e,MATS6e,MATS1i,MATS5i,MATS1s,MATS2s,MATS6s,MATS7s,GATS1c,GATS1m,GATS2m,GATS4m,GATS7m,GATS2v,GATS4v,GATS8v,GATS5e,GATS8p,GATS3i,GATS6i,GATS8i,GATS1s,GATS5s,GATS7s,SpMAD_DzZ,SM1_DzZ,VR1_DzZ,VR3_Dzv,SpMax_Dze,SpMAD_Dze,SM1_Dze,VR1_Dze,VR3_Dze,VE2_Dzp,VR1_Dzp,VR1_Dzi,SpAbs_Dzs,SM1_Dzs,VE2_Dzs,VR1_Dzs,nBase,BCUTw-1h,BCUTp-1l,nBondsS2,nBondsM,SpMax4_Bhm,SpMax5_Bhm,SpMax7_Bhm,SpMin1_Bhm,SpMin2_Bhm,SpMin4_Bhm,SpMax1_Bhv,SpMax8_Bhv,SpMin1_Bhv,SpMin4_Bhv,SpMin7_Bhv,SpMin8_Bhv,SpMax2_Bhe,SpMax5_Bhe,SpMin3_Bhe,SpMin4_Bhe,SpMin8_Bhe,SpMax3_Bhp,SpMin2_Bhp,SpMin7_Bhp,SpMax1_Bhi,SpMax3_Bhi,SpMin1_Bhi,SpMax3_Bhs,SpMax5_Bhs,SpMin5_Bhs,C1SP2,C2SP2,C3SP2,C2SP3,C3SP3,SCH-4,SCH-5,SCH-7,VCH-5,VCH-6,VCH-7,SC-4,SC-5,VC-5,VPC-4,SP-2,ASP-2,ASP-4,VP-1,VP-5,AVP-1,AVP-3,CrippenLogP,CrippenMR,VE1_Dt,VE2_Dt,ECCEN,nwHBa,nHBint3,nHBint5,nHBint10,nHsOH,nHsNH3p,nHsssNHp,nHdCH2,nHaaCH,nsCH3,naaCH,naasC,nsNH3p,naaN,ndO,nsSH,ndssS,SHBd,SwHBd,SHBa,SHBint5,SHBint7,SHssNH,SHCsatu,SHAvin,SdCH2,SaaCH,SsssCH,SaaaC,SssNH,SsssNHp,SdsN,SaaN,SsOH,SdO,SsF,SdsssP,SdS,SaaS,SsBr,minwHBa,minHBint6,minHBint8,minHssNH,minHsNH3p,minHdsCH,minHaaCH,minHCsatu,minsssCH,mintsC,minaasC,minaaaC,minssNH,minaasN,mindO,minsOm,minsF,mindsssP,mindS,minsI,maxHBa,maxwHBa,maxHBint10,maxHsOH,maxHssNH,maxHsssNHp,maxHCHnX,maxHCsatu,maxsCH3,maxssCH2,maxtCH,maxdsCH,maxaaCH,maxaaaC,maxsNH3p,maxsNH2,maxssNH,maxtN,maxsOH,maxdO,maxssO,maxaaO,maxsBr,maxsI,gmin,LipoaffinityIndex,ETA_Epsilon_4,ETA_dEpsilon_D,ETA_dPsi_B,ETA_Shape_X,ETA_Eta_F,ETA_EtaP_F,ETA_EtaP_F_L,ETA_Eta_B,ETA_Eta_B_RC,ETA_EtaP_B_RC,fragC,nHBAcc_Lipinski,nHBDon_Lipinski,HybRatio,IC0,IC1,IC4,TIC0,SIC4,BIC2,BIC3,BIC4,BIC5,MIC0,MIC1,ZMIC0,ZMIC1,Kier3,nAtomP,MDEC-11,MDEO-12,MDEN-13,MDEN-23,MLFER_E,PetitjeanNumber,n6Ring,nF6Ring,nT12Ring,nTG12Ring,n5HeteroRing,nF8HeteroRing,nF9HeteroRing,nT6HeteroRing,nT9HeteroRing,nT11HeteroRing,nT12HeteroRing,GGI1,GGI3,GGI5,GGI8,JGI1,JGI2,JGI3,JGI5,JGI6,JGI8,JGI10,VE2_D,VR1_D,VR2_D,VR3_D,MW,WTPT-2,WTPT-5,XLogP,MACCSFP11,MACCSFP33,MACCSFP36,MACCSFP37,MACCSFP42,MACCSFP47,MACCSFP48,MACCSFP50,MACCSFP55,MACCSFP57,MACCSFP58,MACCSFP59,MACCSFP60,MACCSFP62,MACCSFP63,MACCSFP64,MACCSFP65,MACCSFP69,MACCSFP75,MACCSFP76,MACCSFP79,MACCSFP81,MACCSFP83,MACCSFP84,MACCSFP88,MACCSFP89,MACCSFP91,MACCSFP95,MACCSFP96,MACCSFP101,MACCSFP108,MACCSFP111,MACCSFP115,MACCSFP116,MACCSFP118,MACCSFP120,MACCSFP121,MACCSFP123,MACCSFP126,MACCSFP135,MACCSFP141,MACCSFP145,MACCSFP147,MACCSFP156,MACCSFP165,PubchemFP1,PubchemFP3,PubchemFP11,PubchemFP12,PubchemFP13,PubchemFP17,PubchemFP18,PubchemFP19,PubchemFP23,PubchemFP24,PubchemFP37,PubchemFP131,PubchemFP146,PubchemFP147,PubchemFP148,PubchemFP152,PubchemFP153,PubchemFP160,PubchemFP180,PubchemFP182,PubchemFP189,PubchemFP190,PubchemFP191,PubchemFP192,PubchemFP199,PubchemFP200,PubchemFP213,PubchemFP217,PubchemFP229,PubchemFP258,PubchemFP286,PubchemFP287,PubchemFP293,PubchemFP297,PubchemFP329,PubchemFP331,PubchemFP337,PubchemFP341,PubchemFP345,PubchemFP346,PubchemFP349,PubchemFP353,PubchemFP355,PubchemFP358,PubchemFP360,PubchemFP362,PubchemFP364,PubchemFP371,PubchemFP374,PubchemFP375,PubchemFP376,PubchemFP379,PubchemFP380,PubchemFP386,PubchemFP389,PubchemFP390,PubchemFP391,PubchemFP392,PubchemFP403,PubchemFP406,PubchemFP412,PubchemFP413,PubchemFP416,PubchemFP418,PubchemFP420,PubchemFP425,PubchemFP432,PubchemFP434,PubchemFP437,PubchemFP441,PubchemFP445,PubchemFP446,PubchemFP447,PubchemFP450,PubchemFP464,PubchemFP466,PubchemFP470,PubchemFP471,PubchemFP472,PubchemFP474,PubchemFP476,PubchemFP482,PubchemFP489,PubchemFP491,PubchemFP502,PubchemFP503,PubchemFP507,PubchemFP508,PubchemFP509,PubchemFP523,PubchemFP531,PubchemFP532,PubchemFP533,PubchemFP536,PubchemFP537,PubchemFP543,PubchemFP549,PubchemFP551,PubchemFP554,PubchemFP555,PubchemFP563,PubchemFP565,PubchemFP566,PubchemFP568,PubchemFP569,PubchemFP575,PubchemFP576,PubchemFP581,PubchemFP585,PubchemFP586,PubchemFP587,PubchemFP591,PubchemFP596,PubchemFP602,PubchemFP607,PubchemFP608,PubchemFP609,PubchemFP610,PubchemFP612,PubchemFP620,PubchemFP622,PubchemFP628,PubchemFP630,PubchemFP635,PubchemFP638,PubchemFP645,PubchemFP650,PubchemFP654,PubchemFP655,PubchemFP657,PubchemFP665,PubchemFP673,PubchemFP675,PubchemFP677,PubchemFP678,PubchemFP679,PubchemFP682,PubchemFP684,PubchemFP686,PubchemFP688,PubchemFP697,PubchemFP702,PubchemFP703,PubchemFP706,PubchemFP710,PubchemFP713,PubchemFP714,PubchemFP721,PubchemFP740,PubchemFP741,PubchemFP749,PubchemFP751,PubchemFP753,PubchemFP754,PubchemFP756,PubchemFP759,PubchemFP768,PubchemFP773,PubchemFP774,PubchemFP782,PubchemFP789,PubchemFP791,PubchemFP793,PubchemFP798,PubchemFP810,PubchemFP813,PubchemFP814,PubchemFP820,PubchemFP825,PubchemFP832,PubchemFP835,PubchemFP836,PubchemFP839,PubchemFP844,PubchemFP845,PubchemFP860,PubchemFP877,SubFP5,SubFP12,SubFP17,SubFP21,SubFP28,SubFP37,SubFP52,SubFP57,SubFP62,SubFP75,SubFP150,SubFP171,SubFP172,SubFP180,SubFP181,SubFP183,SubFP184,SubFP201,SubFP212,SubFP229,SubFP246,SubFP278,SubFP279,SubFP280,SubFP281,SubFP283,SubFP294,SubFP296,SubFP303 |
| SSFS | **SET01** | AATSC4p,AATSC2s,SpMax1_Bhm,nHBint5,SwHBa,SssO,minHBa,minHBint2,maxHBint2,maxdO,gmin,ZMIC3,MLFER_A,  R_TpiPCTPC,nT6Ring,n4HeteroRing,VR1_D,WTPT-4,WTPT-5,MACCSFP95,MACCSFP106,PubchemFP331,PubchemFP376,  PubchemFP418,PubchemFP583,PubchemFP609,PubchemFP730,SubFP150,SubFP288 |
|  | **SET02** | ATSC4v,AATSC8i,GATS7c,GATS5s,VR1_Dze,SpMax1_Bhm,SpMin1_Bhe,nHBint5,minHBa,maxHBint2,maxdO,MLFER_A,  R_TpiPCTPC,MACCSFP72,MACCSFP95,MACCSFP106,PubchemFP376,PubchemFP461,PubchemFP468,PubchemFP609,  PubchemFP730,PubchemFP747,SubFP62 |
|  | **SET03** | AATSC3m,AATSC4p,AATSC8i,GATS3c,GATS7c,SpMax1_Bhm,SpMax2_Bhp,SwHBa,minHBa,minHBint2,minHBint5,maxdO,  R_TpiPCTPC,nRotB,TopoPSA,MACCSFP72,MACCSFP95,MACCSFP106,PubchemFP331,PubchemFP376,PubchemFP438,  PubchemFP583,PubchemFP802,SubFP52 |
|  | **SET04** | ATSC1c,ATSC3c,ATSC8c,AATSC4v,AATSC8p,AATSC3i,AATSC2s,SpMax1_Bhm,nHBint5,minHBd,minHBint2,maxHBint2,  maxaaN,gmin,ETA_BetaP,MIC0,R_TpiPCTPC,GGI2,WTPT-5,MACCSFP47,MACCSFP77,MACCSFP97,PubchemFP450,  PubchemFP500,PubchemFP774,PubchemFP830,PubchemFP841,SubFP150 |
|  | **SET05** | ALogP,AATS8s,ATSC7c,ATSC1v,AATSC4m,AATSC5m,AATSC8i,AATSC2s,MATS5c,MATS8s,GATS2c,VR3_Dzv,VE3_Dzi,  SpMax1_Bhm,SPC-4,ASP-0,nHBint5,SHBint2,SsOH,minHBa,maxwHBa,maxHBint2,maxdsCH,maxaaN,maxdO,hmin,gmin,  ETA_dEpsilon_D,IC1,IC2,ZMIC4,nAtomP,MDEC-33,MLFER_BH,R_TpiPCTPC,n6Ring,nTRing,JGI2,TopoPSA,MACCSFP95,  MACCSFP97,MACCSFP106,MACCSFP148,PubchemFP418,PubchemFP449,PubchemFP499,PubchemFP699 |

**Table S3.** 2D structures of 46 hits identified by top 8 models.

| **Molecule Index** | **2D structure** | **Molecule Index** | **2D structure** | **Molecule Index** | **2D structure** |
| --- | --- | --- | --- | --- | --- |
| **1** | 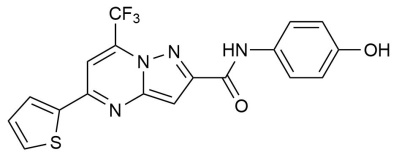 | **17** | 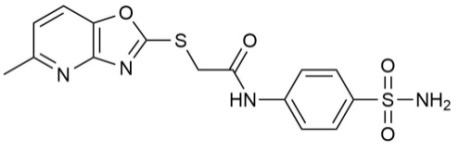 | **33** | 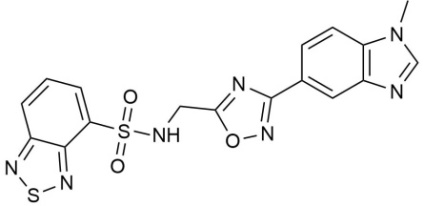 |
| **2** | 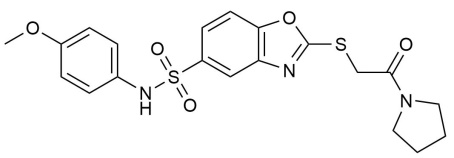 | **18** | 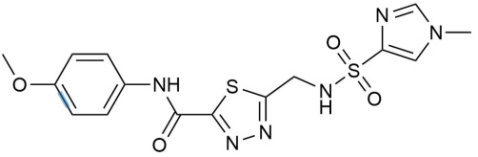 | **34** | 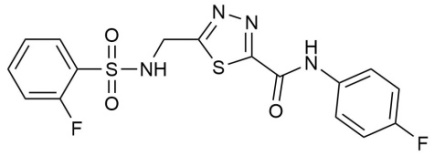 |
| **3** | 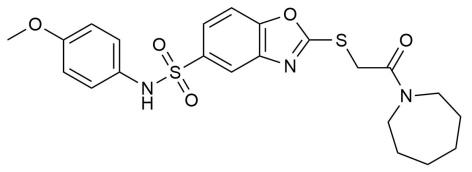 | **19** | 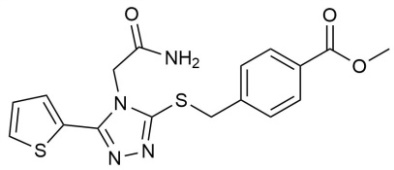 | **35** | 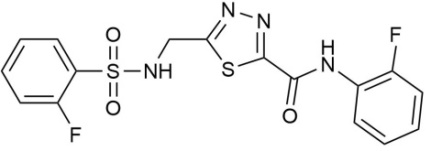 |
| **4** | 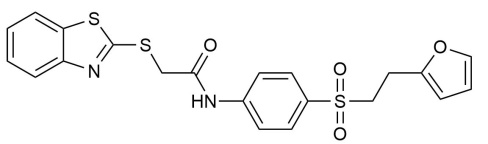 | **20** | 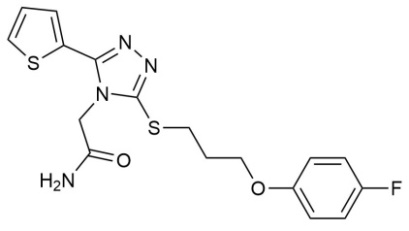 | **36** | 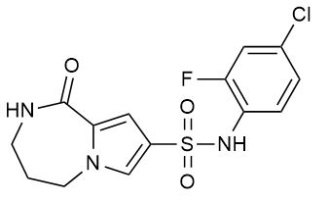 |
| **5** | 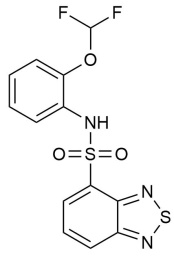 | **21** | 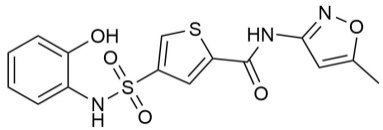 | **37** | 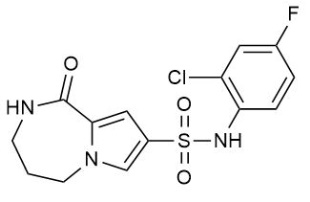 |
| **6** | 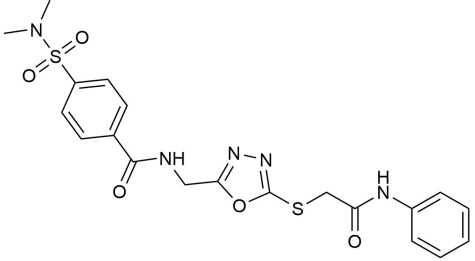 | **22** | 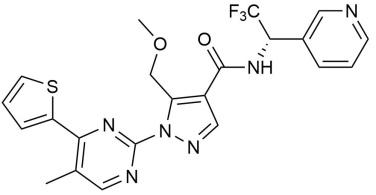 | **38** | 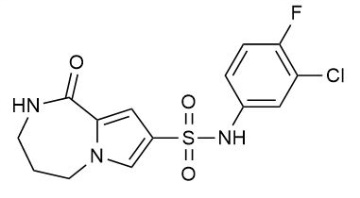 |
| **7** | 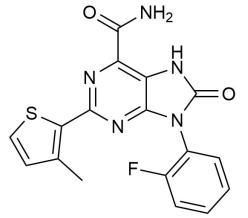 | **23** | 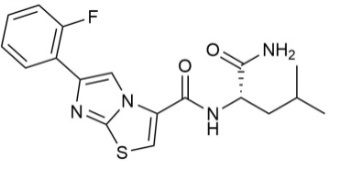 | **39** | 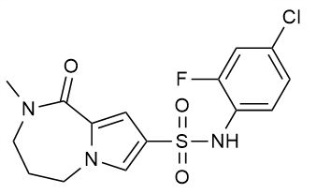 |
| **8** | 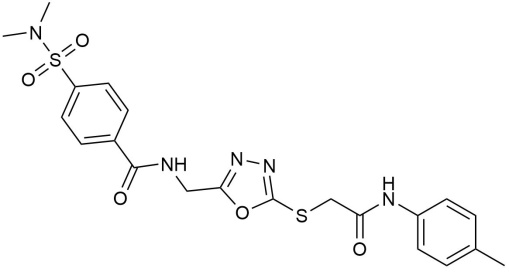 | **24** | 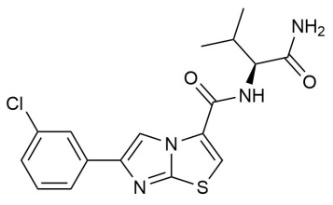 | **40** | 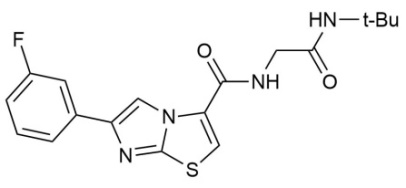 |
| **9** | 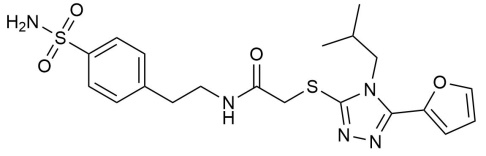 | **25** | 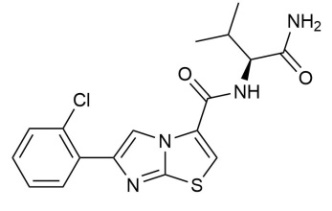 | **41** | 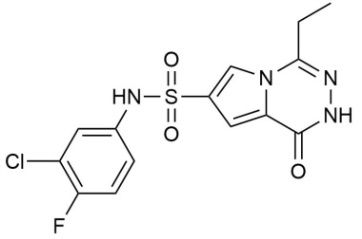 |
| **10** | 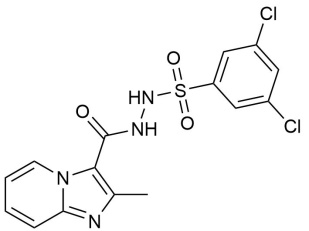 | **26** | 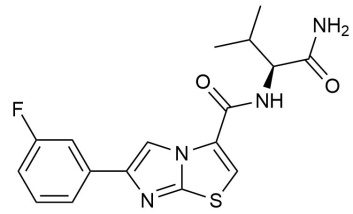 | **42** | 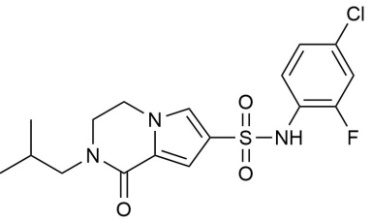 |
| **11** | 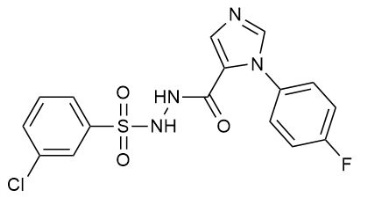 | **27** | 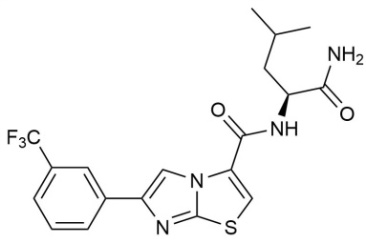 | **43** | 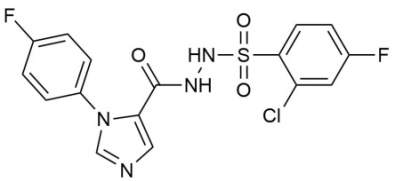 |
| **12** | 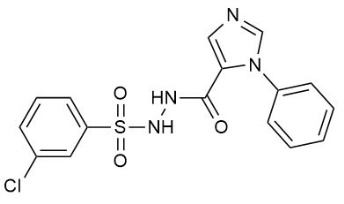 | **28** | 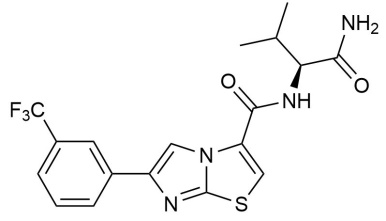 | **44** | 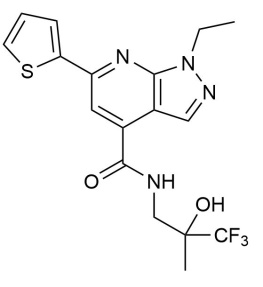 |
| **13** | 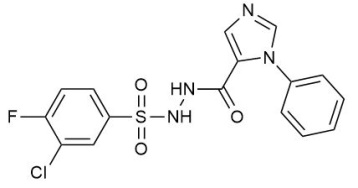 | **29** | 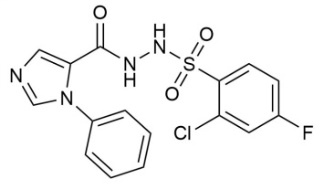 | **45** | 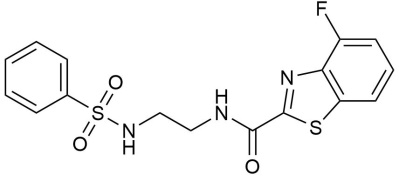 |
| **14** | 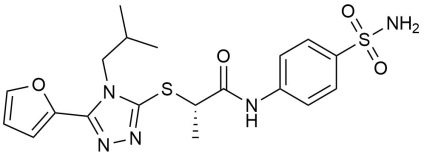 | **30** | 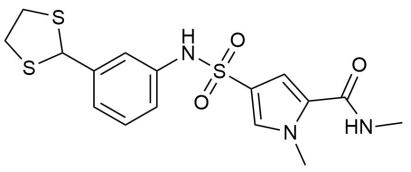 | **46** | 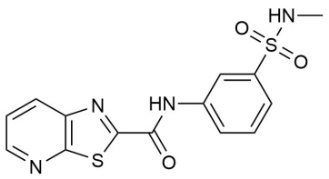 |
| **15** | 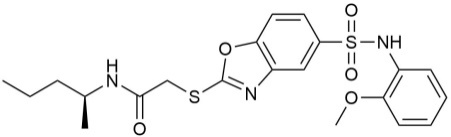 | **31** | 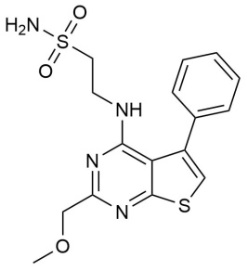 |  |  |
| **16** | 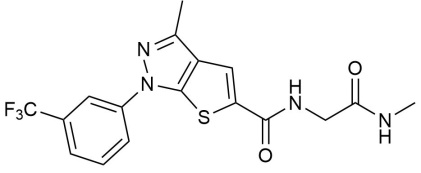 | **32** | 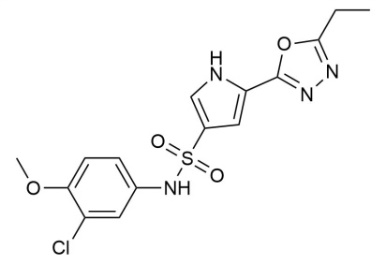 |  |  |

**Figure S1.** Docking pose of representative compound **67** among 266 known S100A9 inhibitors of Table S1: (a) 2D-map of the non-covalent interaction between S100A9 homodimer and compound **67**, (b) 3D-complext of S100A9 homodimer and compound **67.**


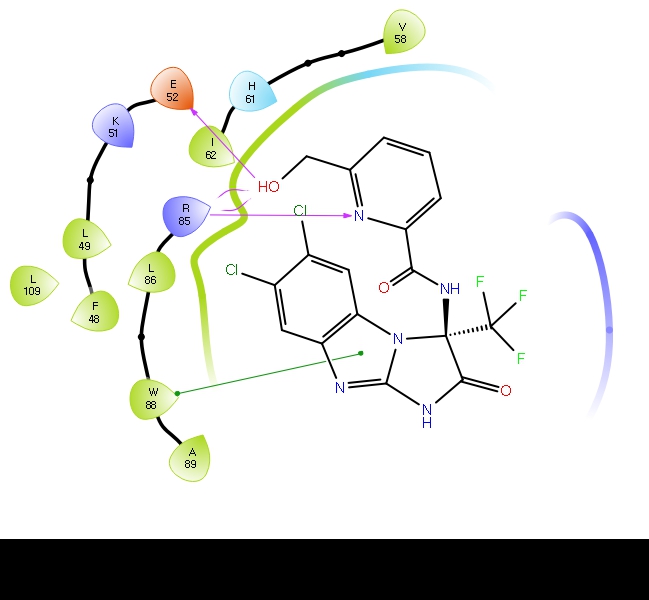

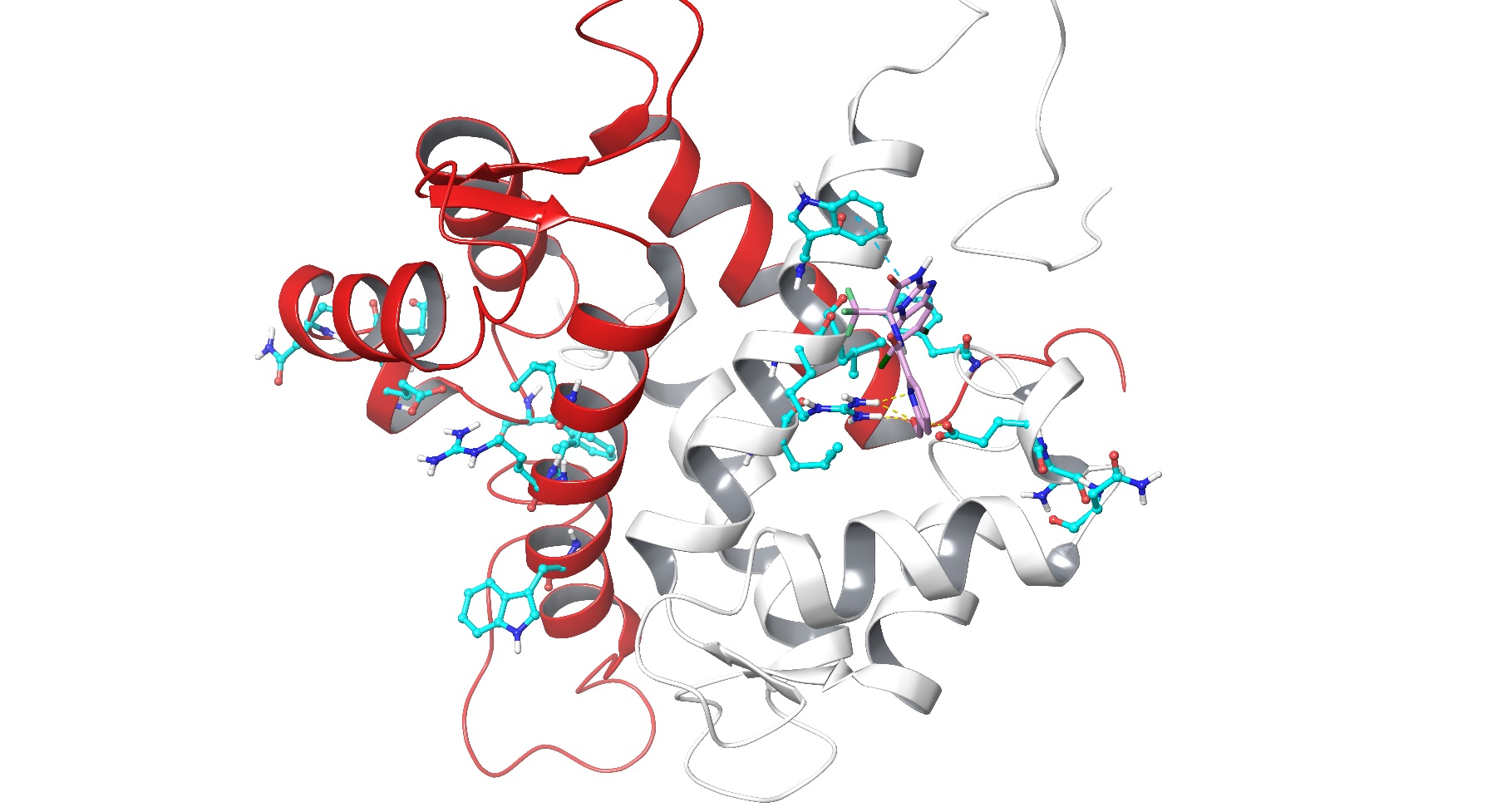


*(a) Red residue: negative charged, blue residue: positive charged, pale green residue: hydrophonic, sky blue: polar, magenta arrow: hydrogen bonding, green line: pi-pi interaction. (b) Two S100A9 were presented by different colored ribbons and key residues are cyan colored ball-and-stick. Cyan dotted line: pi-pi interaction, yellow dotted line: hydrogen bonding.

**Figure S2.** Docking pose of representative compound **110** among 266 known S100A9 inhibitors of Table S1.


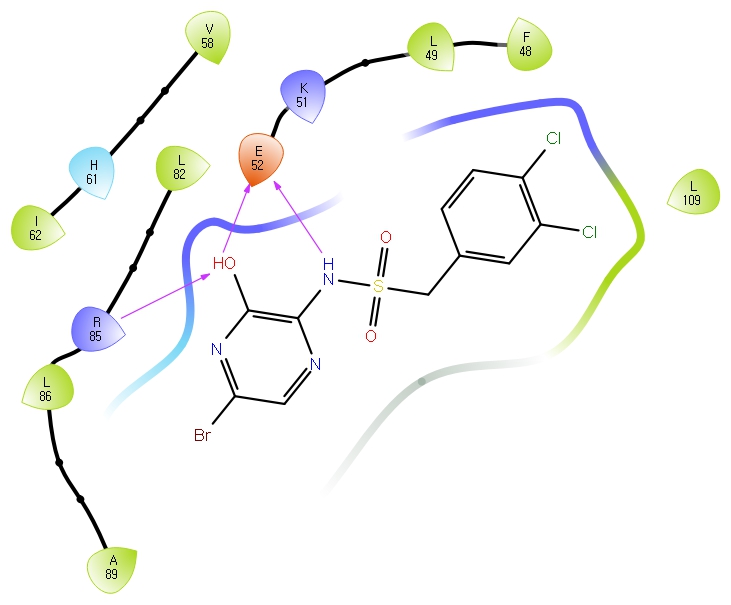

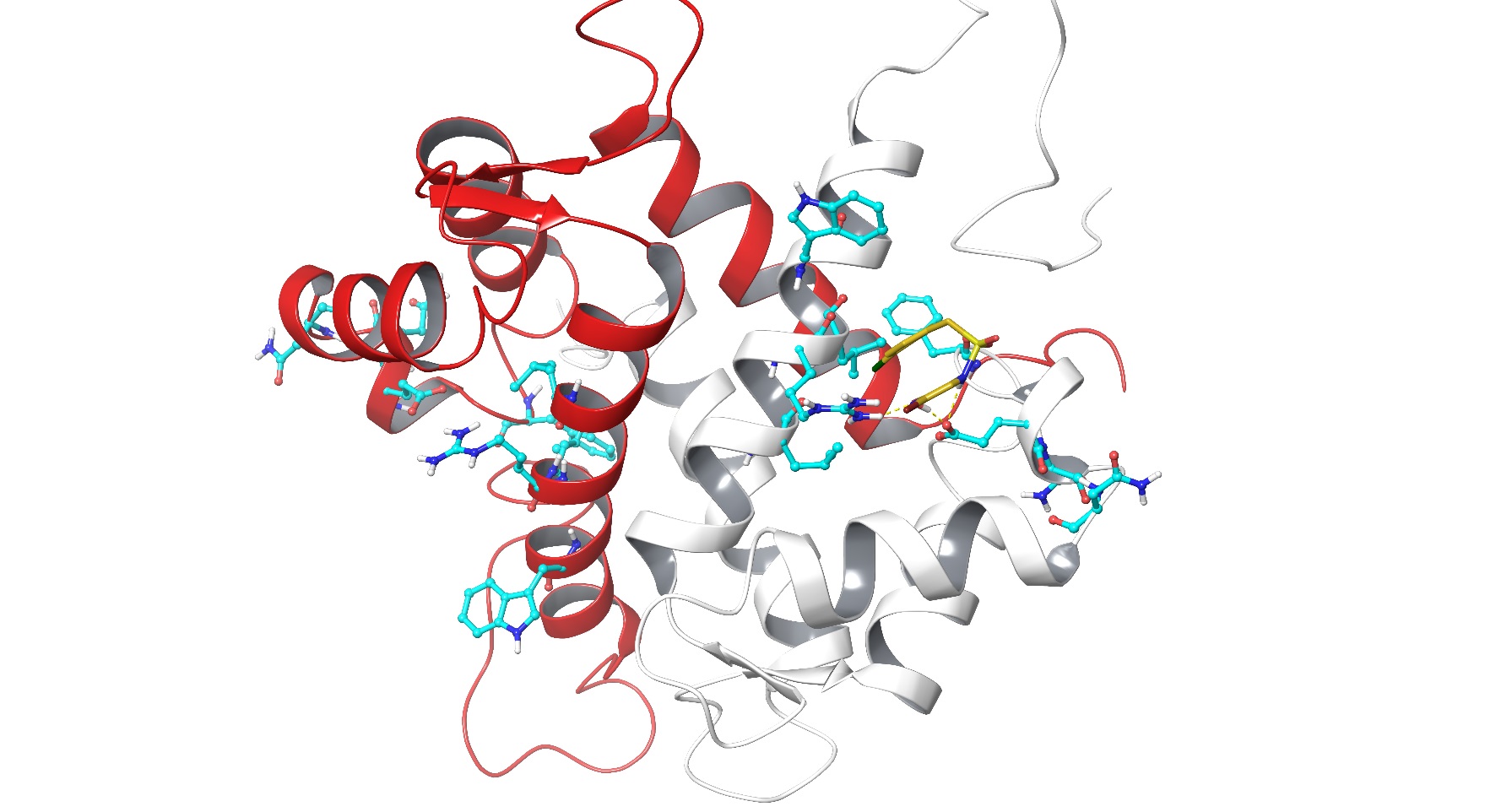


*(a) Red residue: negative charged, blue residue: positive charged, pale green residue: hydrophonic, sky blue: polar, magenta arrow: hydrogen bonding, green line: pi-pi interaction. (b) Two S100A9 were presented by different colored ribbons and key residues are cyan colored ball-and-stick. Cyan dotted line: pi-pi interaction, yellow dotted line: hydrogen bonding.

**Figure S3.** Docking pose of representative compound **227** among 266 known S100A9 inhibitors of Table S1.


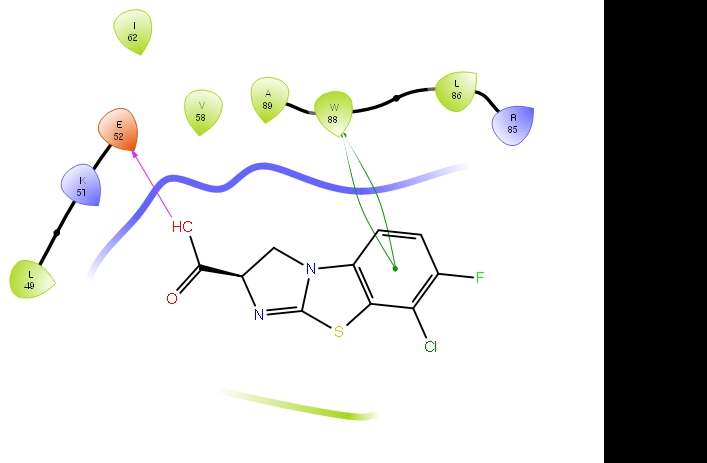

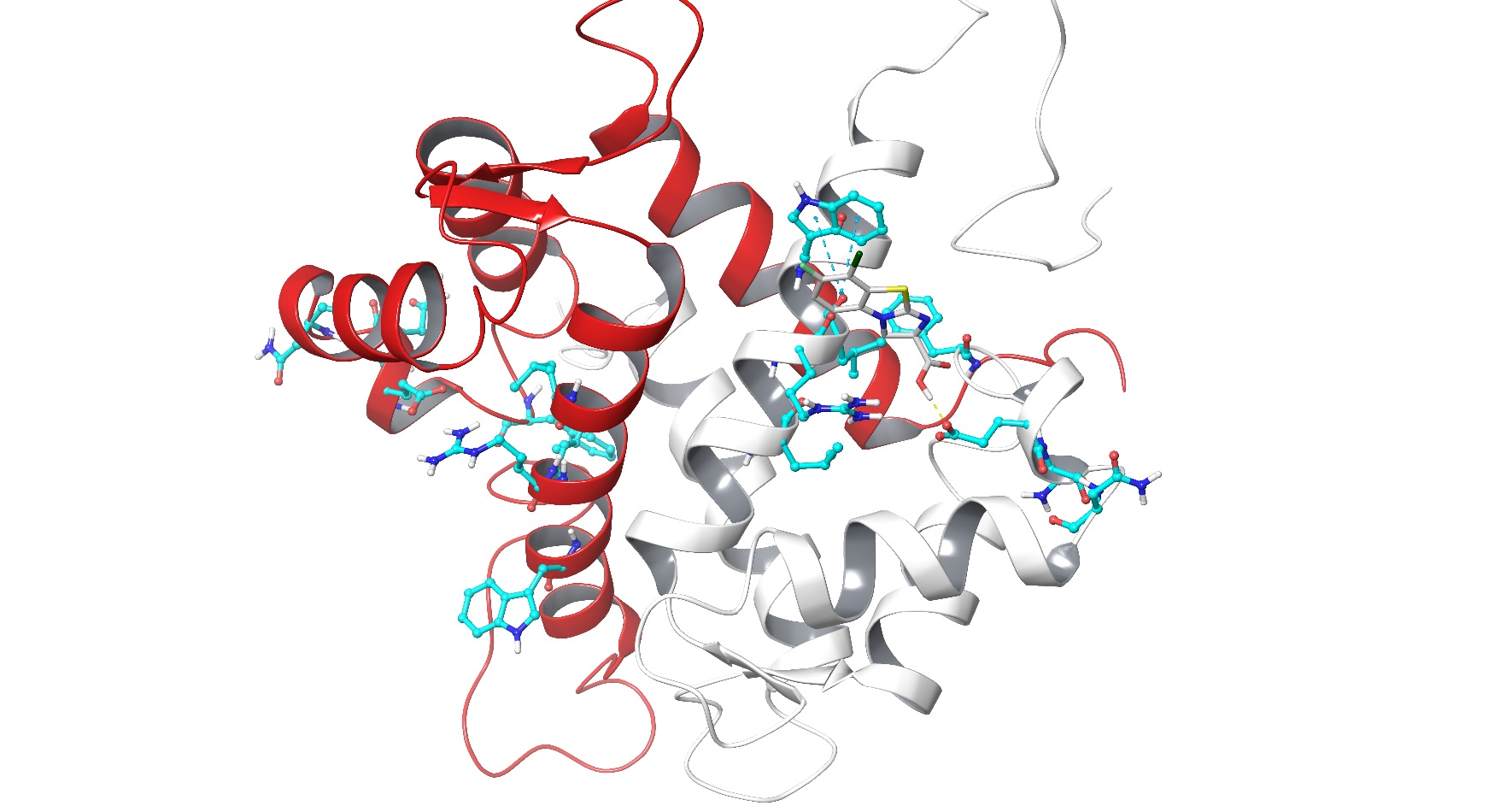


*(a) Red residue: negative charged, blue residue: positive charged, pale green residue: hydrophonic, sky blue: polar, magenta arrow: hydrogen bonding, green line: pi-pi interaction. (b) Two S100A9 were presented by different colored ribbons and key residues are cyan colored ball-and-stick. Cyan dotted line: pi-pi interaction, yellow dotted line: hydrogen bonding.

**Figure S4.** Docking pose of representative compound **1** among 46 hit compounds from Table S3.


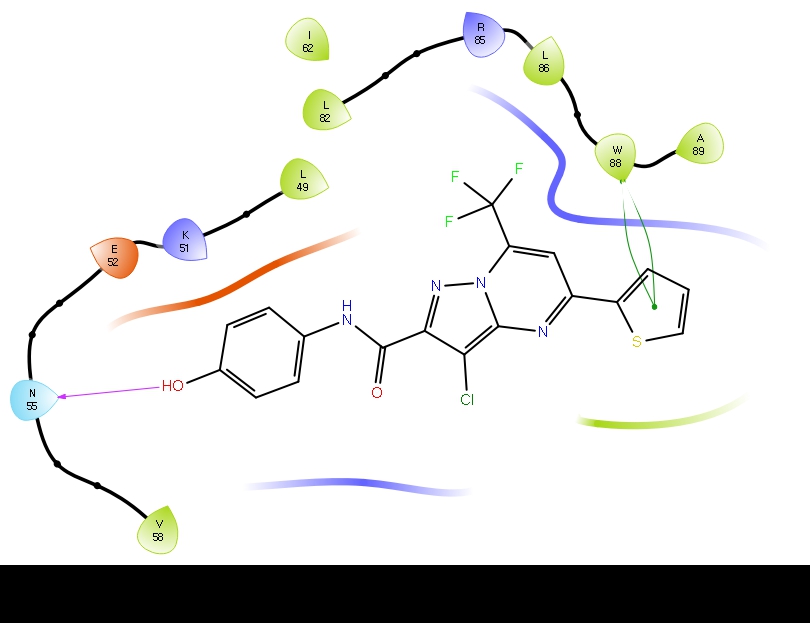

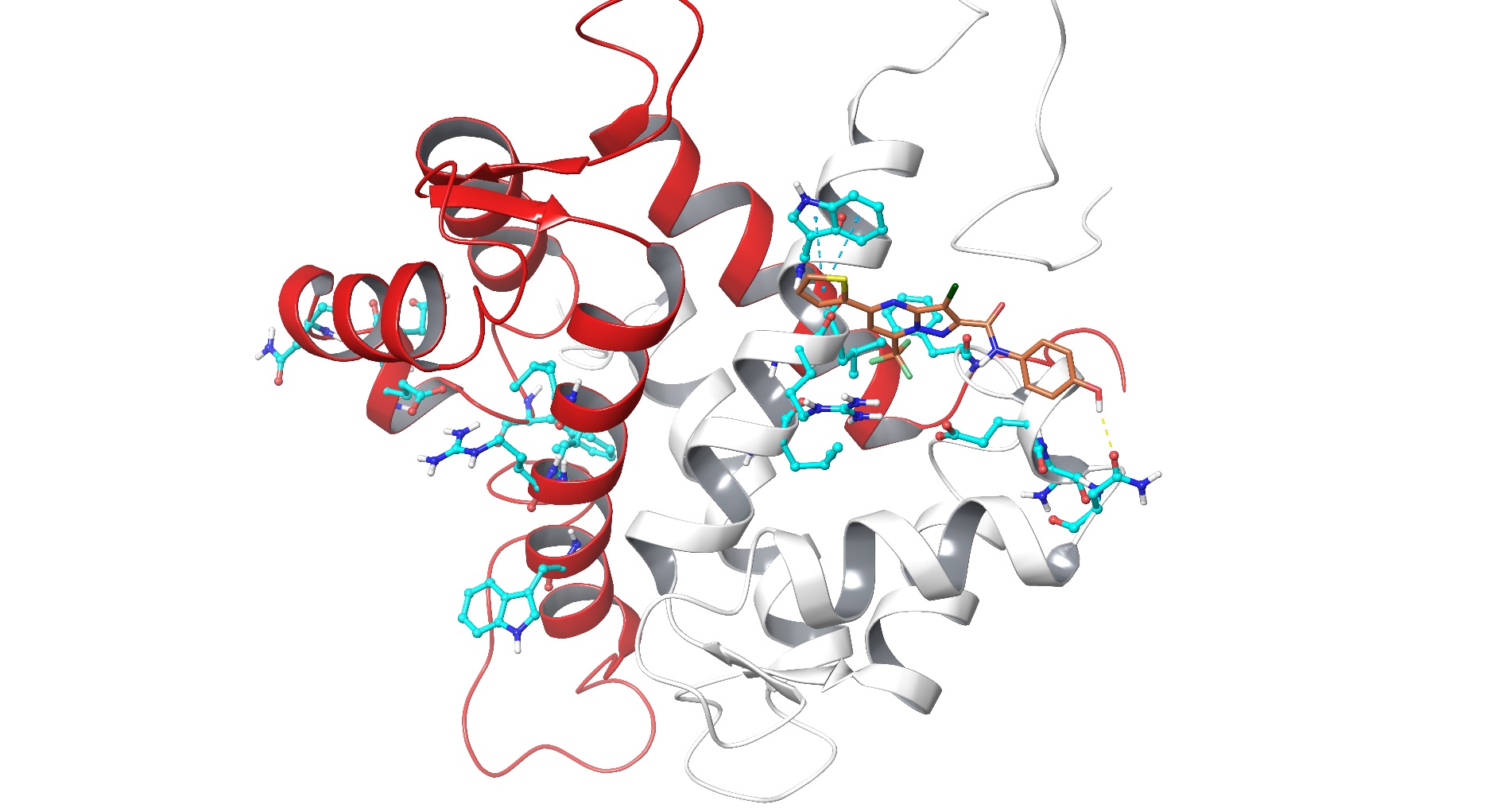


*(a) Red residue: negative charged, blue residue: positive charged, pale green residue: hydrophonic, sky blue: polar, magenta arrow: hydrogen bonding, green line: pi-pi interaction. (b) Two S100A9 were presented by different colored ribbons and key residues are cyan colored ball-and-stick. Cyan dotted line: pi-pi interaction, yellow dotted line: hydrogen bonding.

**Figure S5.** Docking pose of representative compound **32** among 46 hit compounds from Table S3.


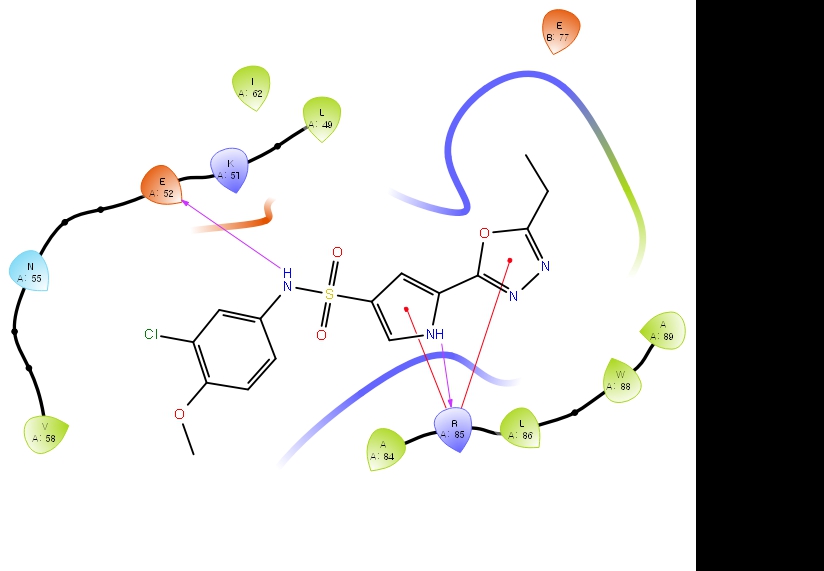

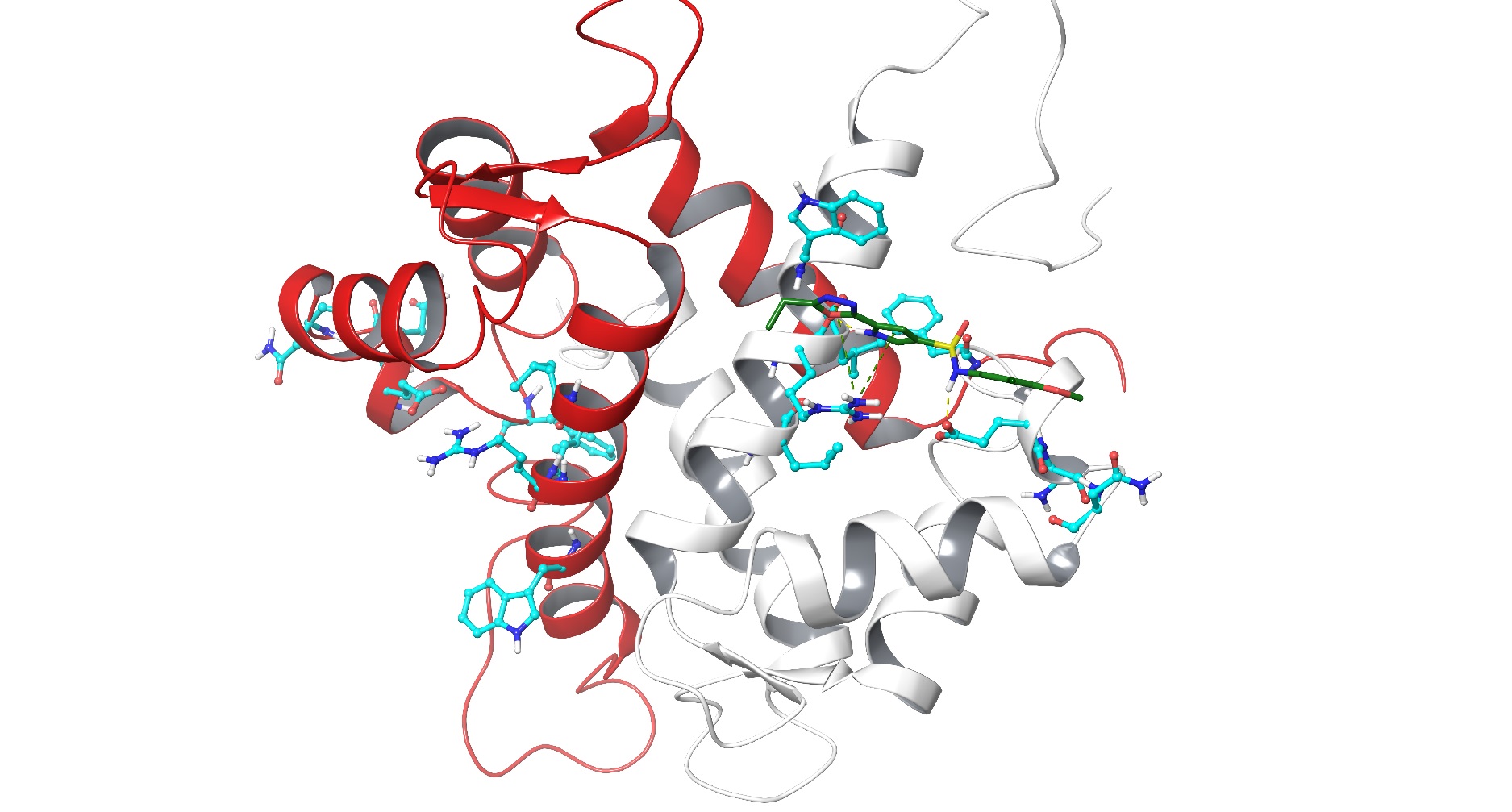


*(a) Red residue: negative charged, blue residue: positive charged, pale green residue: hydrophonic, sky blue: polar, magenta arrow: hydrogen bonding, green line: pi-pi interaction, red line: pi-cation interaction. (b) Two S100A9 were presented by different colored ribbons and key residues are cyan colored ball-and-stick. Cyan dotted line: pi-pi interaction, yellow dotted line: hydrogen bonding.
